# Synthesis of Cyclic Alkenylsiloxanes by Semihydrogenation: A Stereospecific Route to (*Z*)-Alkenyl Polyenes

**DOI:** 10.1002/chem.201403255

**Published:** 2014-06-04

**Authors:** Bryony L Elbert, Diane S W Lim, Haraldur G Gudmundsson, Jack A O'Hanlon, Edward A Anderson

**Affiliations:** [a] Chemistry Research Laboratory, University of Oxford 12 Mansfield Road, Oxford, OX1 3TA, U.K. Fax: (+44) 1865-285002 E-mail: edward.anderson@chem.ox.ac.uk

**Keywords:** alkynes, cross-coupling, hydrogenation, natural products, silanes

## Abstract

Cyclic alkenylsiloxanes were synthesized by semihydrogenation of alkynylsilanes—a reaction previously plagued by poor stereoselectivity. The silanes, which can be synthesized on multigram scale, undergo Hiyama–Denmark coupling to give (*Z*)-alkenyl polyene motifs found in bioactive natural products. The ring size of the silane is crucial: five-membered cyclic siloxanes also couple under fluoride-free conditions, whilst their six-membered homologues do not, enabling orthogonality within this structural motif.

Alkenylsilanes are valuable reagents in organic synthesis.[1] Among many applications, their use in Hiyama–Denmark cross-coupling[2] offers advantages over conventional Stille and Suzuki methods, as organosilanes are non-toxic, stable to a variety of synthetic transformations and can be prepared from inexpensive starting materials. Cyclic alkenylsiloxanes (**1**, Scheme 1)[3] are particularly attractive Hiyama substrates, because they give specific control over the stereochemistry of a (*Z*)-alkene, the geometry of which is “protected” in the cyclic form. Polyenes containing disubstituted (*Z*)-alkenes are common in bioactive natural products, such as the anticancer protein phosphatase inhibitors in Scheme 1,[4] and the efficient synthesis of these motifs is an important goal. Despite this, the cross-coupling of cyclic alkenylsiloxanes has received less attention[5] than their acyclic counterparts,[6, 7] likely due to restrictions in their preparation.[8] The principle approaches employed to date include ring-closing metathesis, which requires the air- and moisture-sensitive Schrock catalyst,[5] intramolecular hydrosilylation, which is limited to internal alkynes,[9] and alkynylsilane enyne metathesis.[10] The latter two methodologies afford trisubstituted alkenes, and are therefore less applicable to natural products of the types depicted in Scheme 1.

**Scheme 1:**
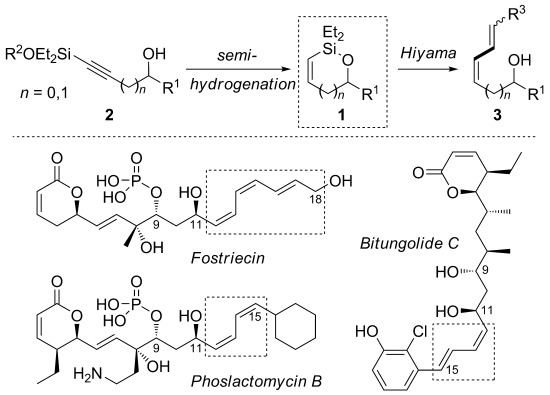
Strategy for the preparation of cyclic alkenylsiloxanes, and examples of (*Z*)-alkene-containing bioactive natural products.

Prominent among methods for the stereoselective formation of disubstituted (*Z*)-alkenes[11] is the semi-reduction of alkynes, typified by Lindlar hydrogenation.[12] Surprisingly, this strategy has not been explored for the synthesis of (*Z*)-alkenylsiloxanes, in which hydrogenation of an alkynylsiloxane **2** (Scheme 2) should give a (*Z*)-alkenylsilane, which in the case of substrate **2** could then undergo intramolecular capture by a proximal alcohol to form **1**. Herein, we report the realization of this chemistry, and the subsequent cross-coupling of these important silanes, including applications to syntheses of the polyene segments of the bioactive natural products in Scheme 1.

**Scheme 2:**
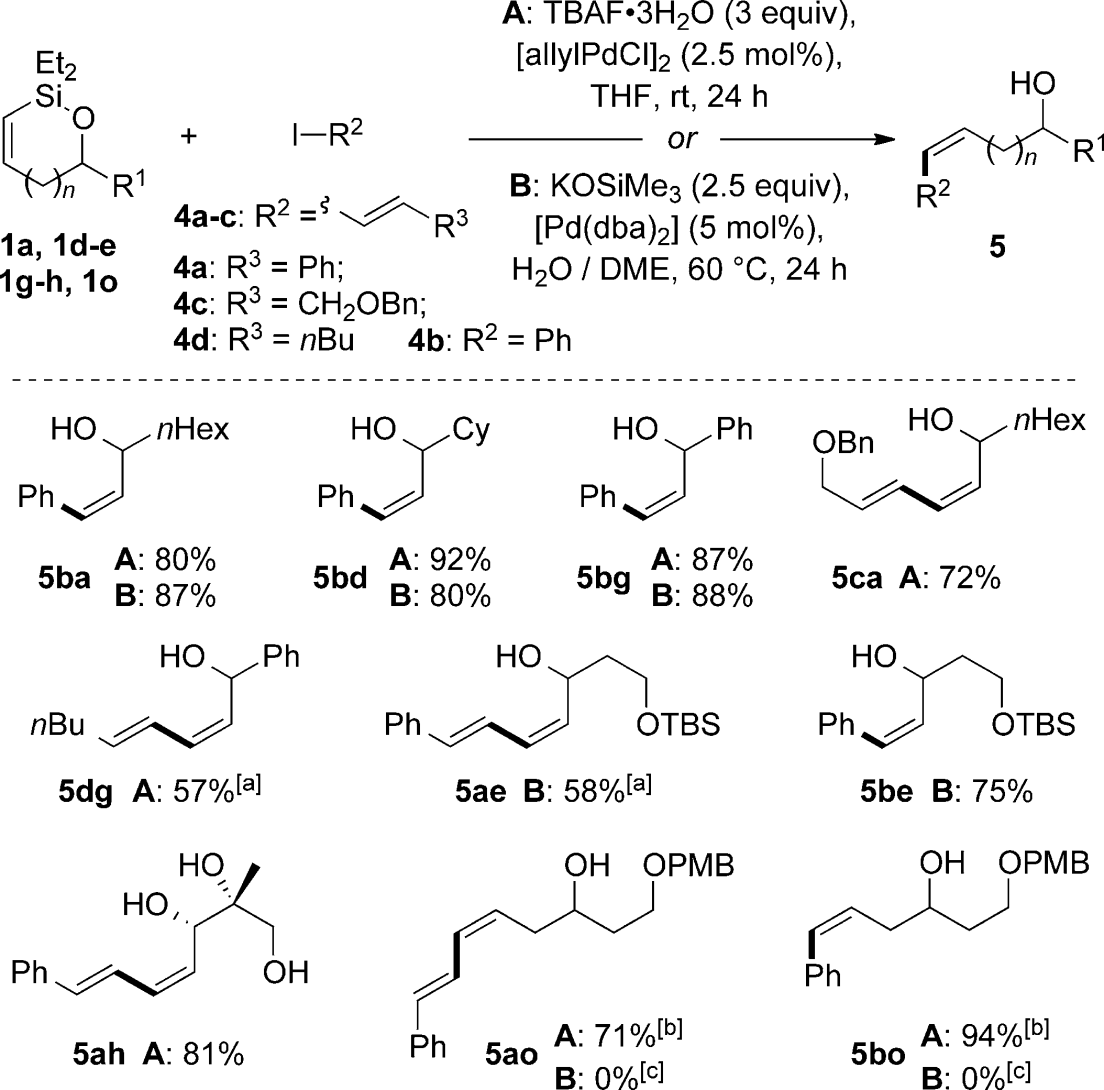
Cross-coupling of cyclic alkenylsiloxanes under fluoride- and base-activated conditions. Reactions performed with one equivalent each of silane/iodide unless otherwise stated. [a] 1.5 equiv iodide was used. [b] Reaction time 48 h. [c] No reaction was observed, see text.

Initial investigations with alkynylsilane **2 a** (Table 1, entry 1) gave unexpectedly high levels of the undesired (*E*)-alkenylsilane **3 a**, and significant over-reduction—a result that correlates with previous reports into the inconsistent selectivity of alkynylsilane hydrogenation.[12] In seeking a solution to this problem, we were drawn to a footnote in a report by Panek and Clark[13] in which hydrogenation of a phenyldimethylsilylalkynyl propargylic acetate exhibited high (*Z*)-selectivity. To our delight, reaction of the acetate derivative **2 b** of our alkynylalkoxysilane (Table 1, entry 2) gave cyclic alkenylsiloxane **1 a** in high yield (75 %, diastereomeric ratio (d.r.)=96:4) following in situ acetate solvolysis/cyclization, with the reaction remaining efficient on multigram scale (Table 1, entry 3). Benzoate ester **2 c** also displayed high selectivity; however, difficulties in the purification of **1 a** from methyl benzoate by-product reduced the isolated yield (entry 4).[14] A solvent screen identified toluene as the optimum choice: alcohol and ester solvents (Table 1, entries 6–9) gave fast reaction times, but were prone to over-reduction, whilst THF led to inconsistent *Z*/*E* ratios on scale-up (entries 10, 11).

**Table 1:** Optimization of the stereoselective semihydrogenation of propargylic alkynylsilanes^[a]^

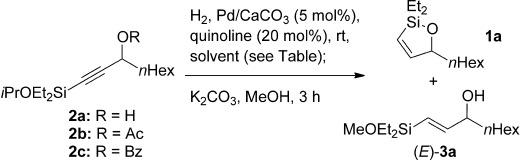					
Entry	Substrate	Solvent	*t*	1 a:(*E)*-3 a^[b]^	Yield [%]^[c]^
1	**2 a**	toluene	8 min	52:48	42
2	**2 b**	toluene	50 min	96:4	75
3	**2 b**	toluene	50 min	96:4^[d]^	80^[d]^
4	**2 c**	toluene	25 min	96:4	20^[e]^
5	**2 b**	benzene	50 min	96:4	73
6	**2 b**	MeOH	8 min	89:11	72^[f]^
7	**2 b**	EtOH	8 min	95:5	75^[f]^
8	**2 b**	*i*PrOH	15 min	93:7	76^[f]^
9	**2 b**	EtOAc	15 min	87:13	66^[f]^
10	**2 b**	THF	1.5 h	95:5	76
11	**2 b**	THF	1.5 h	86:14^[g]^	49^[g]^

[a] Reactions were performed with 0.15 mmol of **2**, 0.1 m, 1 atm H_2_ (balloon). [b] Determined by ^1^H NMR spectroscopic analysis of the crude reaction mixture. [c] Isolated yield. [d] 2.2 g of **2 b**. [e] PhCO_2_Me by-product complicated purification, resulting in lower yield. [f] Variable amounts of over-hydrogenation were observed. [g] 500 mg of **2 b**.

With optimized conditions now in hand, we explored the scope of the hydrogenation/cyclization sequence (Table 2). A wide range of five-membered siloxanes were obtained in high yield, demonstrating the tolerance of branched side chains (**1 d**, Table 2, entry 1), and common protecting groups (**1 e** and **f**, Table 2, entries 2 and 3, the latter being synthesized in enantioenriched form by Noyori transfer hydrogenation of the corresponding ketone).[15] The benzylic product **1 g** (entry 4) proved to be more challenging to obtain, as partial reduction of the benzylic C–O bond was observed. However, employing the benzylic alcohol and more reactive alkynylsilanol (substrate **2 g**) with a lower catalyst loading smoothly accessed **1 g**, providing that cyclohexene was used as a sacrificial co-solvent to prevent over-reduction in this rapid reaction.[16] The heightened reactivity of the silanol over the isopropoxysilane may reflect either a reduced steric effect, or potentially complexation of this free hydroxyl to the catalyst, as has been proposed in other reactions.[17]

**Table 2:** Scope of semihydrogenation of alkynylsilanes^[a]^

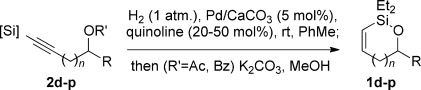				
Entry	Substrate	*t*	Product	Yield [%]^[b]^
1/2	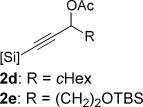	2 h/3 h	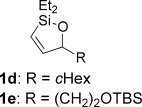	80/80
3	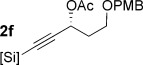	1 h	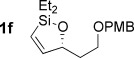	73
4	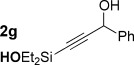	40 min	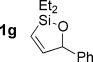	62^[c,d]^
5	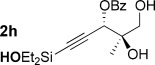	4 h	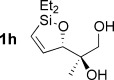	79
6	**2 h**	4 h	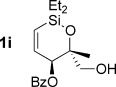	94^[e]^
7	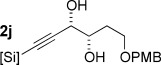	9.5 h	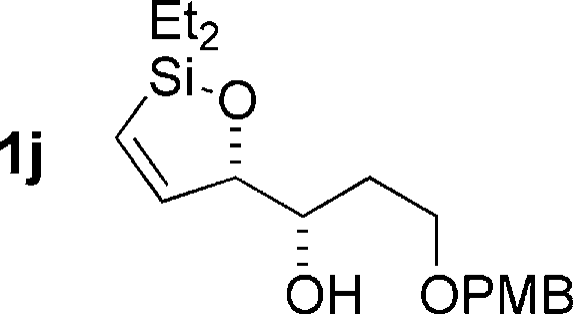	67^[c]^
8	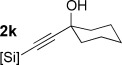	2 h	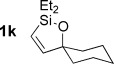	76^[c]^
9/10/11	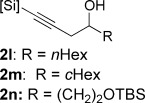	1 h/1 h/15 min	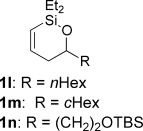	71/66/73
12	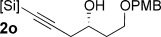	10 min	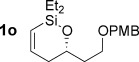	79
13	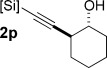	50 min	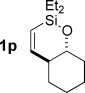	65

[a] Reaction conditions: *n*=0: 20 mol % quinoline; then K_2_CO_3_, MeOH/cyclization. *n*=1: 50 mol % quinoline; then concentrated, MeOH was added; passed through K_2_CO_3_ plug to cyclize. [b] Isolated yield. [c] Cyclohexene was used as co-solvent (PhMe/C_6_H_10_ 10:1). [d] 1 mol % [Pd], 50 mol % quinoline. [e] Cyclized by using PPTS in CH_2_Cl_2_/toluene. [Si]=SiEt_2_O*i*Pr.

Reduction of silanol **2 h** (Table 2, entries 5, 6) illustrates a tolerance of free alcohols in the reaction. This substrate also provides a choice of products: by using a basic methanolysis workup, solely five-membered siloxane **1 h** was obtained, whereas exposure of the crude reaction mixture to mild acidic conditions (pyridinium *p*-toluenesulfonate (PPTS), CH_2_Cl_2_/toluene) instead afforded exclusively the six-membered ring **1 i**. Similar regioselectivity was observed in the case of **2 j**, which gave the five-membered siloxane **1 j** directly, without trace of the six-membered isomer (Table 2, entry 7). These examples demonstrate a predictable differentiation between proximal alcohol functionalities, which could be useful for further manipulations of the cyclic products. It was found that acetylation prior to reduction was unnecessary for homopropargylic or hindered propargylic alcohols; hence, five- and six-membered siloxanes **1 k**–**p** could be obtained in good yields directly from the corresponding alcohols (Table 2, entries 8–13). As the latter reductions again proceeded more rapidly than the esterified substrates, a higher quinoline loading (50 mol %) was required to prevent over-hydrogenation. Siloxane **1 o** was obtained as a single enantiomer from **2 o** (prepared by ring opening of the corresponding non-racemic terminal epoxide)[15] demonstrating that enantioenriched six-membered cyclic siloxanes are also easily accessed.

Having established robust, scalable methods to generate cyclic alkenylsiloxanes, we turned our attention to their application in Hiyama cross-coupling reactions, in which we were particularly intrigued to probe the effect of ring size on reactivity (Table 3). By using Denmark’s conditions for fluoride-activated coupling ([allylPdCl]_2_/tetra-*n*-butylammonium fluoride (TBAF)),[5a,b, 18] we were pleased to find that the reaction of equimolar amounts of siloxane **1 a** and vinyl iodide **4 a** gave allylic diene **5 aa** in good yield, as a single geometrical isomer (entry 1); other catalysts and fluoride sources (entries 2–5)[19] were found to be inferior. We next undertook a study of fluoride-free cross-coupling, which would lead to greater functional group tolerance; to the best of our knowledge, fluoride-free couplings have not been reported for cyclic alkenylsiloxanes of this type.[20, 21] In contrast, a number of conditions have been developed to achieve basic activation of other alkenylsilanes;[2c] selected examples of these, applied to our system, are shown in Table 2 (entries 6–9).[22] The most promising results were obtained with 2.5 equivalents of KOSiMe_3_[23] and 5 mol % [Pd(dba)_2_] in dimethoxyethane (DME), which gave a modest yield of **5 aa** (entry 8), although desilylation (**6**) and silane homocoupling (**7**) were found to compete. Hypothesizing that these by-products might arise from the enhanced basicity of KOSiMe_3_ under anhydrous conditions, we were pleased to find that the addition of 10 equivalents of water to the reaction alleviated these problems (entry 9), albeit requiring heating to 60 °C to attain full conversion. It seems that the success of fluoride-free coupling thus depends crucially on the solvation of the silanolate activator, which is significantly moderated by water.[5c, 24]

**Table 3:** Hiyama cross-coupling of cyclic alkenylsiloxane 1 a^[a]^

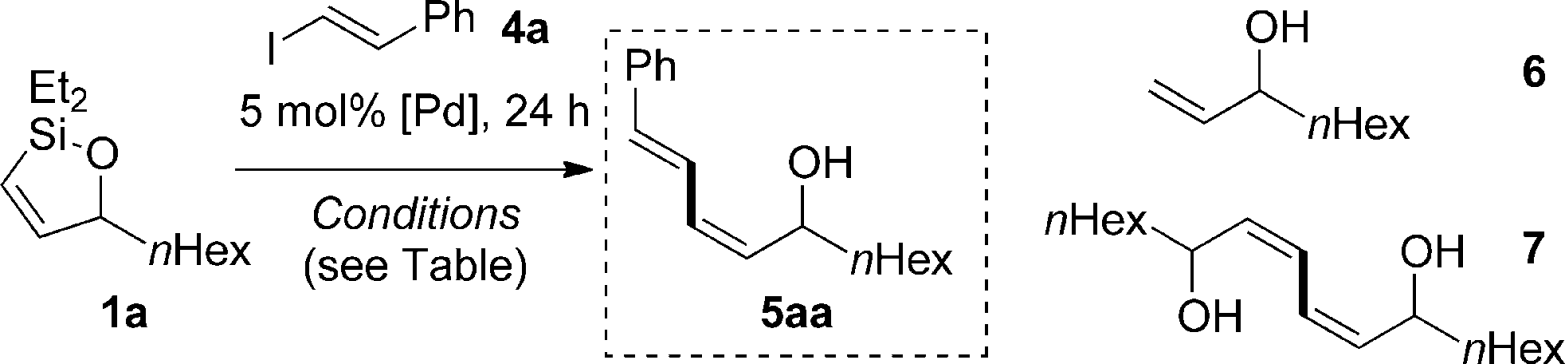					
Entry	[Pd]	Activator (equiv)	Solvent, *T* [°C]	Yield [%]^[b]^	5aa a:6:7^[c]^
1	[allylPdCl]_2_	TBAF**⋅**3 H_2_O (3.0)	THF, RT	72	100:0:0
2	[Pd(dba)_2_]	TBAF**⋅**3 H_2_O (3.0)	THF, RT	65	85:15:0
3	[allylPdCl]_2_	TASF (3.0)	THF, RT	44	99:1:0
4	[allylPdCl]_2_	TBAT (3.0)	THF, RT	0^[d]^	–
5	[allylPdCl]_2_	CsF (3.0)	DMF, 70	47	87:13:0
6	[Pd(PPh_3_)_4_]	Ag_2_O (2.0)	THF, 60	11	30:36:34
7	[allylPdCl]_2_	KOH (3.0)	MeOH, RT	16^[e]^	90:10:0
8	[Pd(dba)_2_]	KOSiMe_3_ (2.5)	DME,^[f]^ RT	47	57:18:25
9	[Pd(dba)_2_]	KOSiMe_3_ (2.5)+10 equiv H_2_O	DME,^[f]^ 60	65	84:11:5

[a] Reaction conditions: 0.22 mmol **1 a**, 0.22 mmol **4 a**, 0.3 m. [b] Isolated yield. [c] Determined by ^1^H NMR spectroscopic analysis of the crude reaction mixture. [d] No reaction. [e]>80 % iodide homocoupling was observed. [f] 0.16 m. TBAF=tetrabutylammonium fluoride; TASF=tris(dimethylamino)sulfonium difluorotrimethylsilicate; TBAT=tetrabutylammonium difluorotriphenylsilicate.

With conditions for both fluoride-promoted and fluoride-free cross-coupling established, a selection of (*Z*)-alkenyl styrenes and dienes were prepared, with variation of siloxane ring size, substituent and iodide coupling partner (Scheme 2). Reaction with iodobenzene gave (*Z*)-allylic alcohols as single isomers in excellent yields with alkyl and aryl-substituted five-membered cyclic siloxanes under both sets of conditions (**5 ba**, **bd**, **bg**).[25] Alkyl-substituted vinyl iodides **4 c** and **d** were also found to be competent coupling partners, giving good yields of *E,Z*-dienes (**5 ca** and **dg**). Notably, the cross-coupling of siloxane **1 e**, which features a primary TBS ether, gave **5 ae** and **be** under fluoride-free conditions without deprotection of the TBS group, demonstrating compatibility with this commonly employed fluoride/acid sensitive functionality.

Although six-membered siloxane **1 o** coupled efficiently with fluoride promotion to give homoallylic alcohols **5 ao** and **bo** in high yield, no cross-coupling was observed under fluoride-free conditions, from which **1 o** was recovered untouched. This result opens exciting possibilities for the orthogonal cross-coupling of five- and six-membered cyclic alkenylsiloxanes,[26] and is likely related to the greater ring strain of the five-membered ring.[14, 27] To definitively illustrate this orthogonality, equimolar amounts of five-membered siloxane **1 f**, six-membered siloxane **1 o** and an iodide were subjected to fluoride-free coupling conditions (Scheme 3). We were delighted to find that both aryl and vinyl iodides reacted selectively with the five-membered substrate **1 f**, with the inert six-membered siloxane returned in high yield in both cases.

**Scheme 3:**
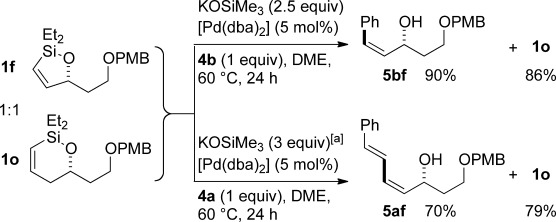
Orthogonal cross-coupling of five-membered silane 1 f in the presence of six-membered silane 1 o (1 equiv each) with PhI (4 b) or 4 a (1 equiv) under fluoride-free conditions. [a] KOSiMe_3_ added in portions.

To underline the synthetic value of this chemistry, we addressed the preparation of the polyene segments of three biologically active natural products featuring (*Z*)-allylic alcohols (see Scheme 1). Although several total syntheses of fostriecin have been reported,[28] the majority have used Stille coupling to assemble the polyene moiety, and/or required the protection of both allylic alcohols. Our methodology (Scheme 4) allowed the construction of the sensitive *Z,Z,E*-triene **8** (C9–C18 fragment of fostriecin) from enantioenriched silane **1 f** and dienyl iodide **4 e**, without the need to protect either allylic alcohol (66 %). Equally pleasing was the success of the challenging cross-coupling of **1 f** with the electron-rich, hindered (*Z*)-vinyl iodide **4 f**, which gave the C9–C21 fragment **9** of phoslactomycin B (52 %).[29, 30] Finally, although much synthetic attention[31] has been focused on the synthesis of (*E*,*E*)-dienes in the bitungolide family, no work on the more challenging C9–C21 portion of bitungolide C, featuring a *Z*,*E*-diene and trisubstituted arene, has been reported to date. We were therefore pleased that **10** could be prepared (without the need for masking of the free phenol), which to our knowledge represents the first synthesis of this bitungolide fragment.

**Scheme 4:**
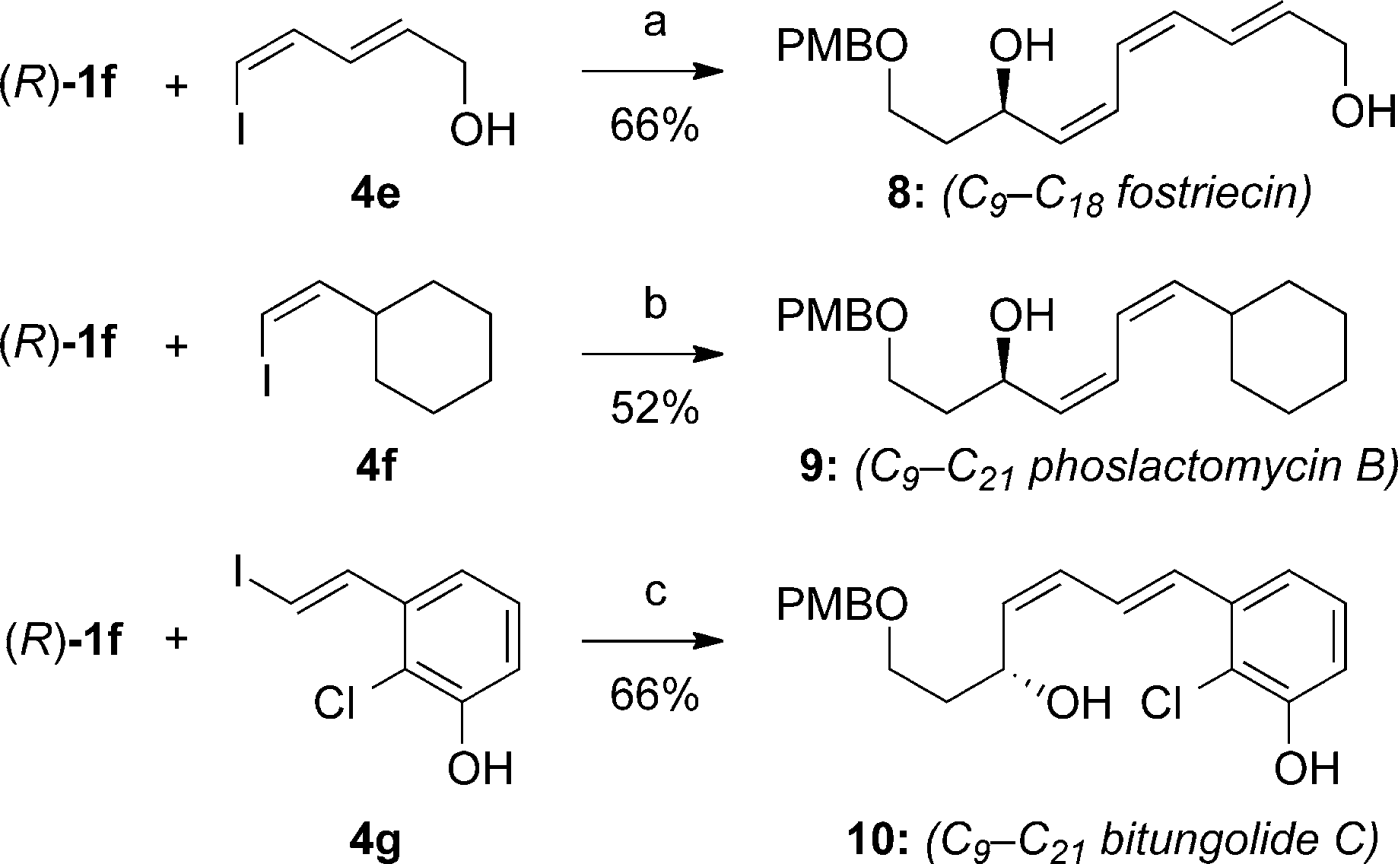
Bioactive natural-product fragments prepared by fluoride-promoted cross-coupling of 1 f. Reaction conditions: common to all: TBAF⋅3 H_2_O (3 equiv), [allylPdCl]_2_ (5 mol %); (a) RT, 48 h; (b) H_2_O (10 equiv), 50 °C, 24 h; c) 50 °C, 24 h.

In summary, we have established a robust, general route to cyclic alkenylsiloxanes based on the Lindlar hydrogenation of alkynylsilane alcohols and esters, which solves a long-standing selectivity issue in alkynylsilane reduction chemistry. Although both five- and six-membered cyclic siloxanes can be stereospecifically cross-coupled to form (*Z*)-alkene-containing products, the additional observation of ring size-dependent orthogonal Hiyama–Denmark reactivity under base-promoted conditions offers exciting synthetic opportunities. The preparation of the diene/triene segments of representative anticancer polyketide natural products illustrates the value and applicability of this chemistry in organic synthesis.

## Experimental Section

### Semihydrogenation of propargylic acetates

Palladium on CaCO_3_ (5 wt % Pd, 0.05 equiv) was added to a stirred solution of acetate (1.0 equiv) and quinoline (0.2 equiv) in toluene (0.1 m). The resulting solution was stirred under an atmosphere of hydrogen (balloon) for the indicated period until completion as monitored by TLC. The mixture was then filtered through Celite and concentrated. The crude residue was redissolved in methanol, then K_2_CO_3_ (2–3 equiv) was added, and the mixture was stirred vigorously for 3 h. The reaction was then diluted with Et_2_O, washed twice with water, dried (MgSO_4_) and concentrated. The residue was purified by rapid flash column chromatography on a short column of silica gel to give the oxasiloles as colourless oils, which are sensitive to silica gel. Typically, 4–5 cm of silica gel (or 8–9 g mmol^−1^ of crude) was employed, and the crude mixture was loaded onto a layer of sand (2–3 cm) prior to elution (petroleum ether/Et_2_O 19:1).

### Fluoride-promoted cross-coupling

A degassed solution of TBAF**⋅**3 H_2_O (1 m solution in THF, 3.0 equiv) was added to the silane (1.0 equiv), iodide (1.0 equiv) and allylpalladium chloride dimer (0.025 equiv) at room temperature. The mixture was stirred for 24–48 h in the dark, then the reaction was diluted with CH_2_Cl_2_ and filtered through a plug of silica gel. The filtrate was concentrated, and the residue purified by flash-column chromatography to give the cross-coupled product.

### Base-promoted cross-coupling

A degassed solution of potassium trimethylsilanolate (98 wt %, as a 0.42 m solution in DME, 2.5 equiv) was added to the silane (1.0 equiv), iodide (1.0 equiv), water (10.0 equiv) and bis(dibenzylideneacetone)palladium (0.05 equiv) at room temperature. The mixture was heated to 60 °C for 24 h in the dark, then it was cooled to room temperature, diluted with Et_2_O and filtered through a plug of silica gel. The filtrate was concentrated, and the residue purified by flash-column chromatography to afford the cross-coupled product.

## Supporting Information

As a service to our authors and readers, this journal provides supporting information supplied by the authors. Such materials are peer reviewed and may be re-organized for online delivery, but are not copy-edited or typeset. Technical support issues arising from supporting information (other than missing files) should be addressed to the authors.

miscellaneous_information
