# Supplementary material for: Synthesis of Cyclic Alkenylsiloxanes by Semihydrogenation: A Stereospecific Route to (Z)-Alkenyl Polyenes
Source: Chemistry. 2014 Jun 4;20(28):8594–8. doi: 10.1002/chem.201403255 (PMC4531823; doi:10.1002/chem.201403255)
Supplement: Supplementary file 1 — miscellaneous_information [file chem0020-8594-sd1.pdf]

CHEMISTRY
A European Journal

Supporting Information

© Copyright Wiley-VCH Verlag GmbH & Co. KGaA, 69451 Weinheim, 2014

Synthesis of Cyclic Alkenylsiloxanes by Semihydrogenation: A Stereospecific Route to (*Z*)-Alkenyl Polyenes

Bryony L. Elbert, Diane S. W. Lim, Haraldur G. Gudmundsson, Jack A. O'Hanlon, and Edward A. Anderson*[a]

chem_201403255_sm_miscellaneous_information.pdf

Supporting Information

Contents

1. Experimental **2**

1.1 General Experimental 2

1.2 General Experimental Procedures 4

1.2.1 General Procedure A: Preparation of Propargylic Alcohols **4**

1.2.2 General Procedure B: Preparation of Homopropargylic Alcohols **4**

1.2.3 General Procedure C: Acetylation of Propargylic Alcohols **4**

1.2.4 General Procedure D: Preparation of Cyclic 5-Membered Alkenylsiloxanes from Acetate Substrates **5**

1.2.5 General Procedure E: Preparation of Cyclic Alkenylsiloxanes from Alcohol Substrates **5**

1.2.6 General Procedures for Cross-Coupling of Cyclic Alkenylsiloxanes **5**

1.3 Preparation of Alkynylsilane S2 7

1.4 Preparation of Hydrogenation Substrates 9

1.4.1 General Scheme for Preparation of Hydrogenation Substrates 2a – 2p, 2z **9**

1.5 Hydrogenation of Alkynylsilanes 40

1.6 Synthesis of Iodide Coupling Partners 51

1.6.1 General Scheme for Synthesis of Iodide Coupling Partners **51**

1.7 Cross-Coupling of Cyclic Siloxanes 62

1.8 Cross-Coupling of Acyclic Siloxanes 77

2. NMR Data of Novel Compounds **78**

3. References **137**

1. Experimental

1.1 General Experimental

Solvents and Reagents. Dichloromethane, tetrahydrofuran, toluene and methanol were obtained anhydrous from solvent dispenser units having been passed through an activated alumina column under argon. Dimethylsulfoxide, 1,2-dimethoxyethane and propane-1,3-diol were distilled over calcium hydride under reduced pressure prior to use. Diethylamine was distilled from calcium hydride and stored under nitrogen over 3 Å molecular sieves. Benzaldehyde was washed with saturated $NaHCO_3$ solution and dried ($MgSO_4$) prior to use. All other reagents were used as received. Tetrabutylammonium fluoride (TBAF) solution was obtained by dissolving tetrabutylammonium fluoride trihydrate in anhydrous THF. TBAF•$3H_2O$, scandium(III) triflate and potassium trimethylsilanolate were weighed out in a glove box under nitrogen. Potassium trimethylsilanolate was purchased from CombiBlocks. Petrol refers to the fraction of petroleum ether which boils in the range 40-60 ºC. Et_2O refers to diethyl ether. EtOAc refers to ethyl acetate. Brine refers to a saturated aqueous solution of NaCl. $NaHCO_3$, K_2CO_3, NH_4Cl and $Na_2S_2O_3$ solutions refer to saturated aqueous solutions. HCl was also used as an aqueous solution.

Reactions. All reactions were carried out under argon or nitrogen unless otherwise stated. Oven-dried glassware was used for reactions requiring anhydrous conditions.

Chromatography. Thin-layer chromatography was performed on Merck aluminium-backed DC 60 F254 0.2 mm precoated plates, which were visualised with UV fluorescence and staining with potassium(VII) manganate or vanillin. Flash column chromatography was performed on MN Kieselgel 60M (particle size 40-63 µm) with solvent system used in parentheses.

Melting points. Melting points were determined using a Griffin melting point apparatus and are uncorrected.

Infrared Spectroscopy. Infrared spectra were recorder on a Bruker Tensor 27 Fourier transform spectrometer, as a thin film on a diamond ATR module.

NMR Spectroscopy. ^{1}H NMR spectra were recorded at 200, 250, 400 or 500 MHz on a Bruker DPX 200, Bruker DPX 250, Bruker DPX 400, Bruker DQX400, Bruker AVN 400, Bruker DRX500 and Bruker AVII 500, respectively. ^{13}C NMR spectra were recorded at 101 MHz or 125 MHz on a Bruker DQX 400, Bruker AVN 400, Bruker DRX500 or a Bruker ACII 500 with ^{13}C cryoprobe, respectively. Chemical shifts (δ_H and δ_C) are expressed in parts per million (ppm), referenced to the residual solvent peak of $CDCl_3$ or C_6D_6. Coupling constants (*J*) are reported to the nearest 0.1 Hz. Spectra are assigned based on chemical shift, coupling constants, COSY, HSQC and HMBC data and comparision with similar compounds. Splitting patterns are described using the following abbreviations: s (singlet), d (doublet), t (triplet), q (quartet), quin. (quintet), sept. (septet).

Mass spectroscopy. Low-resolution mass spectra (*m/z*) were performed on a Micromass LCT Premier Open Access. High-resolution mass spectra were recorded under ES or EI conditions on a Bruker MicroTOF.

Elemental Analysis. Samples were analysed by Mr. Stephen Boyer, Science Centre, London Metropolitan University.

1.2 General Experimental Procedures

1.2.1 General Procedure A: Preparation of Propargylic Alcohols

n-Butyllithium (2.5 M solution in hexanes, 1.1 equiv.) was added to a solution of silyl alkyne **S2** (85 wt% purity, 1.0 equiv.) in THF at –78 ºC, and the reaction was stirred for 1 h. The aldehyde (1.0 equiv.) was added dropwise at –78 ºC and the mixture stirred for a further 2-3 h. The reaction was quenched with NH_4Cl solution (sat., aq.), and the aqueous layer extracted three times with Et_2O and dried ($MgSO_4$). The residue was concentrated *in vacuo* and purified *via* flash column chromatography.

1.2.2 General Procedure B: Preparation of Homopropargylic Alcohols

Prepared according to a modified literature procedure.[1] *n*-Butyllithium (2.5 M solution in hexanes) was added to a solution of silyl alkyne **S2** (85 wt% purity, 1.0 equiv.) in THF, and the reaction was stirred at –78 ºC for one hour. Epoxide (1.0 equiv.) was added, and the mixture stirred for a further 10 minutes before $BF_3•OEt_2$ was added. After stirring for the specified time, the reaction was quenched with NH_4Cl solution (sat., aq.), extracted with Et_2O, dried ($MgSO_4$) and concentrated *in vacuo*. The residue was purified *via* flash column chromatography.

1.2.3 General Procedure C: Acetylation of Propargylic Alcohols

Acetic anhydride (2.0 equiv.) was added dropwise to a given propargyl alcohol (1.0 equiv.), one crystal of DMAP and Et_3N (3.0 equiv.) in CH_2Cl_2. The mixture was stirred for three hours, and then quenched with $NaHCO_3$ (sat., aq.). The aqueous layer was extracted three times with CH_2Cl_2 and the combined organic layers dried ($MgSO_4$) and concentrated to give the acetate as an oil that was used in the next step without further purification.

1.2.4 General Procedure D: Preparation of Cyclic 5-Membered Alkenylsiloxanes from Acetate Substrates

Palladium on $CaCO_3$ (5 wt% Pd, 0.05 equiv.) was added to a stirred solution of acetate (1.0 equiv.) and quinoline (0.2 equiv.) in toluene. The resulting solution was stirred under an atmosphere of hydrogen (using a balloon and 19G needle through a rubber septum) for 1-3 hours until complete as monitored by TLC (visualised with vanillin). The mixture was then filtered through Celite® and concentrated. The crude residue was redissolved in methanol, K_2CO_3 (2-3 equiv.) was added, and the mixture was stirred vigorously for 3 h. The reaction was then diluted with Et_2O, washed twice with water, dried ($MgSO_4$) and concentrated *in vacuo*. The residue was purified by **rapid** flash column chromatography on a short column of silica gel to give the oxasilole as a colourless oil which is sensitive to silica gel. Typically 4-5 cm of silica gel (or 8-9 g / mmol of crude) was employed, and the crude mixture was loaded onto a thick layer of sand (2-3 cm) prior to elution (petrol / Et_2O (19:1)).

1.2.5 General Procedure E: Preparation of Cyclic Alkenylsiloxanes from Alcohol Substrates

Palladium on $CaCO_3$ (5 wt% Pd, 0.05 equiv.) was added to a stirred solution of alcohol (1.0 equiv.) and quinoline (0.2-05 equiv.) in toluene. The resulting solution was stirred under an atmosphere of hydrogen (using a balloon and 19G needle through a rubber septum) until complete as judged by TLC (visualised with vanillin). The mixture was then filtered through Celite® and concentrated. The crude residue was redissolved in methanol, and filtered through a plug of K_2CO_3. The mixture was diluted with Et_2O, washed twice with water, dried ($MgSO_4$) and concentrated *in vacuo*. The residue was purified by **rapid** flash column chromatography on a short column of silica gel (petrol / Et_2O (19:1)) to give the cyclic siloxane as a colourless oil which is unstable towards prolonged exposure to silica gel.

1.2.6 General Procedures for Cross-Coupling of Cyclic Alkenylsiloxanes

Procedure F: A degassed solution of TBAF•$3H_2O$ (1 M solution in THF, 3.0 equiv.) was added to the silane (1.0 equiv.), iodide (1.0 equiv.) and allylpalladium chloride

dimer (0.025 equiv.) at room temperature. The mixture was stirred for 24-48 h in the dark, then diluted with CH_2Cl_2 and filtered through a plug of silica gel. The filtrate was concentrated and purified by flash column chromatography.

Procedure G: A degassed solution of potassium trimethylsilanolate (98 wt%, 0.42 M in DME, 2.5 equiv.) was added to the silane (1.0 equiv.), iodide (1.0 equiv.), water (10.0 equiv.) and bis(dibenzylideneacetone)palladium (0.05 equiv.) at room temperature. The mixture was heated to 60 °C and for 24 h in the dark, then it was diluted with Et_2O and filtered through a plug of silica gel. The crude mixture was concentrated and the residue was purified by flash column chromatography.

1.3 Preparation of Alkynylsilane S2

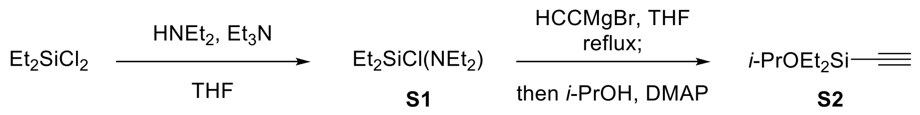

Diethyl(diethylamino)chlorosilane, S1

$ClSiEt_2(NEt_2)$

To a solution of dichlorodiethylsilane (10.0 mL, 67.5 mmol, 1.0 equiv.) and Et_3N (10.3 mL, 74.2 mmol, 1.1 equiv.) in anhydrous THF (10 mL) at 0 ºC was added a solution of Et_2NH (7.05 mL, 67.5 mmol, 1.0 equiv.) in THF (7 mL) over 2 h. The mixture was stirred at room temperature for 16 h before the solvent was removed *in vacuo,* and the crude residue redissolved in anhydrous pentane. The solution was filtered through Celite® under nitrogen to remove hydrochloride salts, washing with anhydrous pentane, then the filtrate was concentrated *in vacuo*. The residue was then distilled under vacuum to afford the title compound as a colourless oil (11.5 g, 59.3 mmol, 88%); **bp** 92-94 ºC, 36 mbar (lit.[2] 104-106 ºC, 56 mmHg); **^{1}H NMR** (200 MHz, $CDCl_3$) δ_H 2.89 (4H, q, J = 7.0 Hz, N(CH_2CH$_3$)$_2$), 1.08-0.99 (12H, m, Si(CH$_2$CH_3)$_2$ and N(CH$_2$CH_3)$_2$), 0.91 (4H, q, J = 6.3 Hz, Si(CH_2CH$_3$)$_2$); **^{13}C NMR** (101 MHz, $CDCl_3$) δ_C 39.5, 15.1, 7.4, 6.9.

The physical and spectroscopic data were found to be in agreement with that reported by Cox and co-workers.[2]

Diethyl(ethynyl)(isopropoxy)silane, S2

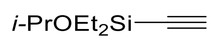

Ethynylmagnesium bromide (253 mL of a 0.5 M solution in THF, 127 mmol, 1.1 equiv.) was added to **S2** (22.3 g, 115 mmol, 1.0 equiv.) at –78 ºC and warmed to room temperature. The mixture was refluxed for 3 h, then cooled to room temperature

whereupon isopropanol (17.5 mL, 230 mmol, 2.0 equiv.) and one crystal of DMAP were added and the reaction left to stir at room temperature for 16 h. The mixture was concentrated *in vacuo*, the residue was redissolved in petrol, and filtered through Celite®. The filtrate was concentrated *in vacuo* and the residue distilled to afford a 5:1 mixture of the title compound **S2** : diethyldiisopropoxysilane as a colourless oil (12.1 g, 59.0 mmol, 83% purity by weight, 51%); **R_f** 0.61 (petrol / Et_2O (9:1)); **bp** 50-52 ºC, 20 mbar (lit.[3] 53 ºC, 15 Torr); **^{1}H NMR** (400 MHz, $CDCl_3$) δ_H 4.18 (1H, sept, J = 6.1 Hz, OCH(CH_3)$_2$), 2.43 (1H, s, C≡CH), 1.21 (6H, d, J = 6.1 Hz, OCH(CH_3)$_2$), 1.02 (6H, t, J = 7.8 Hz, Si(CH_2CH_3)$_2$), 0.68 (4H, q, J = 7.8 Hz, Si(C$H_2$$CH_3$)$_2$); **^{13}C NMR** (101 MHz, $CDCl_3$) δ_C 93.6, 86.5, 66.2, 25.4, 6.6, 6.4.

The physical data were found to be in agreement with that reported by Voronkov and co-workers.[3]

1.4 Preparation of Hydrogenation Substrates

1.4.1 General Scheme for Preparation of Hydrogenation Substrates 2a – 2p, 2z

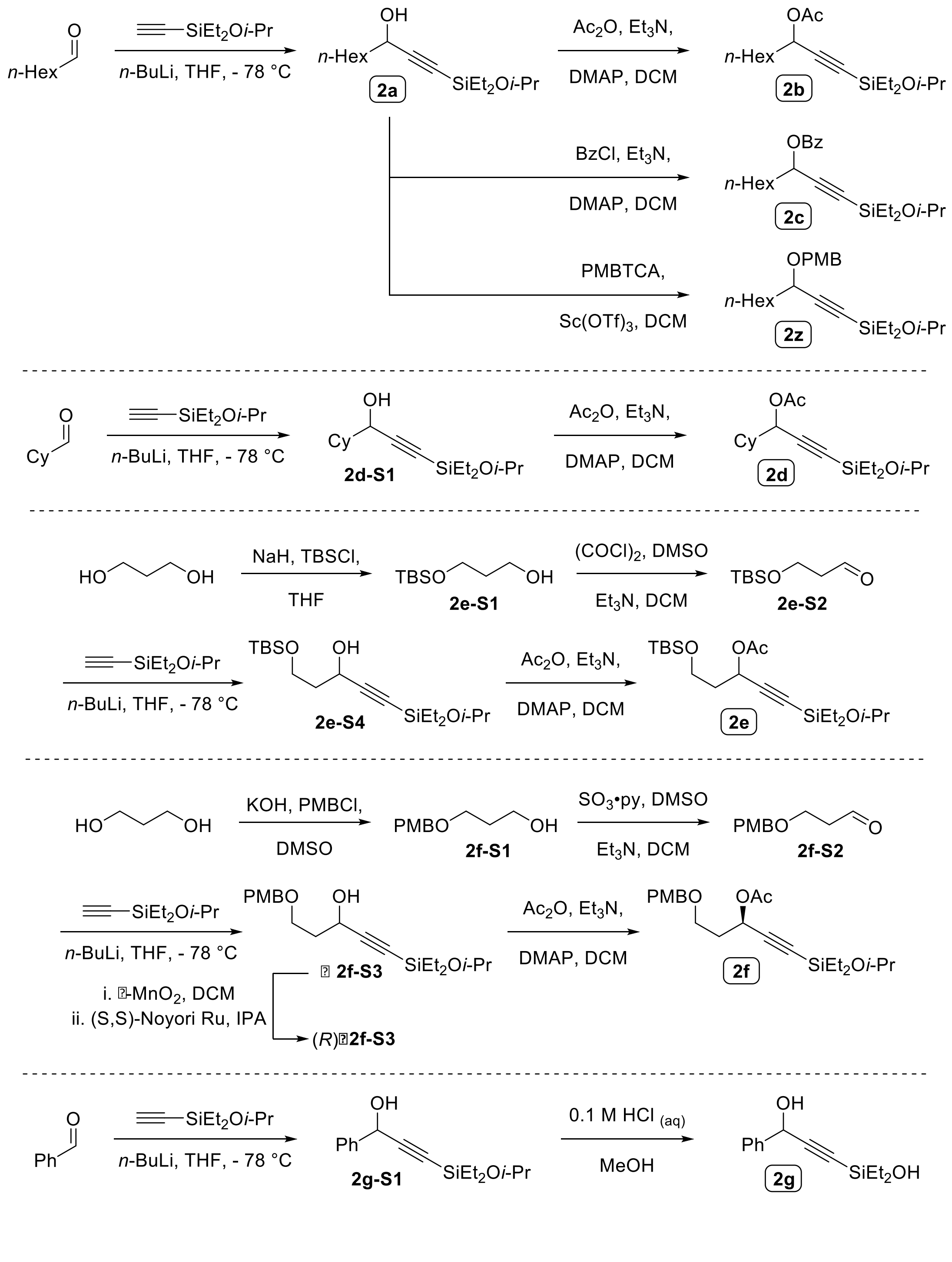

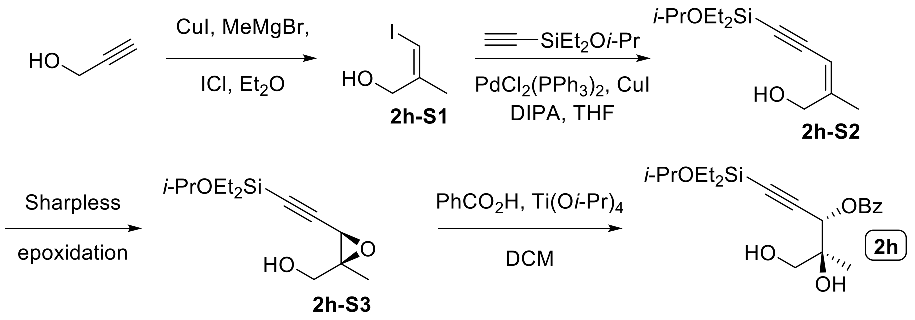
HO
CuI, MeMgBr,
ICl, Et2O
I
HO
2h-S1
SiEt2Oi-Pr
PdCl2(PPh3)2, CuI
DIPA, THF
i-PrOEt2Si
HO
2h-S2
Sharpless
epoxidation
i-PrOEt2Si
O
HO
2h-S3
PhCO2H, Ti(Oi-Pr)4
DCM
i-PrOEt2Si
OBz
HO
OH
2h

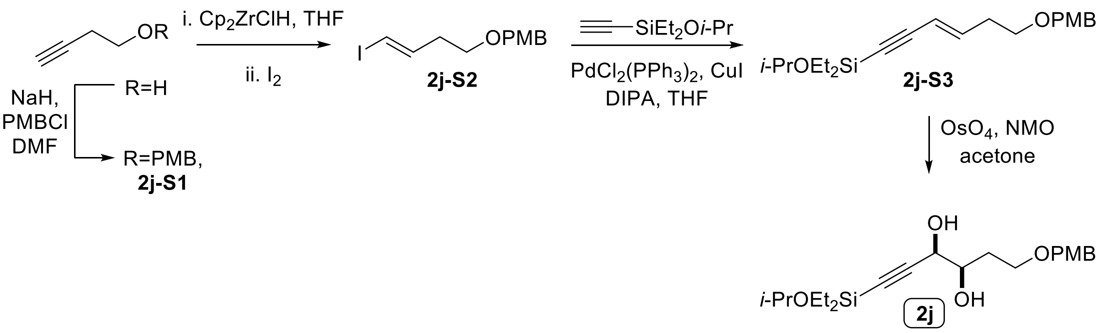
OR
NaH,
PMBCl
DMF
R=H
R=PMB,
2j-S1
i. Cp2ZrClH, THF
ii. I2
I
OPMB
2j-S2
SiEt2Oi-Pr
PdCl2(PPh3)2, CuI
DIPA, THF
i-PrOEt2Si
OPMB
2j-S3
OsO4, NMO
acetone
OH
OPMB
i-PrOEt2Si
OH
2j

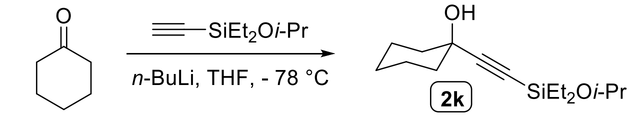
O
SiEt2Oi-Pr
n-BuLi, THF, - 78 °C
OH
SiEt2Oi-Pr
2k

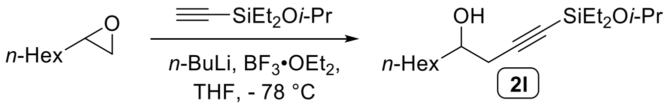
n-Hex
O
SiEt2Oi-Pr
n-BuLi, BF3•OEt2,
THF, - 78 °C
OH
SiEt2Oi-Pr
n-Hex
2l

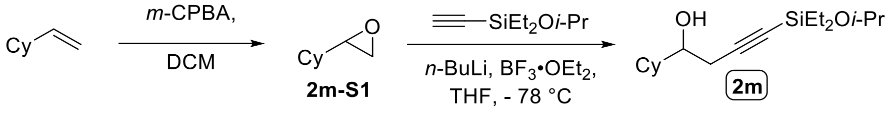
Cy
m-CPBA,
DCM
Cy
O
2m-S1
SiEt2Oi-Pr
n-BuLi, BF3•OEt2,
THF, - 78 °C
OH
SiEt2Oi-Pr
Cy
2m

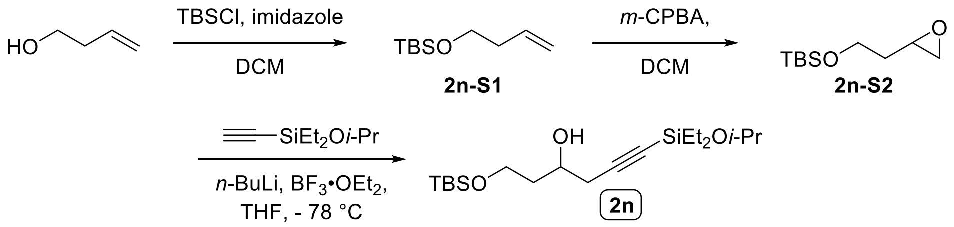
HO
TBSCl, imidazole
DCM
TBSO
2n-S1
m-CPBA,
DCM
TBSO
O
2n-S2
SiEt2Oi-Pr
n-BuLi, BF3•OEt2,
THF, - 78 °C
OH
SiEt2Oi-Pr
TBSO
2n

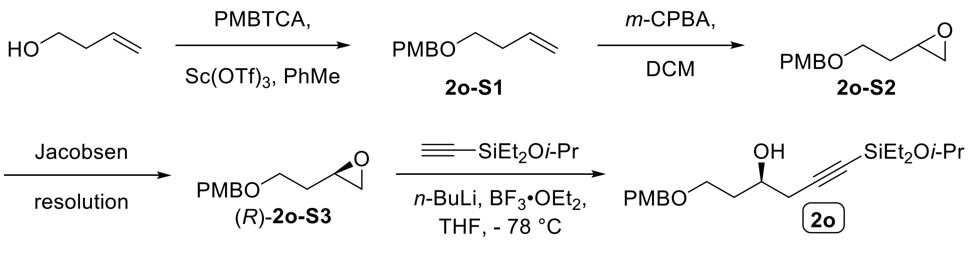
HO
PMBTCA,
Sc(OTf)3, PhMe
PMBO
2o-S1
m-CPBA,
DCM
PMBO
O
2o-S2
Jacobsen
resolution
PMBO
O
(R)-2o-S3
SiEt2Oi-Pr
n-BuLi, BF3•OEt2,
THF, - 78 °C
OH
SiEt2Oi-Pr
PMBO
2o

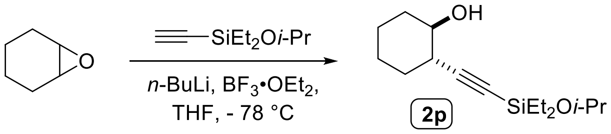
O
SiEt2Oi-Pr
n-BuLi, BF3•OEt2,
THF, - 78 °C
OH
SiEt2Oi-Pr
2p

Preparation of 2a, 2b, 2c and 2z

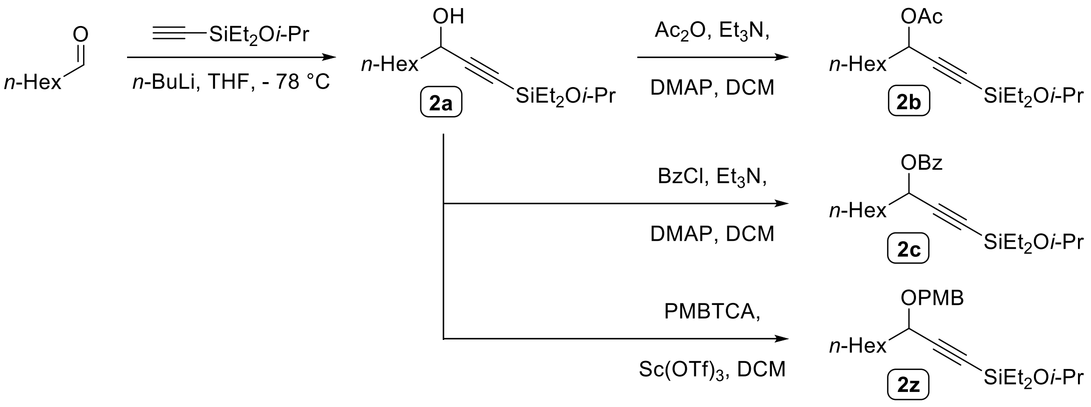

1-(Diethyl(isopropoxy)silyl)non-1-yn-3-ol, 2a

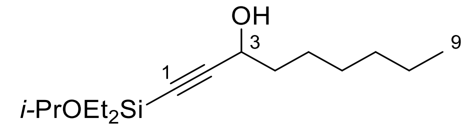

Following general procedure A, *n*-butyllithium (0.96 mL of a 2.5 M solution in hexanes, 2.40 mmol, 1.1 equiv.), silyl alkyne **S2** (450 mg, 83 wt% purity, 2.19 mmol, 1.0 equiv.) in THF (11 mL) and heptanal (0.31 mL, 2.19 mmol, 1.0 equiv.) gave, after purification *via* flash column chromatography (petrol / Et_2O (9:1) + 1% Et_3N), propargylic alcohol **2a** as a colourless oil (515 mg, 1.81 mmol, 83%); $\mathbf{R_f}$ 0.18 (petrol / Et_2O (9:1)); **IR** (thin film, ν_{max} / cm^{-1}) 3353, 2958, 2931, 2876, 2169, 1461, 1380, 1173, 1123, 1032; **^{1}H NMR** (400 MHz, $CDCl_3$) δ_H 4.39 (1H, dt, J = 5.8 and 6.4 Hz, H3), 4.15 (1H, sept, J = 6.1 Hz, OC*H*(CH_3)$_2$), 1.75 (1H, d, J = 5.8 Hz, O*H*), 1.76-1.70 (2H, m H4), 1.51-1.43 (2H, m, H5), 1.36-1.27 (6H, m, H6, H7 and H8), 1.20 (6H, d, J = 6.1 Hz, OCH(C*H*$_3$)$_2$), 1.01 (6H, t, J = 7.8 Hz, Si(CH_2C*H*$_3$)$_2$), 0.89 (3H, t, J = 6.7 Hz, H9), 0.66 (4H, q, J = 7.8 Hz, Si(C*H*$_2$$CH_3$)$_2$); **^{13}C NMR** (125 MHz, $CDCl_3$) δ_C 107.6, 85.8, 66.1, 62.9, 37.9, 31.7, 31.3, 28.8, 25.4, 24.9, 22.5, 14.0, 6.7, 6.6; **HRMS** (ES^+) calc. for $C_{16}H_{32}NaO_2Si$ $[M+Na]^+$ 307.2069, found 307.2064; **EA** calc. for $C_{16}H_{32}O_2Si$: C, 67.54; H, 11.34. Found: C, 67.64; H, 11.24.

1-(Diethyl(isopropoxy)silyl)non-1-yn-3-yl acetate, 2b

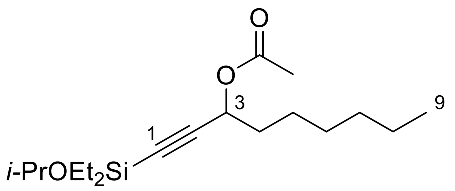

Following general procedure C, acetic anhydride (0.20 mL, 2.11 mmol, 2.0 equiv.), propargyl alcohol **2a** (300 mg, 1.05 mmol, 1.0 equiv.), one crystal of DMAP and Et_3N (0.44 mL, 3.16 mmol, 3.0 equiv.) in CH_2Cl_2 (7 mL) gave acetate **2b** as a colourless oil that was used in the next step without further purification (324 mg, 0.990 mmol, 94%); $\mathbf{R_f}$ 0.34 (petrol / Et_2O (19:1)); **IR** (thin film, ν_{max} / cm^{-1}) 2958, 2930, 2876, 2178, 1747, 1461, 1413, 1369, 1303, 1229, 1122, 1027; ^{1}**H NMR** (500 MHz, $CDCl_3$) δ_H 5.39 (1H, t, J = 6.6 Hz, H3), 4.13 (1H, sept, J = 6.0 Hz, $OCH(CH_3)_2$), 2.08 (3H, s, $COCH_3$), 1.78-1.72 (2H, m, H4), 1.45 (2H, quin, J = 7.4 Hz, H5), 1.35-1.26 (6H, m, H6, H7 and H8), 1.19 (6H, d, J = 6.0 Hz, $OCH(CH_3)_2$), 0.99 (6H, t, J = 7.7 Hz, $Si(CH_2CH_3)_2$), 0.88 (3H, t, J = 6.7 Hz, H9), 0.65 (4H, q, J = 7.7 Hz, $Si(CH_2CH_3)_2$); ^{13}**C NMR** (125 MHz, $CDCl_3$) δ_C 169.8, 103.6, 86.7, 66.1, 64.2, 34.7, 31.6, 28.7, 25.4, 24.9, 22.5, 21.0, 14.0, 6.7, 6.6; **HRMS** (ES^+) calc. for $C_{18}H_{34}NaO_3Si$ $[M+Na]^+$ 349.2175, found 349.2169.

1-(Diethyl(isopropoxy)silyl)non-1-yn-3-yl benzoate, 2c

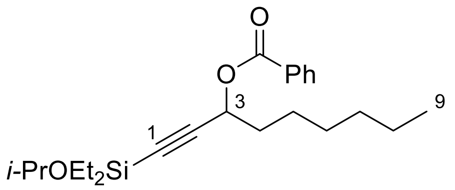

Benzoyl chloride (23 μL, 0.199 mmol, 2.0 equiv.) was added dropwise to a flask charged with propargyl alcohol **2a** (28 mg, 0.099 mmol, 1.0 equiv.), DMAP (2.4 mg, 0.020 mmol, 0.2 equiv.) and Et_3N (41 μL, 0.298 mmol, 3.0 equiv.) in CH_2Cl_2 (1 mL). The mixture was stirred for 16 h, and quenched with $NaHCO_3$ solution. The aqueous

layer was extracted three times with CH_2Cl_2 and the combined organic layers dried ($MgSO_4$) and concentrated to give benzoyl ester **2c** as a colourless oil that was used in the next step without further purification (43 mg of crude, up to 0.099 mmol, ~100%); **R_f** 0.50 (petrol / Et_2O (9:1)); **IR** (thin film, ν_{max} / cm^{-1}) 2958, 2831, 2876, 2179, 1726, 1453, 1381, 1265, 1069, 1028; **^{1}H NMR** (500 MHz, $CDCl_3$) δ_H 8.07 (2H, dd, J = 7.9 and 1.2 Hz, *o*-Ph*H*), 7.58 (1H, tt, J = 7.9 and 1.2 Hz, *p*-Ph*H*), 7.46 (2H, t, J = 7.9 Hz, *m*-Ph*H*), 5.66 (1H, t, J = 6.6 Hz, H3), 4.15 (1H, sept, J = 6.1 Hz, OC*H*(CH_3)$_2$), 1.97-1.86 (2H, m, H4), 1.55 (2H, quin, J = 8.0 Hz, H5), 1.41-1.27 (6H, m, H6, H7 and H8), 1.18 (6H, d, J = 6.1 Hz, OCH(CH_3)$_2$), 1.00 (6H, t, J = 8.0 Hz, Si(CH_2CH_3)$_2$), 0.90 (3H, t, J = 7.0 Hz, H9), 0.67 (4H, q, J = 8.0 Hz, Si(C$H_2$$CH_3$)$_2$); **^{13}C NMR** (125 MHz, $CDCl_3$) δ_C 165.5, 133.1, 130.1, 129.8, 128.7, 103.6, 87.1, 66.2, 64.5, 34.8, 31.7, 28.8, 25.4, 25.0, 22.6, 22.5, 14.0, 6.6, 6.5; **HRMS** (ES$^+$) calc. for $C_{23}H_{36}NaO_3Si$ $[M+Na]^+$ 411.2331, found 411.2326.

Diethyl(isopropoxy)(3-((4-methoxybenzyl)oxy)non-1-yn-1-yl)silane, 2z

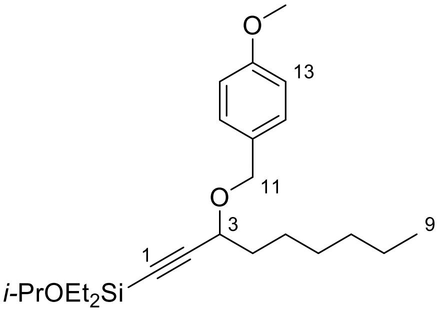

p-(Methoxybenzyl)-trichloroacetimidate (1.38 g, 4.87 mmol, 1.5 equiv.) and scandium (III) triflate (240 mg, 0.487 mmol, 0.15 equiv.) were added to a solution of alcohol **2a** (924 mg, 3.25 mmol, 1.0 equiv.) in toluene (20 mL). The reaction was stirred for 45 minutes, then quenched with $NaHCO_3$ solution, extracted with Et_2O, dried ($MgSO_4$) and concentrated *in vacuo*. The crude was purified by flash column chromatography (petrol + 1% Et_3N) to give **2z** as a colourless oil (1.21 g, 3.00 mmol, 92%); **R_f** 0.29 (petrol / Et_2O (19:1)); **IR** (thin film, ν_{max} / cm^{-1}) 2956, 2932, 2875, 2167, 1613, 1587, 1513, 1463, 1381, 1368, 1332, 1302, 1248, 1173, 1083; **^{1}H NMR** (400 MHz, $CDCl_3$) δ_H 7.29 (2H, d, J = 8.5 Hz, H12), 6.88 (2H, d, J = 8.5 Hz, H13),

4.75 and 4.45 (2 × 1H, d, J = 11.2 Hz, diastereotopic H11), 4.19 (1H, sept, J = 6.0 Hz, OCH(CH$_3$)$_2$), 4.08 (1H, t, J = 6.7 Hz, H3), 3.81 (3H, s, OCH_3), 1.82-1.68 (2H, m, H4), 1.52-1.42 (2H, m, H5), 1.36-1.25 (6H, m, H6, H7 and H8), 1.23 (6H, d, J = 6.0 Hz, OCH(CH_3)$_2$), 1.05 (6H, t, J = 7.7 Hz, Si(CH$_2$CH_3)$_2$), 0.89 (3H, t, J = 6.2 Hz, H9), 0.70 (4H, q, J = 7.7 Hz, Si(CH_2CH$_3$)$_2$); **^{13}C NMR** (101 MHz, CDCl$_3$) δ_C 159.4, 130.1, 129.7, 113.8, 106.0, 86.9, 70.1, 68.7, 66.2, 55.3, 35.7, 31.8, 29.0, 25.5, 25.3, 22.6, 14.1, 6.9, 6.7; **HRMS** (ES$^+$) calc. for $C_{24}H_{40}NaO_3Si$ [M+Na]$^+$ 427.2639, found 427.2645.

Preparation of 2d

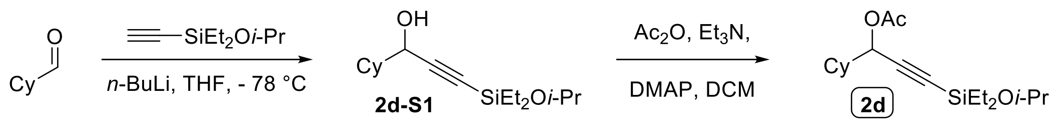

1-Cyclohexyl-3-(diethyl(isopropoxy)silyl)prop-2-yn-1-ol, 2d-S1

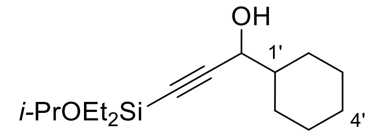

Following general procedure A, n-butyllithium (2.2 mL of a 2.5 M solution in hexanes, 5.50 mmol, 1.1 equiv.), silyl alkyne **S2** (1.00 g, 85 wt% purity, 4.99 mmol, 1.0 equiv.) in THF (40 mL) and cyclohexanecarboxaldehyde (0.60 mL, 4.99 mmol, 1.0 equiv.) gave, after purification *via* flash column chromatography (petrol / Et$_2$O (19:1) + 1% Et$_3$N), propargylic alcohol **2d-S1** as a colourless oil (844 mg, 2.99 mmol, 60%); **R$_f$** 0.26 (petrol / Et$_2$O (9:1)); **IR** (thin film, ν_{max} / cm^{-1}) 3385, 2958, 2927, 2854, 2169, 1451, 1381, 1173, 1123, 1084, 1031; **^{1}H NMR** (500 MHz, CDCl$_3$) δ_H 4.19 (1H, t, J = 5.8 Hz, CHOH), 4.15 (1H, sept, J = 6.1 Hz, OCH(CH$_3$)$_2$), 1.89-1.65 (7H, m, OH, H3' and H4'), 1.62-1.53 (1H, m, H1'), 1.32-1.05 (4H, m, H2'), 1.20 (6H, d, J = 6.1 Hz, OCH(CH_3)$_2$), 1.01 (6H, t, J = 7.9 Hz, Si(CH$_2$CH_3)$_2$), 0.67 (4H, q, J = 7.9 Hz, Si(CH_2CH$_3$)$_2$); **^{13}C NMR** (101 MHz, CDCl$_3$) δ_C 106.5, 86.7, 67.5, 66.1, 44.0, 28.6, 27.9, 26.4, 25.8, 25.8, 25.4, 6.7, 6.6; **HRMS** (ES$^+$) calc. for $C_{16}H_{30}NaO_2Si$

$[M+Na]^+$ 305.1913, found 305.1902; **EA** calc. for $C_{16}H_{30}O_2Si$: C, 68.03; H, 10.70. Found: C, 67.94; H, 10.72.

1-Cyclohexyl-3-(diethyl(isopropoxy)silyl)prop-2-yn-1-yl acetate, 2d

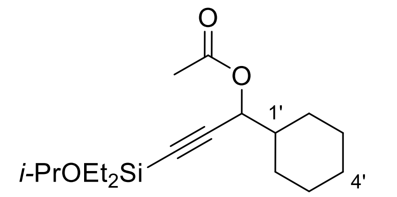

Following general procedure C, acetic anhydride (0.56 mL, 5.95 mmol, 2.0 equiv.), propargyl alcohol **2d-S1** (840 mg, 2.97 mmol, 1.0 equiv.), one crystal of DMAP and Et_3N (1.2 mL, 8.92 mmol, 3.0 equiv.) in CH_2Cl_2 gave acetate **2d** as a colourless oil that was used in the next step without further purification (968 mg, ~2.97 mmol, ~100%); $\mathbf{R_f}$ 0.42 (petrol / Et_2O (9:1)); **IR** (thin film, ν_{max} / cm^{-1}) 2960, 2931, 2878, 2856, 2176, 1746, 1452, 1370, 1228, 1173, 1122, 1082, 1032; **^{1}H NMR** (400 MHz, $CDCl_3$) δ_H 5.26 (1H, d, J = 6.0 Hz, CHOAc), 4.15 (1H, sept, J = 6.1 Hz, OCH(CH_3)$_2$), 2.09 (3H, s, COCH_3), 1.88-1.61 (7H, m, H1’, H3’ and H4’), 1.30-1.10 (4H, m, H2’), 1.20 (6H, d, J = 6.1 Hz, OCH(CH_3)$_2$), 1.00 (6H, t, J = 8.0 Hz, Si(CH_2CH_3)$_2$), 0.66 (4H, q, J = 8.0 Hz, Si(C$H_2$$CH_3$)$_2$); **^{13}C NMR** (101 MHz, $CDCl_3$) δ_C 169.9, 102.5, 87.4, 68.5, 66.1, 41.7, 28.5, 27.9, 26.4, 25.7, 25.6, 25.4, 20.9, 6.7, 6.5; **HRMS** (ES^+) calc. for $C_{18}H_{32}NaO_3Si$ $[M+Na]^+$ 347.2018, found 347.2002.

Preparation of 2e

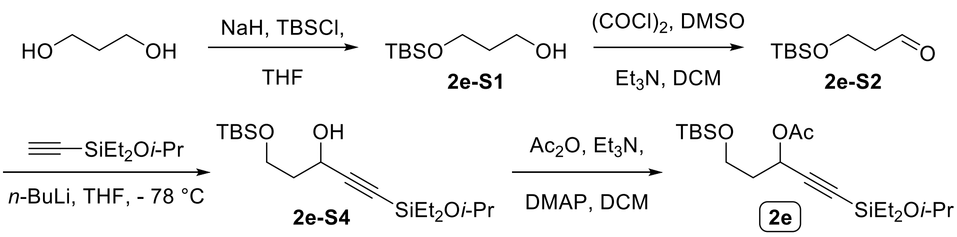

3-((*Tert*-butyldimethylsilyl)oxy)propan-1-ol, 2e-S1

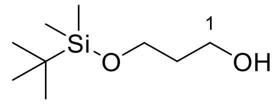

Prepared according to a literature procedure.[4] Sodium hydride (1.58 g of a 60 wt% dispersion in mineral oil, 39.4 mmol, 1.0 equiv.) was taken up in THF (80 mL) and propane-1,3-diol (3.0 g, 39.4 mmol, 1.0 equiv.) was added dropwise at room temperature. The mixture was stirred for 45 minutes, *tert*-butyldimethylsilyl chloride (5.94 g, 39.4 mmol, 1.0 equiv.) added, and stirred for a further 45 minutes. The reaction was then diluted with 500 mL of Et_2O, washed with K_2CO_3 solution and brine, and dried ($MgSO_4$). The concentrated crude was purified *via* flash column chromatography (petrol / EtOAc (9:1→1:1)) to give alcohol **2e-S1** as a colourless oil (5.37 g, 28.22 mmol, 72%); **R_f** 0.19 (petrol / Et_2O (2:1)); **^{1}H NMR** ($CDCl_3$, 200 MHz) δ_H 3.88-3.78 (4H, m, H1 and H3), 2.61 (1H, t, J = 5.4 Hz, O*H*), 1.79 (2H, quin, J = 5.6 Hz, H2), 0.91 (9H, s, (C*H*$_3$)$_3$CSi), 0.09 (6H, s, 2 × SiC*H*$_3$); **^{13}C NMR** (101 MHz, $CDCl_3$) δ_C 62.4, 61.8, 34.3, 25.8, 18.1, -5.6; **LRMS** (ES$^+$) calc. for $C_9H_{22}NaO_2Si$ $[M]^+$ 213.1, found 213.1.

The spectroscopic data were found to be in agreement with that reported by Chavan and co-workers.[5]

3-((*Tert*-butyldimethylsilyl)oxy)propanal, 2e-S2

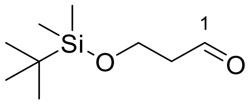

Prepared according to a literature procedure.[4] Dimethyl sulfoxide (0.41 mL, 5.78 mmol, 2.2 equiv.) was added dropwise to oxalyl chloride (0.25 mL, 2.89 mmol, 1.1 equiv.) in CH_2Cl_2 (20 mL) at –78 ºC and stirred for 15 minutes before alcohol **2e-S1** (500 mg, 2.63 mmol, 1.0 equiv.) was cannulated in as a solution in CH_2Cl_2 (5 mL). The mixture was stirred for a further 30 minutes at –78 ºC, Et_3N (2.2 mL, 15.8 mmol, 6.0 equiv.) added, and after 10 minutes at –78 ºC was warmed to room temperature and stirred for one hour. The mixture was washed with $NaHCO_3$ solution, and the aqueous layer extracted three times with CH_2Cl_2. The organic layers were combined, dried ($MgSO_4$), concentrated and purified *via* flash column chromatography (petrol / EtOAc (19:1)) to give aldehyde **2e-S2** as a colourless oil (289 mg, 1.83 mmol, 58%); $\mathbf{R_f}$ 0.50 (petrol / Et_2O (2:1)); **^{1}H NMR** ($CDCl_3$, 200 MHz) δ_H 9.81 (1H, t, J = 2.1 Hz, H1), 4.00 (2H, t, J = 6.0 Hz, H3), 2.61 (2H, dt, J = 6.0 and 2.1 Hz, H2), 0.89 (9H, s, $(CH_3)_3CSi$), 0.07 (6H, s, 2 × $SiCH_3$); **^{13}C NMR** (101 MHz, $CDCl_3$) δ_C 201.9, 57.3, 46.5, 25.8, 18.2, -5.5; **HRMS** (ES^+) calc. for $C_9H_{20}NaO_2Si$ $[M]^+$ 211.11, found 211.11.

The spectroscopic data were found to be in agreement with that reported by Gieseler and co-workers.[6]

10,10-Diethyl-2,2,3,3,12-pentamethyl-4,11-dioxa-3,10-disilatridec-8-yn–7-ol, 2e-S3

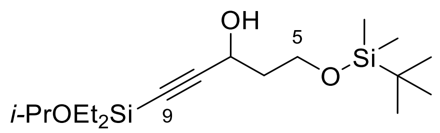

Following general procedure A, *n*-butyllithium (0.67 mL of a 2.5 M solution in hexanes, 1.67 mmol, 1.1 equiv.), silyl alkyne **S2** (306 mg, 85 wt% purity, 1.52 mmol, 1.0 equiv.) in THF (8 mL) and 3-((*tert*-butyldimethylsilyl)oxy)propanal **2e-S2** (285 mg, 1.52 mmol, 1.0 equiv.) gave, after purification *via* flash column chromatography (petrol / Et_2O (9:1) + 1% Et_3N), propargylic alcohol **2e-S3** as a colourless oil (495 mg, 1.38 mmol, 91%); $\mathbf{R_f}$ 0.30 (petrol / Et_2O (4:1)); **IR** (thin film, ν_{max} / cm^{-1}) 2957, 2930, 2879, 2172, 1463, 1383, 1255, 1173, 1093, 1032; **^{1}H NMR** (400 MHz, $CDCl_3$) δ_H 4.65 (1H, dt, $J = 6.2$ and 4.4 Hz, H7), 4.16 (1H, sept, $J = 6.1$ Hz, OC*H*(CH_3)$_2$), 4.12-4.06 and 3.87-3.82 (2 × 1H, m, diastereotopic H5), 3.48 (1H, d, $J = 4.4$ Hz, O*H*), 2.08-1.99 and 1.91-1.83 (2 × 1H, m, diastereotopic H6), 1.20 (6H, d, $J = 6.1$ Hz, OCH(C*H*$_3$)$_2$), 1.01 (6H, t, $J = 7.9$ Hz, Si(CH_2C*H*$_3$)$_2$), 0.91 (9H, s, Si(CH_3)$_2$C(C*H*$_3$)$_3$), 0.67 (4H, q, $J = 7.9$ Hz, Si(C*H*$_2$$CH_3$)$_2$), 0.10 and 0.09 (2 × 3H, s, diastereotopic Si(C*H*$_3$)$_2$C(CH_3)$_3$); **^{13}C NMR** (101 MHz, $CDCl_3$) 106.9, 85.7, 66.1, 62.3, 61.0, 38.4, 25.8, 25.5, 18.1, 6.7, 6.6, -5.6; **HRMS** (ES^+) calc. for $C_{18}H_{38}NaO_3Si_2$ $[M+Na]^+$ 381.2257, found 381.2242.

10,10-Diethyl-2,2,3,3,12-pentamethyl-4,11-dioxa-3,10-disilatridec-8-yn–7-yl acetate, 2e

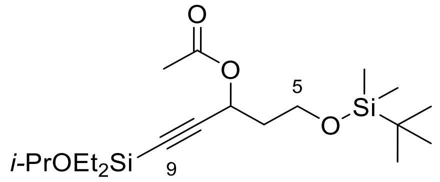

Following general procedure C, acetic anhydride (0.22 mL, 2.30 mmol, 2.0 equiv.), propargyl alcohol **2e-S3** (412 mg, 1.15 mmol, 1.0 equiv.), one crystal of DMAP and

Et_3N (0.48 mL, 3.45 mmol, 3.0 equiv.) in CH_2Cl_2 gave acetate **2e** as a colourless oil that was used in the next step without further purification (462 mg, ~1.15 mmol, ~100%); **R$_f$** 0.28 (petrol / Et_2O (19:1)); **IR** (thin film, ν_{max} / cm^{-1}) 2958, 2878, 2178, 1750, 1463, 1370, 1229, 1173, 1120, 1032; **^{1}H NMR** (500 MHz, $CDCl_3$) δ_H 5.52 (1H, t, J = 6.9 Hz, H7), 4.13 (1H, sept, J = 6.0 Hz, OCH(CH_3)$_2$), 3.79-3.71 (2H, m, H5), 2.07 (3H, s, COCH_3), 2.04-1.94 (2H, m, H6), 1.18 (6H, d, J = 6.0 Hz, OCH(CH_3)$_2$), 0.99 (6H, t, J = 7.9 Hz, Si(CH_2CH_3)$_2$), 0.89 (9H, s, Si(CH_3)$_2$C(CH_3)$_3$), 0.65 (4H, q, J = 7.9 Hz, Si(C$H_2$$CH_3$)$_2$), 0.05 and 0.05 (2 × 3H, s, diastereotopic Si(CH_3)$_2$C(CH_3)$_3$); **^{13}C NMR** (125 MHz, $CDCl_3$) 169.5, 103.3, 86.9, 66.1, 61.5, 58.7, 37.7, 25.8, 25.4, 20.9, 18.2, 7.8, 6.6, 6.5, -5.5, -5.5; **HRMS** (ES^+) calc. for $C_{20}H_{40}NaO_4Si_2$ $[M+Na]^+$ 423.2363, found 423.2365.

Preparation of 2f

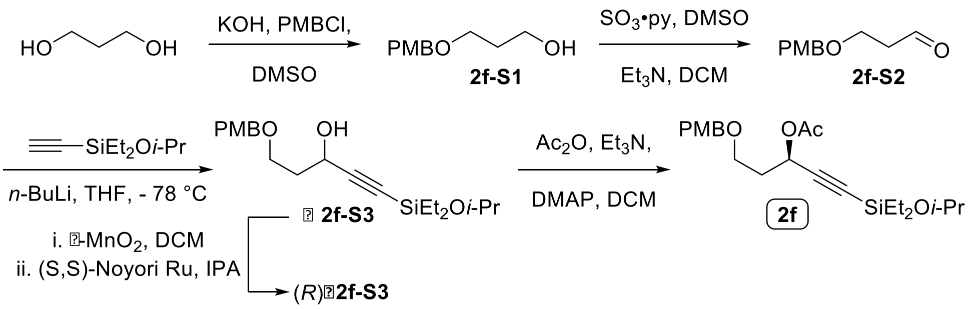

3-((4-Methoxybenzyl)oxy)propan-1-ol, 2f-S1

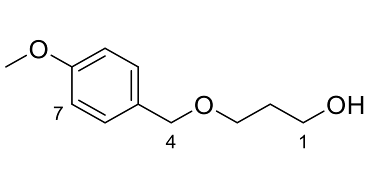

Prepared according to a literature procedure.[7] Potassium hydroxide (3.40 g, 60.7 mmol, 2.0 equiv.) was added portionwise to a solution of propane-1,3-diol (4.4 mL, 60.7 mmol, 2.0 equiv.) in anhydrous dimethyl sulfoxide (20 mL) at 0 ºC. The mixture was warmed to room temperature and stirred for one hour until it became clear, then cooled to 0 ºC again and *p*-methoxybenzyl chloride (4.1 mL, 30.4 mmol, 1.0 equiv.)

added. The reaction was warmed to room temperature and stirred for three hours, when TLC revealed the starting material had been consumed. It was then diluted with 30 mL of Et_2O at 0 ºC and 15 mL of 4 M HCl was slowly added. The mixture was extracted three times with Et_2O, dried (Na_2SO_4) and concentrated *in vacuo*. The concentrated crude was purified *via* flash column chromatography (petrol / EtOAc (5:1→1:1)) to give alcohol **2f-S1** as a colourless oil (4.43 g, 22.6 mmol, 74%); **R_f** 0.17 (petrol / EtOAc (3:2)); **^{1}H NMR** ($CDCl_3$, 400 MHz) δ_H 7.26 (2H, d, J = 8.8 Hz, H6), 6.89 (2H, d, J = 8.8 Hz, H7), 4.46 (2H, s, H4), 3.81 (3H, s, ArOCH_3), 3.81-3.76 (2H, m, H1), 3.64 (2H, t, J = 5.9 Hz, H3), 2.35 (1H, br, OH), 1.86 (2H, quin, J = 5.9 Hz, H2); **^{13}C NMR** (101 MHz, $CDCl_3$) δ_C 159.2, 130.2, 129.3, 113.8, 72.8, 68.8, 61.5, 55.2, 32.1; **LRMS** (ES^+) calc. for $C_{11}H_{16}NaO_3$ $[M+Na]^+$ 219.1, found 219.1.

The spectroscopic data were found to be in agreement with that reported by Kretschmer and co-workers.[77]

3-((4-Methoxybenzyl)oxy)propanal, 2f-S2

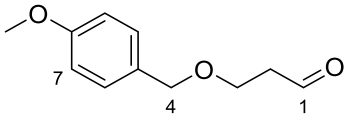

Prepared according to a literature procedure.[8] Et_3N (13.5 mL, 97.4 mmol, 5.0 equiv.), dimethyl sulfoxide (9.7 mL, 137 mmol, 7.0 equiv.) and SO_3·py (9.23 g, 58.0 mmol, 3.0 equiv.) were added to a solution of alcohol **2f-S1** (3.82 g, 19.5 mmol, 1.0 equiv.) in CH_2Cl_2 (10 mL) at 0 ºC and stirred for 30 minutes. The reaction was then quenched with aqueous pH 7 phosphate buffer, extracted with EtOAc, washed with brine and dried (Na_2SO_4). The concentrated crude was purified *via* flash column chromatography (petrol / EtOAc (9:1→4:1)) to give aldehyde **2f-S2** as a colourless oil (2.85 g, 14.7 mmol, 75%); **R_f** 0.37 (petrol / EtOAc (4:1)); **^{1}H NMR** ($CDCl_3$, 400 MHz) δ_H 9.80 (1H, t, J = 1.9 Hz, H1), 7.26 (2H, d, J = 8.7 Hz, H6), 6.89 (2H, d, J = 8.7 Hz, H7), 4.47 (2H, s, H4), 3.81 (3H, s, ArOCH_3), 3.64 (2H, t, J = 5.9 Hz, H3), 2.69 (2H, dt, J = 5.9 and 1.9 Hz, H2); **^{13}C NMR** (101 MHz, $CDCl_3$) δ_C 201.3, 159.3,

129.9, 129.3, 113.8, 72.9, 63.5, 55.2, 43.9; **LRMS** (ES^+) calc. for $C_{11}H_{14}NaO_3$ $[M+Na]^+$ 217.1, found 217.1.

The spectroscopic data were found to be in agreement with that reported by Hayashi and co-workers.[8]

1-(Diethyl(isopropoxy)silyl)-5-((4-methoxybenzyl)oxy)pent-1-yn-3-ol, 2f-S3

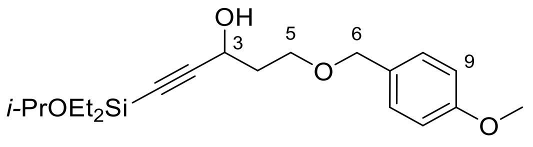

Following general procedure A, *n*-butyllithium (4.5 mL of a 2.5 M solution in hexanes, 11.3 mmol, 1.1 equiv.), silyl alkyne **S2** (2.06 g, 85 wt% purity, 10.3 mmol, 1.0 equiv.) in THF (50 mL) and 3-((4-methoxybenzyl)oxy)propanal, **2f-S2** (2.00 g, 10.3 mmol, 1.0 equiv.) gave, after purification *via* flash column chromatography (petrol / EtOAc (9:1) + 1% Et_3N), propargylic alcohol **2f-S3** as a colourless oil (3.54 g, 9.71 mmol, 94%); **R_f** 0.23 (petrol / EtOAc (4:1)); **IR** (thin film, ν_{max} / cm^{-1}) 3426, 2959, 2876, 2170, 1613, 1513, 1412, 1367, 1247, 1173, 1096, 1028; **^{1}H NMR** (400 MHz, $CDCl_3$) δ_H 7.26 (2H, d, J = 8.7 Hz, H8), 6.89 (2H, d, J = 8.7 Hz, H9), 4.63 (1H, dt, J = 6.5 and 4.5 Hz, H3), 4.48 and 4.45 (2 × 1H, d, J = 11.4 Hz, diastereotopic H6), 4.14 (1H, sept, J = 6.1 Hz, OC*H*(CH_3)$_2$), 3.90-3.84 (1H, m, diastereotopic H5), 3.81 (3H, s, OC*H*$_3$), 3.70-3.65 (1H, m, diastereotopic H5), 3.08 (1H, d, J = 6.5 Hz, O*H*), 2.15-2.07 and 1.99-1.91 (2 × 1H, m, diastereotopic H4), 1.19 (6H, d, J = 6.1 Hz, OCH(C*H*$_3$)$_2$), 1.00 (6H, t, J = 7.9 Hz, Si(CH_2C*H*$_3$)$_2$), 0.66 (4H, q, J = 7.9 Hz, Si(C*H*$_2$$CH_3$)$_2$); **^{13}C NMR** (101 MHz, $CDCl_3$) 159.3, 129.9, 129.3, 113.8, 107.0, 85.8, 73.1, 67.3, 66.1, 61.6, 55.2, 36.7, 25.5, 6.7, 6.6; **HRMS** (ES^+) calc. for $C_{20}H_{32}NaO_4Si$ $[M+Na]^+$ 387.1968, found 387.1960.

1-(Diethyl(isopropoxy)silyl)-5-((4-methoxybenzyl)oxy)pent-1-yn-3-one, 2f-S4

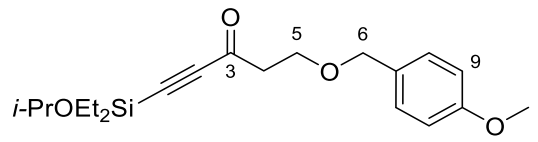

Alcohol **2f-S3** (1.17 g, 3.20 mmol, 1.0 equiv.) was stirred with activated manganese dioxide (5.56 g, 64.0 mmol, 20 equiv.) and 4 Å molecular sieves in CH_2Cl_2 (30 mL) for 4 hours, then filtered through Celite® and concentrated to give ketone **2f-S4** as a colourless oil (865 mg, 2.39 mmol, 75%); $\mathbf{R_f}$ 0.46 (petrol / Et_2O (3:2)); **IR** (thin film, ν_{max} / cm^{-1}) 2964, 2936, 2878, 2149, 1681, 1613, 1514, 1248, 1119, 1034; **^{1}H NMR** (400 MHz, $CDCl_3$) δ_H 7.24 (2H, d, J = 8.6 Hz, H8), 6.86 (2H, d, J = 8.6 Hz, H9), 4.45 (2H, s, H6), 4.13 (1H, sept, J = 6.1 Hz, OCH(CH_3)$_2$), 3.81-3.77 (5H, m, H5 and OCH_3), 2.85 (2H, t, J = 6.1 Hz, H4), 1.19 (6H, d, J = 6.1 Hz, OCH(CH_3)$_2$), 1.01 (6H, t, J = 8.0 Hz, Si(CH_2CH_3)$_2$), 0.71 (4H, q, J = 8.0 Hz, Si(C$H_2$$CH_3$)$_2$); **^{13}C NMR** (101 MHz, $CDCl_3$) 185.2, 159.3, 130.0, 129.3, 113.8, 101.8, 94.6, 72.9, 66.7, 64.5, 55.2, 45.7, 25.4, 6.4, 6.4; **HRMS** (FI^+) calc. for $C_{20}H_{30}O_4Si$ $[M+]^+$ 362.1913, found 362.1917.

(*R*)-1-(Diethyl(isopropoxy)silyl)-5-((4-methoxybenzyl)oxy)pent-1-yn-3-ol, (*R*)-2f-S3

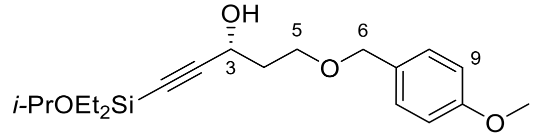

A solution of ketone **2f-S4** (1.88 g, 5.20 mmol, 1.0 equiv.) in isopropyl alcohol (10 mL) was degassed with argon for one hour, before the addition of (1*R*,2*R*)-(+)-*N*-tosyl-1,2-diphenylethane-1,2-diamine[η^6-1-isopropyl-4-methylbenzene)-ruthenium(II) (Noyori catalyst[9]) (74 mg, 0.104 mmol, 0.02 equiv.) as a solution in CH_2Cl_2 (3 mL). The mixture was stirred for 45 minutes before being concentrated under reduced pressure. The crude residue was purified *via* flash column

chromatography (petrol / EtOAc (9:1) + 1% Et_3N) to give propargylic alcohol (*R*)-**2f-S3** as a colourless oil (1.75 g, 4.79 mmol, 92%); **ee** 97% (CHIRALPAK-IC, 2% IPA/*n*-Hex, 1.3 mL/min, *R* - 7.85 min, *S* - 8.72 min); $[\alpha]_D^{25}$ +24.4° (*c* 1.0, $CHCl_3$). Other data were identical to that reported for (±)-**2f-S3**.

(*R*)-1-(Diethyl(isopropoxy)silyl)-5-((4-methoxybenzyl)oxy)pent-1-yn-3-yl, (R)-2f

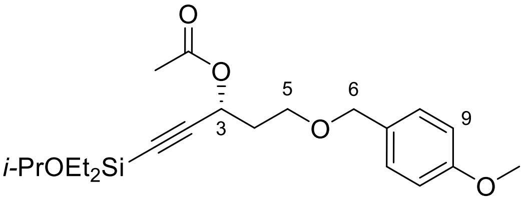

Following general procedure C, acetic anhydride (0.20 mL, 2.11 mmol, 2.0 equiv.), propargyl alcohol (*R*)-**2f-S3** (300 mg, 1.05 mmol, 1.0 equiv.), one crystal of DMAP and Et_3N (0.44 mL, 3.16 mmol, 3.0 equiv.) in CH_2Cl_2 (7 mL) gave acetate (*R*)-**2f** as a colourless oil that was used in the next step without further purification (324 mg, 0.99 mmol, 94%); $\mathbf{R_f}$ 0.34 (petrol / Et_2O (19:1)); $[\alpha]_D^{25}$ +56.6° (*c* 1.0, $CHCl_3$) for 97% ee; **IR** (thin film, ν_{max} / cm^{-1}) 2963, 2936, 2877, 2179, 1747, 1613, 1587, 1514, 1463, 1369, 1302, 1229, 1173, 1120, 1099, 1030; ^{1}**H NMR** (400 MHz, $CDCl_3$) δ_H 7.25 (2H, d, *J* = 8.6 Hz, H8), 6.88 (2H, d, *J* = 8.7 Hz, H9), 5.57 (1H, t, *J* = 6.7 Hz, H3), 4.43 and 4.42 (2 × 1H, d, *J* = 11.5 Hz, diastereotopic H6), 4.11 (1H, sept, *J* = 6.0 Hz, $OCH(CH_3)_2$), 3.81 (3H, s, OCH_3), 3.63-3.52 (2H, m, diastereotopic H5), 2.14-2.01 (2H, m, diastereotopic H4), 2.05 (3H, s, $COCH_3$), 1.18 (6H, d, *J* = 6.0 Hz, $OCH(CH_3)_2$), 0.98 (6H, t, *J* = 7.8 Hz, $Si(CH_2CH_3)_2$), 0.64 (4H, q, *J* = 7.8 Hz, $Si(CH_2CH_3)_2$); ^{13}**C NMR** (400 MHz, $CDCl_3$) 169.6, 159.2, 130.2, 129.3, 113.7, 103.2, 86.9, 72.7, 66.1, 65.4, 61.7, 55.2, 35.1, 25.4, 20.9, 6.6, 6.5; **HRMS** (FI^+) calc. for $C_{22}H_{34}O_5Si$ $[M]^+$ 406.2176, found 406.2187.

Preparation of 2g

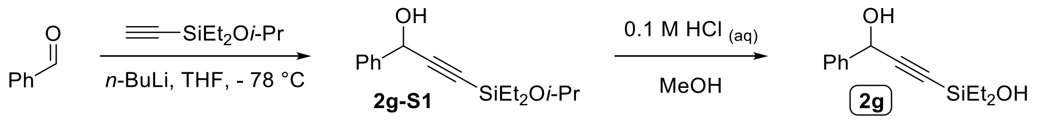

3-(Diethyl(isopropoxy)silyl)-1-phenylprop-2-yn-1-ol, 18, 2g-S1

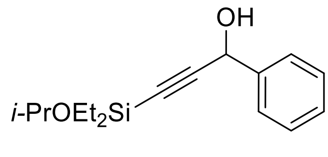

Following general procedure A, *n*-butyllithium (4.4 mL of a 2.5 M solution in hexanes, 11.0 mmol, 1.1 equiv.), silyl alkyne **S2** (2.00g, 85 wt% purity, 10.0 mmol, 1.0 equiv.) in THF (40 mL) and benzaldehyde (1.02 mL, 10.0 mmol, 1.0 equiv.) gave, after purification *via* flash column chromatography (petrol / Et_2O (9:1→4:1) + 1% Et_3N), propargylic alcohol **2g-S1** as a colourless oil (2.73 g, 9.86 mmol, 99%); $\mathbf{R_f}$ 0.13 (petrol / Et_2O (9:1)); **IR** (thin film, ν_{max} / cm^{-1}) 3340, 2967, 2878, 2173, 1455, 1381, 1369, 1237, 1173, 1121, 1031; **^{1}H NMR** (500 MHz, $CDCl_3$) δ_H 7.57 (2H, d, *J* = 7.4 Hz, *o*-Ar*H*), 7.40 (2H, app t, *J* = 7.4 Hz, *m*-Ar*H*), 7.35 (1H, t, *J* = 7.4 Hz, *p*-Ar*H*), 5.50 (1H, s, C*H*OH), 4.17 (1H, sept, *J* = 6.1 Hz, OC*H*$(CH_3)_2$), 2.17 (1H, br s, O*H*), 1.20 (6H, d, *J* = 6.1 Hz, $OCH(CH_3)_2$), 1.03 (6H, t, *J* = 8.0 Hz, $Si(CH_2CH_3)_2$), 0.70 (4H, q, *J* = 8.0 Hz, $Si(CH_2CH_3)_2$); **^{13}C NMR** (125 MHz, $CDCl_3$) δ_C 140.2, 128.6, 128.4, 126.7, 105.5, 88.3, 66.2, 65.0, 25.4, 6.7, 6.6; **HRMS** (ES^+) calc. for $C_{16}H_{24}NaO_2Si$ $[M+Na]^+$ 299.1443, found 299.1430.

Diethyl(3-hydroxy-3-phenylprop-1-yn-1-yl)silanol, 2g

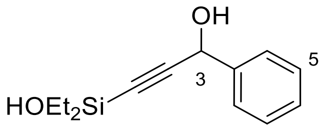

A mixture of alcohol **2g-S1** (96 g, 0.357 mmol, 1.0 equiv.), HCl (2 mL of a 0.1 M aqueous solution) and MeOH (2 mL) was stirred for 10 minutes. The mixture was aqueous neutralised with $NaHCO_3$, saturated with NaCl(s), and extracted four times with EtOAc. The combined organic layer was dried ($MgSO_4$) and concentrated to give silanol **2g** as a colourless, viscous oil, which was immediately diluted with toluene and used in the next step with no further purification (77 mg, 0.329 mmol, 92%); $\mathbf{R_f}$ 0.27 (petrol / Et_2O (1:1)); **IR** (thin film, ν_{max} / cm^{-1}) 3302, 2958, 2914, 2876, 2175, 1494, 1454, 1412, 1238, 1192, 1082, 1039, 1004; ^{1}**H NMR** (400 MHz, $CDCl_3$) δ_H 7.53 (2H, d, J = 7.0 Hz, H4), 7.39–7.29 (3H, m, H5 and H6), 5.44 (1H, s, H3), 3.03 (2H, br s, O*H* and SiO*H*), 1.02 (6H, t, J = 7.9 Hz, $Si(CH_2CH_3)_2$), 0.70 (4H, q, J = 7.9 Hz, $Si(CH_2CH_3)_2$); ^{13}**C NMR** (101 MHz, $CDCl_3$) δ_C 140.0, 128.6, 128.5, 126.8, 105.6, 88.7, 64.8, 7.4, 6.5; **HRMS** (ES^+) calc. for $C_{13}H_{18}NaO_2Si$ $[M+Na]^+$ 257.0968, found 257.0970.

Preparation of 2h

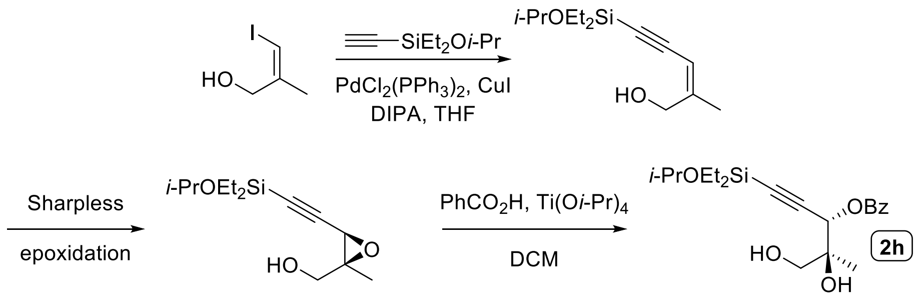

(3*S*,4*R*)-1-(Diethyl(hydroxy)silyl)-4,5-dihydroxy-4-methylpent-1-yn-3-yl benzoate, 2h

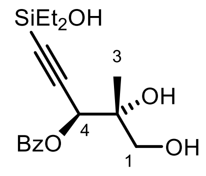

R$_f$ 0.16 (petrol / EtOAc (1:1)); **IR** (thin film, v_{max} / cm^{-1}) 3438, 2179, 1729, 1602, 1269, 1118, 1028; **^{1}H NMR** (500 MHz, $CDCl_3$) δ_H 8.04 (2H, d, *J* = 8.0 Hz, *o*-ArH), 7.60 (1H, t, *J* = 8.0 Hz, *p*-Ar*H*), 7.46 (2H, t, *J* = 8.0 Hz, *m*-Ar*H*), 5.74 (1H, s, H4), 3.68 (1H, d, *J* = 12.0 Hz, 1 × H1), 3.56 (1H, d, *J* = 12.0 Hz, 1 × H1), 2.94 (3H, br s, SiO*H*, CO*H* and CH_2O*H*), 1.34 (3H, s, H3), 1.03 and 1.02 (2 × 3H, t, *J* = 8.0 Hz, diastereotopic $Si(CH_2CH_3)_2$), 0.74−0.67 (4H, m, $Si(CH_2CH_3)_2$); **^{13}C NMR** (125 MHz, $CDCl_3$) δ_C 165.6, 133.6, 129.9, 129.1, 128.6, 100.3, 90.1, 74.0, 67.7, 66.2, 19.6, 7.3, 7.2, 6.4, 6.3; **HRMS** (ESI+) calc. for $C_{17}H_{24}O_5SiNa$ [M+Na]$^+$ 359.1285, found 359.1281.

Preparation of 2j

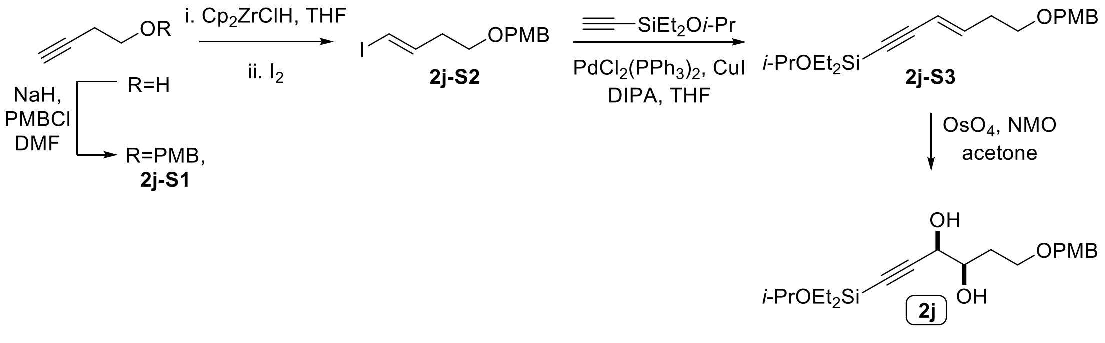

1-((But-3-yn-1-yloxy)methyl)-4-methoxybenzene, 2j-S1

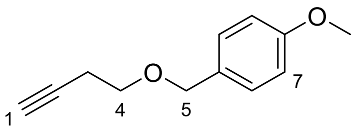

A solution of 4-methoxybenzyl chloride (10.6 mL, 78.4 mmol, 1.1 equiv.) in anhydrous DMF (40 mL) was cooled to 0 °C and 3-butyn-1-ol (5.4 mL, 71.3 mmol, 1.0 equiv.) was added in one portion. Sodium hydride (3.14 g, 60 wt% in mineral oil, 78.4 mmol, 1.1 equiv.) was added portionwise over 15 minutes and the solution stirred at 0 °C for 45 minutes. The reaction was quenched with NH_4Cl solution, and extracted three times with Et_2O. The combined organic layers were washed 5 times with water, dried ($MgSO_4$) and concentrated. The crude was purified by flash column chromatography (petrol / Et_2O (7:3)) to afford alkyne **2j-S1** as a colourless oil (9.00 g, 47.3 mmol, 66%); $\mathbf{R_f}$ 0.69 (petrol / Et_2O (1:1)); ^{1}**H NMR** (400 MHz, $CDCl_3$) δ_H 7.28 (2H, d, J = 8.7 Hz, H6), 6.89 (2H, d, J = 8.7 Hz, H7), 4.49 (2H, s, H5), 3.80 (3H, s, OCH_3), 3.58 (2H, t, J = 7.0 Hz, H4), 2.49 (2H, td, J = 7.0 and 2.5 Hz, H3), 2.01 (1H, t, J = 2.5 Hz, H1); ^{13}**C NMR** (101 MHz, $CDCl_3$) δ_C 159.3, 130.1, 129.4, 113.8, 81.5, 72.6, 69.4, 67.9, 55.2, 19.9; **HRMS** (ES+) calc. for $C_{12}H_{14}NaO_2$ $[M+Na]^+$ 213.0886, found 213.0886.

The spectroscopic data were found to be in agreement with that reported by Crimmins and co-workers.[10]

(*E*)-1-(((4-Iodobut-3-en-1-yl)oxy)methyl)-4-methoxybenzene, 2j-S2

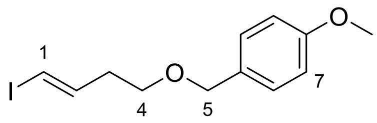

Based on a modified literature procedure.[11] Alkyne **2j-S1** (1.64 g, 8.60 mmol, 1.0 equiv.) was added *via* cannula to a solution of $Cp_2Zr(H)Cl$ (2.44 g, 10.3 mmol, 1.2 equiv.) in THF (20 mL) to form a cloudy solution. The reaction was stirred until the

mixture cleared and was then cooled to 0 °C. A solution of iodine (2.73 g, 10.8 mmol, 1.25 equiv.) in anhydrous THF (10 mL) was added *via* cannula and the reaction stirred at 0 °C for one hour, then diluted with wet Et_2O, quenched with $Na_2S_2O_3$ solution at 0 °C, and stirred at room temperature for one hour. The mixture was separated, the aqueous layer extracted with Et_2O and the combined organic layers washed with brine, dried ($MgSO_4$), and concentrated. The crude was purified by flash chromatography (petrol / EtOAc (19:1)) to give iodide **2j-S2** as a yellow oil (2.10 g, 6.60 mmol, 77%); **R_f** 0.52 (petrol / EtOAc (17:3); **IR** (thin film, ν_{max} / cm^{-1}) 3001, 2856, 1612, 1513, 1247, 1098; **^{1}H NMR** (400 MHz, $CDCl_3$) δ_H 7.26 (2H, d, *J* = 8.6 Hz, H6), 6.90 (2H, d, *J* = 8.6 Hz, H7), 6.56 (1H, dt, *J* = 14.5 and 7.1 Hz, H2), 6.11 (1H, dt, *J* = 14.5 and 1.5 Hz, H1), 4.45 (2H, s, H5), 3.82 (3H, s, OCH_3), 3.49 (2H, t, *J* = 6.6 Hz, H4), 2.35 (2H, dtd, *J* = 7.1, 6.6 and 1.5 Hz, H3); **^{13}C NMR** (101 MHz, $CDCl_3$) δ_C 159.2, 143.1, 130.3, 129.4, 113.9, 76.7, 72.7, 68.2, 55.4, 36.4; **HRMS** (ES+) calc. for $C_{12}H_{15}INaO_2$ $[M+Na]^+$ 341.0009, found 341.0003.

(*E*)-Diethyl(isopropoxy)(6-((4-methoxybenzyl)oxy)hex-3-en-1-yn-1-yl)silane, 2j-S3

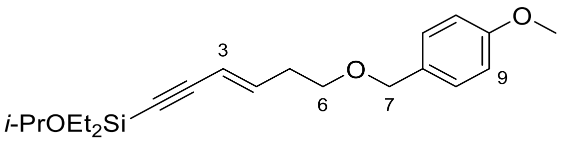

A solution of vinyl iodide **2j-S2** (1.04 g, 3.27 mmol, 1.0 equiv.) and alkynyl silane **S2** (612 mg, 3.60 mmol, 1.1 equiv.) in anhydrous THF (36 mL) was degassed with nitrogen for 5 minutes before addition of $PdCl_2(PPh_3)_2$ (138 mg, 0.20 mmol, 0.06 equiv.) and CuI (69 mg, 0.36 mmol, 0.1 equiv.). The solution was degassed for 5 minutes, diisopropylamine (3.8 mL, 28.5 mmol, 8.0 equiv.) added and the mixture degassed for a further 5 minutes before being left to stir at room temperature for 5 hours. The reaction was quenched with $NaHCO_3$ solution, and stirred at room temperature for one hour before separation and extraction of the aqueous layer with CH_2Cl_2. The combined organic layers were dried ($MgSO_4$) and concentrated. The crude was purified by flash column chromatography (petrol / Et_2O (9:1) + 1% Et_3N) to give enyne **2j-S3** as a colourless oil (993 mg, 2.75 mmol, 84%); **R_f** 0.44 (petrol /

Et_2O (7:3)); **IR** (thin film, ν_{max} / cm^{-1}) 2959, 2136, 1613, 1513, 1247, 1173, 1098, 1033; **^{1}H NMR** (400 MHz, $CDCl_3$) δ_H 7.27 (2H, d, 8.6 Hz, H8), 6.89 (2H, d, *J* = 8.6 Hz, H9), 6.26 (1H, dt, *J* = 16.0 and 7.1 Hz, H4), 5.61 (1H, dt, *J* = 16.0 and 1.5 Hz, H3), 4.45 (2H, s, H7), 4.16 (1H, sept, *J* = 6.1 Hz, OC*H*(CH_3)$_2$), 3.82 (3H, s, OC*H*$_3$), 3.50 (2H, t, *J* = 6.7 Hz, H6), 2.43 (2H, dtd, *J* = 7.1, 6.7 and 1.5 Hz, H5), 1.20 (6H, d, *J* = 6.1 Hz, OCH(C*H*$_3$)$_2$), 1.01 (6H, t, *J* = 8.0 Hz, Si(CH_2C*H*$_3$)$_2$), 0.67 (4H, q, *J* = 8.0 Hz, Si(C*H*$_2$$CH_3$)$_2$); **^{13}C NMR** (101 MHz, $CDCl_3$) δ_C 159.1, 142.9, 130.2, 129.4, 113.9, 111.3, 104.5, 89.7, 72.6, 68.7, 66.0, 55.3, 33.7, 25.6, 6.8, 6.6; **HRMS** (ES+) calc. for $C_{21}H_{32}NaO_3Si$ $[M+Na]^+$ 383.2013, found 383.2005.

1-(Diethyl(isopropoxy)silyl)-6-((4-methoxybenzyl)oxy)hex-1-yne-3,4-diol, 2j

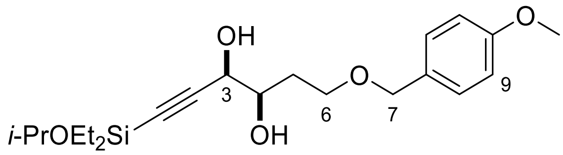

Based on a modified literature procedure.[12] To a solution of enyne **2j-S3** (180 mg, 0.500 mmol, 1.0 equiv.) in acetone (0.5 mL) and water (0.24 mL) was added OsO_4 (130 µL of a 2.5 wt% solution in *t*-BuOH, 0.011 mmol, 2.0 mol%) and N-methylmorpholine-N-oxide (0.26 mL of a 50 wt% solution in water, 1.25 mmol, 2.5 equiv.) and the mixture stirred for one hour. The reaction was diluted with EtOAc, washed sequentially with $Na_2S_2O_3$ solution, water and brine, and dried (Na_2SO_4). The concentrated crude was purified by flash chromatography (petrol / Et_2O (3:2) + 1 % Et_3N) to alkyne **2j** as a colourless oil (115 mg, 0.291 mmol, 58 %); **R$_f$** 0.56 (petrol / EtOAc (1:1)); **IR** (thin film, ν_{max} / cm^{-1}) 3406, 2960, 2877, 2174, 1613, 1514, 1248, 1033; **^{1}H NMR** (400 MHz, $CDCl_3$) δ_H 7.25 (2H, d, *J* = 8.6 Hz, H8), 6.89 (2H, d, *J* = 8.6 Hz, H9), 4.47 (2H, s, H7), 4.26 (1H, dd, *J* = 6.3 and 5.3 Hz, H3), 4.13 (1H, sept, *J* = 6.2 Hz, OC*H*(CH_3)$_2$), 3.87-3.82 (1H, m, H4), 3.81 (3H, s, OC*H*$_3$), 3.74-3.65 (2H, m, H6), 3.24 (1H, d, *J* = 3.6 Hz, C^3-O*H*), 2.74 (1H, d, *J* = 5.3 Hz, C^4-O*H*), 2.08-1.98 and 1.91-1.82 (2 × 1H, m, diastereotopic H5), 1.19 (6H, d, *J* = 6.2 Hz, OCH(C*H*$_3$)$_2$), 1.00 (6H, t, *J* = 7.9 Hz, Si(CH_2C*H*$_3$)$_2$), 0.66 (4H, q, *J* = 7.9 Hz, Si(C*H*$_2$$CH_3$)$_2$); **^{13}C NMR** (101 MHz, $CDCl_3$) δ_C 159.4, 129.9, 129.4, 113.9, 104.6, 87.6, 73.8, 72.9, 67.7,

66.6, 66.2, 55.3, 32.3, 25.5, 6.7, 6.6; **HRMS** (ES+) calc. for $C_{21}H_{34}NaO_5Si$ $[M+Na]^+$ 417.2068, found 417.2057.

Preparation of 2k

1-((Diethyl(isopropoxy)silyl)ethynyl)cyclohexan-1-ol, 2k

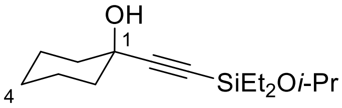

n-Butyllithium (0.92 mL of a 2.5 M solution in hexanes, 2.31 mmol, 1.2 equiv.) was added dropwise to a solution of alkyne **S2** (500 mg, 85 wt% purity, 2.50 mmol, 1.3 equiv.) in THF (10 mL) at –78 °C, and stirred for one hour, before dropwise addition of cyclohexanone (0.20 mL, 1.92 mmol, 1.0 equiv.). The mixture was stirred at –78 °C for two hours, before it was quenched with NH_4Cl, extracted with Et_2O, dried ($MgSO_4$) and concentrated. The crude was purified *via* flash column chromatography (petrol / Et_2O (9:1) + 1% Et_3N) to give tertiary alcohol **2k** as a colourless oil (515 mg, 1.92 mmol, quant.); $\mathbf{R_f}$ 0.18 (petrol / Et_2O (4:1)); **IR** (thin film, ν_{max} / cm^{-1}) 3345, 2935, 2877, 2165, 1448, 1340, 1122, 1070, 1008; **^{1}H NMR** (400 MHz, $CDCl_3$) δ_H 4.16 (1H, sept, J = 6.1 Hz, $OC\mathit{H}(CH_3)_2$), 2.27-2.20 (1H, m, O*H*), 1.97-1.89 (2H, m, 2 × H3), 1.75-1.67 (2H, m, 2 × H2), 1.63-1.51 (5H, m, 2 × H2, 2 × H3 and 1 × H4), 1.30-1.18 (1H,m, 1 × H4), 1.20 (6H, d, J = 6.1 Hz, $OCH(C\mathit{H}_3)_2$), 1.01 (6H, t, J = 7.8 Hz, $Si(CH_2C\mathit{H}_3)_2$), 0.66 (4H, q, J = 7.8 Hz, $Si(C\mathit{H}_2CH_3)_2$); **^{13}C NMR** (101 MHz, $CDCl_3$) 110.4, 85.0, 69.0, 66.1, 39.9, 24.5, 25.2, 23.4, 6.8, 6.7; **HRMS** (ES^+) calc. for $C_{15}H_{28}NaO_2Si$ $[M+Na]^+$ 291.1751, found 291.1742.

Preparation of 2l

1-(Diethyl(isopropoxy)silyl)dec-1-yn-4-ol, 2l

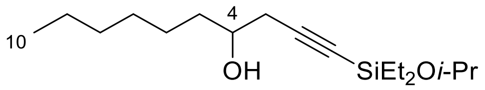

According to general procedure B, *n*-butyllithium (2.1 mL of a 2.5 M solution in hexanes, 5.28 mmol, 1.5 equiv.) and silyl alkyne **S2** (1.00 g, 90 wt% purity, 5.28 mmol, 1.5 equiv.) in THF (20 mL) were stirred at –78 ºC for one hour. 1,2-Epoxyoctane (0.54 mL, 3.52 mmol, 1.0 equiv.) was added, and the mixture stirred for a further 10 minutes before $BF_3{\cdot}OEt_2$ (0.65 mL, 5.28 mmol, 1.5 equiv.) was added. The mixture was stirred for 4.5 hours. The crude was purified *via* flash column chromatography (petrol / Et_2O (9:1 → 4:1) + 1% Et_3N) to give homopropargylic alcohol **2l** as a colourless oil (542 mg, 1.82 mmol, 52%); $\mathbf{R_f}$ 0.24 (petrol / Et_2O (9:1)); **IR** (thin film, ν_{max} / cm^{-1}) 3374, 2958, 2931, 2876, 2174, 1461, 1380,1236, 1124, 1031; ^{1}**H NMR** (400 MHz, $CDCl_3$) δ_H 4.07 (1H, sept, *J* = 6.0 Hz, OC*H*(CH_3)$_2$), 3.72-3.65 (1H, m, H4), 2.42 (1H, dd, *J* = 16.7 and 5.1 Hz, 1 × H3), 2.33 (1H, dd, *J* = 16.7 and 6.5 Hz, 1 × H3), 1.93 (1H, br s, O*H*), 1.53-1.19 (10H, m, H5, H6, H7, H8 and H9), 1.12 (6H, d, *J* = 6.0 Hz, OCH(C*H*$_3$)$_2$), 0.93 (6H, t, *J* = 7.8 Hz, Si(CH_2C*H*$_3$)$_2$), 0.82 (3H, t, *J* = 6.6 Hz, H10), 0.58 (4H, q, *J* = 7.8 Hz, Si(C*H*$_2$$CH_3$)$_2$); ^{13}**C NMR** (101 MHz, $CDCl_3$) 104.1, 84.1, 69.9, 66.0, 36.3, 31.8, 29.3, 28.8, 25.5, 25.5, 22.6, 14.1, 6.9, 6.7; **HRMS** (ES^+) calc. for $C_{17}H_{34}NaO_2Si$ $[M+Na]^+$ 321.2220, found 321.2221.

Preparation of 2m

2-Cyclohexyloxirane, 2m-S1

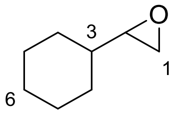

Prepared according to a modified literature procedure.[13] 3-Chloroperoxybenzoic acid (2.75 g of 77 wt% solid, 8.77 mmol, 1.2 equiv.) was added to vinylcyclohexane (1.00 mL, 7.30 mmol, 1.0 equiv.) in CH_2Cl_2 (40 mL) at 0 ºC, and stirred at room temperature for 16 hours. The mixture was diluted with petrol, washed sequentially with $NaHCO_3$, $Na_2S_2O_3$, $NaHCO_3$, and brine solutions, and dried ($MgSO_4$). The residue was concentrated *in vacuo* to give epoxide **2m-S1** as a colourless oil (795 mg, 6.30 mmol, 86%); $\mathbf{R_f}$ 0.32 (CH_2Cl_2 / petrol (2:3)); **^{1}H NMR** ($CDCl_3$, 400 MHz) δ_H 2.65-2.62 (2H, m, H1), 2.46-2.44 (1H, m, H2), 1.83-1.78 (1H, m, H3), 1.70-1.57 (4H, m, H5), 1.22-0.98 (6H, m, H4 and H6); **^{13}C NMR** (101 MHz, $CDCl_3$) δ_C 56.7, 46.0, 40.4, 29.7, 28.8, 26.3, 25.7, 25.5.

The spectroscopic data were found to be in agreement with that reported by Piccinini and co-workers.[14]

1-Cyclohexyl-4-(diethyl(isopropoxy)silyl)but-3-yn-1-ol, 2m

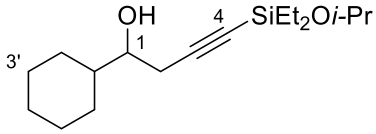

According to general procedure B, *n*-butyllithium (6.6 mL of a 2.0 M solution in hexanes, 13.2 mmol, 2.1 equiv.) and silyl alkyne **S2** (2.52 g, 85 wt% purity, 12.6 mmol, 2.0 equiv.) in THF (30 mL) were stirred at –78 ºC for one hour. Epoxide **2m-S1** (795 mg, 6.30 mmol, 1.0 equiv.) was added, and the mixture stirred for a further

10 minutes before $BF_3 \cdot OEt_2$ (1.55 mL, 12.6 mmol, 2.0 equiv.) was added. The mixture was stirred for three hours. The crude was purified *via* flash column chromatography (petrol / Et_2O (19:1) + 1% Et_3N) to give homopropargylic alcohol **2m** as a colourless oil (1.24 g, 4.18 mmol, 66%); **R$_f$** 0.34 (petrol / Et_2O (19:1)); **IR** (thin film, ν_{max} / cm^{-1}) 3439, 2927, 2877, 2174, 1451, 1380, 1124, 1031; **^{1}H NMR** (400 MHz, $CDCl_3$) δ_H 4.07 (1H, sept, *J* = 6.1 Hz, OC*H*(CH_3)$_2$), 3.45-3.40 (1H, m, H1), 2.44 (1H, dd, *J* = 16.9 and 4.6 Hz, 1 × H2), 2.35 (1H, dd, *J* = 16.9 and 7.2 Hz, 1 × H2), 1.98 (1H, s, O*H*), 1.88-1.81 (1H, m, 1 × diastereotopic H2'), 1.74-1.67 and 1.63-1.56 (2 × 2H, m, diastereotopic H3'), 1.46-1.37 (1H, m, H1'), 1.21-1.02 (5H, m, 3 × diastereotopic H2' and H4'), 1.12 (6H, d, *J* = 6.1 Hz, OCH(C*H*$_3$)$_2$), 0.93 (6H, t, *J* = 7.9 Hz, Si(CH_2C*H*$_3$)$_2$), 0.57 (4H, q, *J* = 7.9 Hz, Si(C*H*$_2$$CH_3$)$_2$); **^{13}C NMR** (101 MHz, $CDCl_3$) 104.5, 84.0, 73.9, 66.0, 42.7, 29.1, 28.1, 26.4, 26.2, 26.1, 26.0, 25.5, 6.8, 6.7; **HRMS** (ES$^+$) calc. for $C_{17}H_{32}NaO_2Si$ [M+Na]$^+$ 319.2064, found 319.2053.

Preparation of 2n

(But-3-en-1-yloxy)(*tert*-butyl)dimethylsilane, 2n-S1

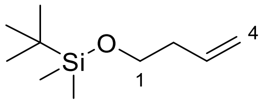

Prepared according to a literature procedure.[15] Butenol (0.50 mL, 5.80 mmol, 1.0 equiv.) was added dropwise to a solution of imidazole (435 mg, 6.39 mmol, 1.1 equiv.) and *tert*-butyldimethylsilyl chloride (963 mg, 6.39 mmol, 1.1 equiv.) in CH_2Cl_2 (15 mL). The reaction was stirred for 2.5 hours, diluted with Et_2O, washed three times with water, once with brine, and dried ($MgSO_4$). The crude was concentrated to give alkene **2n-S1** as a colourless oil used without further purification (940 mg, 5.04 mmol, 87%); **R$_f$** 0.70 (petrol / Et_2O (2:1)); **^{1}H NMR** ($CDCl_3$, 400 MHz) δ_H 5.88-5.77 (1H, m, H3), 5.10-5.01 (2H, m, H4), 3.66 (2H, t, *J* = 6.8 Hz, H1), 2.31-2.26 (2H, m, H2), 0.90 (9H, s, (C*H*$_3$)$_3$CSi), 0.06 (6H, s, 2 × SiC*H*$_3$); **^{13}C NMR**

(400 MHz, $CDCl_3$) δ_C 135.4, 116.3, 62.8, 37.5, 25.9, 18.3, -5.3; **HRMS** (ES^+) calc. for $C_{10}H_{22}NaOSi$ $[M]^+$ 209.13, found 209.13.

The physical and spectroscopic data was found to be in agreement with that reported by Gieseler and co-workers.[6]

Tert-butyldimethyl(2-oxiran-2-yl)ethoxy)silane, 2n-S2

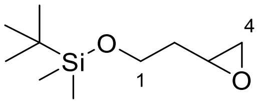

Prepared according to a modified literature procedure.[13] 3-chloroperoxybenzoic acid (1.23 g, 5.49 mmol, 1.2 equiv.) was added to alkene **2n-S1** (853 mg, 4.58 mmol, 1.0 equiv.) in CH_2Cl_2 (25 mL) at 0 ºC under argon, and stirred at room temperature for 16 hours. The mixture was diluted with petrol, washed sequentially with $NaHCO_3$, $Na_2S_2O_3$, $NaHCO_3$, and brine solutions, and dried ($MgSO_4$). The residue was concentrated and purified by column chromatography (petrol / Et_2O (9:1)) to give epoxide **2n-S2** as a colourless oil (814 mg, 4.02 mmol, 88%); $\mathbf{R_f}$ 0.27 (petrol / Et_2O (2:1)); **^{1}H NMR** ($CDCl_3$, 400 MHz) δ_H 3.78 (2H, t, J = 5.9 Hz, H1), 3.08-3.03 (1H, m, H3), 2.79 (1H, t, J = 4.7 Hz, 1 × H4), 2.53 (1H, dd, J = 4.7 and 2.8 Hz, 1 × H4), 1.83-1.66 (2H, m, H2), 0.90 (9H, s, $(CH_3)_3CSi$), 0.07 (6H, s, 2 × $SiCH_3$); **^{13}C NMR** (400 MHz, $CDCl_3$) δ_C 59.9, 50.0, 47.1, 35.9, 25.8, 18.2, -5.4; **HRMS** (ES^+) calc. for $C_{10}H_{22}NaO_2Si$ $[M]^+$ 225.1287, found 225.11.

The physical and spectroscopic data was found to be in agreement with that reported by Tan and co-workers.[16]

11,11-Diethyl-2,2,3,3,13-pentamethyl-4,12-dioxa-3,11-disilatetradec-9-yn–7-ol, 2n

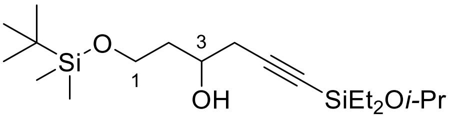

According to general procedure B, *n*-butyllithium (0.74 mL of a 2.5 M solution in hexanes, 1.86 mmol, 1.6 equiv.) and silyl alkyne **S2** (344 mg, 85 wt% purity, 1.72 mmol, 1.4 equiv.) in THF (8 mL) were stirred at –78 ºC for one hour. Epoxide **2n-S2** (243 mg, 1.20 mmol, 1.0 equiv.) was added, and the mixture stirred for a further 10 minutes before $BF_3{\cdot}OEt_2$ (0.21 mL, 1.72 mmol, 1.4 equiv.) was added. The mixture was stirred for two hours. The crude was purified *via* flash column chromatography (petrol / Et_2O (9:1) + 1% Et_3N) to give homopropargylic alcohol **2n** as a colourless oil (377 mg, 1.19 mmol, 99%); $\mathbf{R_f}$ 0.23 (petrol / Et_2O (4:1)); **IR** (thin film, ν_{max} / cm^{-1}) 3449, 2957, 2931, 2878, 2175, 1471, 1382, 1367, 1120, 1093; **^{1}H NMR** (400 MHz, $CDCl_3$) δ_H 4.14 (1H, sept, J = 6.1 Hz, OC*H*(CH_3)$_2$), 4.03-3.97 (1H, m, H3), 3.97-3.91 and 3.86-3.80 (2 × 1H, m, diastereotopic H1), 3.48 (1H, d, J = 3.1 Hz, O*H*), 2.53 (1H, dd, J = 16.9 and 5.6 Hz, diastereotopic H4), 2.44 (1H, dd, J = 16.9 and 6.9 Hz, diastereotopic H4), 1.92-1.85 and 1.81-1.70 (2 × 1H, m, diastereotopic H2), 1.19 (6H, d, J = 6.1 Hz, OCH(C*H*$_3$)$_2$), 1.00 (6H, t, J = 7.8 Hz, Si(CH_2C*H*$_3$)$_2$), 0.90 (9H, s, SiC(C*H*$_3$)$_3$), 0.64 (4H, q, J = 7.8 Hz, Si(C*H*$_2$$CH_3$)$_2$), 0.09 (6H, s, Si(C*H*$_3$)$_2$); **^{13}C NMR** (101 MHz, $CDCl_3$) 104.4, 83.5, 70.4, 66.1, 62.1, 37.3, 28.4, 25.8, 25.4, 18.1, 6.8, 6.6, -5.6; **HRMS** (ES^+) calc. for $C_{19}H_{40}NaO_3Si_2$ $[M+Na]^+$ 395.2408, found 395.2408.

Preparation of 2o

1-((But-3-en-1-yloxy)methyl)-4-methoxybenzene, 2o-S1

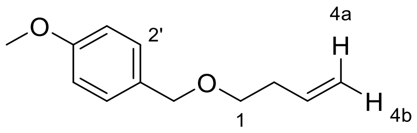

p-(Methoxybenzyl)-trichloroacetimidate (1.23 g, 4.36 mmol, 1.5 equiv.) and scandium (III) triflate (71 mg, 0.145 mmol, 0.05 equiv.) were added to a solution of 3-buten-1-ol (0.25 mL, 2.91 mmol, 1.0 equiv.) in toluene (50 mL). The reaction was stirred for 15 minutes, then quenched with $NaHCO_3$ solution, extracted with Et_2O, dried ($MgSO_4$) and concentrated *in vacuo*. The crude was purified by flash column chromatography (petrol / Et_2O (19:1)) to give protected alkene **2o-S1** as a colourless oil (560 mg, ~2.91 mmol, ~100%); $\mathbf{R_f}$ 0.39 (petrol / Et_2O (2:1)); **^{1}H NMR** ($CDCl_3$, 400 MHz) δ_H 7.27 (2H, d, *J* = 8.9 Hz, H2’), 6.89 (2H, d, *J* = 8.9 Hz, H3’), 5.89-5.79 (1H, m, H3), 5.10 (1H, d, *J* = 17.4 Hz, H4a), 5.05 (1H, d, *J* = 10.3 Hz, H4b), 4.46 (2H, s, ArCH_2O), 3.81 (3H , s, OCH_3), 3.50 (2H, t, *J* = 6.8 Hz, H1), 2.37 (2H, qt, *J* = 6.8 and 1.7 Hz, H2); **^{13}C NMR** (101 MHz, $CDCl_3$) δ_C 159.1, 135.3, 130.6, 129.3, 116.3, 113.8, 72.6, 69.3, 55.3, 34.3; **LRMS** (ES^+) calc. for $C_{12}H_{16}NaO_2$ $[M+Na]^+$ 215.1, found 215.1.

The spectroscopic data were found to be in agreement with that reported by Barbazanges and co-workers.[17]

2-(2-((4-Methoxybenzyl)oxy)ethyl)oxirane, 2o-S2

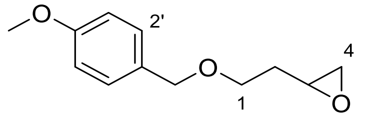

Prepared according to a modified literature procedure.[13] 3-Chloroperoxybenzoic acid, 77% (700 mg, 3.12 mmol, 1.2 equiv.) was added to alkene **2o-S1** (500 mg, 2.60 mmol, 1.0 equiv.) in CH_2Cl_2 (12 mL) at 0 ºC, and stirred at room temperature for 16 hours. The mixture was diluted with petrol, washed sequentially with $NaHCO_3$, $Na_2S_2O_3$, $NaHCO_3$, and brine solutions, and dried ($MgSO_4$). The residue was concentrated *in vacuo* and purified by column chromatography (petrol / Et_2O (6:1→3:1)) to give epoxide **2o-S2** as a colourless oil (455 mg, 2.19 mmol, 84%); $\mathbf{R_f}$ 0.15 (petrol / Et_2O (4:1)); **^{1}H NMR** ($CDCl_3$, 400 MHz) δ_H 7.28 (2H, d, *J* = 8.7 Hz, H2'), 6.89 (2H, d, *J* = 8.7 Hz, H3'), 4.47 and 4.47 (2 × 1H, d, *J* = 11.7 Hz, diastereotopic ArCH_2O), 3.82 (3H , s, OCH_3), 3.62-3.58 (2H, m, H1), 3.09-3.05 (1H, m, H3), 2.79 (1H, t, *J* = 4.3 Hz, diastereotopic H4), 2.53 (1H, dd, *J* = 4.3 and 2.7 Hz, diastereotopic H4), 1.95-1.87 and 1.81-1.73 (2 × 1H, m, diastereotopic H2); **^{13}C NMR** (101 MHz, $CDCl_3$) δ_C 159.2, 130.4, 129.2, 113.8, 72.7, 66.7, 55.2, 50.1, 47.1, 33.0; **LRMS** (ES$^+$) calc. for $C_{12}H_{16}NaO_3$ $[M+Na]^+$ 231.1, found 231.1.

The spectroscopic data were found to be in agreement with that reported by Dubey and co-workers.[18]

(*R*)-2-(2-((4-Methoxybenzyl)oxy)ethyl)oxirane, (*R*)-2o-S2

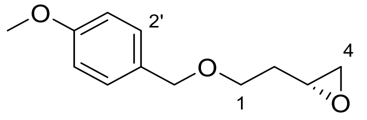

Prepared according to a modified literature procedure.[19] (*R,R*)-(-)-*N,N'*-Bis(3,5-di-*tert*-butylsalicyclidene)-1,2-cyclohexanediaminocobalt (II) (61 mg, 0.100 mmol, 0.02 equiv.) was added to neat epoxide (±)-**2o-S2** (1.04 g, 5.01 mmol, 1.0 equiv.),

followed by acetic acid (23 μL, 0.401 mmol, 0.08 equiv.). The mixture was cooled to 0 °C, water (50 μL, 2.76 mmol, 0.55 equiv.) added and left to stir at room temperature for 96 hours. The crude was purified directly *via* flash column chromatography (petrol / Et_2O (100:0→99:1) + 1% Et_3N) to give (*R*)-**2o-S2** as a pale yellow oil (379 mg, 1.82 mmol, 36%); **ee** >99% (CHIRALPAK-IC, 1% IPA/*n*-Hex, 1.3 mL/min, *R* - 22.10 min, *S* - 23.79 min); $[\alpha]_D^{25}$ +14.7° (*c* 1.0, $CHCl_3$) [lit.[20] +13.6° (*c* 3.44, $CHCl_3$)]. Other data were identical to that reported for **(±)-2o-S2**.

(*R*)-6-(Diethyl(isopropoxy)silyl)-1-((4-methoxybenzyl)oxy)hex-5-yn-3-ol, 2o

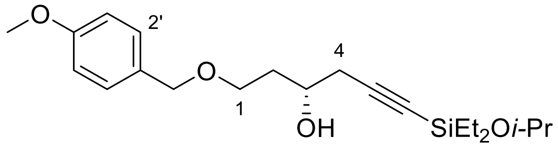

According to general procedure B, *n*-butyllithium (0.81 mL of a 2.5 M solution in hexanes, 2.02 mmol, 2.1 equiv.) and silyl alkyne **S2** (385 mg, 85 wt% purity, 1.92 mmol, 2.0 equiv.) in THF (8 mL) were stirred at –78 ºC for one hour. Epoxide (*R*)-**2o-S2** (200 mg, 0.961 mmol, 1.0 equiv.) was added, and the mixture stirred for a further 10 minutes before $BF_3{\cdot}OEt_2$ (0.24 mL, 1.92 mmol, 2.0 equiv.) was added. The mixture was stirred for 4 hours. The crude was purified *via* flash column chromatography (petrol / EtOAc (9:1→4:1) + 1% Et_3N) to give homopropargylic alcohol (*R*)-**2o** as a colourless oil (287 mg, 0.758 mmol, 79%); $[\alpha]_D^{20}$ +0.42° (*c* 0.5, $CHCl_3$); **R_f** 0.20 (petrol / Et_2O (2:1)); **IR** (thin film, ν_{max} / cm^{-1}) 3452, 2959, 2876, 2173, 1613, 1514, 1248, 1173, 1120, 1096, 1031; **^{1}H NMR** (400 MHz, $CDCl_3$) δ_H 7.26 (2H, d, *J* = 8.6 Hz, H2'), 6.88 (2H, d, *J* = 8.6 Hz, H3'), 4.46 (2H, s, H1'), 4.13 (1H, sept, *J* = 6.1 Hz, OC*H*(CH_3)$_2$), 4.00-3.95 (1H, m, H3), 3.81 (3H, s, OC*H*$_3$), 3.75-3.70 and 3.66-3.60 (2 × 1H, m, diastereotopic H1), 3.08 (1H, br s, O*H*), 2.50 (1H, dd, *J* = 16.8 and 5.6 Hz, diastereotopic H4), 2.44 (1H, dd, *J* = 16.8 and 6.9 Hz, diastereotopic H4), 1.98-1.91 and 1.88-1.80 (2 × 1H, m, diastereotopic H2), 1.18 (6H, d, *J* = 6.1 Hz, OCH(C*H*$_3$)$_2$), 0.99 (6H, t, *J* = 7.8 Hz, Si(CH_2C*H*$_3$)$_2$), 0.64 (4H, q, *J* = 7.8 Hz, Si(C*H*$_2$$CH_3$)$_2$); **^{13}C NMR** (101 MHz, $CDCl_3$) 159.3, 130.0, 129.3, 113.8,

104.2, 83.7, 72.9, 69.6, 68.2, 65.9, 55.2, 35.3, 28.4, 25.6, 6.8, 6.7; **HRMS** (ES^+) calc. for $C_{21}H_{34}NaO_4Si$ $[M+Na]^+$ 401.2124, found 401.2114.

Preparation of 2p

2-((Diethyl(isopropoxy)silyl)ethynyl)cyclohexan-1-ol, 2p

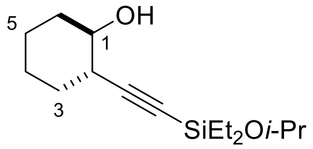

According to general procedure B, *n*-butyllithium (2.7 mL of a 2.5 M solution in hexanes, 6.87 mmol, 1.8 equiv.) and silyl alkyne **S2** (1.30 g, 85 wt% purity, 6.49 mmol, 1.7 equiv.) in THF (15 mL) were stirred at –78 ºC for one hour. Cyclohexene oxide (0.38 mL, 3.74 mmol, 1.0 equiv.) was added, and the mixture stirred for a further 10 minutes before $BF_3{\cdot}OEt_2$ (0.80 mL, 6.49 mmol, 1.7 equiv.) was added. The mixture was stirred for 5 hours. The crude was purified *via* flash column chromatography (petrol / Et_2O (9:1) + 1% Et_3N) to give homopropargylic alcohol **2p** as a colourless oil (574 mg, 2.14 mmol, 57%); $\mathbf{R_f}$ 0.17 (petrol / Et_2O (4:1)); **IR** (thin film, ν_{max} / cm^{-1}); 3412, 2936, 2877, 2170, 1450, 1366, 1235, 1173, 1124, 1032; **^{1}H NMR** (400 MHz, $CDCl_3$) δ_H 4.14 (1H, sept, J = 6.1 Hz, $OCH(CH_3)_2$), 3.52-3.45 (1H, m, H1), 2.38 (1H, br s, O*H*), 2.32-2.26 (1H, m, H2), 2.05-1.98 (2H, m, 1 × H3 and 1 × H6), 1.79-1.74 (1H, m, 1 × H4 or 1 × H5), 1.70-1.64 (1H, m, 1 × H4 or 1 × H5), 1.47-1.14 (4H, m, 1 × H3, 1 × H4, 1 × H5 and 1 × H6), 1.20 (6H, d, J = 6.1 Hz, $OCH(CH_3)_2$), 1.00 (6H, t, J = 7.8 Hz, $Si(CH_2CH_3)_2$), 0.65 (4H, q, J = 7.8 Hz, $Si(CH_2CH_3)_2$); **^{13}C NMR** (101 MHz, $CDCl_3$) 109.0, 83.3, 73.3, 66.0, 39.8, 32.8, 30.7, 25.5, 24.7, 24.0, 6.9, 6.7; **HRMS** (ES^+) calc. for $C_{15}H_{28}NaO_2Si$ $[M+Na]^+$ 291.1751, found 291.1743.

1.5 Hydrogenation of Alkynylsilanes

2,2-Diethyl-5-hexyl-2,5-dihydro-1,2-oxasilole, 1a

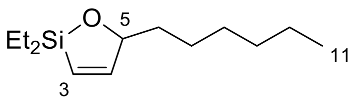

According to general procedure D, palladium on $CaCO_3$ (722 mg, 5 wt % Pd, 0.339 mmol, 0.05 equiv.), acetate **2a** (2.21 g, 6.78 mmol, 1.0 equiv.) and quinoline (0.16 mL, 1.36 mmol, 0.2 equiv.) in toluene (20 mL) were stirred under a hydrogen atmosphere for 50 minutes and gave, after purification by **rapid** flash column chromatography (petrol / Et_2O (19:1)), oxasilole **1a** as a colourless oil which was unstable to silica gel (1.23 g, 5.43 mmol, 80% isolated); $\mathbf{R_f}$ 0.57 (petrol / Et_2O (19:1)); **IR** (thin film, ν_{max} / cm^{-1}) 2957, 2930, 2875, 2858, 1740, 1558, 1460, 1236, 1133, 1021; **^{1}H NMR** (500 MHz, $CDCl_3$) δ_H 6.91 (1H, dd, J = 10.5 and 1.5 Hz, H3), 5.96 (1H, dd, J = 10.5 and 2.0 Hz, H4), 4.67 (1H, ddt, J = 5.2, 2.0 and 1.5 Hz, H5), 1.51-1.24 (10H, m, H6-H10), 0.95 and 0.92 (2 × 3H, t, J = 7.8 Hz, 2 × diastereotopic $Si(CH_2CH_3)$), 0.89 (3H, t, J = 7.1 Hz, H11), 0.76-0.59 (4H, m, $Si(CH_2CH_3)_2$); **^{13}C NMR** (125 MHz, $CDCl_3$) δ_C 154.6, 124.1, 83.3, 37.7, 31.8, 29.4, 25.4, 22.6, 14.0, 7.2, 7.1, 6.8, 6.5; **HRMS** (ES^+) calc. for $C_{13}H_{26}OSi$ $[M]^+$ 226.1753, found 226.1760.

(*E*)-1-(diethyl(methoxy)silyl)non-1-en-3-ol, (*E*)-3a

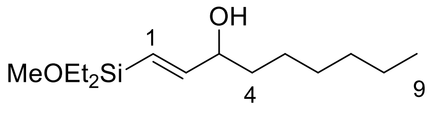

Isolated as a minor side product from the above reaction. $\mathbf{R_f}$ 0.12 (petrol / Et_2O (9:1)); **IR** (thin film, ν_{max} / cm^{-1}) 3392, 2956, 2930, 2858, 1739, 1622, 1460, 1413, 1378, 1237, 1188, 1088, 1007; **^{1}H NMR** (500 MHz, $CDCl_3$) δ_H 6.22 (1H, dd, J = 19.0and 5.3Hz, H2), 5.78 (1H, dd, J = 19.0 and 1.4Hz, H1), 4.18 – 4.12 (1H, m, H3), 3.46

(3H, s, OCH_3), 1.60 – 1.52 (2H, m, H4), 1.44 – 1.38 (1H, m, O*H*), 1.36 – 1.25 (8H, m, H5, H6, H7 and H8), 0.98 (6H, t, *J* = 7.9 Hz, Si(CH_2CH_3)$_2$), 0.89 (3H, t, *J* = 6.9 Hz, H9), 0.69 (4H, q, *J* = 7.9 Hz Si(CH_2CH_3)$_2$); **^{13}C NMR** (125 MHz, $CDCl_3$) δ_C 151.9, 123.1, 74.6, 50.8, 36.9, 31.8, 29.2, 25.3, 22.6, 14.0, 6.6, 4.6; **HRMS** (ES^+) calc. for $C_{14}H_{30}NaO_2Si$ $[M+Na]^+$ 281.1913, found 281.1907.

(*Z*)-Diethyl(isopropoxy)(3-((4-methoxybenzyl)oxy)non-1-en-1-yl)silane, 1z

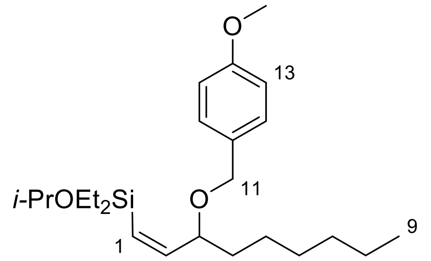

Palladium on $CaCO_3$ (139 mg, 5 wt % Pd, 0.066 mmol, 0.05 equiv.), **2z** (530 mg, 1.31 mmol, 1.0 equiv.) and quinoline (31 μL, 0.262 mmol, 0.2 equiv.) in toluene (7 mL) were stirred under an atmosphere of hydrogen for three hours before the reaction mixture was filtered through Celite® and concentrate. The crude (92:8 *Z*:*E*) was purified by flash column chromatography (petrol + 1% Et_3N) to give **1z** as a colourless oil (458 mg, 1.13 mmol, 86%, 93:7 *Z*:*E* (up to 97% *Z* in some fractions)); **R_f** 0.33 (petrol / Et_2O (19:1)); **IR** (thin film, ν_{max} / cm^{-1}) 2956, 2931, 2874, 1613, 1513, 1463, 1368, 1301, 1246, 1172, 1123, 1081, 1026; **^{1}H NMR** (400 MHz, $CDCl_3$) δ_H 7.26 (2H, d, *J* = 8.5 Hz, H12), 6.87 (2H, d, *J* = 8.5 Hz, H13), 6.33 (1H, dd, *J* = 14.7 and 9.1 Hz, H2), 5.63 (1H, d, *J* = 14.7 Hz, H1), 4.52 and 4.33 (2 × 1H, d, *J* = 11.3 Hz, diastereotopic H11), 4.22-4.18 (1H, m, H3), 4.04 (1H, sept, *J* = 6.0 Hz, OC*H*(CH_3)$_2$), 3.80 (3H, s, OCH_3), 1.68-1.59 (1H, m, 1 × H4), 1.53-1.44 (2H, m, 1 × H4 and 1 × H5), 1.40-1.26 (7H, m, 1 × H5, H6, H7 and H8), 1.18-1.15 (6H, m, OCH(CH_3)$_2$), 0.99 and 0.98 (2 × 3H, t, *J* = 7.6 Hz, diastereotopic Si(CH_2CH_3)$_2$), 0.89 (3H, t, *J* = 7.2 Hz, H9), 0.67 (4H, q, *J* = 7.6 Hz, Si(CH_2CH_3)$_2$); **^{13}C NMR** (101 MHz, $CDCl_3$) δ_C 159.0, 152.1, 131.4, 129.2, 127.5, 113.7, 79.3, 70.1, 65.3, 55.3, 36.0, 31.9, 29.5, 25.9, 25.5, 22.7, 14.1, 7.0, 6.9; **HRMS** (ES^+) calc. for $C_{24}H_{42}NaO_3Si$ $[M+Na]^+$ 429.2795, found 429.2780.

5-Cyclohexyl-2,2-diethyl-2,5-dihydro-1,2-oxasilole, 1d

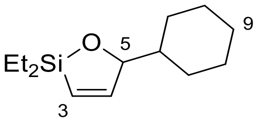

According to general procedure D, palladium on $CaCO_3$ (53 mg, 5 wt % Pd, 0.025 mmol, 0.05 equiv.), acetate **2d** (160 mg, 0.493 mmol, 1.0 equiv.) and quinoline (12 µL, 0.099 mmol, 0.2 equiv.) in toluene (6 mL) were stirred under a hydrogen atmosphere for two hours and gave, after purification by **rapid** flash column chromatography (petrol / Et_2O (19:1)), oxasilole **1d** as a colourless oil which was unstable to silica gel (89 mg, 0.397 mmol, 80% isolated); $\mathbf{R_f}$ 0.67 (petrol / Et_2O (4:1)); **IR** (thin film, ν_{max} / cm^{-1}) 2955, 2926, 2875, 2853, 1557, 1450, 1376, 1261, 1231, 1134, 1022; ^{1}**H NMR** (400 MHz, $CDCl_3$) δ_H 6.95 (1H, dd, J = 10.7 and 1.4 Hz, H3), 6.01 (1H, dd, J = 10.7 and 2.2 Hz, H4), 4.48 (1H, m, H5), 1.79-1.65 (6H, m, H8 and H9), 1.44 (1H, m, H6), 1.29-1.02 (4H, m, H7), 0.97 and 0.91 (2 × 3H, t, J = 8.0 Hz, diastereotopic Si(CH_2CH_3)), 0.77-0.57 (4H, m, Si(CH_2CH_3)$_2$); ^{13}**C NMR** (125 MHz, $CDCl_3$) δ_C 153.0, 125.0, 87.7, 44.2, 29.0, 28.5, 26.6, 26.3, 7.2, 6.9, 6.9, 6.5; **HRMS** (ES^+) calc. for $C_{13}H_{24}OSi$ $[M+]^+$ 224.1596 found 224.1591.

5-(2-((*Tert*-butyldimethylsilyl)oxy)ethyl)-2,2-diethyl-2,5-dihydro-1,2-oxasilole, 1e

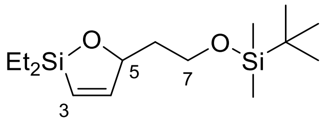

According to general procedure D, palladium on $CaCO_3$ (240 mg, 5 wt % Pd, 0.112 mmol, 0.05 equiv.), acetate **2e** (900 mg, 2.25 mmol, 1.0 equiv.) and quinoline (53 µL, 0.45 mmol, 0.2 equiv.) in toluene (15 mL) were stirred under a hydrogen atmosphere for three hours and gave, after purification by **rapid** flash column chromatography (petrol / Et_2O (50:1)), oxasilole **1e** as a colourless oil which was unstable to silica gel (540 mg, 1.80 mmol, 80% isolated); $\mathbf{R_f}$ 0.70 (petrol / Et_2O (4:1)); **IR** (thin film, ν_{max} / cm^{-1}) 2956, 2931, 2876, 2858, 1740, 1558, 1463, 1255, 1093; ^{1}**H NMR** (400 MHz,

$CDCl_3$) δ_H 6.93 (1H, dd, *J* = 10.6 and 1.5 Hz, H3), 5.95 (1H, dd, *J* = 10.6 and 2.2 Hz, H4), 4.79-4.75 (1H, m, H5), 3.82-3.78 (2H, m, H7), 1.87-1.79 and 1.66-1.57 (2 × 1H, m, diastereotopic H6), 0.95-0.90 (6H, m, diastereotopic $Si(CH_2CH_3)_2$), 0.90 (9H, s, $SiC(CH_3)_3$), 0.76-0.57 (4H, m, diastereotopic $Si(CH_2CH_3)_2$), 0.07 (6H, s, $Si(CH_3)_2$); **^{13}C NMR** (101 MHz, $CDCl_3$) δ_C 154.7, 123.9, 80.4, 60.2, 40.7, 25.9, 18.3, 7.2, 7.1, 6.8, 6.5, -5.4; **HRMS** (ES^+) calc. for $C_{15}H_{32}NaO_2Si_2$ $[M+Na]^+$ 323.1839, found 323.1830.

(*R*)-2,2-Diethyl-5-(2-((4-methoxybenzyl)oxy)ethyl)-2,5-dihydro-1,2-oxasilole, (*R*)-1f

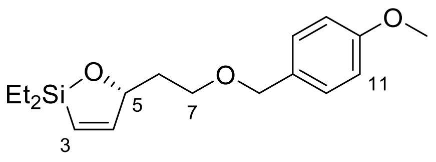

According to general procedure D, palladium on $CaCO_3$ (440 mg, 5 wt % Pd, 0.207 mmol, 0.05 equiv.), acetate (*R*)-**2f** (1.68 g, 4.13 mmol, 1.0 equiv.) and quinoline (101 µL, 0.826 mmol, 0.2 equiv.) in toluene (25 mL) were stirred under a hydrogen atmosphere for one hour and gave, after purification by **rapid** flash column chromatography (petrol / Et_2O (19:1)), oxasilole (*R*)-**1f** as a colourless oil which was unstable to silica gel (925 mg, 3.02 mmol, 73% isolated); $[\alpha]_D^{25}$ -40.9° (*c* 1.0, $CHCl_3$) for 97% ee; **R_f** 0.55 (petrol / EtOAc (4:1)); **IR** (thin film, ν_{max} / cm^{-1}) 2955, 2875, 1613, 1248, 1095, 1038; **^{1}H NMR** (400 MHz, $CDCl_3$) δ_H 7.28 (2H, d, *J* = 8.7 Hz, H10), 6.91 (1H, dd, *J* = 10.5 and 1.5 Hz, H3), 6.88 (2H, d, *J* = 8.7 Hz, H11), 5.96 (1H, dd, *J* = 10.5 and 2.2 Hz, H4), 4.82-4.79 (1H, m, H5), 4.47 and 4.45 (2 × 1H, d, *J* = 11.6 Hz, diastereotopic H8), 3.81 (3H, s, OCH_3), 3.68-3.59 (2H, m, H7), 1.97- 1.89 and 1.74-1.66 (2 × 1H, m, diastereotopic H6), 0.93 and 0.92 (2 × 3H, t, *J* = 7.9 Hz, diastereotopic $Si(CH_2CH_3)$), 0.76-0.58 (4H, m, $Si(CH_2CH_3)_2$); **^{13}C NMR** (101 MHz, $CDCl_3$) δ_C 159.1, 154.5, 130.7, 129.3, 124.1, 113.7, 80.7, 72.7, 67.0, 55.2, 37.7, 7.2, 7.1, 6.8, 6.5; **HRMS** (ES^+) calc. for $C_{17}H_{26}NaO_3Si$ $[M+Na]^+$ 329.1543, found 329.1536.

2,2,2-Diethyl-5-phenyl-2,5-dihydro-1,2-oxasilole, 1g

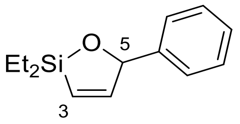

According to a modified version of general procedure E, palladium on $CaCO_3$ (90 mg, 5 wt % Pd, 0.04 mmol, 0.01 equiv.) was added to a stirred solution of silanol **2g** (990 mg, 4.22 mmol, 1.0 equiv.) and quinoline (0.25 mL, 0.211 mmol, 0.5 equiv.) in toluene (20 mL) and cyclohexene (2 mL). The solution was stirred under a hydrogen atmosphere for 40 minutes, then filtered through celite and concentrated as the cyclic product. The crude was purified by **rapid** flash column chromatography (petrol / Et_2O (19:1)) to give oxasilole **1g** as a colourless oil which was unstable to silica gel (569 g, 2.61 mmol, 62%); $\mathbf{R_f}$ 0.55 (petrol / Et_2O (19:1)); **IR** (thin film, ν_{max} / cm^{-1}) 2956, 2876, 1556, 1455, 1234, 1090, 1021; **^{1}H NMR** (500 MHz, $CDCl_3$) δ_H 7.37–7.25 (5H, m, Ar*H*), 6.99 (1H, dd, *J* = 10.6 and 1.6 Hz, H3), 6.11 (1H, dd, *J* = 10.6 and 2.5 Hz, H4), 5.71 (1H, m, H5), 1.02 and 1.00 (2 × 3H, t, *J* = 7.9 Hz, diastereotopic Si($CH_2C\mathit{H}_3$)), 0.86-0.68 (4H, m, Si($C\mathit{H}_2CH_3)_2$); **^{13}C NMR** (125 MHz, $CDCl_3$) δ_C 153.8, 142.1, 128.4, 127.5, 126.1, 124.5, 84.9, 7.4, 6.9, 6.9, 6.5; **HRMS** (ES^+) calc. for $C_{13}H_{18}NaOSi$ $[M+Na]^+$ 241.1025, found 241.1028.

(5*S*,6*R*)-2,2-Diethyl-6-[hydroxymethyl]-6-methyl-5,6-dihydro-2*H*-1,2-oxasilin-5-yl benzoate, 1i

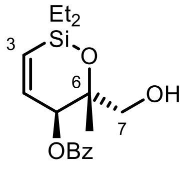

Following a modified version of general procedure E, a solution of palladium on $CaCO_3$ (16 mg, 5 w% Pd, 0.0075 mmol, 0.05 equiv.), **2h** (49 mg, 0.15 mmol, 1.0 equiv.) and quinoline (4 µL, 0.03 mmol, 0.2 equiv.) in toluene (2 mL) was stirred under an atmosphere of hydrogen for 4 hours. The reaction mixture was then filtered through Celite® and rinsed with CH_2Cl_2 (10 mL). Pyridinium *p*-toluenesulfonate (PPTS) (4 mg, 0.015 mmol, 0.1 equiv.) was then added to the filtrate and the resulting pale yellow solution stirred at room temperature for one hour. The reaction mixture

was concentrated *in vacuo* and the resulting residue purified *via* column chromatography (petrol / EtOAc (9:1)) to give oxasilole **1i** (45 mg, 0.14 mmol, 94%) as a colourless oil; $[\alpha]_D^{20}$ −4.22 (*c* 0.51, $CHCl_3$); **R_f** 0.20 (petrol / EtOAc (9:1)); **IR** (thin film, ν_{max} / cm^{-1}) 3406, 1606, 1503, 1310, 1269, 1096, 1007; **^{1}H NMR** (400 MHz, $CDCl_3$) δ_H 8.08–7.37 (5H, m, Ar*H*), 6.64 (1H, dd, *J* = 14.5 and 2.5 Hz, H4), 6.00 (1H, dd, *J* = 14.5 and 2.5, H3), 5.78 (1H, t, *J* = 2.5 Hz, H5), 3.48 (1H, dd, *J* = 11.5 and 7.5 Hz, 1 × H7), 3.43 (1H, dd, *J* = 11.5 and 4.5 Hz, 1 × H7), 2.32-2.23 (1H, m, CH_2OH), 1.34 (1H, s, CCH_3), 1.01 and 0.99 (2 × 3H, t, *J* = 8.0 Hz, diastereotopic $SiCH_2CH_3$), 0.78–0.55 (4H, m, $SiCH_2CH_3$); **^{13}C NMR** (100 MHz, $CDCl_3$) δ_C 165.7, 148.1, 133.2, 129.9, 129.7, 129.5, 128.5, 126.5, 71.5, 68.2, 19.3, 7.3, 6.7, 6.5, 6.3; **HRMS** (ESI+) calc. for $C_{17}H_{24}O_4Si$ [M+Na]+ 343.1336, found 343.1332.

(*R*)-2-((*S*)-2,2-Diethyl-2,5-dihydro-1,2-oxasilol-5-yl)propane-1,2-diol, 1h

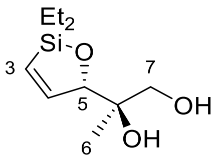

To a solution of **1i** (348 mg, 1.09 mmol, 1.0 equiv.) in MeOH (11 mL) was added K_2CO_3 (180 mg, 1.30 mmol, 1.2 equiv.). After stirring vigorously overnight at room temperature, the reaction mixture was quenched with a saturated aqueous NH_4Cl solution, the MeOH removed *in vacuo*, the resulting mixture extracted with Et_2O, dried over anhydrous Na_2SO_4, filtered and concentrated *in vacuo*. The crude product was purified *via* column chromatography (petrol / EtOAc (7:3)) to give **1h** as a colourless oil (198 mg, 0.92 mmol, 84%); **R_f** 0.24 (petrol / EtOAc (7:3)); $[\alpha]_D^{20}$ +79.2 (*c* 1.0, $CHCl_3$); **IR** (thin film, ν_{max} / cm^{-1}) 3396, 2957, 2877, 1557, 1114, 1030; **^{1}H NMR** ($CDCl_3$, 500 MHz) δ_H 7.00 (1H, dd, *J* = 10.5 and 1.5 Hz, H4), 6.16 (1H, dd, *J* = 10.5 and 2.5 Hz, H3), 4.67 (1H, dd, *J* = 2.5 and 1.5 Hz, H5), 3.64 and 3.40 (2 × 1H, d, *J* = 11.0 Hz, diastereotopic H7), 1.13 (3H, s, H6), 1.00 and 0.87 (2 × 3H, t, *J* = 8.0 Hz, diastereotopic $Si(CH_2CH_3)$), 0.74 (4H, q, *J* = 8.0 Hz, $Si(CH_2CH_3)$); **^{13}C NMR** ($CDCl_3$, 125 MHz) δ_C 150.1, 127.3, 87.6, 73.9, 67.5, 19.5, 7.1, 6.7, 6.3; **HRMS** (ES^+)

calc. for $C_{10}H_{20}O_3Si$ $[M+Na]^+$ 239.1074, found 239.1075; **EA** calc. for $C_{10}H_{20}O_3Si$: C, 55.52; H, 9.32. Found: C, 55.48, H, 9.25.

1-(2,2-Diethyl-2,5-dihydro-1,2-oxasilol-5-yl)-3-((4-methoxybenzyl)oxy)propan-1-ol, 1j

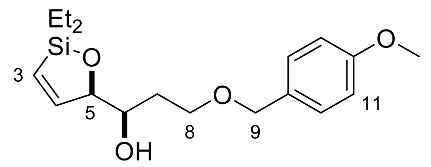

Following a modified version of general procedure E, a solution of palladium on $CaCO_3$ (11 mg, 5 wt % Pd, 0.005 mmol, 0.05 equiv.), **2j** (40 mg, 0.10 mmol, 1.0 equiv.) and quinoline (0.20 mL of a 0.1 M solution in toluene, 0.02 mmol, 0.2 equiv.) in toluene (1.5 mL) and cyclohexene (0.15 mL) was stirred under an atmosphere of hydrogen for 9.5 hours. The reaction mixture was filtered through Celite® and concentrated (cyclisation occurred *in situ*). The crude was purified by **rapid** flash column chromatography (petrol / EtOAc (4:1)) to give oxasilole **1j** as a colourless oil (22.5 mg, 0.067 mmol, 67%); $\mathbf{R_f}$ 0.75 (petrol / EtOAc (1:1)); **IR** (thin film, ν_{max} / cm^{-1}) 3031, 2955, 2875, 1613, 1513, 1462, 1247, 1095, 1031; **^{1}H NMR** (400 MHz, $CDCl_3$) δ_H 7.26 (2H, d, J = 8.6 Hz, H10), 6.91 (1H, dd, J = 10.7 and 1.5 Hz, H3), 6.88 (2H, d, J = 8.6 Hz, H11), 6.11 (1H, dd, J = 10.7 and 2.2 Hz, H4), 4.57 (1H, m, H5), 4.47 (2H, s, H9), 3.81 (3H, s, OCH_3), 3.71-3.63 (2H, m, H8), 2.82 (1H, d, J = 2.9 Hz, O*H*), 1.89-1.76 (2H, m, H7), 0.97 and 0.92 (2 × 3H, t, J = 7.7 Hz, diastereotopic $Si(CH_2CH_3)$), 0.75-0.62 (4H, m, $Si(CH_2CH_3)_2$); **^{13}C NMR** (101 MHz, $CDCl_3$) δ_C 159.0, 150.7, 130.5, 129.3, 127.2, 113.8, 86.5, 73.2, 72.8, 67.7, 55.4, 32.8, 7.27, 7.07, 6.93, 6.55; **HRMS** (ES+) calc. for $C_{18}H_{28}NaO_4Si$ $[M+Na]^+$ 359.1649, found 359.1644.

2,2-Diethyl-1-oxa-2-silaspiro[4.5]dec-3-ene, 1k

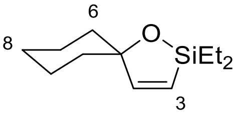

Following a modified version of general procedure E, a solution of palladium on $CaCO_3$ (78 mg, 5 wt% Pd, 0.037 mmol, 0.05 equiv.), **2k** (196 mg, 0.730 mmol, 1.0 equiv.) and quinoline (17 μL, 0.146 mmol, 0.2 equiv.) in toluene (7 mL) and cyclohexene (0.7 mL) was stirred under an atmosphere of hydrogen for two hours. The reaction mixture was filtered through Celite® and concentrated (cyclisation occurred *in situ*). The crude was purified by **rapid** flash column chromatography (petrol / Et_2O (19:1)) to give silaspiro **1k** as a colourless oil (116 mg, 0.551 mmol, 76%); $\mathbf{R_f}$ 0.15 (petrol); **IR** (thin film, ν_{max} / cm^{-1}) 2931, 2875, 1558, 1458, 1099, 1031; $\mathbf{^1H}$ **NMR** (400 MHz, $CDCl_3$) 6.94 (1H, d, J = 10.5 Hz, H3), 5.84 (1H, d, J = 10.5 Hz, H4), 1.74-1.65 (2H, m, 2 × H7), 1.57-1.48 (7H, m, 4 × H6, 2 × H7 and 1 × H8), 1.38-1.31 (1H, m, 1 × H8), 0.91 (2 × 3H, t, J = 8.0 Hz, diastereotopic $Si(CH_2CH_3)$), 0.74-0.55 (4H, m, $Si(CH_2CH_3)_2$); $\mathbf{^{13}C}$ **NMR** (101 MHz, $CDCl_3$) δ_C 159.0, 122.7, 85.4, 38.5, 25.5, 22.7, 7.5, 7.0; **HRMS** (FI^+) calc. for $C_{12}H_{22}OSi$ $[M+]^+$ 210.1440, found 210.1445.

2,2-diethyl-6-hexyl-5,6-dihydro-2H-1,2-oxasiline, 1l

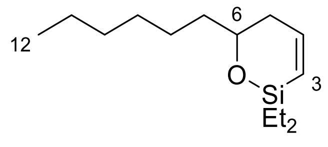

Following general procedure E, a solution of palladium on $CaCO_3$ (108 mg, 5 wt% Pd, 0.050 mmol, 0.05 equiv.), **2l** (303 mg, 1.01 mmol, 1.0 equiv.) and quinoline (60 μL, 0.507 mmol, 0.5 equiv.) in toluene (10 mL) was stirred under an atmosphere of hydrogen for one hour. The crude was purified by flash column chromatography (petrol / Et_2O (19:1)) to give oxasiline **1l** as a colourless oil (173 mg, 0.720 mmol,

71%); **R$_f$** 0.26 (petrol / Et_2O (99:1)); **IR** (thin film, ν_{max} / cm^{-1}) 2956, 2927, 2875, 1588, 1460, 1353, 1234, 1085, 1005; **^{1}H NMR** (400 MHz, $CDCl_3$) δ_H 6.85 (1H, ddd, *J* = 14.1, 5.4 and 2.9 Hz, H4), 5.72 (1H, ddd, *J* = 14.1, 2.5 and 1.2 Hz, H3), 3.92-3.86 (1H, m, H6), 2.17-2.04 (2H, m, H5), 1.58-1.50 (1H, m, 1 × H7), 1.47-1.37 (2H, m, 1 × H7 and 1 × H8), 1.34-1.23 (7H, m, 1 × H8, H9, H10 and H11), 0.98 and 0.93 (2 × 3H, t, *J* = 7.9 Hz, diastereotopic Si($CH_2C\mathit{H}_3$)), 0.88 (3H, t, *J* = 7.0 Hz, H12), 0.68-0.52 (4H, m, Si($C\mathit{H}_2CH_3)_2$); **^{13}C NMR** (101 MHz, $CDCl_3$) δ_C 148.5, 124.9, 71.8, 38.0, 36.6, 31.9, 29.3, 25.5, 22.7, 14.1, 6.8, 6.7, 6.5, 5.9; **HRMS** (FI^+) calc. for $C_{14}H_{28}OSi$ $[M+]^+$ 240.1909, found 240.1903.

6-cyclohexyl-2,2-diethyl-5,6-dihydro-2H-1,2-oxasiline, 1m

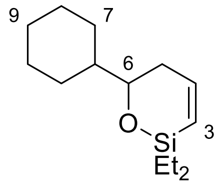

Following a modified version of general procedure E, a solution of palladium on $CaCO_3$ (300 mg of a 5 wt% solid, 0.141 mmol, 0.05 equiv.), **2m** (837 mg, 2.82 mmol, 1.0 equiv.) and quinoline (170 μL, 0.141 mmol, 0.5 equiv.) in THF (30 mL) was stirred under an atmosphere of hydrogen for one hour. The crude was purified by flash column chromatography (petrol / Et_2O (19:1)) to give oxasiline **1m** as a colourless oil (447 mg, 1.88 mmol, 66%); **R$_f$** 0.45 (petrol / Et_2O (99:1)); **IR** (thin film, ν_{max} / cm^{-1}) 2954, 2853, 1588, 1450, 1351, 1234, 1048; **^{1}H NMR** (400 MHz, $CDCl_3$) δ_H 6.81 (1H, dt, *J* = 14.0 and 4.2 Hz, H4), 5.65 (1H, dt, *J* = 14.0 and 1.8 Hz, H3), 3.58-3.51 (1H, m, H6), 2.07-2.04 (2H, m, H7), 1.89-1.83 (1H, m, 1 × H8), 1.69-1.64 and 1.61-1.55 (2 × 1H, m, H9), 1.34-1.25 (1H, m, H7), 1.24-0.84 (11H, m, 3 × H8, H10 and Si($CH_2C\mathit{H}_3)_2$), 0.60-0.46 (4H, m, Si($C\mathit{H}_2CH_3)_2$); **^{13}C NMR** (400 MHz, $CDCl_3$) δ_C 148.9, 124.9, 76.0, 44.2, 33.4, 28.8, 28.8, 26.7, 26.3, 26.2, 6.9, 6.7, 6.5, 5.9; **HRMS** (FI^+) calc. for $C_{14}H_{26}OSi$ $[M]^+$ 238.1753, found 238.1758.

6-(2-((*Tert*-butyldimethylsilyl)oxy)ethyl)-2,2-diethyl-5,6-dihydro-2H-1,2-oxasiline, 1n

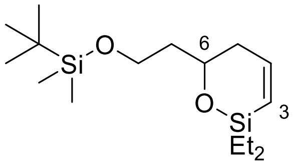

Following a modified version of general procedure E, a solution of palladium on $CaCO_3$ (12 mg of a 5 wt% solid, 0.005 mmol, 0.05 equiv.), **2n** (34 mg, 0.091 mmol, 1.0 equiv.) and quinoline (2.1 µL, 0.018 mmol, 0.2 equiv.) in toluene (1 mL) was stirred under an atmosphere of hydrogen for 15 minutes. The crude was purified by flash column chromatography (petrol / Et_2O (19:1)) to give oxasiline **1n** as a colourless oil (21 mg, 0.067 mmol, 73%); **R_f** 0.25 (petrol / Et_2O (99:1)); **IR** (thin film, ν_{max} / cm^{-1}) 2987, 2955, 2877, 1588, 1471, 1463, 1255, 1092; **^1H NMR** (400 MHz, $CDCl_3$) δ_H 6.86 (1H, dt, J = 14.0 and 4.0 Hz, H4), 5.74 (1H, dt, J = 14.0 and 2.0 Hz, H3), 4.14-4.07 (1H, m, H6), 3.81-3.75 and 3.73-3.68 (2 × 1H, m, diastereotopic H8), 2.16-2.12 (2H, m, H5), 1.76-1.62 (2H, m, H7), 0.98 and 0.94 (2 × 3H, t, J = 8.0 Hz, diastereotopic Si($CH_2C\mathit{H}_3$)), 0.89 (9H, s, SiC($C\mathit{H}_3$)$_3$), 0.69-0.52 (4H, m, Si($C\mathit{H}_2CH_3$)$_2$), 0.05 (6H, s, Si($C\mathit{H}_3$)$_2$); **^{13}C NMR** (101 MHz, $CDCl_3$) δ_C 148.5, 124.9, 68.4, 59.5, 41.1, 36.8, 26.0, 18.3, 6.8, 6.7, 6.4, 5.9, -5.4; **HRMS** (ES^+) calc. for $C_{16}H_{34}NaO_2Si_2$ $[M+Na]^+$ 337.1990, found 337.1983.

(*R*)-2,2-Diethyl-6-(2-((4-methoxybenzyl)oxy)ethyl)-5,6-dihydro-2H-1,2-oxasiline, (*R*)-1o

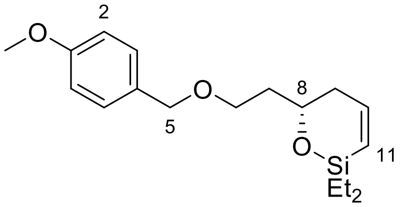

Following general procedure E, a solution of palladium on $CaCO_3$ (14 mg, 5 wt% Pd, 0.007 mmol, 0.05 equiv.), (*R*)-**2o** (50 mg, 0.132 mmol, 1.0 equiv.) and quinoline (8 µL, 0.066 mmol, 0.5 equiv.) in toluene (1.3 mL) was stirred under an atmosphere of

hydrogen for 10 minutes. The crude was purified by flash column chromatography (petrol / Et_2O (19:1)) to give oxasiline (*R*)-**1o** as a colourless oil (34 mg, 0.106 mmol, 79%); $[\alpha]_D^{25}$ +23.9° (*c* 1.0, $CHCl_3$); **R$_f$** 0.52 (petrol / Et_2O (2:1)); **IR** (thin film, ν_{max} / cm^{-1}) 2986, 2875, 1613, 1587, 1513, 1248, 1094, 1037; **^{1}H NMR** (400 MHz, $CDCl_3$) δ_H 7.27 (2H, d, *J* = 8.8 Hz, H3), 6.90-6.82 (3H, m, H2 and H10), 5.73 (1H, dt, *J* = 14.3 and 1.8 Hz, H11), 4.45 and 4.43 (2 × 1H, d, *J* = 11.7 Hz, diastereotopic H5), 4.15-4.09 (1H, m, H8), 3.81 (3H, s, OCH_3), 3.67-3.54 (2H, m, H6), 2.15-2.12 (2H, m, H9), 1.81-1.75 (2H, m, H7), 0.99 and 0.94 (2 × 3H, t, *J* = 7.8 Hz, diastereotopic $Si(CH_2CH_3)$), 0.69-0.54 (4H, m, $Si(CH_2CH_3)_2$); **^{13}C NMR** (101 MHz, $CDCl_3$) δ_C 159.1, 148.4, 130.7, 129.3, 124.8, 113.7, 72.8, 68.7, 66.6, 55.3, 38.0, 36.8, 6.8, 6.7, 6.4, 5.9; **HRMS** (ES^+) calc. for $C_{18}H_{28}NaO_3Si$ $[M+Na]^+$ 343.1705, found 343.1695.

2,2-Diethyl-4a,5,6,7,8,8a-hexahydro-2H-benzo[e][1,2]oxasiline, 1p

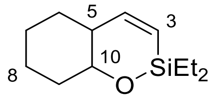

Following general procedure E, a solution of palladium on $CaCO_3$ (176 mg, 5 wt% Pd, 0.083 mmol, 0.05 equiv.), **2p** (444 mg, 1.65 mmol, 1.0 equiv.) and quinoline (39 µL, 0.330 mmol, 0.5 equiv.) in toluene (17 mL) was stirred under an atmosphere of hydrogen for 50 minutes. The crude was purified by flash column chromatography (petrol / Et_2O (19:1)) to give oxasiline **1p** as a colourless oil (278 mg, 1.07 mmol, 65%); **R$_f$** 0.31 (petrol / Et_2O (99:1)); **IR** (thin film, ν_{max} / cm^{-1}) 2930, 2875, 2856, 1584, 1449, 1130, 1073, 1004; **^{1}H NMR** (500 MHz, $CDCl_3$) δ_H) 6.56 (1H, d, *J* = 14.1 Hz, H3), 5.71 (1H, dd, *J* = 14.1 and 3.5 Hz, H4), 3.50-3.45 (1H, m, H10), 2.00-1.93 (2H, m, H5, 1 × H9), 1.86-1.75 (2H, m, 1 × H6 and 1 × H8), 1.70-1.65 (1H, m, 1 × H7), 1.42-1.21 (3H, m, 1 × H7, 1 × H8 and 1 × H9), 1.13-1.04 (1H, m, 1 × H6), 0.99 and 0.94 (2 × 3H, t, *J* = 8.0 Hz, diastereotopic $Si(CH_2CH_3)$), 0.70-0.53 (4H, m, $Si(CH_2CH_3)_2$); **^{13}C NMR** (125 MHz, $CDCl_3$) δ_C 155.0, 124.2, 76.3, 45.3, 35.3, 31.7, 25.9, 25.0, 6.7, 6.8, 6.8, 6.4; **HRMS** (FI^+) calc. for $C_{12}H_{22}O_3Si$ $[M+]^+$ 210.1440, found 210.1446.

1.6 Synthesis of Iodide Coupling Partners

1.6.1 General Scheme for Synthesis of Iodide Coupling Partners

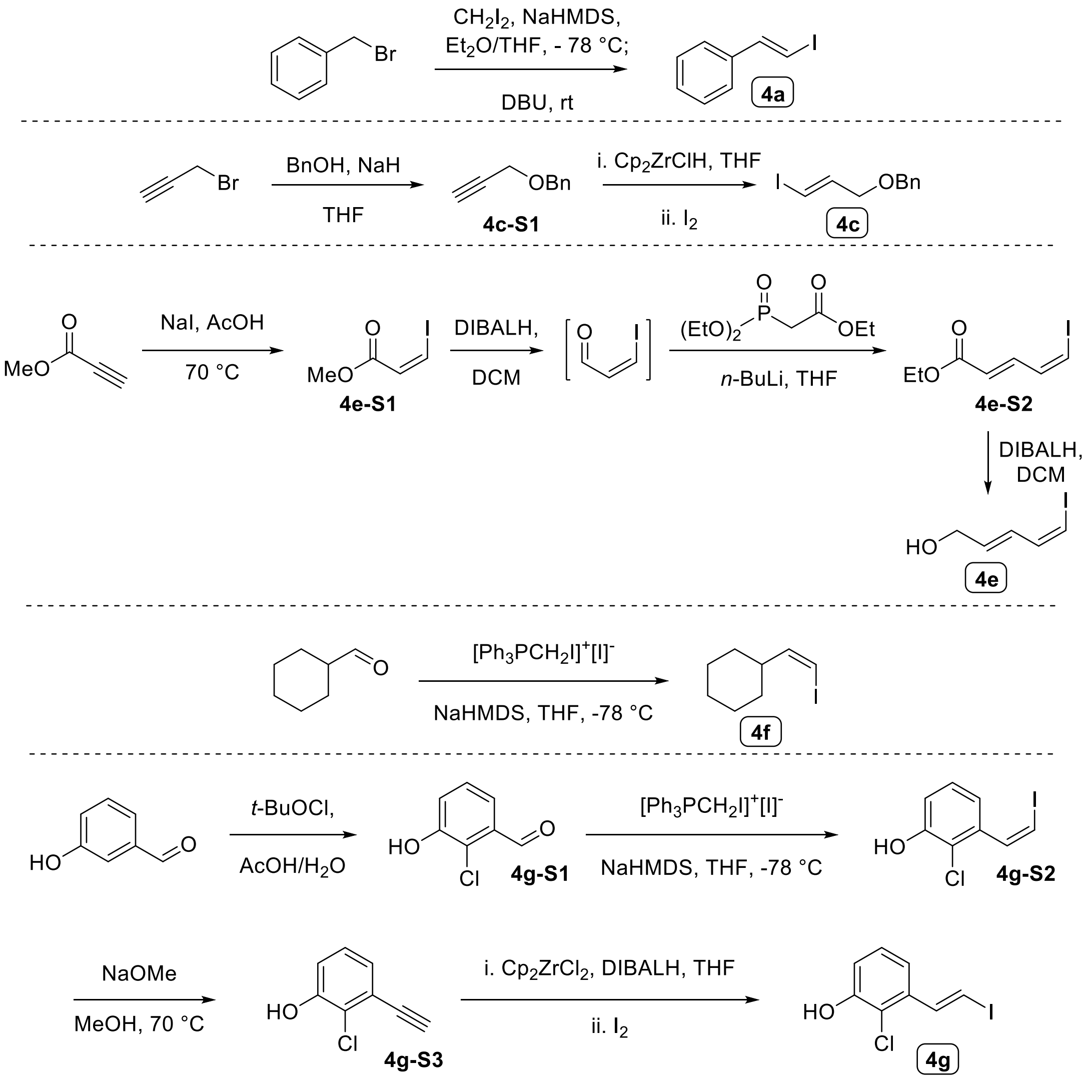

Preparation of 4a

E-Iodostyrene, 4a

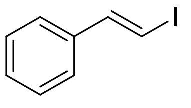

Prepared according to a literature procedure.[21] Diiodomethane (2.0 mL, 24.8 mmol, 1.5 equiv.) in THF (6 mL) was added over 30 minutes in the dark to a solution of sodium bis(trimethylsilyl)amide (25 mL of a 2.0 M solution in THF, 49.7 mmol, 3.0 equiv.) in Et_2O (15 mL) at - 78 ºC and stirred for 20 minutes before benzyl bromide (1.97 mL, 16.6 mmol, 1.0 equiv.) in THF (10 mL) was added over 15 minutes. The mixture was stirred for a further 90 minutes in the dark at –78 ºC, before warming to room temperature over 30 minutes. 1,8-Diazabicycloundec–7-ene (2.5 mL, 16.6 mmol, 1.0 equiv.) was added dropwise and the reaction stirred at room temperature for two hours. The mixture was then diluted with Et_2O (100 mL) and filtered through a plug of Celite® over silica gel and concentrated *in vacuo*. The crude was purified by flash column chromatography (petrol) to give iodostyrene, **4a** as a yellow oil (3.56 g, 15.5 mmol, 93%); $\mathbf{R_f}$ 0.40 (petrol); **^{1}H NMR** ($CDCl_3$, 250 MHz) δ_H 7.45 (1H, d, *J* = 14.9 Hz, C=C*H*I), 7.37–7.27 (5H, m, Ar*H*), 6.83 (1H, d, *J* = 14.9 Hz, C=C*H*Ar); **^{13}C NMR** (101 MHz, $CDCl_3$) δ_C 145.0, 137.7, 128.8, 128.4, 126.1, 76.9.

The spectroscopic data were found to be in agreement with that reported by Charette and co-workers.[21]

Preparation of 4c

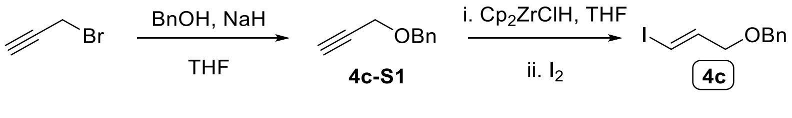

((Prop-2-yn-1-yloxy)methyl)benzene, 4c-S1

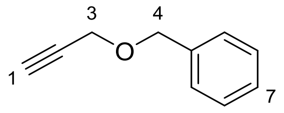

Prepared according to a literature procedure.[22] Benzyl alcohol (1.00 mL, 9.66 mmol, 1.0 equiv.) was added slowly to a suspension of sodium hydride (464 mg, 60 wt% dispersion in mineral oil, 11.6 mmol, 1.2 equiv.) in THF (10 mL) at 0 °C. The reaction was stirred until the evolution of hydrogen gas subsided, then propargyl bromide (1.1 mL, 80% in toluene, 11.6 mmol, 1.2 equiv.) was added and the mixture warmed slowly to room temperature and stirred for 24 hours. Water was added, the mixture separated and the aqueous layer extracted twice with Et_2O. The combined organic phase was dried ($MgSO_4$), concentrated and purified *via* flash column chromatography (petrol / Et_2O (100:0→99:1)) to give propargylic **4c-S1** as a colourless oil (1.12 g, 7.68 mmol, 80%); **R_f** 0.26 (petrol); **^{1}H NMR** ($CDCl_3$, 250 MHz) δ_H 7.38–7.27 (5H, m, H5, H6 and H7), 4.62 (2H, s, H4), 4.18 (2H, d, J = 2.4 Hz, H3), 2.47 (1H, t, J = 2.4 Hz, H1); **^{13}C NMR** (101 MHz, $CDCl_3$) δ_C 137.4, 128.5, 128.2, 128.0, 79.7, 74.7, 71.6, 57.1. **LRMS** (ES^+) calc. for $C_{10}H_{10}NaO$ $[M+Na]^+$ 169.1, found 169.1.

The spectroscopic data were found to be in agreement with that reported by Li and co-workers.[22]

(*E*)-(((3-Iodoallyl)oxy)methyl)benzene, 4c

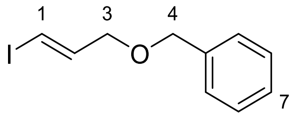

Prepared according to a modified literature procedure.[11] Alkyne **4c-S1** (0.50 mL, 3.46 mmol, 1.0 equiv.) was added to a suspension of Cp_2ZrHCl (936 mg, 3.63 mmol, 1.05 equiv.) in THF (20 mL) at room temperature and stirred for 15 minutes until the

solution became clear. The reaction was cooled to 0 °C, iodine added (1.05 g, 4.15 mmol, 1.2 equiv.) and the mixture stirred at 0 °C for two hours, then room temperature for 4 hours. The reaction was diluted with wet Et_2O (10 mL), $Na_2S_2O_3$ (30 mL) added, and stirred for one hour, then separated, the aqueous extracted with Et_2O and the combined organic phase dried ($MgSO_4$) and concentrated. The crude residue was purified *via* flash column chromatography (petrol / Et_2O (100:0→99:1)) to give iodide **4c** as a pale yellow oil (720 mg, 2.63 mmol, 76%); **R_f** 0.11 (petrol / Et_2O (99:1)); **^{1}H NMR** ($CDCl_3$, 400 MHz) δ_H 7.40–7.30 (5H, m, H5, H6 and H7), 6.66 (1H, dt, *J* = 14.4 and 5.6 Hz, H2), 4.43 (1H, d, *J* = 14.4 Hz, H1), 4.53 (2H, s, H4), 3.97 (2H, d, *J* = 5.6 Hz, H3); **^{13}C NMR** (101 MHz, $CDCl_3$) δ_C 142.2, 137.8, 128.5, 127.9, 127.8, 78.9, 72.4, 71.8. **LRMS** (ES^+) calc. for $C_{10}H_{11}ILiO$ $[M+Li]^+$ 281.0, found 281.0.

The spectroscopic data were found to be in agreement with that reported by Hu and co-workers.[23]

Preparation of 4e

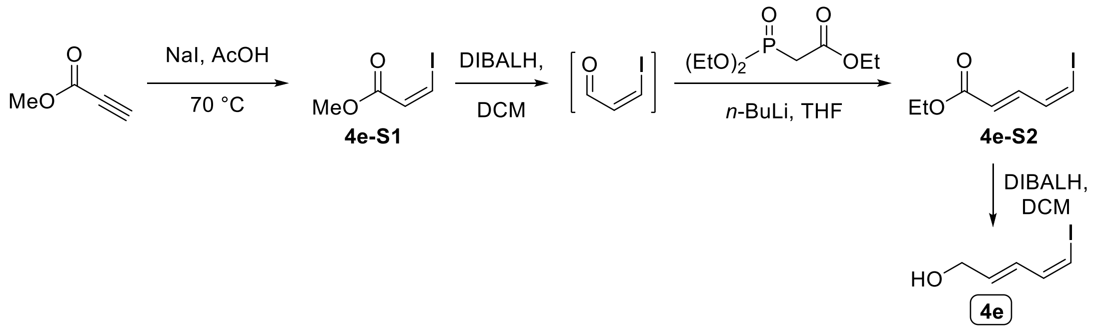

(*Z*)-Methyl 3-iodoacrylate, 4e-S1

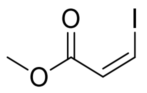

Prepared according to a literature procedure.[24] Sodium iodide (1.68 g, 11.2 mmol, 2.0 equiv.) was added to a solution of methyl propiolate (0.50 mL, 5.62 mmol, 1.0 equiv.)

in acetic acid (3 mL) and stirred at 70 ºC for 16 hours. The reaction was then diluted with Et_2O and 1 M NaOH (aq) added (3 mL). The aqueous layer was extracted with Et_2O, and the combined organic layers washed with K_2CO_3 and $NaHCO_3$ solutions, then dried ($MgSO_4$) and concentrated *in vacuo* to give iodide **4e-S1** as a brown oil (1.20 g, ~5.62 mmol, ~100%); **R_f** 0.31 (petrol / Et_2O (9:1)); **^{1}H NMR** ($CDCl_3$, 400 MHz) δ_H 7.49 (1H, d, J = 8.9 Hz, C*H*I=CHCO$_2$CH$_3$), 6.93 (1H, d, J = 8.9 Hz, CHI=C*H*CO$_2$CH$_3$), 3.80 (3H, s, CHI=CHCO$_2$C*H*$_3$); **^{13}C NMR** (101 MHz, $CDCl_3$) δ_C 165.0, 129.5, 95.2, 51.7; **LRMS** (ES$^+$) calc. for $C_4H_5INaO_2$ [M+Na]$^+$ 234.9, found 234.9.

The spectroscopic data were found to be in agreement with that reported by Spino and co-workers.[24]

(2*E*,4*Z*)-Ethyl 5-iodopenta-2,4-dienoate, 4e-S2

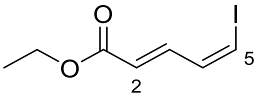

Prepared according to a literature procedure.[25] Diisobutylaluminium hydride (6.6 mL of a 1 M solution in hexanes, 6.60 mmol, 1.1 equiv.) was added slowly over 10 minutes to a solution of iodide **4e-S1** (1.27 g, 6.00 mmol, 1.0 equiv.) in CH_2Cl_2 (12 mL) at –78 ºC and stirred for a further 5 minutes. Methanol (1 mL) was added, followed by aqueous sodium/potassium tartrate solution (24 mL) and the mixture was warmed to room temperature. Et_2O (12 mL) was added and the reaction stirred in the dark for one hour, before being diluted with Et_2O and water, and the aqueous layer extracted with Et_2O. The combined organic extracts were washed with brine, dried (K_2CO_3) and concentrated *in vacuo*. The aldehyde obtained was used immediately in the next step without further purification.

n-Butyllithium (2.5 mL of a 2.5 M solution in hexanes, 6.30 mmol, 1.05 equiv.) was added to a solution of triethylphosphonoacetate (1.25 mL, 6.30 mmol, 1.05 equiv.) in THF (10 mL) at –78 ºC and stirred for 30 minutes, before a THF solution of the

intermediate aldehyde previously prepared was added *via* cannula. The mixture was stirred in the dark for two hours while warming to room temperature, and then a further hour once this had been reached. Et_2O and water were added, the aqueous phase extracted with Et_2O and the combined organic extracts washed with brine, dried ($MgSO_4$) and concentrated *in vacuo*. The crude was purified by flash column chromatography (petrol / EtOAc (97:3)) to give vinyl iodide **4e-S2** as a pale orange oil (789 mg, 3.13 mmol, 52%, 99:1 *E,Z*:*E,E*); $\mathbf{R_f}$ 0.31 (petrol / Et_2O (9:1)); **^{1}H NMR** (400 MHz, $CDCl_3$) δ_H 7.41 (1H, dd, *J* = 15.3 and 10.3 Hz, H3), 6.91 (1H, dd, *J* = 10.3 and 7.9 Hz, H4), 6.84 (1H, d, *J* = 7.9 Hz, H5), 6.14 (1H, d, *J* = 15.3 Hz, H2), 4.25 (2H, q, *J* = 7.1 Hz, CH_2CH_3), 1.33 (3H, t, *J* = 7.1 Hz, CH_2CH_3); **^{13}C NMR** (101 MHz, $CDCl_3$) δ_C 166.4, 143.0, 136.6, 125.8, 92.0, 60.7, 14.3; **HRMS** (EI^+) calc. for $C_7H_9IO_2$ $[M]^+$ 251.9647, found 251.9649.

The spectroscopic data were found to be in agreement with that reported by Trost and co-workers.[25]

(2*E*,4*Z*)-5-Iodopenta-2,4-dien-1-ol, 4e

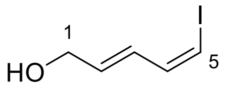

Prepared according to a literature procedure.[25] Diisobutylaluminium hydride (7.7 mL of a 1 M solution in hexanes, 7.70 mmol, 2.5 equiv.) was added dropwise a solution of ester **4e-S2** (780 mg, 3.10 mmol, 1.0 equiv.) in CH_2Cl_2 (8 mL) at 0 ºC and stirred for 50 minutes in the dark. The reaction was then quenched with aqueous sodium/potassium tartrate solution (8 mL), stirred for 5 minutes, the aqueous phase extracted with Et_2O, and the combined organic extracts dried ($MgSO_4$) and concentrated *in vacuo*. The crude was purified by flash column chromatography (petrol / EtOAc (3:1)) to give vinyl iodide **4e** as a pale yellow oil (470 mg, 2.24 mmol, 72%, 99:1 *E,Z*:*E,E*); $\mathbf{R_f}$ 0.10 (petrol / Et_2O (9:1)); **^{1}H NMR** (400 MHz, $CDCl_3$) δ_H 6.76 (1H, dd, *J* = 10.3 and 7.4 Hz, H4), 6.44 (1H, dd, *J* = 15.3 and 10.3 Hz, H3), 6.29 (1H, d, *J* = 7.4 Hz, H5), 6.12 (1H, dt, *J* = 15.3 and 5.1 Hz, H2), 4.26-

4.22 (2H, m, H1), 1.99 (1H, br s, O*H*); **^{13}C NMR** (101 MHz, $CDCl_3$) δ_C 137.6, 137.1, 130.7, 82.8, 63.1; **HRMS** (EI^+) calc. for C_5H_7IO $[M]^+$ 209.9542, found 209.9542.

The spectroscopic data were found to be in agreement with that reported by Trost and co-workers.[25]

Preparation of 4f

(*Z*)-(2-Iodovinyl)cyclohexane, 4f

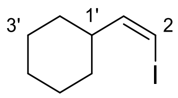

Prepared according to a modified literature procedure.[26] Sodium bis(trimethylsilyl)amide (0.91 mL of a 2 M solution in THF, 1.82 mmol, 1.1 equiv.) was added dropwise at room temperature to a suspension of Stork-Wittig reagent $[Ph_3PCH_2I]^+[I]^-$ (960 mg, 1.82 mmol, 1.1 equiv.) in THF (8 mL) and stirred for 10 minutes. This solution was then cooled to –78 ºC, and cyclohexanecarboxaldehyde (0.20 mL, 1.65 mmol, 1.0 equiv.) added dropwise. The mixture was stirred at –78 ºC in the dark for 4 hours, then quenched with $NaHCO_3$ solution, diluted with petrol, and filtered through a pad of Celite®. The biphasic mixture was separated and the organic phase dried ($MgSO_4$) and concentrated *in vacuo*. The crude was purified by flash column chromatography (petrol) to give vinyl iodide **4f** as a colourless oil (230 mg, 0.974 mmol, 59%, >99:1 *Z*:*E*); **R_f** 0.64 (petrol); **^{1}H NMR** (400 MHz, $CDCl_3$) δ_H 6.07 (1H, d, $J = 7.4$ Hz, H2), 5.99 (1H, dd, $J = 8.4$ and 7.4 Hz, H1), 2.37-2.28 (1H, m, H1’), 1.75-1.70 (4H, m, H2’), 1.39-1.29 (2H, m, H4’), 1.24-1.09 (4H, m, H3’); **^{13}C NMR** (101 MHz, $CDCl_3$) δ_C 146.3, 79.6, 43.6, 31.1, 25.9, 25.5.

The spectroscopic data were found to be in agreement with that reported by Beshai and co-workers.[26]

Preparation of 4g

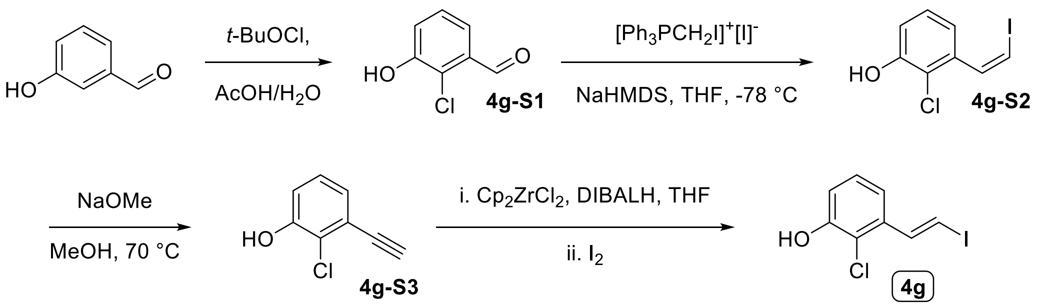

2-Chloro-3-hydroxybenzaldehyde, 4g-S1

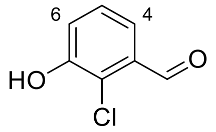

Prepared according to a literature procedure.[27] Freshly prepared *t*-butyl hypochlorite[28] (4.9 mL, 43 mmol, 1.05 equiv.) was added dropwise in the dark to 3-hydroxybenzaldehyde (5.00 g, 41 mmol, 1.0 equiv.) in 90% acetic acid (20 mL). The mixture was stirred for three hours, before a colourless precipitate was removed by filtration and recrystallised from a hot solution of 50 % acetic acid/water to give the chlorinated product as tan-coloured crystals (2.52 g, 16.1 mmol, 39%); $\mathbf{R_f}$ 0.32 (petrol / Et_2O (1:1)); **mp** 136-137 ºC (lit.[27] 137-138 ºC); ^{1}**H NMR** (400 MHz, $CDCl_3$) δ_H 10.41 (1H, s, C*H*O), 7.53 (1H, dd, *J* = 7.5 and 1.9 Hz, H6), 7.34 (1H, t, *J* = 7.5 Hz, H5), 7.30 (1H, dd, *J* = 7.5 and 1.9 Hz, H4), 5.82 (1H, br s, O*H*); ^{13}**C NMR** (101 MHz, $CDCl_3$) δ_C 189.3, 152.1, 132.7, 128.1, 122.8, 122.1, 121.8; **LRMS** (ES^-) calc. for $C_7H_4ClO_2$ [^{35}ClM-H]$^-$ 154.99, found 154.99.

The physical and spectroscopic data were found to be in agreement with that reported by Giles and co-workers.[27]

(*Z*)-2-Chloro-3-(2-iodovinyl)phenol, 4g-S2

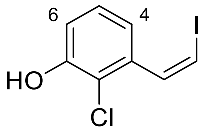

Prepared according to a modified literature procedure.[26] Sodium bis(trimethylsilyl)amide (1.9 mL of a 2 M solution in THF, 3.80 mmol, 1.2 equiv.) was added dropwise to a solution of aldehyde **4g-S1** (500 mg, 3.19 mmol, 1.0 equiv.) in THF (5 mL) at –78 ºC and stirred at that temperature for 20 minutes. Meanwhile, sodium bis(trimethylsilyl)amide (1.8 mL of a 2 M solution in THF, 3.60 mmol, 1.1 equiv.) was added dropwise to a suspension of Stork-Wittig reagent $[Ph_3PCH_2I]^+[I]^-$ (1.86 g, 3.51 mmol, 1.1 equiv.) in THF (20 mL) at room temperature and stirred for 15 minutes. This solution was then cooled to –78 ºC, and the solution of deprotonated aldehyde added *via* cannula. The mixture was stirred at –78 ºC in the dark for 5 hours, then quenched with $NaHCO_3$ solution, diluted with Et_2O, and filtered through a pad of Celite®. The biphasic mixture was separated and the organic phase dried ($MgSO_4$) and concentrated *in vacuo*. The crude was purified by flash column chromatography (petrol / Et_2O (19:1)) to give vinyl iodide **4g-S2** as a yellow oil (444 mg, 1.59 mmol, 50%, >99:1 *Z*:*E*); **R_f** 0.23 (petrol / EtOAc (4:1)); **IR** (thin film, ν_{max} / cm^{-1}) 3512, 1594, 1578, 1474, 1466, 1435, 1300, 1262, 1198, 1166, 1099, 1042; **^{1}H NMR** (400 MHz, $CDCl_3$) δ_H 7.35 (1H, d, *J* = 8.6 Hz, ArCH=C*H*I), 7.27–7.21 (2H, m, H4 and H5), 7.04 (1H, dd, *J* = 6.8 and 3.0 Hz, H6), 6.79 (1H, d, *J* = 8.6 Hz, ArC*H*=CHI), 5.61 (1H, br s, O*H*); **^{13}C NMR** (101 MHz, $CDCl_3$) δ_C 151.6, 136.5, 136.4, 127.3, 121.7, 119.0, 115.8, 84.3; **HRMS** (ES^+) calc. for C_8H_5ClIO $[^{35}ClM]^-$ 278.9079, found 278.9086.

2-Chloro-3-ethynylphenol, 4g-S3

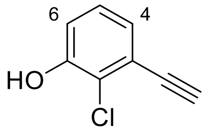

Prepared according to a modified literature procedure.[29] Freshly prepared sodium methoxide solution (1.8 mL of a 1 M solution in MeOH, 1.80 mmol, 2.5 equiv.) was added to *Z*-iodide **4g-S2** (200 mg, 0.715 mmol, 1.0 equiv.) and heated to 70 ºC for 24 hours. The mixture was then quenched with NH_4Cl solution and extracted with CH_2Cl_2, dried ($MgSO_4$) and concentrated *in vacuo*. The crude was used in the next step without further purification as a brown oil (96 mg, 0.629 mmol, 88%); $\mathbf{R_f}$ 0.23 (petrol / EtOAc (4:1)); **IR** (thin film, ν_{max} / cm^{-1}) 3510, 3293, 2103, 1578, 1462, 1439, 1288, 1251, 1188; **^{1}H NMR** (400 MHz, $CDCl_3$) δ_H 7.18–7.13 (2H, m, H4 and H5), 7.07–7.02 (1H, m, H6), 5.62 (1H, br s, O*H*), 3.38 (1H, s, C≡C*H*); **^{13}C NMR** (101 MHz, $CDCl_3$) δ_C 151.6, 127.7, 126.0, 122.4, 122.0, 116.9, 82.5, 79.9; **HRMS** (ES$^-$) calc. for C_8H_4ClO [^{35}ClM-H]$^-$ 150.9956, found 150.9955.

(*E*)-2-Chloro-3-(2-iodovinyl)phenol, 4g

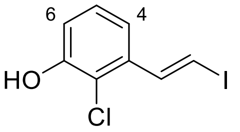

Prepared according to a literature procedure.[30] Diisobutylaluminium hydride (0.69 mL of a 1 M solution in hexanes, 0.690 mmol, 1.1 equiv.) was added dropwise to zirconocenedichloride (202 mg, 0.692 mmol, 1.1 equiv.), in THF (2 mL) at 0 ºC and stirred for 30 minutes before a solution of alkyne **4g-S3** (96 mg, 0.629 mmol, 1.0 equiv.) in THF (1.5 mL) was added *via* cannula. The mixture was stirred at that temperature for a further 40 minutes before cooling to –78 ºC. A solution of iodine (208 mg, 0.818 mmol, 1.3 equiv.) in THF (1 mL) was added *via* cannula and the mixture stirred for two hours, then quenched with 1 M HCl solution, extracted with Et_2O and washed sequentially with $Na_2S_2O_3$, $NaHCO_3$ and brine solutions. The

organic layer was then dried ($MgSO_4$) and concentrated *in vacuo*. The crude was purified by flash column chromatography (petrol / Et_2O (19:1)) to give iodide **4g** as a colourless solid (62 mg, 0.222 mmol, 35%); **R_f** 0.23 (petrol / EtOAc (4:1)); **mp** 91-93 ºC; **IR** (thin film, ν_{max} / cm^{-1}) 3339, 3056, 2921, 2850, 1571, 1463, 1356, 1288, 1181; **^{1}H NMR** (400 MHz, $CDCl_3$) δ_H 7.72 (1H, d, *J* = 14.8 Hz, ArCH=C*H*I), 7.16 (2H, t, *J* = 7.9 Hz, H5), 7.00 (2H, m, H4 and H6), 6.93 (1H, d, *J* = 14.8 Hz, ArC*H*=CHI), 5.66 (1H, br s, O*H*); **^{13}C NMR** (101 MHz, $CDCl_3$) δ_C 151.6, 141.0, 136.3, 127.8, 118.7, 118.1, 115.7, 80.5; **HRMS** (ES^-) calc. for C_8H_5ClIO [^{35}ClM-H]$^-$ 278.9079, found 278.9086.

1.7 Cross-Coupling of Cyclic Siloxanes

(1*E*,3*Z*)-1-Phenylundeca-1,3-dien-5-ol, 5aa

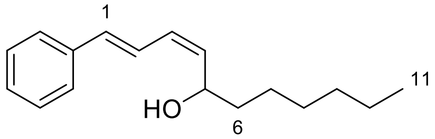

Procedure F: A degassed solution of TBAF•3H_2O (0.66 mL of a 1 M solution in THF, 0.660 mmol, 3.0 equiv.) was added to *E*-iodostyrene **4a** (51 mg, 0.221 mmol, 1.0 equiv.) oxasilole **1a** (50 mg, 0.221 mmol, 1.0 equiv.) and allylpalladium chloride dimer (2 mg, 0.005 mmol, 0.025 equiv.) at room temperature and stirred for 24 hours. The concentrated crude was purified by flash column chromatography (petrol / Et_2O (9:1→4:1) + 1% Et_3N) to give diene **5aa** as a yellow solid (39 mg, 0.159 mmol, 72%; isomerized to minor amounts of *E,E*-**5aa** on exposure to light).

Procedure G: A degassed solution of potassium trimethylsilanolate (73 mg of a 98 wt% solid in 1.32 mL of DME, 0.552 mmol, 2.5 equiv.) was added to silane **1a** (50 mg, 0.221 mmol, 1.0 equiv.), *E*-iodostyrene **4a** (51 mg, 0.221 mmol, 1.0 equiv.), water (40 μL, 2.21 mmol, 10 equiv.) and bis(dibenzylideneacetone)palladium (6 mg, 0.011 mmol, 0.05 equiv.) at room temperature and stirred at 60 °C for 24 hours. The concentrated crude was purified by flash column chromatography (petrol / Et_2O (9:1→17:3) + 1% Et_3N) to give alkene **5aa** as a yellow solid (35 mg, 0.143 mmol, 65%; isomerized to minor amounts of *E,E*-**5aa** on exposure to light); $\mathbf{R_f}$ 0.16 (petrol / Et_2O (19:1)); **mp** 50-51 ºC, **IR** (thin film, ν_{max} / cm^{-1}) 3352, 3080, 2955, 2927, 2856, 1637, 1494, 1465, 1449, 1378, 1030; **^{1}H NMR** (400 MHz, C_6D_6) δ_H 7.27 (2H, d, *J* = 7.8 Hz, *o*-Ar*H*), 7.17–7.09 (3H, m, *m*-Ar*H* and H2), 7.03 (1H, t, *J* = 7.8 Hz, *p*-Ar*H*), 6.43 (1H, d, *J* = 15.5 Hz, H1), 6.10 (1H, app t, *J* = 11.1 Hz, H3), 5.44 (1H, app t, *J* = 11.1 Hz, H4), 4.59-4.52 (1H, m, H5), 1.66-1.57 (1H, m, 1 × H6), 1.50-1.17 (9H, m, 1 × H6, H7, H8, H9 and H10), 1.11 (1H, s, O*H*), 0.85 (3H, t, *J* = 6.8 Hz, H11); **^{13}C NMR** (101 MHz, C_6D_6) δ_C 137.6, 135.6, 134.3, 129.9, 128.9, 128.3, 126.9, 124.4, 68.1, 38.1, 32.2, 29.7, 25.7, 23.0, 14.3; **HRMS** (ES^+) calc. for $C_{17}H_{24}NaO$ $[M+Na]^+$ 267.1725, found 267.1725.

Non-1-en-3-ol, 6

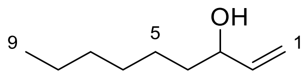

Isolated as a minor side product from the above reaction. **R_f** 0.31 (petrol / Et_2O (7:3)); **^{1}H NMR** (400 MHz, C_6D_6) δ_H 5.80-5.71 (1H, m, H2), 5.13 (1H, dt, *J* = 17.2 and 1.4 Hz, *trans* H1), 4.96 (1H, dt, *J* = 10.5 and 1.4 Hz, *cis* H1), 3.91-3.86 (1H, m, H3), 1.47-1.34 (3H, m, H4 and O*H*), 1.30-1.18 (8H, m, H5, H6, H7 and H8), 0.88 (3H, t, *J* = 7.0 Hz, H9); **^{13}C NMR** (101 MHz, C_6D_6) δ_C 141.9, 113.3, 72.6, 37.2, 31.9, 29.3, 25.3, 22.7, 14.0; **HRMS** (ES^+) calc. for $C_9H_{22}NO$ $[M+NH_4]^+$ 160.1696, found 160.1688.

All spectroscopic data were found to be in agreement with that reported by Bourland and co-workers.[31]

(8*Z*,10*Z*)-Octadeca-8,10-diene–7,12-diol, 7

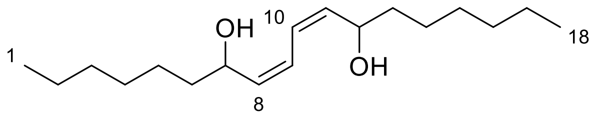

Isolated as a minor side product from the above reaction. **R_f** 0.24 (petrol / Et_2O (7:3)); **mp** 79-82 ºC; **IR** (thin film, ν_{max} / cm^{-1}) 3336, 2959, 2930, 2858, 1461, 1041, 1009; **^{1}H NMR** (400 MHz, C_6D_6) δ_H 6.41-6.33 (2H, m, H9 and H10), 5.58-5.49 (2H, m, H8 and H11), 4.57-4.51 (2H, m, H7 and H12), 1.74-1.64 (2H, m, 1 × diastereotopic H6 and 1 × diastereotopic H13), 1.58-1.30 (18H, m, H2, H3, H4, H5, 1 × diastereotopic H6, 1 × diastereotopic H13, H14, H15, H16 and H17), 1.09 (2H, br s, 2 × O*H*), 1.00 (6H, t, *J* = 7.2 Hz, H1 and H18); **^{13}C NMR** (101 MHz, C_6D_6) δ_C 136.5, 123.9, 67.5, 38.0, 32.2, 29.7, 25.6, 23.0, 14.3; **HRMS** (ES^+) calc. for $C_{18}H_{34}NaO_2$ $[M+Na]^+$ 305.2451, found 305.2450.

(*Z*)-1-Phenylnon-1-en-3-ol, 5ba

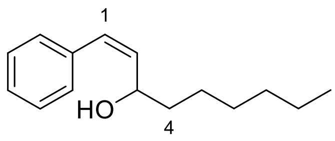

Procedure F: A solution of TBAF•3H_2O (0.32 mL of a 1 M solution in THF, 0.320 mmol, 3.0 equiv.) was added to iodobenzene (12 µL, 0.106 mmol, 1.0 equiv.), silane **1a** (24 mg, 0.106 mmol, 1.0 equiv.) and allylpalladium chloride dimer (1 mg, 0.003 mmol, 0.025 equiv.) at room temperature and the mixture was stirred for 24 hours. The concentrated crude was purified by flash column chromatography (petrol / Et_2O (19:1→9:1) + 1% Et_3N) to give alkene **5ba** as a yellow oil (18 mg, 0.084 mmol, 80%).

Procedure G: A degassed solution of potassium trimethylsilanolate (73 mg of a 98 wt% solid in 1.32 mL of DME, 0.552 mmol, 2.5 equiv.) was added to silane **1a** (50 mg, 0.221 mmol, 1.0 equiv.), iodobenzene (25 µL, 0.221 mmol, 1.0 equiv.), water (40 µL, 2.21 mmol, 10 equiv.) and bis(dibenzylideneacetone)palladium (6 mg, 0.011 mmol, 0.05 equiv.) at room temperature and stirred at 60 °C for 24 hours. The concentrated crude was purified by flash column chromatography (petrol / Et_2O (9:1→17:3) + 1% Et_3N) to give alkene **5ba** as a yellow oil (42 mg, 0.192 mmol, 87%); **R_f** 0.20 (petrol / Et_2O (4:1)); **IR** (thin film, ν_{max} / cm^{-1}) 3332, 3010, 2955, 2927, 2856, 1736, 1494, 1459, 1377, 1232, 1040, 1012; **^{1}H NMR** (400 MHz, $CDCl_3$) δ_H 7.38–7.25 (5H, m, Ar*H*), 6.57 (1H, d, *J* = 11.5 Hz, H1), 5.68 (1H, dd, *J* = 11.5 and 9.2 Hz, H2), 4.61-4.55 (1H, m, H3), 1.71-1.53 (2H, m, H4), 1.43-1.24 (8H, m, H5, H6, H7, H8 and O*H*), 0.88 (3H, t, *J* = 6.8 Hz, H9); **^{13}C NMR** (101 MHz, $CDCl_3$) δ_C 136.7, 134.7, 131.0, 128.7, 128.3, 127.2, 67.9, 37.6, 31.8, 29.2, 25.3, 22.6, 14.1; **HRMS** (ES^+) calc. for $C_{15}H_{22}NaO$ $[M+Na]^+$ 241.1568, found 241.1567.

(*Z*)-1-Cyclohexyl-3-phenylprop-2-en-1-ol, 5bd

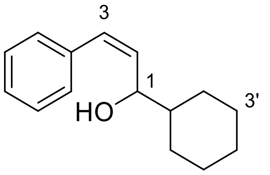

Procedure F: A solution of TBAF•3H_2O (0.64 mL of a 1 M solution in THF, 0.640 mmol, 3.0 equiv.) was added to iodobenzene (24 μL, 0.214 mmol, 1.0 equiv.), silane **1d** (48 mg, 0.214 mmol, 1.0 equiv.) and allylpalladium chloride dimer (2 mg, 0.005 mmol, 0.025 equiv.) at room temperature and the mixture was stirred for 24 hours. The concentrated crude was purified by flash column chromatography (petrol / Et_2O (9:1) + 1% Et_3N) to give alkene **5bd** as a colourless solid (42 mg, 0.196 mmol, 92%).

Procedure G: A degassed solution of potassium trimethylsilanolate (73 mg of a 98 wt% solid in 1.32 mL of DME, 0.552 mmol, 2.5 equiv.) was added to silane **1d** (50 mg, 0.221 mmol, 1.0 equiv.), iodobenzene (25 μL, 0.221 mmol, 1.0 equiv.), water (40 μL, 2.21 mmol, 10 equiv.) and bis(dibenzylideneacetone)palladium (6 mg, 0.011 mmol, 0.05 equiv.) at room temperature and stirred at 60 °C for 24 hours. The concentrated crude was purified by flash column chromatography (petrol / Et_2O (9:1→17:3) + 1% Et_3N) to give alkene **5bd** as a colourless solid (38 mg, 0.176 mmol, 80%); **R$_f$** 0.31 (petrol / Et_2O (2:1)); **mp** 77–78 ºC; **IR** (thin film, ν_{max} / cm^{-1}) 3393, 3023, 2920, 2849, 1494, 1306, 1239, 1209, 1140, 1094, 1081, 1005; **^{1}H NMR** (400 MHz, C_6D_6) δ_H 7.32 (2H, d, *J* = 7.5 Hz, *o*-ArH), 7.15 (2H, t, *J* = 7.5 Hz, *m*-ArH), 7.04 (1H, t, *J* = 7.5 Hz, *p*-ArH), 6.46 (1H, d, *J* = 11.8 Hz, H3), 5.55 (1H, dd, *J* = 11.8 and 9.6 Hz, H2), 4.24 (1H, dd, *J* = 9.6 and 7.2 Hz, H1), 1.97-1.91 (1H, m, O*H*), 1.68-1.54 (4H, m, H2'), 1.35-1.26 (1H, m, H1'), 1.16-0.86 (6H, m, H3' and H4'); **^{13}C NMR** (101 MHz, C_6D_6) δ_C 137.4, 134.3, 131.6, 129.2, 128.5, 127.3, 71.8, 44.7, 29.2, 28.7, 26.8, 26.5, 26.4; **HRMS** (ES^+) calc. for $C_{15}H_{20}NaO$ $[M+Na]^+$ 239.1412, found 239.1416.

(*Z*)-1,3-Diphenylprop-2-en-1-ol, 5bg

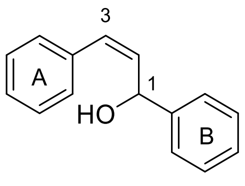

Procedure F: A solution of TBAF•3H_2O (0.69 mL of a 1 M solution in THF, 0.690 mmol, 3.0 equiv.) was added to iodobenzene (26 µL, 0.230 mmol, 1.0 equiv.), silane **1g** (50 mg, 0.230 mmol, 1.0 equiv.) and allylpalladium chloride dimer (2 mg, 0.006 mmol, 0.025 equiv.) at room temperature and the mixture was stirred for a further 24 hours. The concentrated crude was purified by flash column chromatography (petrol / Et_2O (9:1→3:1) + 1% Et_3N) to give alkene **5bg** as a colourless oil (42 mg, 0.200 mmol, 87%).

Procedure G: A degassed solution of potassium trimethylsilanolate (73 mg of a 98 wt% solid in 1.32 mL of DME, 0.552 mmol, 2.5 equiv.) was added to silane **1g** (48 mg, 0.221 mmol, 1.0 equiv.), iodobenzene (25 µL, 0.221 mmol, 1.0 equiv.), water (40 µL, 2.21 mmol, 10 equiv.) and bis(dibenzylideneacetone)palladium (6 mg, 0.011 mmol, 0.05 equiv.) at room temperature and stirred at 60 °C for 24 hours. The concentrated crude was purified by flash column chromatography (petrol / Et_2O (9:1→17:3) + 1% Et_3N) to give alkene **5bg** as a colourless oil (41 mg, 0.195 mmol, 88%); $\mathbf{R_f}$ 0.26 (petrol / Et_2O (2:1)); **^{1}H NMR** (500 MHz, C_6D_6) δ_H 7.47 (2H, d, *J* = 7.6 Hz, *o*-Ar^{B}H), 7.39 (2H, d, *J* = 7.6 Hz, *o*-Ar^{A}H), 7.28–7.15 (6H, m, *m*-Ar^{A}H, *p*-Ar^{A}H, *m*-Ar^{B}H and *p*-Ar^{B}H), 6.55 (1H, d, *J* = 11.6 Hz, H3), 5.91 (1H, dd, *J* = 11.6 and 9.4 Hz, H2), 5.68 (1H, d, *J* = 9.4 Hz, H1), 1.84 (1H, br, O*H*); **^{13}C NMR** (125 MHz, C_6D_6) δ_C 144.1, 137.0, 134.4, 130.9, 129.2, 128.7, 128.6, 127.7, 127.6, 126.6, 70.0; **LRMS** (ES$^+$) calc. for $C_{15}H_{14}NaO$ $[M+Na]^+$ 233.1, found 233.1.

The spectroscopic data were found to be in agreement with that reported by Banert and co-workers.[32]

(2*E*,4*Z*)-1-(Benzyloxy)dodeca-2,4-dien-6-ol, 5ca

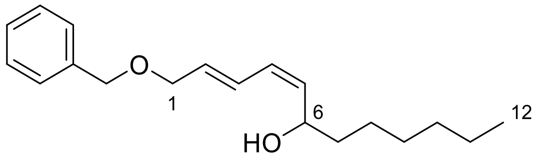

Procedure F: A solution of TBAF•3H_2O (0.53 mL of a 1 M solution in THF, 0.530 mmol, 3.0 equiv.) was added to iodide **4c** (48 mg, 0.177 mmol, 1.0 equiv.), oxasilole **1a** (40 mg, 0.177 mmol, 1.0 equiv.) and allylpalladium chloride dimer (2 mg, 0.004 mmol, 0.025 equiv.) at room temperature and stirred for 24 hours. The concentrated crude was purified by flash column chromatography (petrol / Et_2O (9:1→17:3) + 1% Et_3N) to give diene **5ca** as a pale yellow oil (35 mg, 0.128 mmol, 72%; isomerized to minor amounts of *E,E*-**5ca** on exposure to light); **R$_f$** 0.18 (petrol / Et_2O (4:1)); **IR** (thin film, ν_{max} / cm^{-1}) 3400, 2957, 2929, 2855, 1496, 1359, 1114, 1095, 1065, 1028; **^{1}H NMR** (500 MHz, C_6D_6) δ_H 7.29 (2H, d, *J* = 7.7 Hz, *o*-Ar*H*), 7.17 (1H, t, *J* = 7.7 Hz, *m*-Ar*H*), 7.08 (1H, t, *J* = 7.7 Hz, *p*-Ar*H*), 6.64 (1H, dd, *J* = 14.8 and 11.4 Hz, H3), 5.95 (1H, t, *J* = 11.4 Hz, H4), 5.68 (1H, dt, *J* = 14.8 and 5.6 Hz, H2), 5.35 (1H, app t, *J* = 11.4 Hz, H5), 4.47-4.37 (1H, m, H6), 4.33 (2H, s, ArC*H*$_2$O), 3.86 (2H, d, *J* = 5.6 Hz, H1), 1.59-1.52 (1H, m, 1 × H7), 1.43-1.12 (10H, m, 1 × H7, H8, H9, H10, H11 and O*H*), 0.75 (3H, t, *J* = 7.0 Hz, H12); **^{13}C NMR** (125 MHz, C_6D_6) δ_C 139.0, 135.3, 131.9, 128.9, 128.6, 128.3, 127.7, 127.2, 72.4, 70.3, 67.9, 38.1, 32.2, 29.7, 25.7, 23.0, 14.3; **HRMS** (ES^+) calc. for $C_{19}H_{28}NaO_2$ $[M+Na]^+$ 311.1982, found 311.1970.

(2*Z*,4*E*)-1-Phenylnona-2,4-dien-1-ol, 5dg

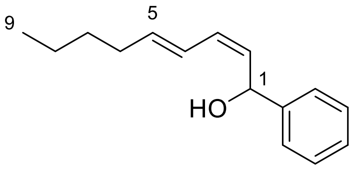

Procedure F: A solution of TBAF•3H_2O (0.47 mL of a 1 M solution in THF, 0.470 mmol, 3.0 equiv.) was added to (*E*)-1-iodohex-1-ene (49 mg, 0.234 mmol, 1.5 equiv.), silane **1g** (34 mg, 0.156 mmol, 1.0 equiv.) and allylpalladium chloride dimer (1.4 mg, 0.004 mmol, 0.025 equiv.) at room temperature and the mixture was stirred for 24 hours. The concentrated crude was purified by flash column chromatography (petrol / Et_2O (19:1→9:1) + 1% Et_3N) to give alkene **5dg** as a colourless oil (19.4 mg, 0.090 mmol, 57%); R_f 0.40 (petrol / Et_2O (2:1)); **IR** (thin film, ν_{max} / cm^{-1}) 3338, 2957, 2926, 2872, 1652, 1493, 1452, 1024; ^{1}**H NMR** (500 MHz, C_6D_6) δ_H 7.41 (2H, d, J = 7.7 Hz, *o*-ArH), 7.17 (2H, t, J = 7.7 Hz, *m*-ArH), 7.07 (1H, t, J = 7.7 Hz, *p*-ArH), 6.47 (1H, dd, J = 14.8 and 11.4 Hz, H4), 5.99 (1H, app t, J = 11.4 Hz, H3), 5.60 (1H, dt, J = 14.8 and 7.2 Hz, H5), 5.55 (1H, d, J = 9.3 Hz, H1), 5.45 (1H, m, H2), 1.98 (2H, q, J = 7.2 Hz, H6), 1.36-1.18 (5H, m, H7, H8 and O*H*), 0.84 (3H, t, J = 7.0 Hz, H9); ^{13}**C NMR** (125 MHz, C_6D_6) δ_C 144.6, 137.8, 132.1, 130.4, 128.9, 127.7, 126.5, 126.0, 70.2, 33.1, 31.9, 22.9, 14.4; **HRMS** (ES^+) calc. for $C_{15}H_{20}NaO$ $[M+Na]^+$ 239.1412, found 239.1408.

(4*Z*,6*E*)-1-((*Tert*-butyldimethylsilyl)oxy)–7-phenylhepta-4,6-dien-3-ol, 5ae

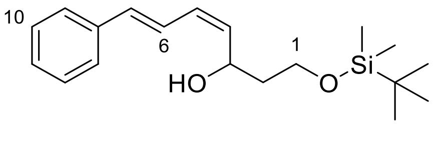

Procedure G: A degassed solution of potassium trimethylsilanolate (73 mg of a 98 wt% solid in 1.32 mL of DME, 0.552 mmol, 2.5 equiv.) was added to silane **1e** (50 mg, 0.221 mmol, 1.0 equiv.), iodide **4a** (76 mg, 0.332 mmol, 1.5 equiv.), water (40 µL, 2.21 mmol, 10 equiv.) and bis(dibenzylideneacetone)palladium (6 mg, 0.011

mmol, 0.05 equiv.) at room temperature and stirred at 60 °C for 24 hours. The concentrated crude was purified by flash column chromatography (petrol / Et_2O (9:1→17:3) + 1% Et_3N) to give alkene **5ae** as a pale yellow oil (39 mg, 0.128 mmol, 58%); **R$_f$** 0.15 (petrol / Et_2O (2:1)); **IR** (thin film, ν_{max} / cm^{-1}) 3417, 2954, 2929, 2857, 1471, 1256, 1086; **^{1}H NMR** (500 MHz, C_6D_6) δ_H 7.29 (2H, d, *J* = 7.3 Hz, H9), 7.23 (1H, dd, *J* = 15.5 and11.2 Hz, H6), 7.11 (2H, t, *J* = 7.3 Hz, H10), 7.01 (1H, t, *J* = 7.3 Hz, H11), 6.41 (1H, d, *J* = 15.5 Hz, H7), 6.09 (1H, t, *J* = 11.2 Hz, H5), 5.54 (1H, dd, *J* = 11.2 and 8.7 Hz, H4), 4.96-4.92 (1H, m, H3), 3.71-3.67 and 3.60-3.55 (2 × 1H, m, diastereotopic H1), 2.28 (1H, br, O*H*), 1.81-1.74 and 1.64-1.58 (2 × 1H, m, diastereotopic H2), 0.92 (9H, s, SiC(C*H*$_3$)$_3$), 0.00 and -0.02 (2 × 3H, s, diastereotopic Si(C*H*$_3$)$_2$); **^{13}C NMR** (125 MHz, C_6D_6) δ_C 138.0, 135.6, 134.5, 129.8, 129.2, 128.2, 127.2, 124.8, 67.2, 61.3, 40.5, 26.4, 18.6, -5.1, -5.2; **HRMS** (ES^+) calc. for $C_{19}H_{30}NaO_2Si$ $[M+Na]^+$ 341.1913, found 341.1900.

(*Z*)-5-((*Tert*-butyldimethylsilyl)oxy)-1-phenylpent-1-en-3-ol, 5be

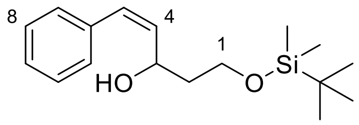

Procedure G: A degassed solution of potassium trimethylsilanolate (73 mg of a 98 wt% solid in 1.32 mL of DME, 0.552 mmol, 2.5 equiv.) was added to silane **1e** (66 mg, 0.221 mmol, 1.0 equiv.), iodobenzene (25 μL, 0.221 mmol, 1.0 equiv.), water (40 μL, 2.21 mmol, 10 equiv.) and bis(dibenzylideneacetone)palladium (6 mg, 0.011 mmol, 0.05 equiv.) at room temperature and stirred at 60 °C for 24 hours. The concentrated crude was purified by flash column chromatography (petrol / Et_2O (9:1→17:3) + 1% Et_3N) to give alkene **5be** as a pale yellow oil (46 mg, 0.165 mmol, 75%); **R$_f$** 0.33 (petrol / Et_2O (2:1)); **IR** (thin film, ν_{max} / cm^{-1}) 3429, 2954, 2929, 2857, 1471, 1255, 1085, 1006; **^{1}H NMR** (500 MHz, C_6D_6) δ_H 7.39 (2H, d, *J* = 7.5 Hz, H7), 7.21 (2H, t, *J* = 7.5 Hz, H8), 7.08 (1H, t, *J* = 7.5 Hz, H9), 6.41 (1H, d, *J* = 11.7 Hz, H5), 5.72 (1H, dd, *J* = 11.7 and 9.1 Hz, H4), 4.93 (1H, dt, *J* = 9.1 and 3.4 Hz, H3), 3.74-3.70 and 3.59-3.55 (2 × 1H, m, diastereotopic H1), 2.38 (1H, br, O*H*), 1.81-1.75 and 1.71-1.65 (2 × 1H, m, diastereotopic H2), 0.93 (9H, s, SiC(C*H*$_3$)$_3$), 0.01 and 0.00

(2 × 3H, s, diastereotopic Si(CH_3)$_2$); **^{13}C NMR** (125 MHz, C_6D_6) δ_C 137.6, 135.9, 130.4, 129.6, 128.9, 127.6, 66.7, 61.3, 40.1, 26.3, 18.6, -5.2; **HRMS** (ES$^+$) calc. for $C_{17}H_{28}NaO_2Si$ [M+Na]$^+$ 315.1756, found 315.1745.

((2*R*,3*S*,4*Z*,6*E*)-2-Methyl–7-phenylhepta-4,6-diene-1,2,3-triol, 5ah

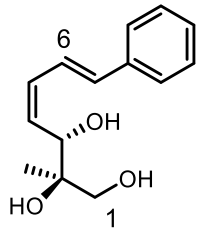

Procedure F: A solution of TBAF•3H_2O (0.27 mL of a 1 M solution in THF, 0.270 mmol, 3.0 equiv.) was added to *E*-iodostyrene **4a** (21 mg, 0.09 mmol, 1.0 equiv.), silane **1h** (20 mg, 0.09 mmol, 1.0 equiv.) and allylpalladium chloride dimer (1 mg, 0.0027 mmol, 0.025 equiv.) at room temperature and the mixture was stirred for 24 hours. The concentrated crude was purified by flash column chromatography (petrol / EtOAc (1:4)) to give title compound **5ah** (17 mg, 0.38 mmol, 81%); **R_f** 0.28 (petrol / EtOAc (1:4)); $[\alpha]_D^{20}$ +45.7 (*c* 0.73, $CHCl_3$); **IR** (thin film, ν_{max} / cm^{-1}) 3368, 2927, 1450, 1043, 1002; **^{1}H NMR** (400 MHz, C_6D_6) δ_H 7.42−6.98 (5H, m, Ar*H*), 7.23 (1H, dd, *J* = 15.5 and 11.0 Hz, H6), 6.41 (1H, d, *J* = 15.5 Hz, H7), 6.18 (1H, t, *J* = 11.0 Hz, H5), 5.57 (1H, t, *J* = 11.0 Hz, H4), 4.82 (1H, d, *J* = 11.0 Hz, H3), 3.94 (1H, bs, O*H*), 3.87 (1H, d, *J* = 11.5 Hz, 1 × H1), 3.77 (1H, br s, O*H*), 3.59 (1H, br s, O*H*), 3.52 (1H, d, *J* = 11.5 Hz, 1 × H1), 1.14 (3H, s, CCH_3); **^{13}C NMR** (100 MHz, $CDCl_3$) δ_C 137.1, 135.0, 132.2, 129.8, 128.6, 127.8, 126.7, 123.9, 74.3, 73.9, 67.5, 20.8; **HRMS** (ESI+) calc. for $C_{14}H_{18}O_3$ [M+Na]$^+$ 257.1148 found 257.1151.

(5*Z*,7*E*)-1-((4-Methoxybenzyl)oxy)-8-phenylocta-5,7-dien-3-ol, 5ao

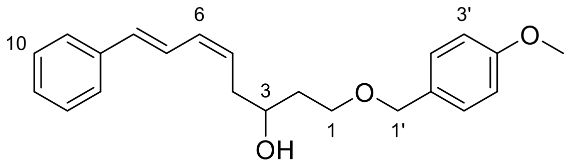

Procedure F: A solution of TBAF•3H_2O (0.46 mL of a 1 M solution in THF, 0.460 mmol, 3.0 equiv.) was added to *E*-iodostyrene **4a** (35 mg, 0.153 mmol, 1.0 equiv.), silane **1o** (49 mg, 0.153 mmol, 1.0 equiv.) and allylpalladium chloride dimer (1.4 mg, 0.004 mmol, 0.025 equiv.) at room temperature and the mixture was stirred for 48 hours. The concentrated crude was purified by flash column chromatography (petrol / EtOAc (4:1) + 1% Et_3N) to give alkene **5ao** as a yellow oil (37 mg, 0.109 mmol, 71%); **R$_f$** 0.24 (petrol / EtOAc (4:1)); **IR** (thin film, ν_{max} / cm^{-1}) 3458, 3026, 2934, 2861, 1612, 1513, 1248, 1089; **^{1}H NMR** (400 MHz, C_6D_6) δ_H 7.39 (2H, d, *J* = 7.5 Hz, H9), 7.30–7.21 (5H, m, H7, H10 and H2'), 7.14 (1H, t, *J* = 7.5 Hz, H11), 6.88 (2H, d, *J* = 8.4 Hz, H3'), 6.57 (1H, d, *J* = 15.5 Hz, H8), 6.36 (1H, t, *J* = 11.0 Hz, H6), 5.75-5.68 (1H, m, H5), 4.32 (2H, s, H1'), 4.01-3.94 (1H, m, H3), 3.59-3.54 and 3.51-3.45 (2 × 1H, m, diastereotopic H1), 3.39 (3H, s, OC*H*$_3$), 2.89 (1H, br s, O*H*), 2.65-2.58 and 2.56-2.49 (2 × 1H, m, diastereotopic H4), 1.86-1.77 and 1.74-1.67 (2 × 1H, m, diastereotopic H2); **^{13}C NMR** (101 MHz, C_6D_6) δ_C 159.8, 138.0, 133.3, 131.2, 130.6, 129.4, 129.0, 128.9, 127.7, 126.8, 124.7, 114.1, 73.1, 71.1, 68.8, 54.8, 36.5, 36.5; **HRMS** (ES$^+$) calc. for $C_{22}H_{26}NaO_3$ [M+Na]$^+$ 361.1774, found 361.1764.

(*Z*)-1-((4-Methoxybenzyl)oxy)-6-phenylhex-5-en-3-ol, 5bo

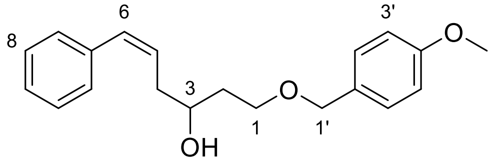

Procedure F: A solution of TBAF•3H_2O (0.47 mL of a 1 M solution in THF, 0.470 mmol, 3.0 equiv.) was added to iodobenzene (32 mg, 0.156 mmol, 1.0 eq, silane **1o**

(50 mg, 0.156 mmol, 1.0 equiv.) and allylpalladium chloride dimer (1.5 mg, 0.004 mmol, 0.025 equiv.) at room temperature and the mixture was stirred for 48 hours. The concentrated crude was purified by flash column chromatography (petrol / EtOAc (17:3) + 1% Et_3N) to give alkene **5bo** as a colourless oil (46 mg, 0.147 mmol, 94%); **R_f** 0.18 (petrol / EtOAc (4:1)); **IR** (thin film, ν_{max} / cm^{-1}) 3435, 3012, 2935, 2861, 1612, 1513, 1248, 1087, 1034; **^{1}H NMR** (400 MHz, C_6D_6) δ_H 7.41 (2H, d, *J* = 7.6 Hz, H7), 7.29–7.22 (4H, m, H8 and H2'), 7.16 (1H, t, *J* = 7.6 Hz, H9), 6.87 (2H, d, *J* = 8.6 Hz, H3'), 6.62 (1H, d, *J* = 12.3 Hz, H6), 5.93 (1H, dt, *J* = 12.3 and 7.3 Hz, H5), 4.16 and 4.15 (2 × 1H, d, *J* = 11.9 Hz, diastereotopic H1'), 3.98-3.92 (1H, m, H3), 3.52-3.47 and 3.44-3.40 (2 × 1H, m, diastereotopic H1), 3.40 (3H, s, OC*H*$_3$), 2.93 (1H, br s, O*H*), 2.71-2.63 and 2.61-2.54 (2 × 1H, m, diastereotopic H4), 1.77-1.68 and 1.62-1.55 (2 × 1H, m, diastereotopic H2); **^{13}C NMR** (101 MHz, C_6D_6) δ_C 159.8, 138.0, 130.9, 130.6, 129.4, 129.3, 129.2, 128.6, 126.9, 114.1, 73.0, 71.1, 68.7, 54.8, 37.0, 36.6; **HRMS** (ES^+) calc. for $C_{20}H_{24}NaO_3$ $[M+Na]^+$ 335.1618, found 335.1618.

(*Z*)-5-((4-Methoxybenzyl)oxy)-1-phenylpent-1-en-3-ol, 5bf

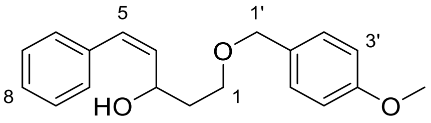

Procedure G: A degassed solution of potassium trimethylsilanolate (54 mg of a 98 wt% solid in 0.98 mL of DME, 0.408 mmol, 2.5 equiv.) was added to silane **1f** (50 mg, 0.163 mmol, 1.0 equiv.), silane **1o** (52 mg, 0.163 mmol, 1.0 equiv.), iodobenzene (18 µL, 0.163 mmol, 1.0 equiv.), and bis(dibenzylideneacetone)palladium (5 mg, 0.008 mmol, 0.05 equiv.) at room temperature and stirred at 60 °C for 24 hours. The concentrated crude was purified by flash column chromatography (petrol / EtOAc (19:1→3:1)) to give silane **1o** (45 mg, 0.140 mmol, 86% recovered) and alkene **5bf** as a pale yellow oil (44 mg, 0.147 mmol, 90%); **R_f** 0.17 (petrol / EtOAc (7:3)); **IR** (thin film, ν_{max} / cm^{-1}) 3413, 2937, 2913, 2861, 1613, 1513, 1463, 1362, 1302, 1247, 1174, 1090, 1033; **^{1}H NMR** (400 MHz, C_6D_6) δ_H 7.39 (2H, d, *J* = 8.7 Hz, H2'), 7.19–7.13 (4H, m, H6 and H7), 7.06 (1H, t, *J* = 7.4 Hz, H8), 6.79 (2H, d, *J* = 8.7 Hz, H3'), 6.42

(1H, d, J = 11.7 Hz, H5), 5.72 (1H, dd, J = 11.7 and 9.2 Hz, H4), 4.97-4.91 (1H, m, H3), 4.24-4.17 (2H, m, H1'), 3.53-3.46 and 3.40-3.35 (2 × 1H, m, diastereotopic H1), 3.33 (3H, s, OCH_3), 2.55 (1H, br s, OH), 1.95-1.87 and 1.79-1.71 (2 × 1H, m, diastereotopic H2); **^{13}C NMR** (101 MHz, C_6D_6) δ_C 159.4, 137.0, 135.0, 130.5, 130.0, 129.0, 129.0, 128.2, 127.0, 113.8, 72.6, 67.6, 66.5, 54.4, 37.6; **HRMS** (ES^+) calc. for $C_{19}H_{22}NaO_3$ $[M+Na]^+$ 321.1461, found 321.1460.

(4*Z*,6*E*)-1-((4-Methoxybenzyl)oxy)–7-phenylhepta-4,6-dien-3-ol, 5af

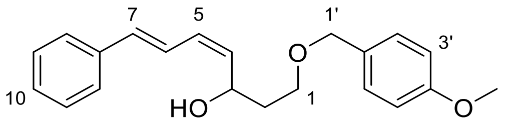

Procedure G: A degassed solution of potassium trimethylsilanolate was added portionwise at 0 h, 2 h and 6 h (3 × 75 µL of a 1M solution in DME, 3 × 0.075 mmol, 3 × 1.0 equiv.) to silane **1f** (23 mg, 0.075 mmol, 1.0 equiv.), silane **1o** (24 mg, 0.075 mmol, 1.0 equiv.), iodide **4a** (17 mg, 0.075 mmol, 1.0 equiv.), and bis(dibenzylideneacetone)palladium (4 mg, 0.008 mmol, 0.10 equiv.) at room temperature and stirred for 24 hours. The concentrated crude was purified by flash column chromatography (petrol / EtOAc (100:0→4:1) + 1% Et_3N) to give silane **1o** (19 mg, 0.059 mmol, 79% recovered) and alkene **5af** as a yellow oil (17 mg, 0.052 mmol, 70%); **R$_f$** 0.25 (petrol / EtOAc (7:3)); **IR** (thin film, ν_{max} / cm^{-1}) 3407, 3035, 2934, 2861, 2839, 1613, 1513, 1248, 1095, 1034; **^{1}H NMR** (500 MHz, C_6D_6) δ_H 7.28–7.22 (3H, m, H6 and H2'), 7.19–7.15 (2H, m, H8), 7.11 (2H, t, J = 7.7 Hz, H9), 7.03 (1H, t, J = 7.7 Hz, H10), 6.77 (2H, d, J = 8.4 Hz, H3'), 6.44 (1H, d, J = 15.6 Hz, H7), 6.13 (1H, t, J = 11.0 Hz, H5), 5.53 (1H, dd, J = 11.0 and 8.4 Hz, H4), 5.01-4.96 (1H, m, H3), 4.25 (2H, s, H1'), 3.51-3.47 and 3.41-3.36 (2 × 1H, m, diastereotopic H1), 3.31 (3H, s, OCH_3), 2.34 (1H, br s, OH), 2.00-1.93 and 1.72-1.66 (2 × 1H, m, diastereotopic H2); **^{13}C NMR** (125 MHz, C_6D_6) δ_C 160.0, 138.0, 135.4, 134.5, 131.1, 130.1, 129.7, 129.2, 128.6, 127.2, 124.9, 114.4, 73.3, 68.0, 67.3, 55.0, 38.1; **HRMS** (ES^+) calc. for $C_{21}H_{24}NaO_3$ $[M+Na]^+$ 347.1618, found 347.1621.

(2*E*,4*Z*,6*Z*)-10-((4-Methoxybenzyl)oxy)deca-2,4,6-triene-1,8-diol, 8

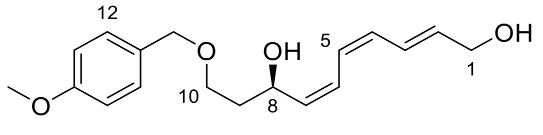

Procedure F (modified)**:** TBAF•3H_2O (0.49 mL of a 1 M solution in THF, 0.490 mmol, 3.0 equiv.) was added to silane (*R*)-**1f** (50 mg, 0.163 mmol, 1.0 equiv.), iodide **4e** (45 mg, 0.212 mmol, 1.3 equiv.), and allylpalladium chloride dimer (3 mg, 0.008 mmol, 0.05 equiv.) and stirred at room temperature in the dark for 48 hours. The reaction mixture was diluted with EtOAc, washed with water three times and dried (Na_2SO_4). The concentrated crude, which was kept in the dark and not heated above 20 ºC, was purified by flash column chromatography (CH_2Cl_2 / methanol (100:0→99.75:0.25) + 1% Et_3N) to give triene **8** as a pale yellow oil (34 mg, 0.107 mmol, 66%); $[\alpha]_D^{20}$ –70.1° (*c* 0.5, $CHCl_3$) for 97% ee; $\mathbf{R_f}$ 0.16 (petrol / EtOAc / methanol (49:50:1)); **IR** (thin film, ν_{max} / cm^{-1}) 3375, 2920, 2853, 1612, 1512, 1246, 1088; **^{1}H NMR** (400 MHz, $CDCl_3$) δ_H 7.26 (2H, d, *J* = 8.5 Hz, H12), 6.89 (2H, d, *J* = 8.5 Hz, H13), 6.73 (1H, dd, *J* = 15.0 and 11.3 Hz, H3), 6.50 (1H, t, *J* = 11.3 Hz, H6), 6.27 (1H, t, *J* = 11.3 Hz, H5), 6.06 (1H, t, *J* = 11.3 Hz, H4), 5.91 (1H, dt, *J* = 15.0 and 5.7 Hz, H2), 5.56-5.50 (1H, m, H7), 4.89-4.81 (1H, m, H8), 4.44 (2H, s, H11), 4.24 (2H, d, *J* = 5.7 Hz, H1), 3.81 (3H, s, OC*H*$_3$), 3.70-3.65 and 3.61-3.56 (2 × 1H, m, diastereotopic H10), 2.80 (1H, br s, O*H*), 1.97-1.88 and 1.80-1.73 (2 × 1H, m, diastereotopic H9), 1.64 (1H, br s, O*H*); **^{13}C NMR** (101 MHz, $CDCl_3$) δ_C 159.4, 134.5, 134.1, 130.0, 129.9, 129.4, 125.8, 124.2, 124.1, 113.8, 73.0, 67.9, 67.0, 63.3, 55.3, 36.8; **HRMS** (ES^+) calc. for $C_{18}H_{24}NaO_4$ $[M+Na]^+$ 327.1567, found 327.1563.

(4*Z*,6*Z*)–7-Cyclohexyl-1-((4-methoxybenzyl)oxy)hepta-4,6-dien-3-ol, 9

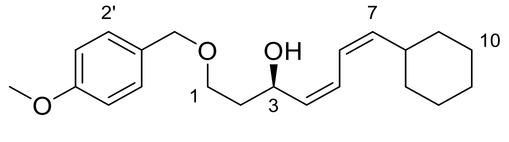

Procedure G: TBAF•$3H_2O$ (0.50 mL of a 1 M solution in THF, 0.500 mmol, 3.0 equiv.) was added to silane (*R*)**-1f** (50 mg, 0.163 mmol, 1.0 equiv.), iodide **4f** (77 mg, 0.326 mmol, 2.0 equiv.), water (29 µL, 1.63 mmol, 10 equiv.) and allylpalladium chloride dimer (3 mg, 0.008 mmol, 0.05 equiv.) and the mixture stirred at 50 °C in the dark for a 24 hours. The concentrated crude was purified by flash column chromatography (petrol / EtOAc (100:0→9:1) + 1% Et_3N) to give diene **9** as a pale yellow oil (28 mg, 0.085 mmol, 52%; isomerized to minor amounts of *E,Z*- and *E,E*-**9** on exposure to light); $\mathbf{R_f}$ 0.26 (petrol / EtOAc (4:1)); $[\alpha]_D^{25}$ -43.6° (*c* 1.0, $CHCl_3$) for 97% ee; **IR** (thin film, ν_{max} / cm^{-1}) 3415, 2924, 2851, 1613, 1513, 1248, 1094, 1036; **^{1}H NMR** (500 MHz, C_6D_6) δ_H 7.17 (2H, d, *J* = 8.8 Hz, H2'), 6.77 (2H, d, *J* = 8.8 Hz, H3'), 6.34-6.26 (2H, m, H5 and H6), 5.55-5.51 (1H, m, H4), 5.34-5.30 (1H, m, H7), 4.90-4.86 (1H, m, H3), 4.24 (2H, s, H1'), 3.49-3.45 and 3.40-3.35 (2 × 1H, m, diastereotopic H1), 3.29 (3H, s, OCH_3), 2.46-2.31 (2H, m, H8 and O*H*), 1.94-1.87 and 1.72-1.68 (2 × 1H, m, diastereotopic H2), 1.67-1.53 (4H, m, H10), 1.23-1.15 (2H, m, H11), 1.12-0.97 (4H, m, H9); **^{13}C NMR** (125 MHz, C_6D_6) δ_C 159.8, 139.5, 134.8, 129.5, 128.3, 124.4, 122.2, 114.1, 73.0, 67.9, 66.8, 54.8, 37.8, 36.8, 33.5, 26.3, 26.1; **HRMS** (ES^+) calc. for $C_{21}H_{30}NaO_3$ $[M+Na]^+$ 353.2087, found 353.2076.

2-Chloro-3-((1*E*,3*Z*)-5-hydroxy–7-((4-methoxybenzyl)oxy)hepta-1,3-dien-1-yl)phenol, 10

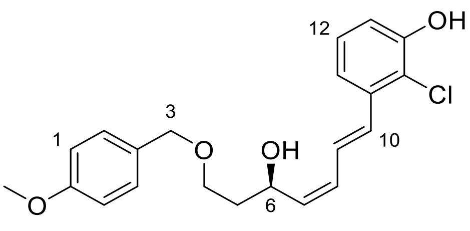

Procedure F (modified)**:** TBAF•3H_2O (0.39 mL of a 1 M solution in THF, 0.390 mmol, 3.0 equiv.) was added to silane (*R*)**-1f** (40 mg, 0.129 mmol, 1.0 equiv.), *E*-iodide **4g** (47 mg, 0.214 mmol, 1.3 equiv.) and allylpalladium chloride dimer (2 mg, 0.006 mmol, 0.05 equiv.) and the mixture stirred at 50 °C in the dark for a 24 hours. The mixture was filtered through a plug of SiO_2 eluting with EtOAc, then CH_2Cl_2, diluted with Et_2O, washed 5 times with water and dried (Na_2SO_4). The concentrated crude was purified by flash column chromatography (CH_2Cl_2 / MeOH (100:0→99.6:0.4) + 1% Et_3N) to give diene **10** as a pale yellow oil (33 mg, 97 wt% with Et_3N, 0.085 mmol, 66%, 98:2 *Z*:*E*); $[\alpha]_D^{20}$ –76.8° (*c* 0.5, $CHCl_3$) for 97% ee; $\mathbf{R_f}$ 0.09 (petrol / EtOAc (1:1)); **IR** (thin film, ν_{max} / cm^{-1}) 3374, 2924, 2862, 1612, 1513, 1465, 1245, 1035; **^{1}H NMR** (400 MHz, C_6D_6) δ_H 7.23–7.12 (m, 3H, H2 and H9), 6.96 (1H, d, *J* = 15.5 Hz, H10), 6.94 (1H, dd, *J* = 7.6 and 1.4 Hz, H11), 6.84 (1H, dd, *J* = 7.9 and 1.4 Hz, H13), 6.77-6.73 (3H, m, H1 and H12), 6.08 (1H, t, *J* = 11.1 Hz, H8), 5.49 (1H, dd, *J* = 11.1 and 8.6 Hz, H7), 4.93-4.87 (1H, m, H6), 4.26 (1H, s, ArO*H*), 4.23-4.16 (2H, m, H3), 3.47-3.40 and 3.35-3.28 (2 × 1H, m, diastereotopic H4), 3.29 (3H, s, OC*H*$_3$), 1.94-1.86 and 1.65-1.57 (2 × 1H, m, diastereotopic H5), 1.36 (1H, br s, 2° O*H*); **^{13}C NMR** (125 MHz, C_6D_6) δ_C 160.0, 152.5, 136.4, 130.7, 129.5, 129.5, 129.4, 129.4, 128.5, 127.7, 119.7, 118.6, 115.3, 114.1, 73.0, 67.7, 67.2, 54.8, 37.7; **HRMS** (ES^+) calc. for $C_{21}H_{23}ClNaO_4$ $[^{35}ClM+Na]^+$ 397.1177, found 397.1165.

1.8 Cross-Coupling of Acyclic Siloxanes

1-Methoxy-4-((((1*E*,3*Z*)-1-phenylundeca-1,3-dien-5-yl)oxy)methyl)benzene, 5az

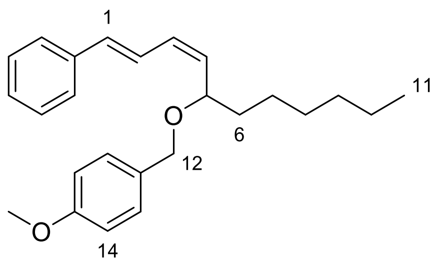

Procedure F: A solution of TBAF (0.37 mL of a 1 M solution in THF, 0.370 mmol, 3.0 equiv.) was added to iodide **4a** (28 mg, 0.123 mmol, 1.0 equiv.), acyclic silane **1z** (50 mg, 0.123 mmol, 1.0 eq, 94:6 *Z*:*E*) and allylpalladium chloride dimer (1 mg, 0.003 mmol, 0.025 equiv.) at room temperature and stirred for 24 hours. The concentrated crude was purified by flash column chromatography (petrol) to give diene **5az** as a yellow oil (8 mg, 0.022 mmol, 18%, 85:15 *Z,E*:*E,E*); **R_f** 0.24 (petrol / Et_2O (19:1)); **IR** (thin film, ν_{max} / cm^{-1}) 2929, 2857, 1612, 1511, 1464, 1301, 1246, 1173, 1072, 1037; **^{1}H NMR** (500 MHz, C_6D_6) δ_H 7.32 (2H, d, *J* = 7.8 Hz, H13), 7.28 (2H, d, *J* = 7.2 Hz, *o*-Ar*H*), 7.15–7.10 (3H, m, *m*-Ar*H* and H2), 7.04 (1H, t, *J* = 7.2 Hz, *p*-Ar*H*), 6.82 (2H, d, *J* = 7.8 Hz, H14), 6.47 (1H, d, *J* = 15.6 Hz, H1), 6.32 (1H, app t, *J* = 10.5 Hz, H3), 5.52 (1H, app t, *J* = 10.5 Hz, H4), 4.69 and 4.39 (2 × 1H, d, *J* = 11.4 Hz, diastereotopic H12), 4.47-4.43 (1H, m, H5), 3.31 (3H, s, OC*H*$_3$), 1.92-1.83 and 1.68-1.61 (2 × 1H, m, diastereotopic H6), 1.57-1.40 (2H, m, H7), 1.32-1.21 (6H, m, H8, H9 and H10), 0.86 (3H, t, *J* = 7.0 Hz, H11); **^{13}C NMR** (125 MHz, C_6D_6) δ_C 159.7, 137.6, 134.4, 133.7, 132.0, 131.6, 129.8, 129.4, 128.9, 126.9, 124.4, 114.1, 73.7, 69.7, 54.7, 36.5, 32.2, 29.8, 25.8, 23.0, 14.3; **HRMS** (ES^+) calc. for $C_{25}H_{32}NaO_2$ $[M+Na]^+$ 387.2295, found 387.2286.

2. NMR Data of Novel Compounds

1-(Diethyl(isopropoxy)silyl)non-1-yn-3-ol, 2a

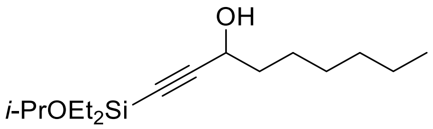

^{1}H (400 MHz, $CDCl_3$)

^{13}C (125 MHz, $CDCl_3$)

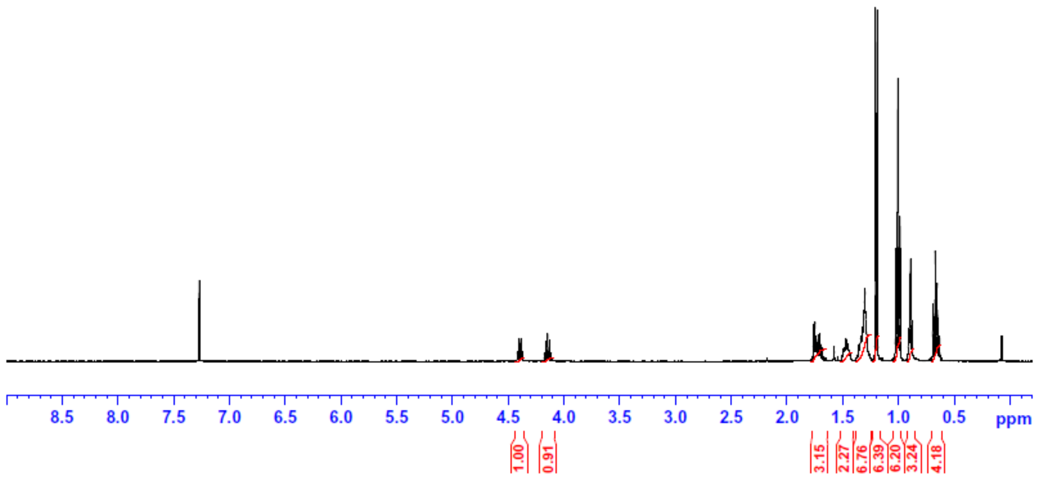

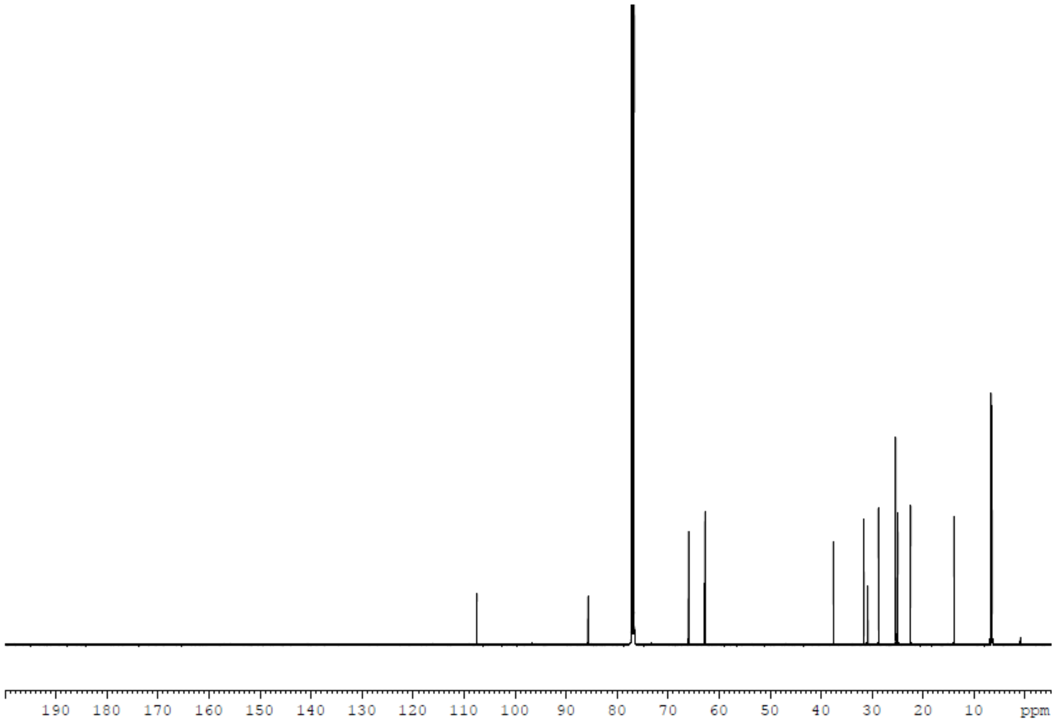

1-(Diethyl(isopropoxy)silyl)non-1-yn-3-yl acetate, 2b

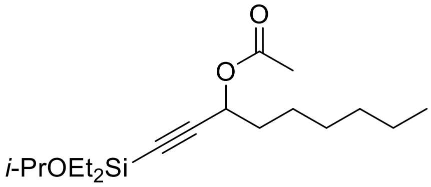

^{1}H (500 MHz, $CDCl_3$)

^{13}C (125 MHz, $CDCl_3$)

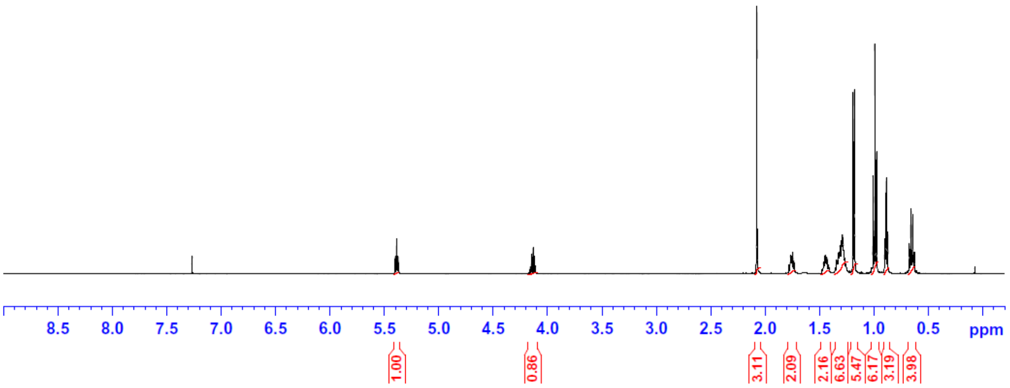

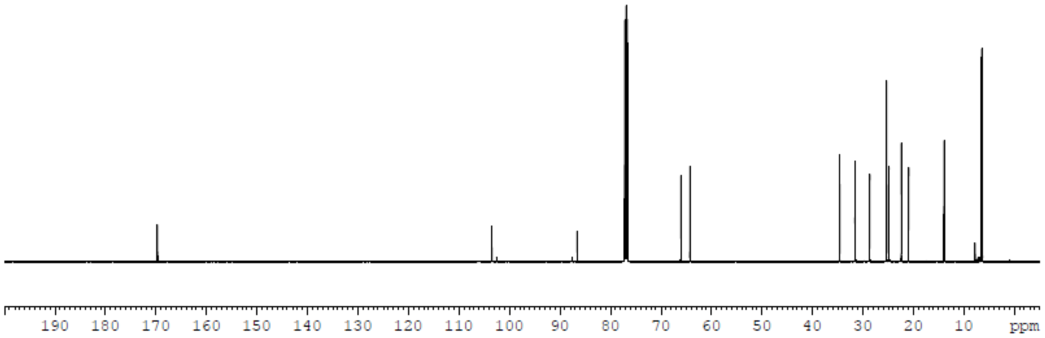

1-(Diethyl(isopropoxy)silyl)non-1-yn-3-yl benzoate, 2c

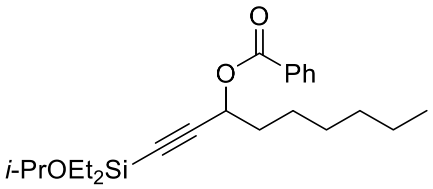

^{1}H (400 MHz, $CDCl_3$)

^{13}C (101 MHz, $CDCl_3$)

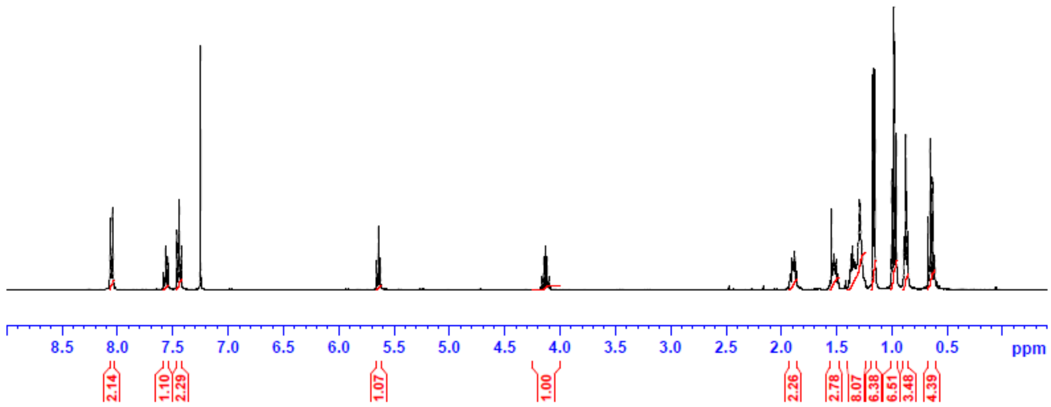

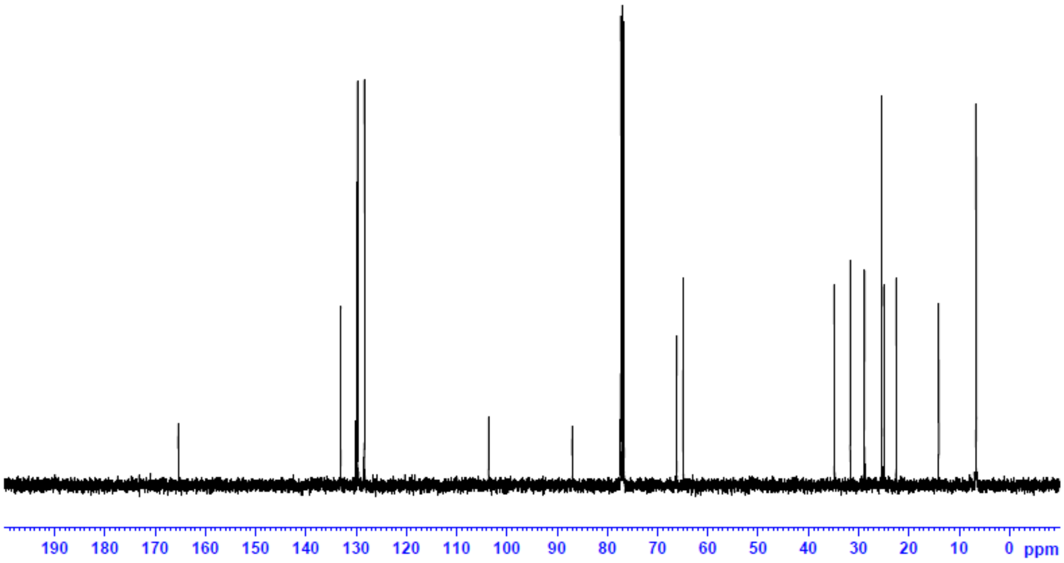

Diethyl(isopropoxy)(3-((4-methoxybenzyl)oxy)non-1-yn-1-yl)silane, 2z

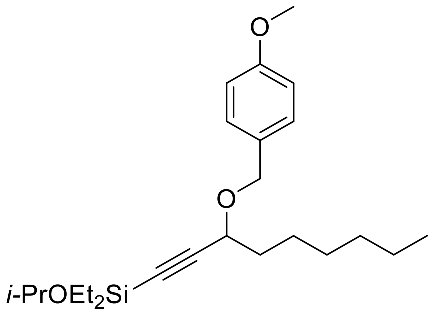

^{1}H (400 MHz, $CDCl_3$)

^{13}C (100 MHz, $CDCl_3$)

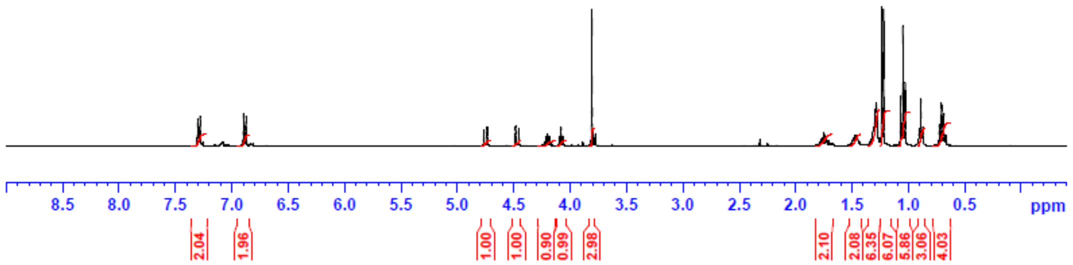

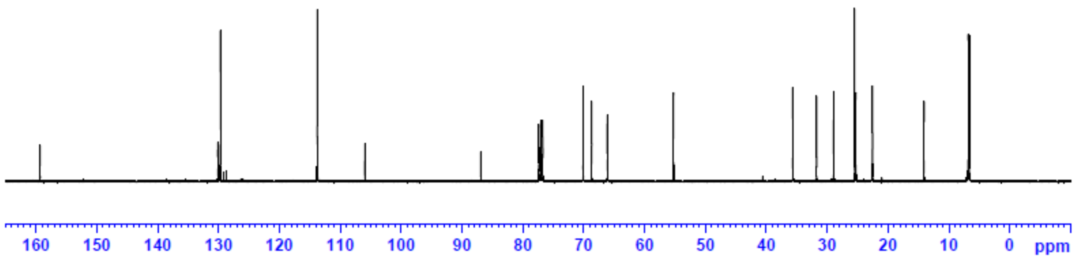

1-Cyclohexyl-3-(diethyl(isopropoxy)silyl)prop-2-yn-1-ol, 2d-S1

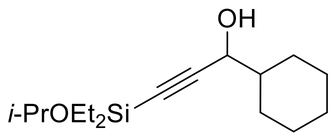

^{1}H (400 MHz, $CDCl_3$)

^{13}C (100 MHz, $CDCl_3$)

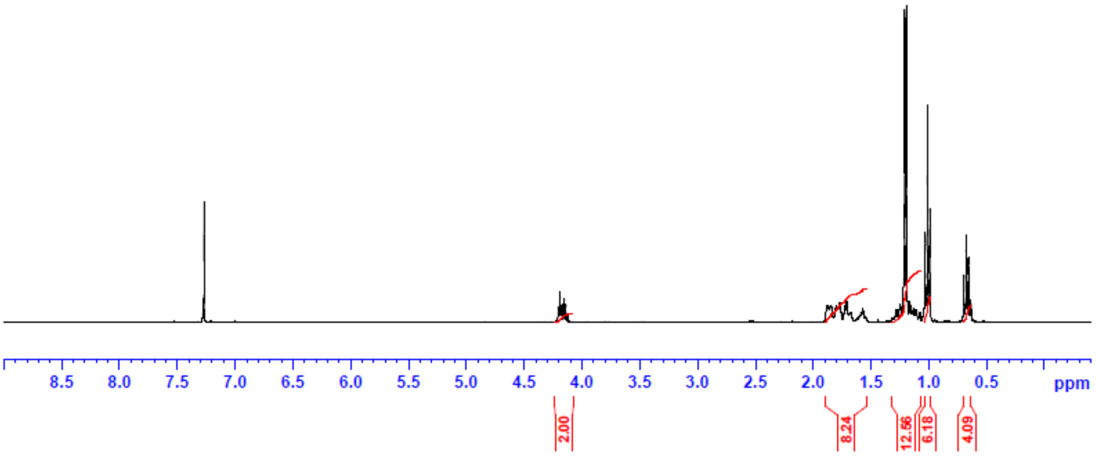

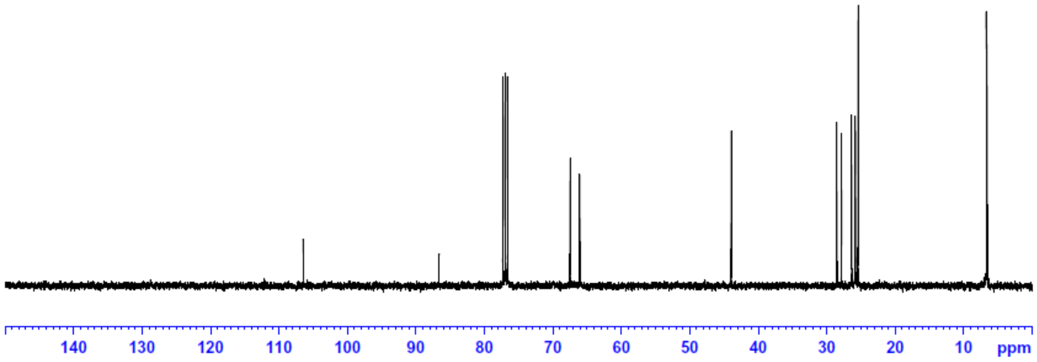

1-Cyclohexyl-3-(diethyl(isopropoxy)silyl)prop-2-yn-1-yl acetate, 2d

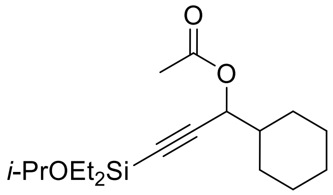

^{1}H (400 MHz, CDCl$_3$)

^{13}C (100 MHz, CDCl$_3$)

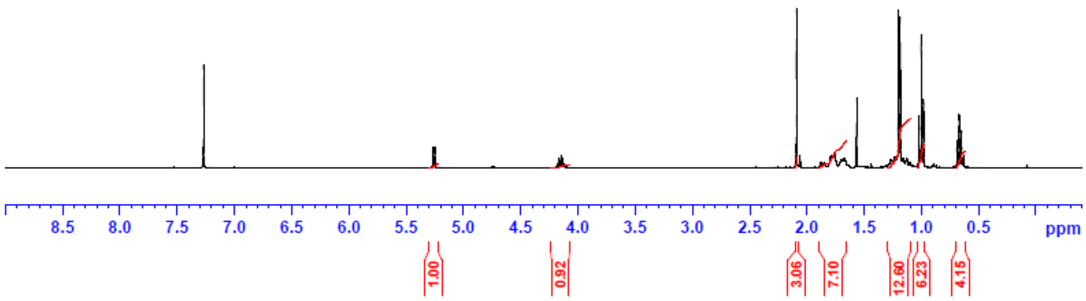

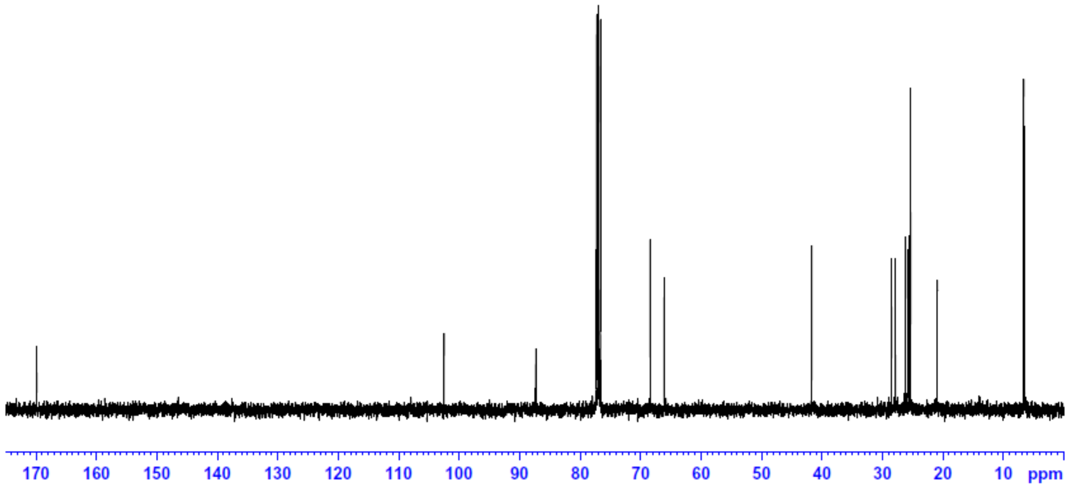

10,10-Diethyl-2,2,3,3,12-pentamethyl-4,11-dioxa-3,10-disilatridec-8-yn–7-ol, 2e-S3

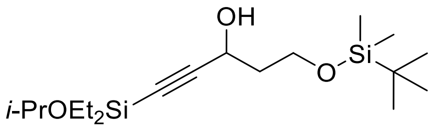

^{1}H (400 MHz, CDCl$_3$)

^{13}C (100 MHz, CDCl$_3$)

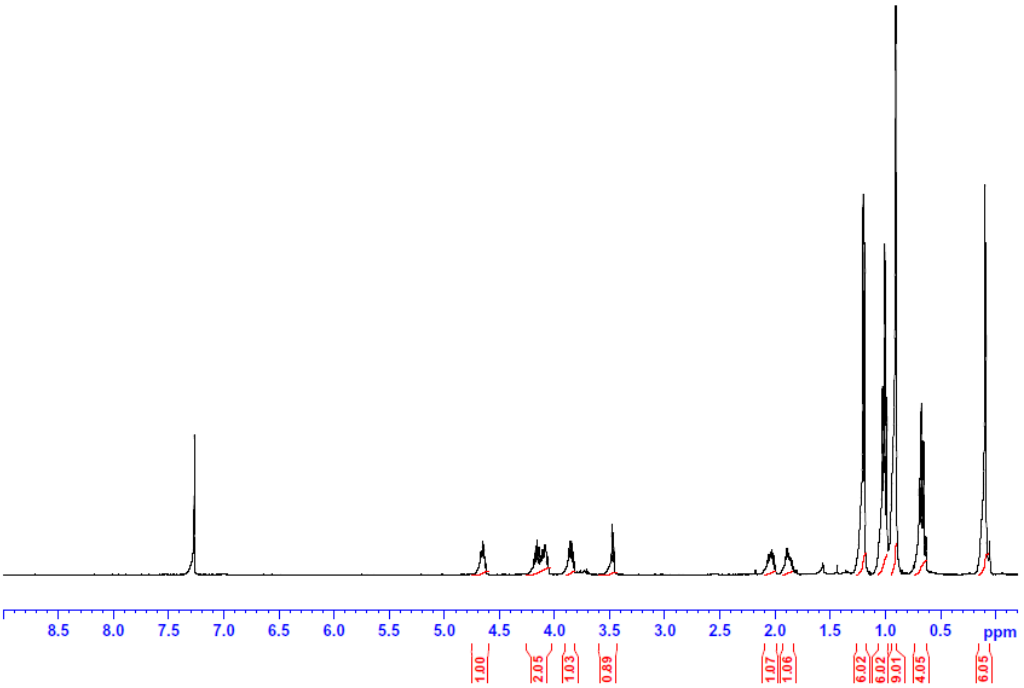

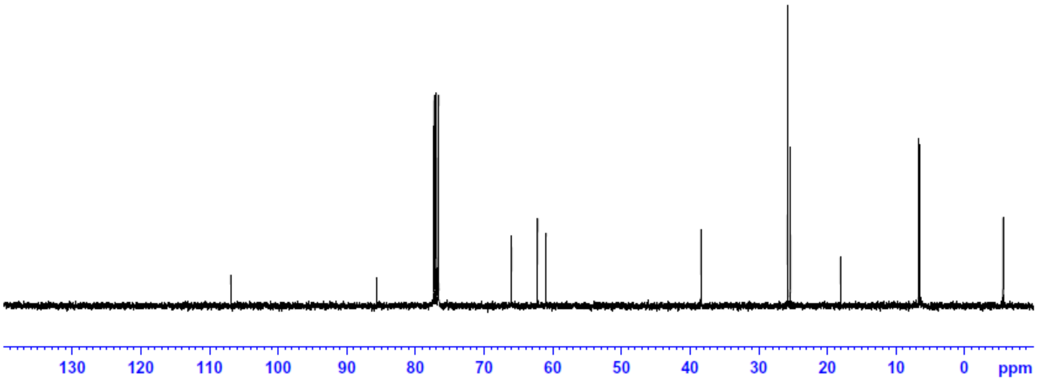

10,10-Diethyl-2,2,3,3,12-pentamethyl-4,11-dioxa-3,10-disilatridec-8-yn–7-yl acetate, 2e

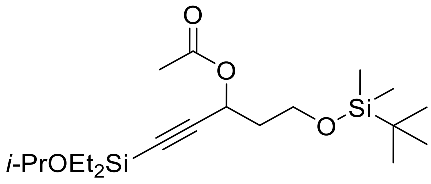

^{1}H (400 MHz, CDCl$_3$)

^{13}C (101 MHz, CDCl$_3$)

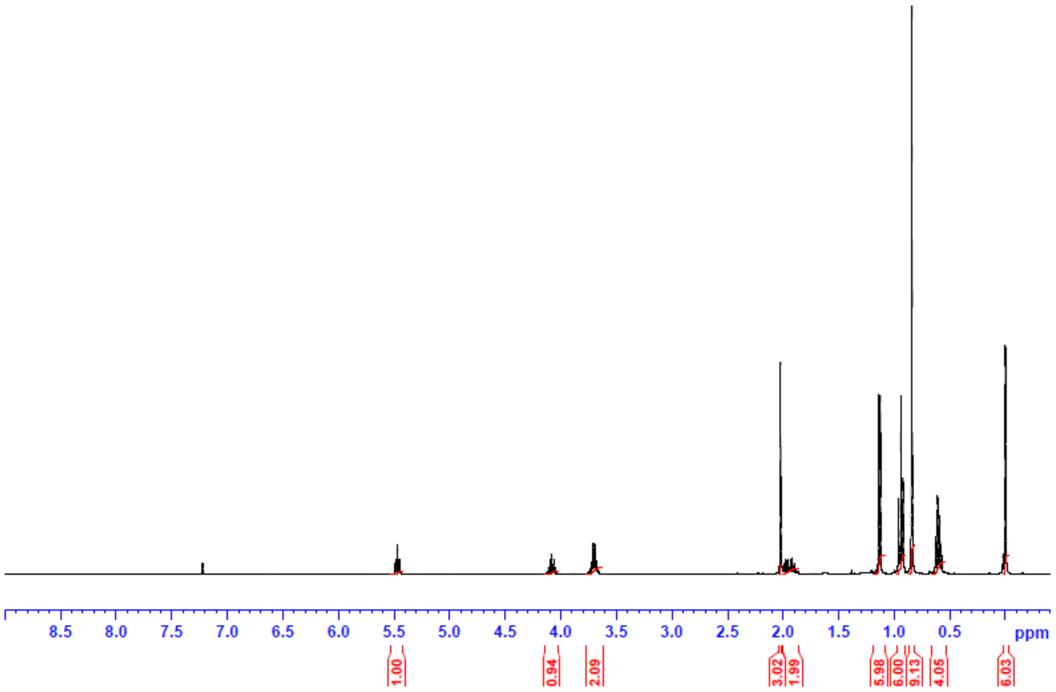

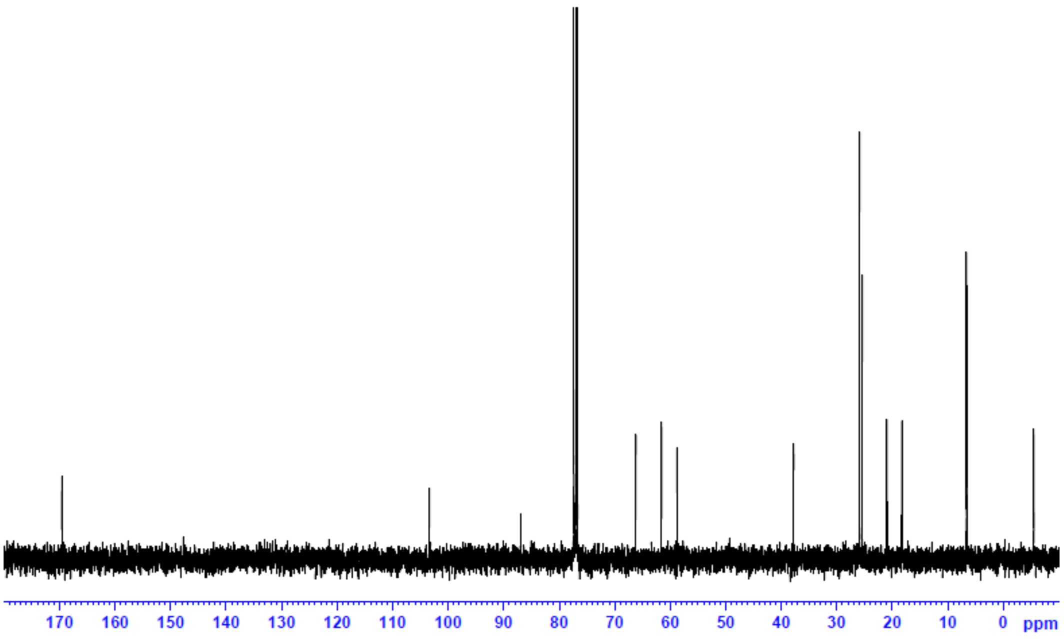

1-(Diethyl(isopropoxy)silyl)-5-((4-methoxybenzyl)oxy)pent-1-yn-3-ol, 2f-S3

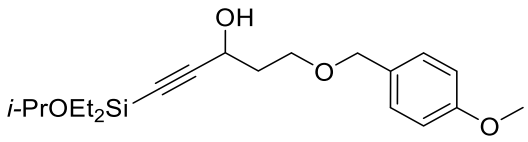

^{1}H (400 MHz, $CDCl_3$)

^{13}C (100 MHz, $CDCl_3$)

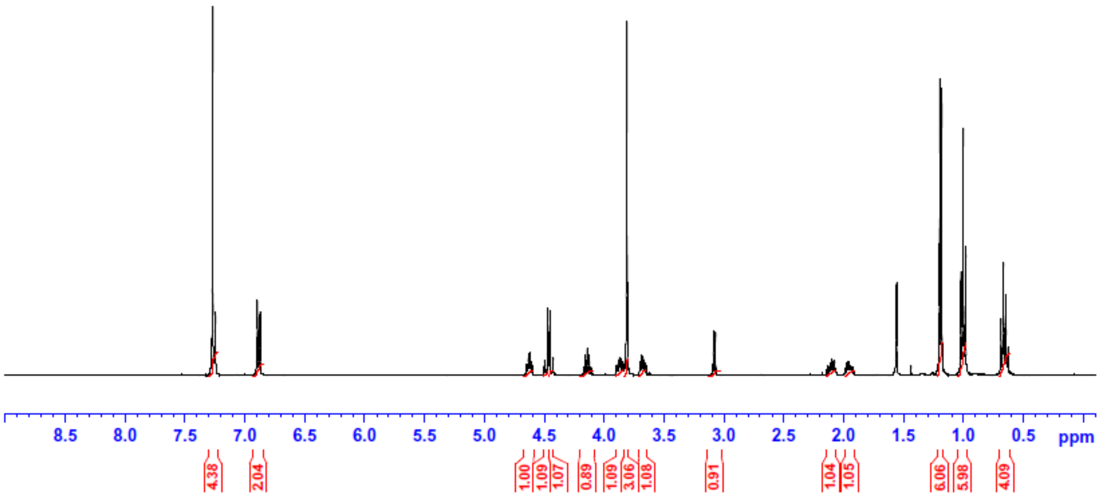

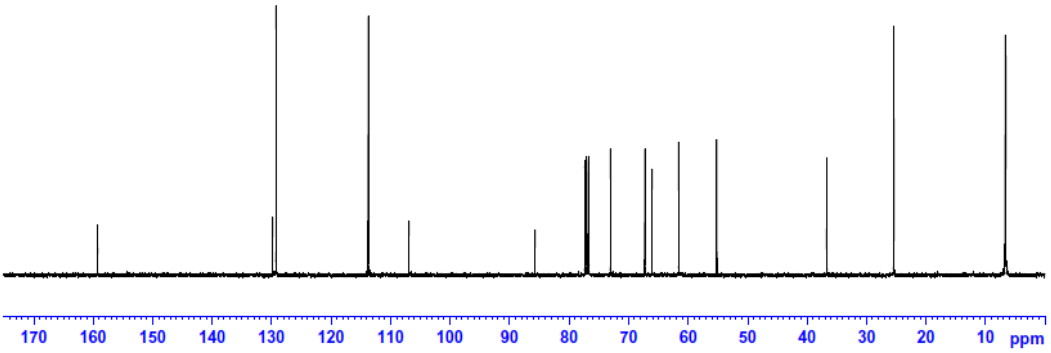

1-(Diethyl(isopropoxy)silyl)-5-((4-methoxybenzyl)oxy)pent-1-yn-3-one, 2f-S4

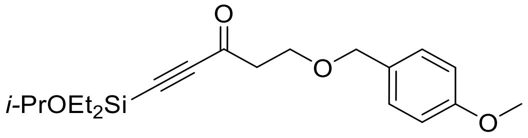

^{1}H (400 MHz, $CDCl_3$)

^{13}C (100 MHz, $CDCl_3$)

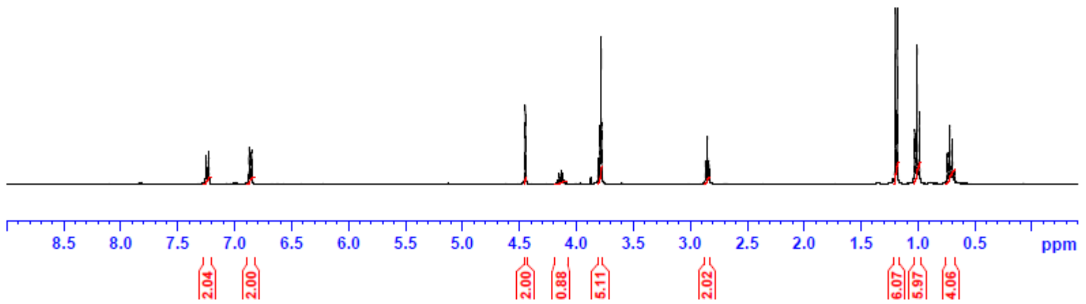

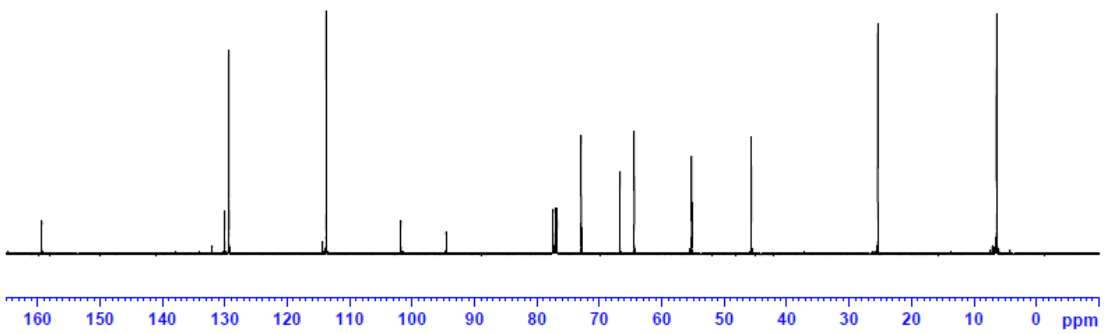

1-(diethyl(isopropoxy)silyl)-5-((4-methoxybenzyl)oxy)pent-1-yn-3-yl, 2f

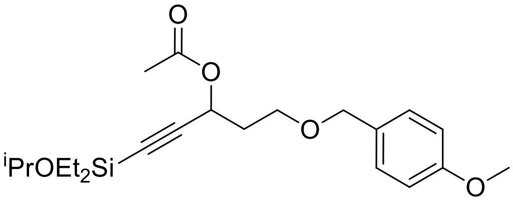

^{1}H (400 MHz, $CDCl_3$)

^{13}C (100 MHz, $CDCl_3$)

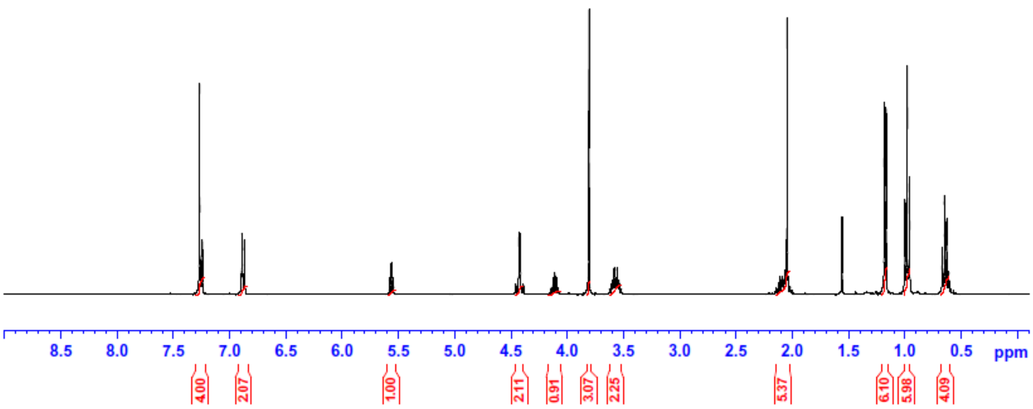

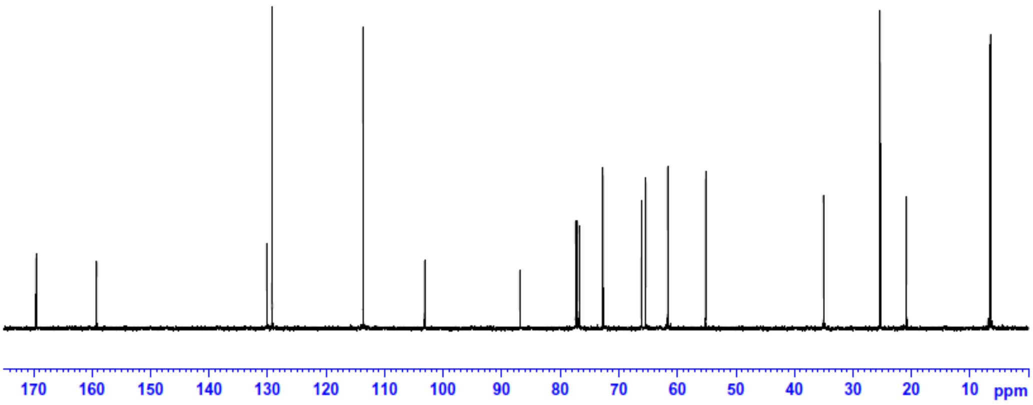

3-(Diethyl(isopropoxy)silyl)-1-phenylprop-2-yn-1-ol, 2g-S1

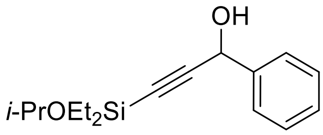

^{1}H (500 MHz, CDCl$_3$)

^{13}C (125 MHz, CDCl$_3$)

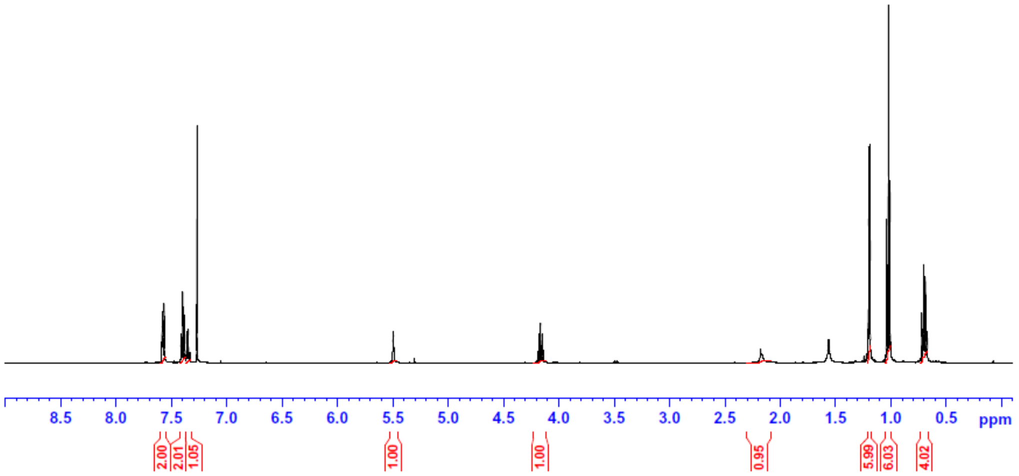

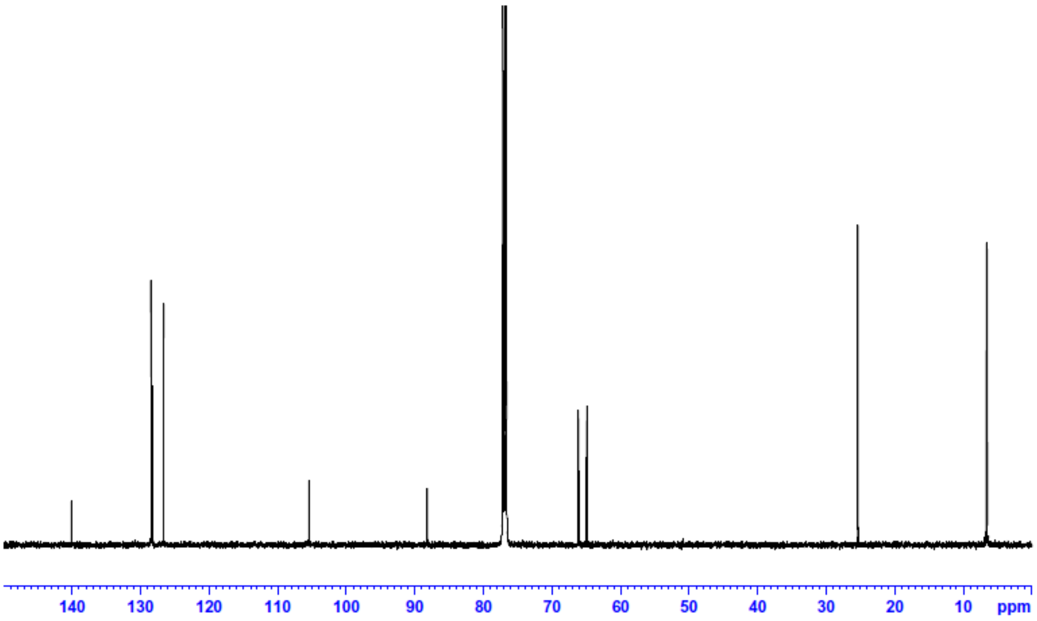

Diethyl(3-hydroxy-3-phenylprop-1-yn-1-yl)silanol, 2g

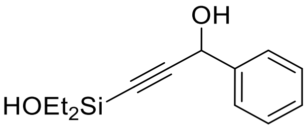

^{1}H (400 MHz, $CDCl_3$)

^{13}C (101 MHz, $CDCl_3$)

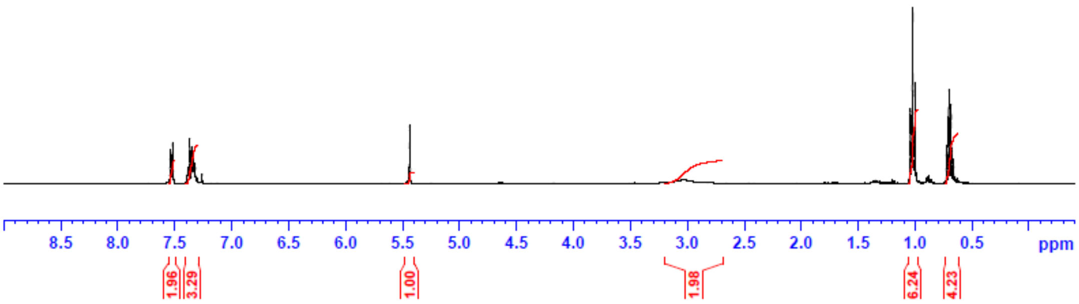

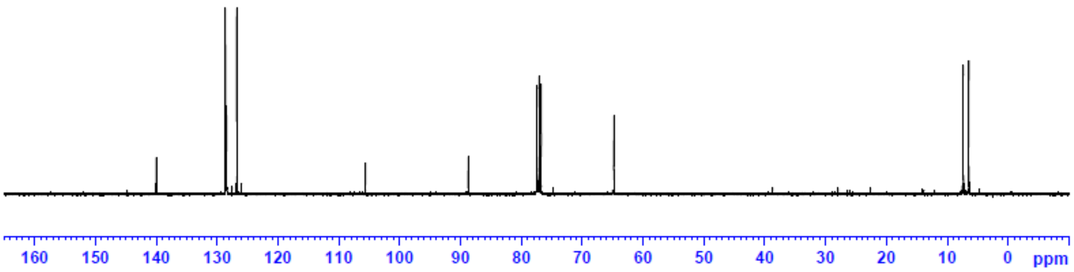

(3*S*,4*R*)-1-(Diethyl(hydroxy)silyl)-4,5-dihydroxy-4-methylpent-1-yn-3-yl benzoate, 2h

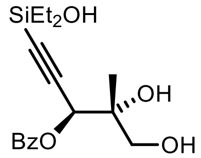

^{1}H (500 MHz, $CDCl_3$)

^{13}C (125 MHz, $CDCl_3$)

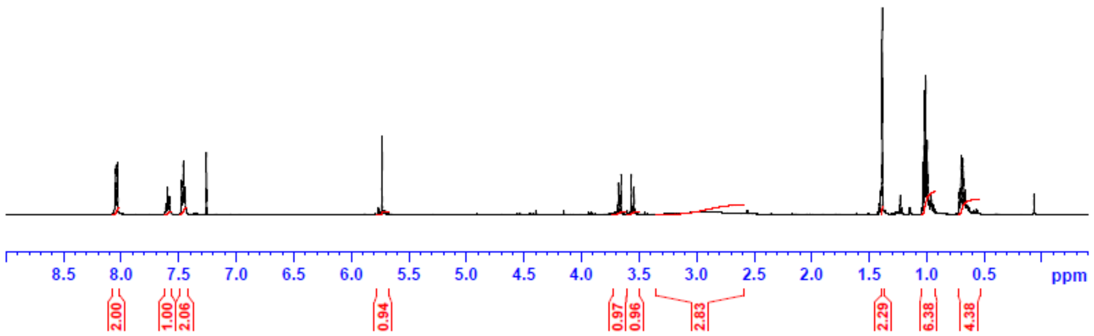

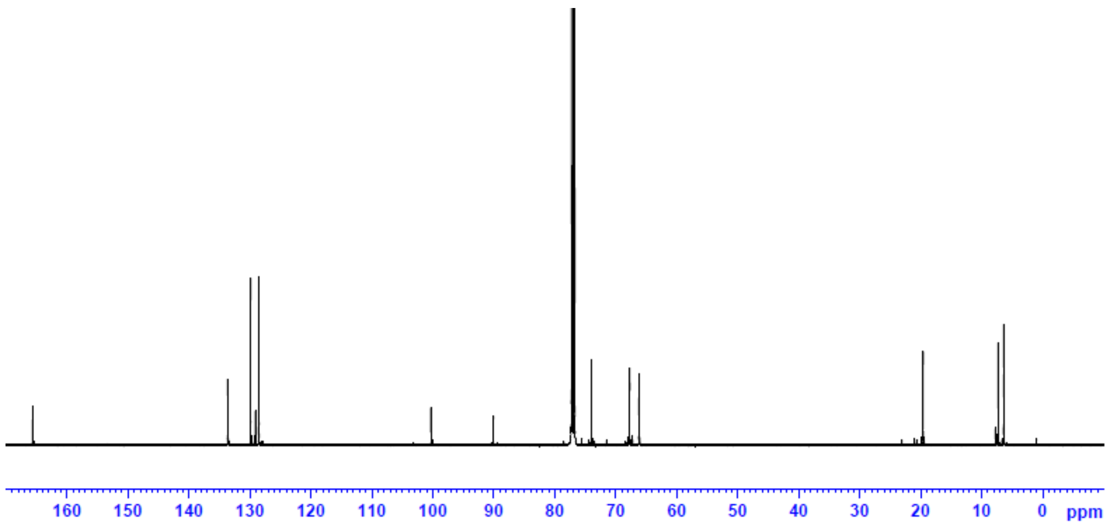

(*E*)-1-(((4-Iodobut-3-en-1-yl)oxy)methyl)-4-methoxybenzene, 2j-S2

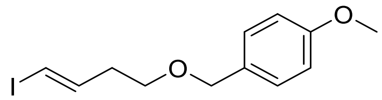

^{1}H (400 MHz, $CDCl_3$)

^{13}C (100 MHz, $CDCl_3$)

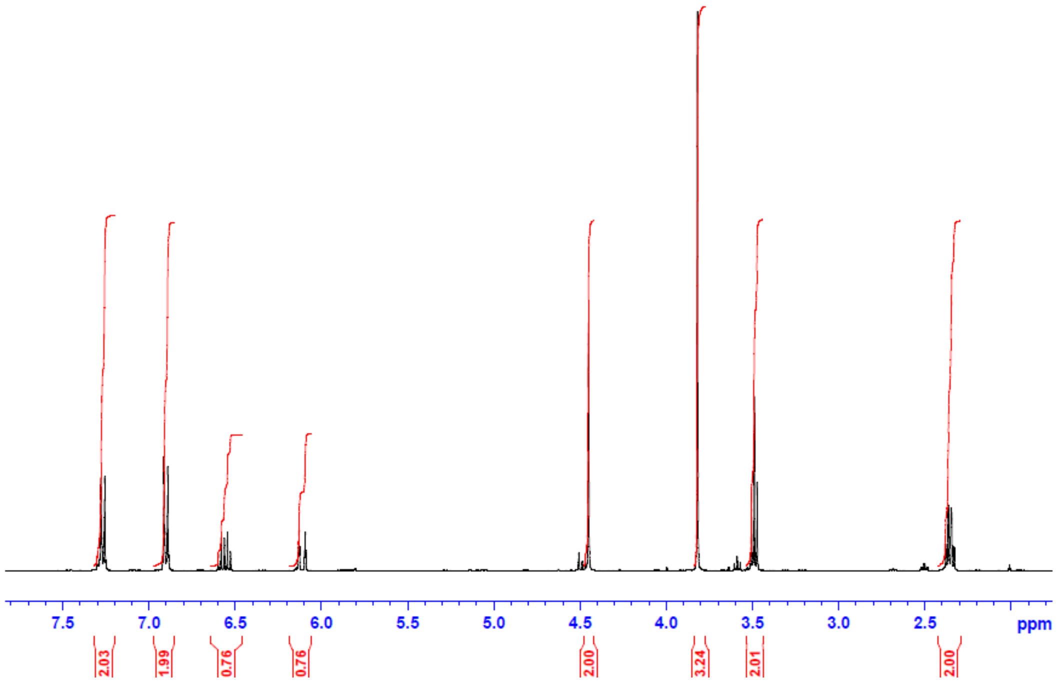

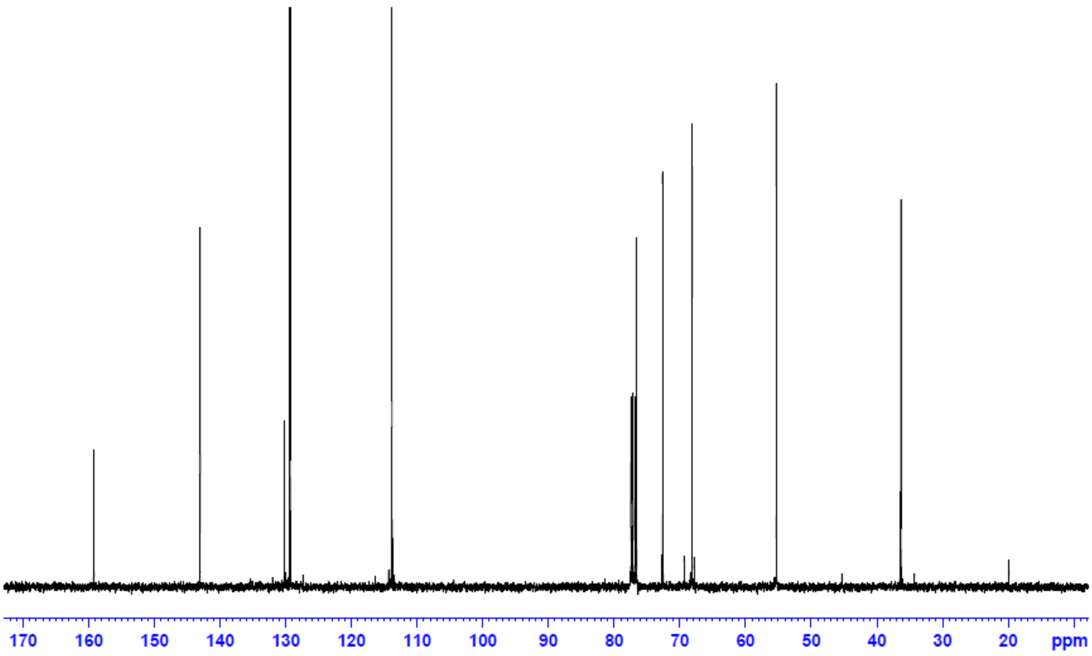

(*E*)-Diethyl(isopropoxy)(6-((4-methoxybenzyl)oxy)hex-3-en-1-yn-1-yl)silane, 2j-S3

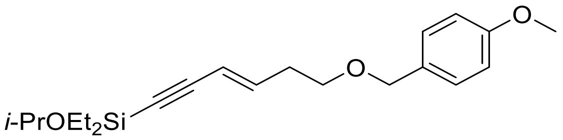

^{1}H (400 MHz, $CDCl_3$)

^{13}C (100 MHz, $CDCl_3$)

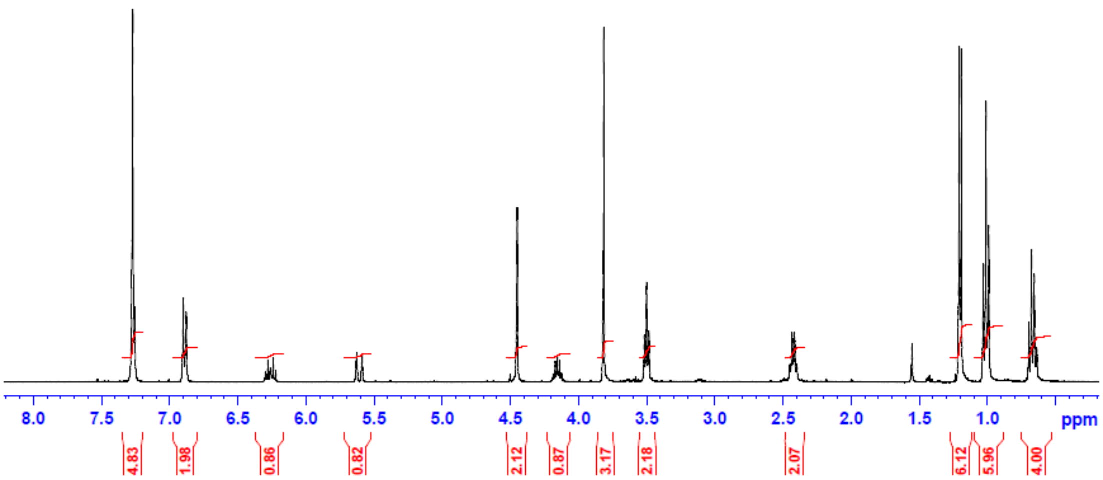

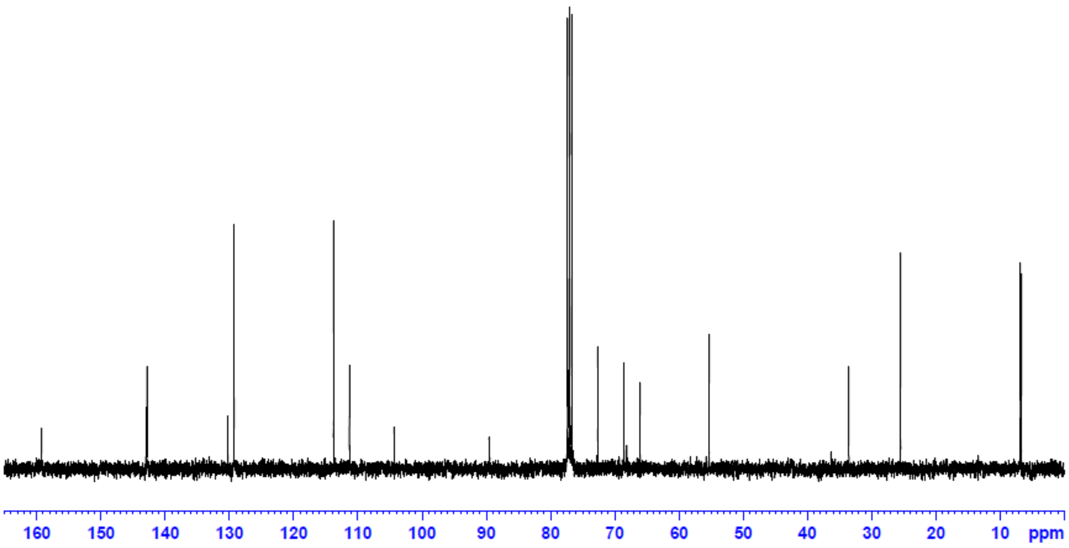

1-(Diethyl(isopropoxy)silyl)-6-((4-methoxybenzyl)oxy)hex-1-yne-3,4-diol, 2j

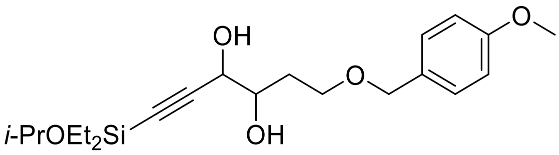

^{1}H (400 MHz, $CDCl_3$)

^{13}C (100 MHz, $CDCl_3$)

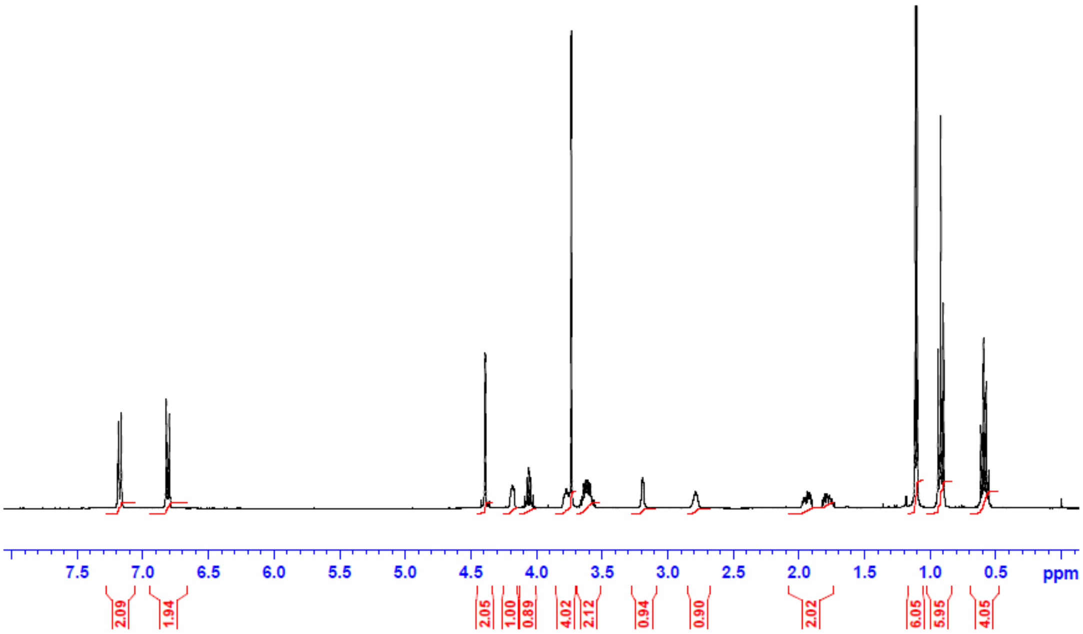

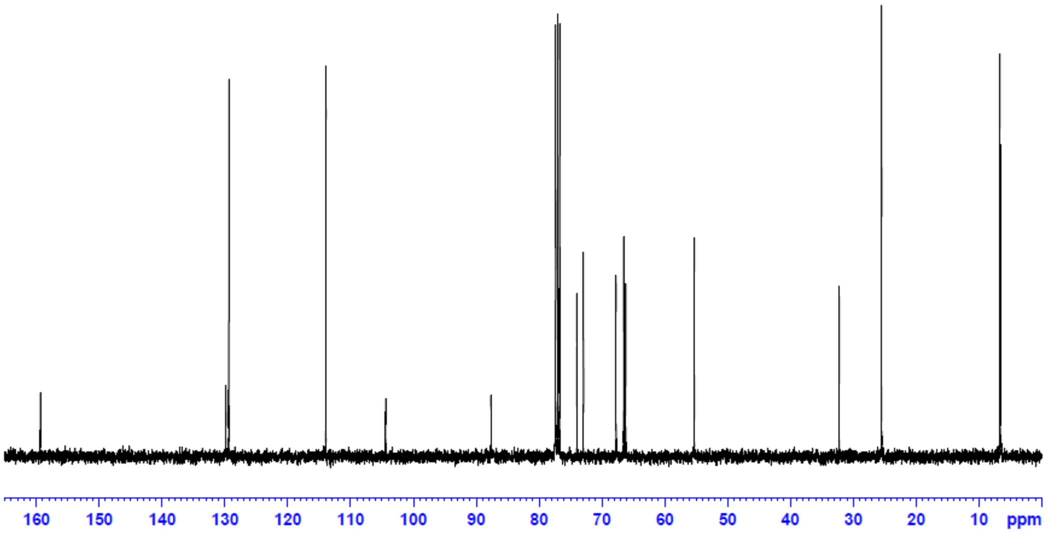

1-((Diethyl(isopropoxy)silyl)ethynyl)cyclohexan-1-ol, 2k

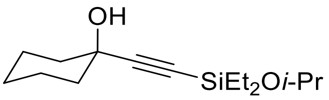

^{1}H (400 MHz, $CDCl_3$)

^{13}C (100 MHz, $CDCl_3$)

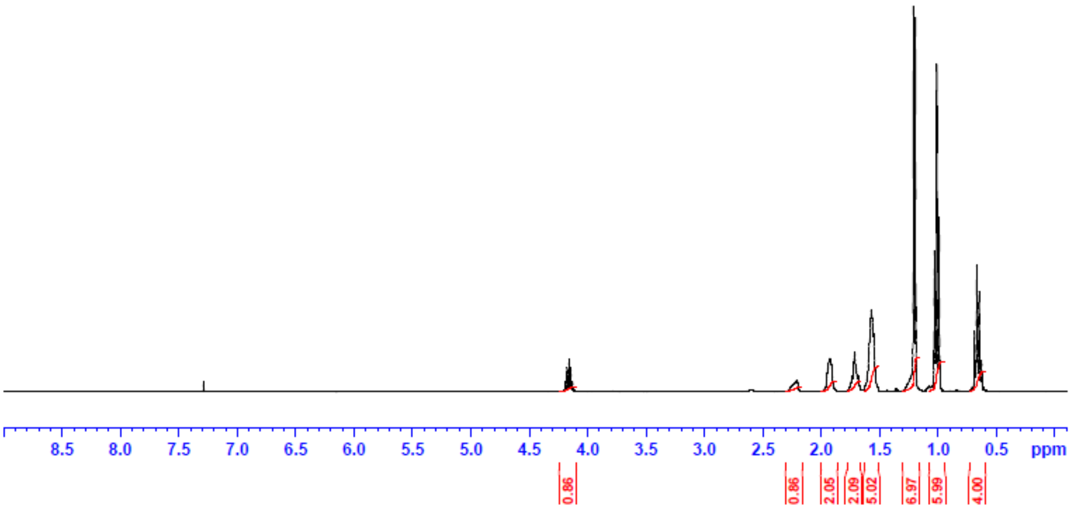

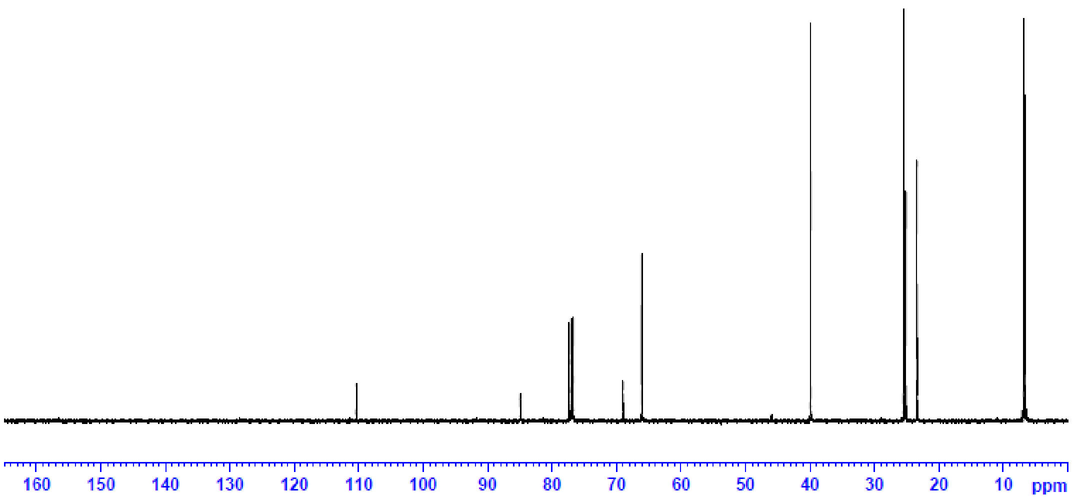

1-(Diethyl(isopropoxy)silyl)dec-1-yn-4-ol, 2l

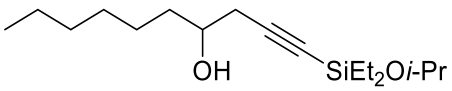

^{1}H (400 MHz, $CDCl_3$)

^{13}C (100 MHz, $CDCl_3$)

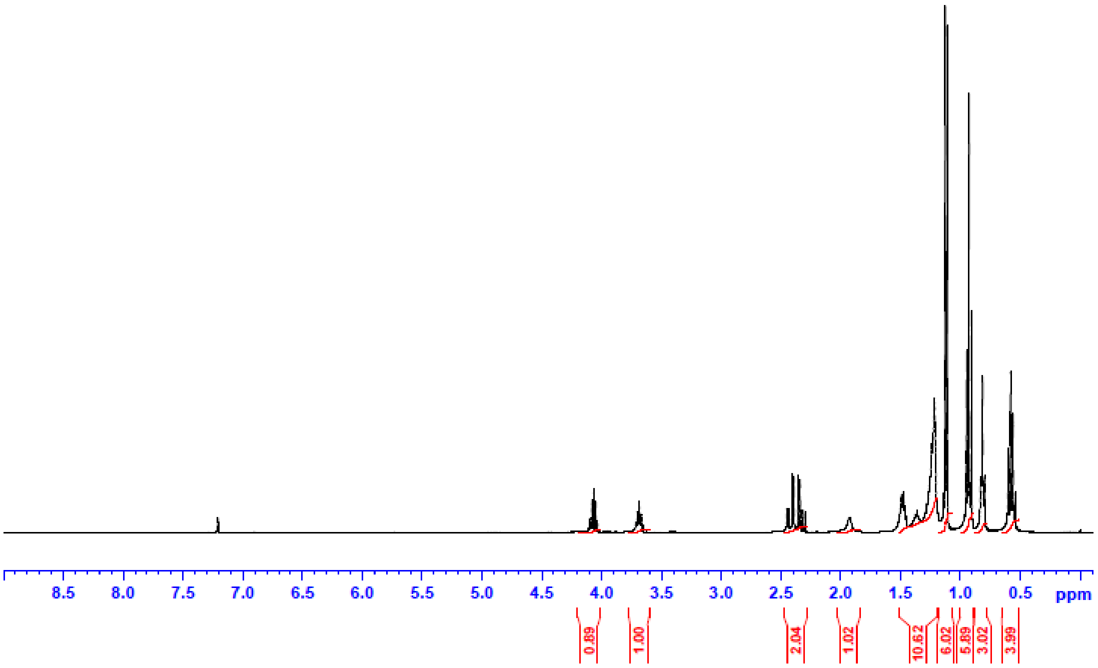

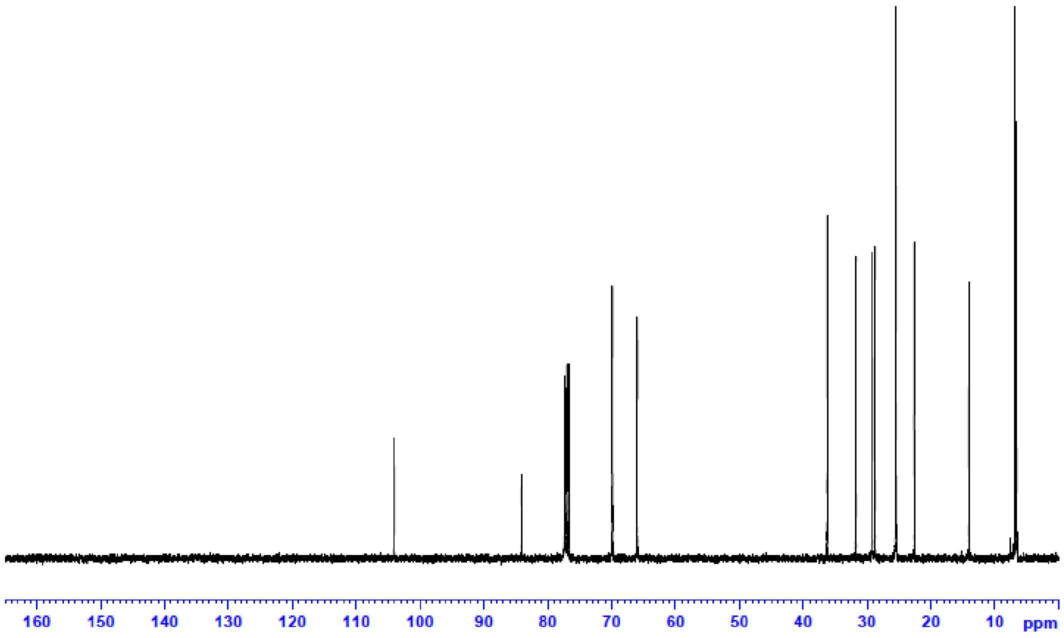

1-Cyclohexyl-4-(diethyl(isopropoxy)silyl)but-3-yn-1-ol, 2m

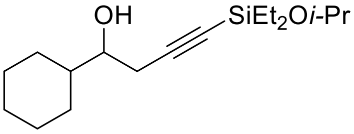

^{1}H (400 MHz, $CDCl_3$)

^{13}C (100 MHz, $CDCl_3$)

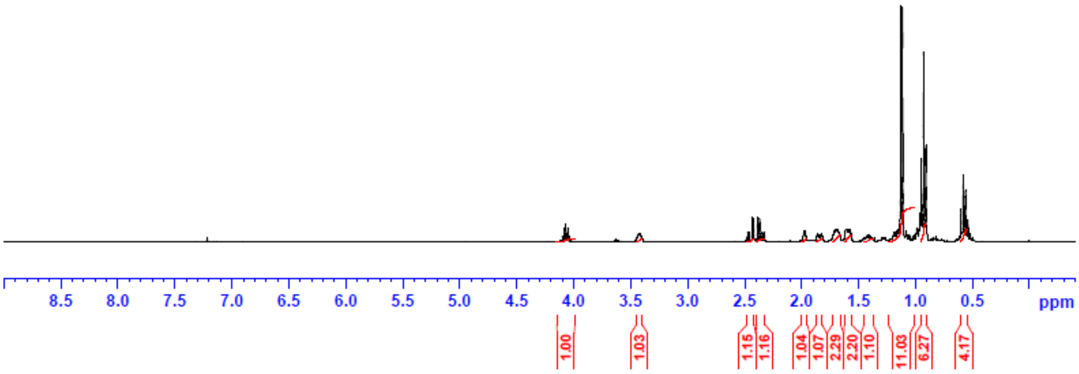

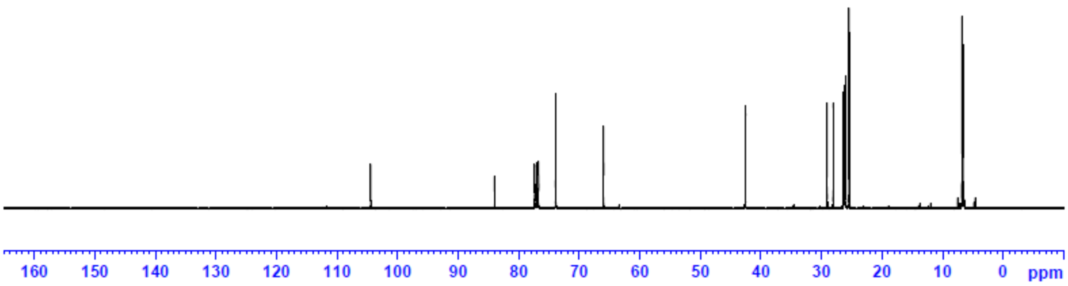

11,11-Diethyl-2,2,3,3,13-pentamethyl-4,12-dioxa-3,11-disilatetradec-9-yn–7-ol, 2n

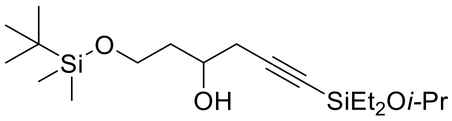

^{1}H (400 MHz, $CDCl_3$)

^{13}C (100 MHz, $CDCl_3$)

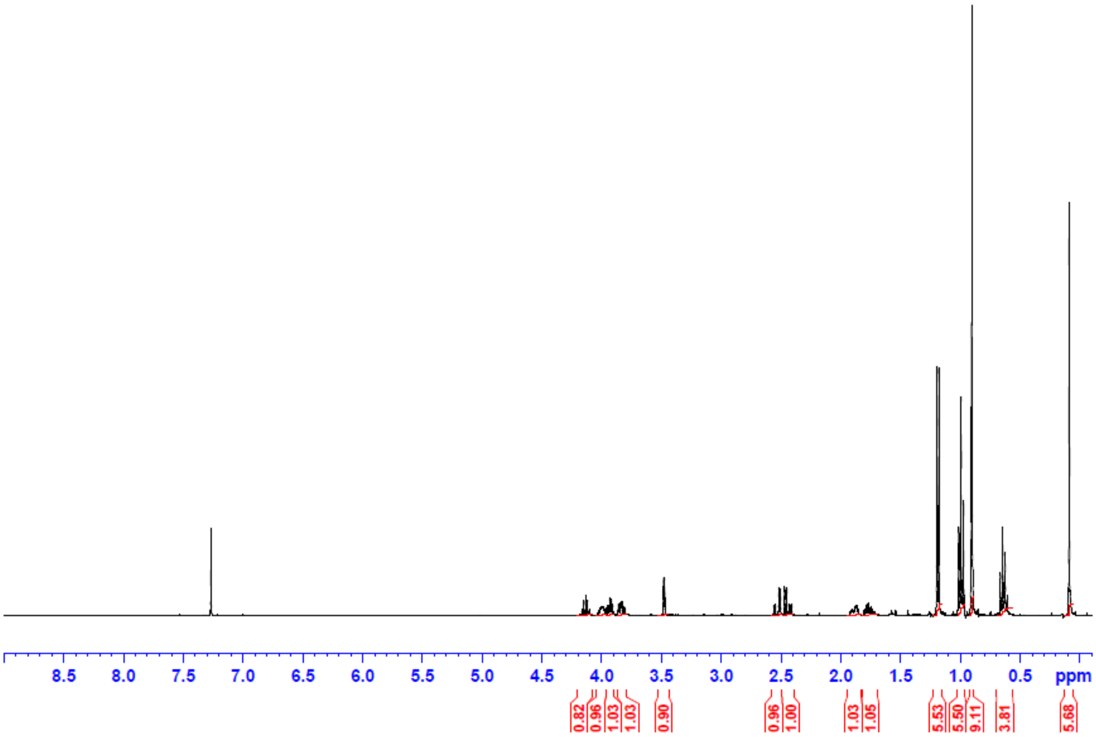

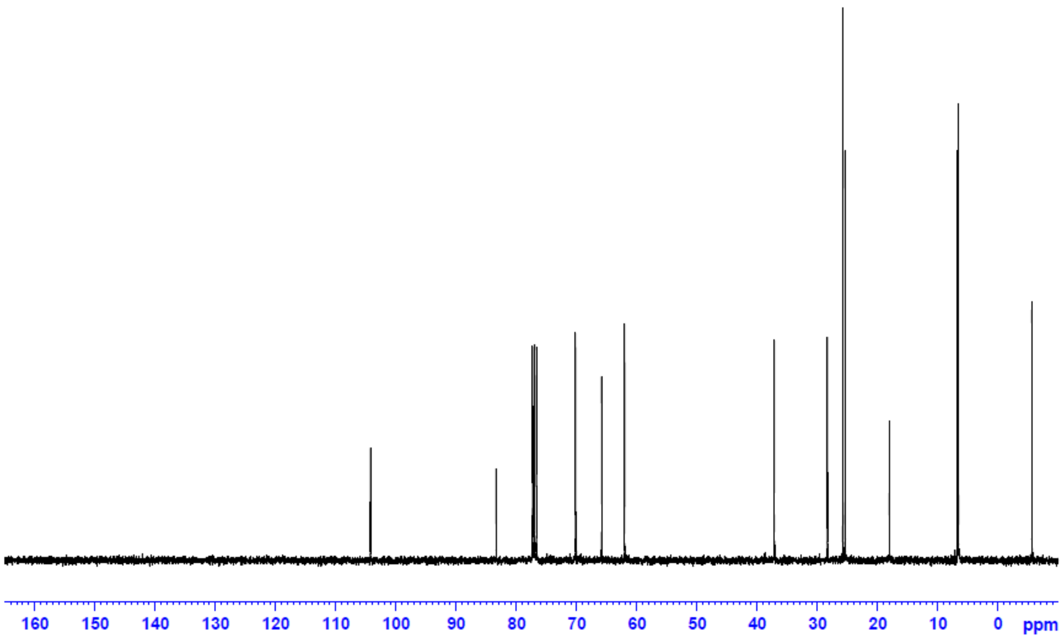

6-(Diethyl(isopropoxy)silyl)-1-((4-methoxybenzyl)oxy)hex-5-yn-3-ol, 2o

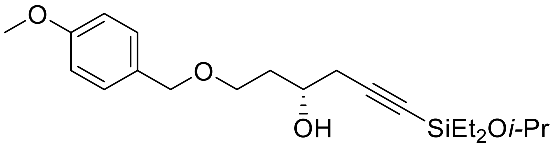

^{1}H (400 MHz, $CDCl_3$)

^{13}C (100 MHz, $CDCl_3$)

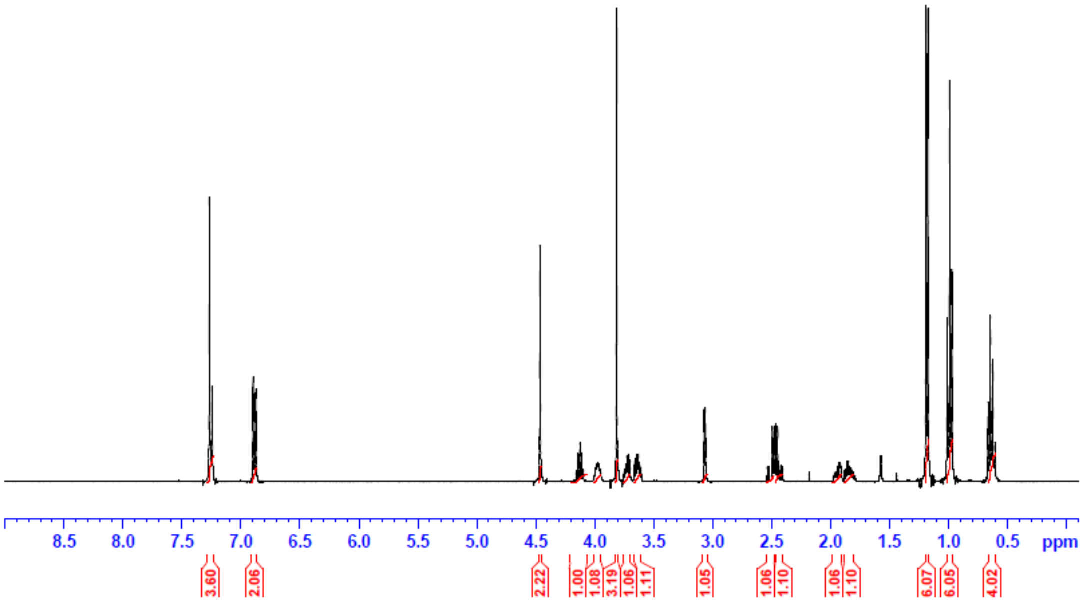

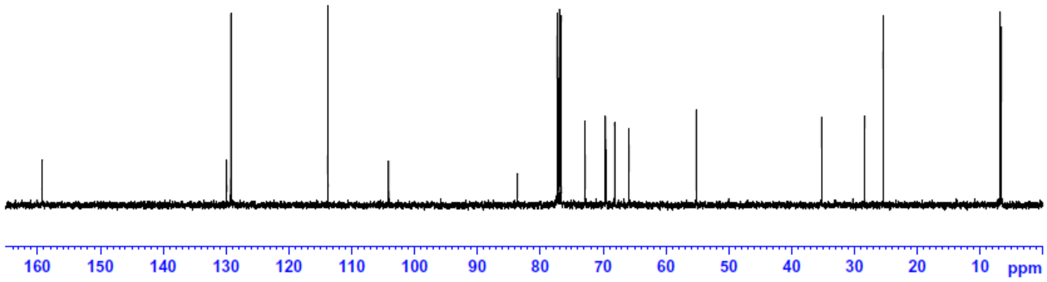

2-((Diethyl(isopropoxy)silyl)ethynyl)cyclohexan-1-ol, 2p

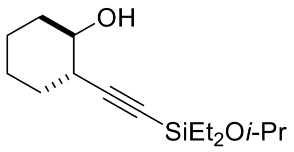

^{1}H (400 MHz, $CDCl_3$)

^{13}C (100 MHz, $CDCl_3$)

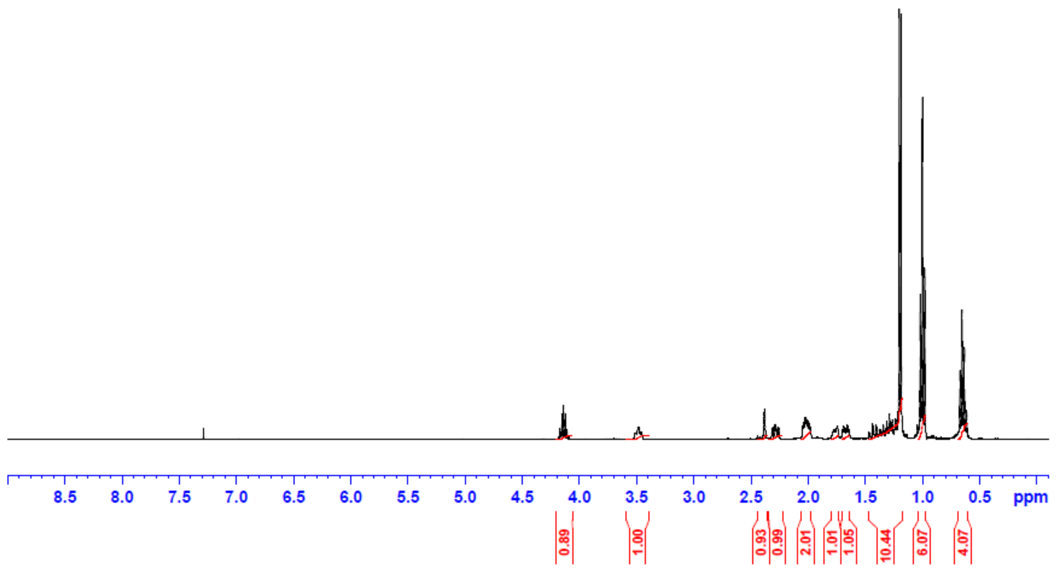

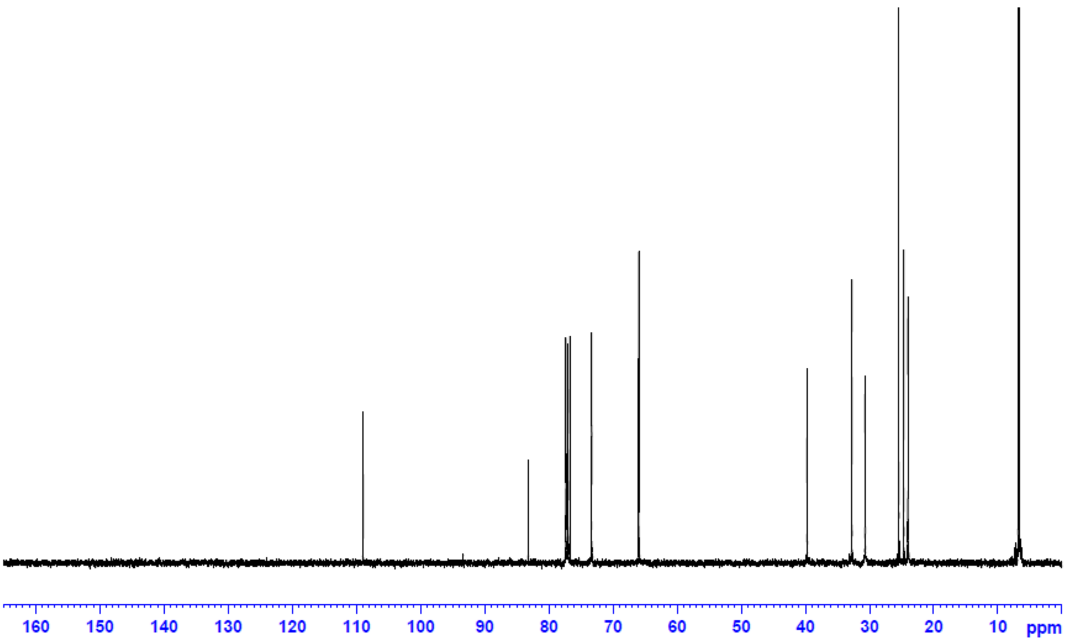

2,2-Diethyl-5-hexyl-2,5-dihydro-1,2-oxasilole, 1a

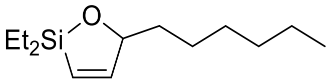

^{1}H (500 MHz, $CDCl_3$)

^{13}C (125 MHz, $CDCl_3$)

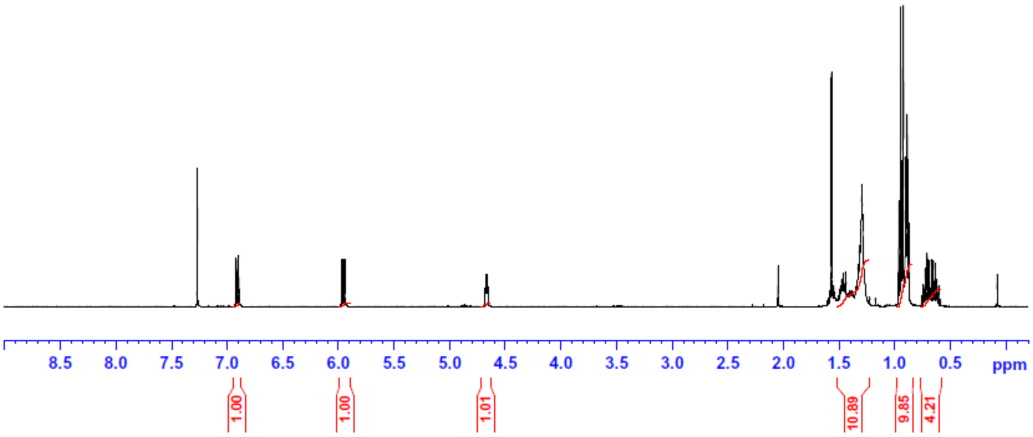

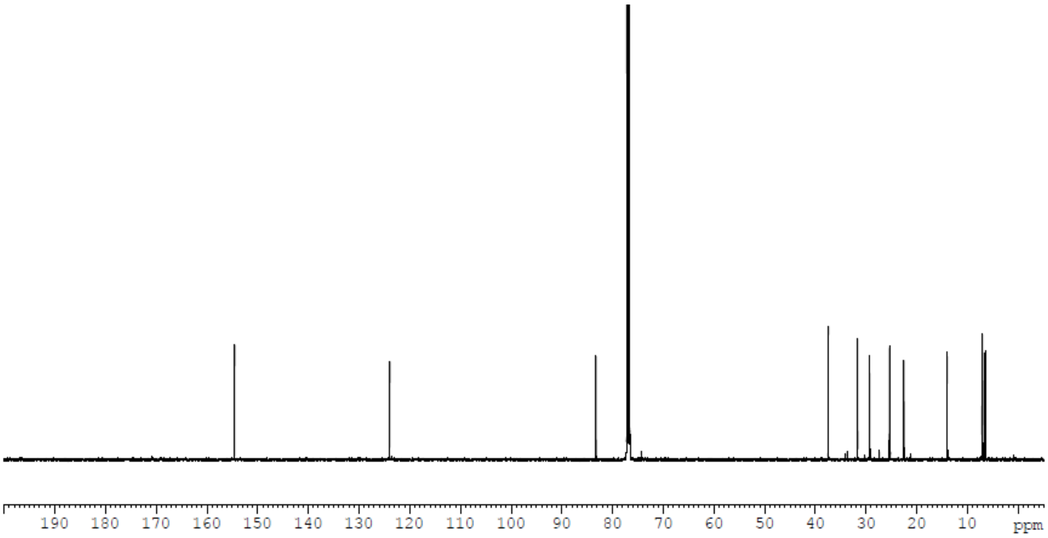

(*E*)-1-(diethyl(methoxy)silyl)non-1-en-3-ol, 9

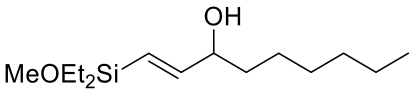

^{1}H (500 MHz, $CDCl_3$)

^{13}C (125 MHz, $CDCl_3$)

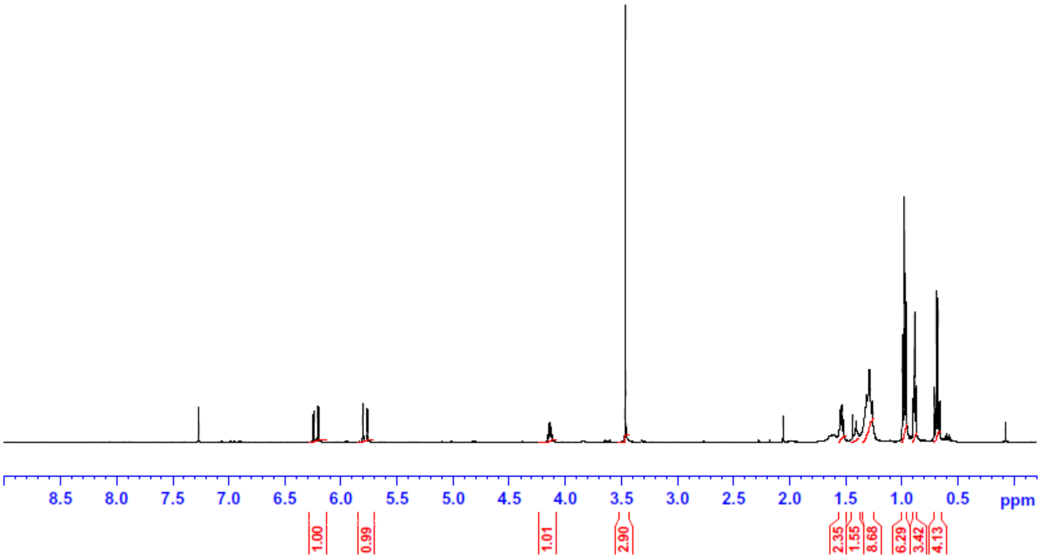

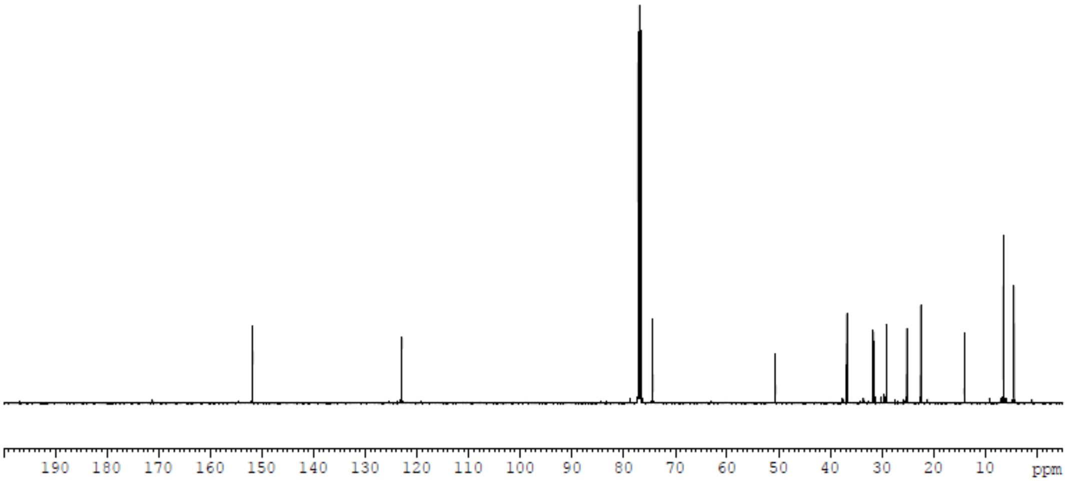

(*Z*)-Diethyl(isopropoxy)(3-((4-methoxybenzyl)oxy)non-1-en-1-yl)silane, 1z

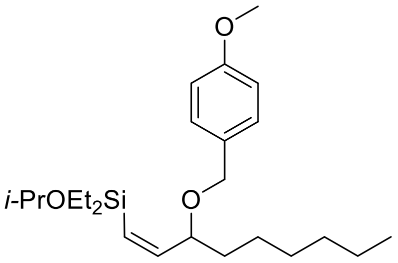

^{1}H (400 MHz, $CDCl_3$)

^{13}C (100 MHz, $CDCl_3$)

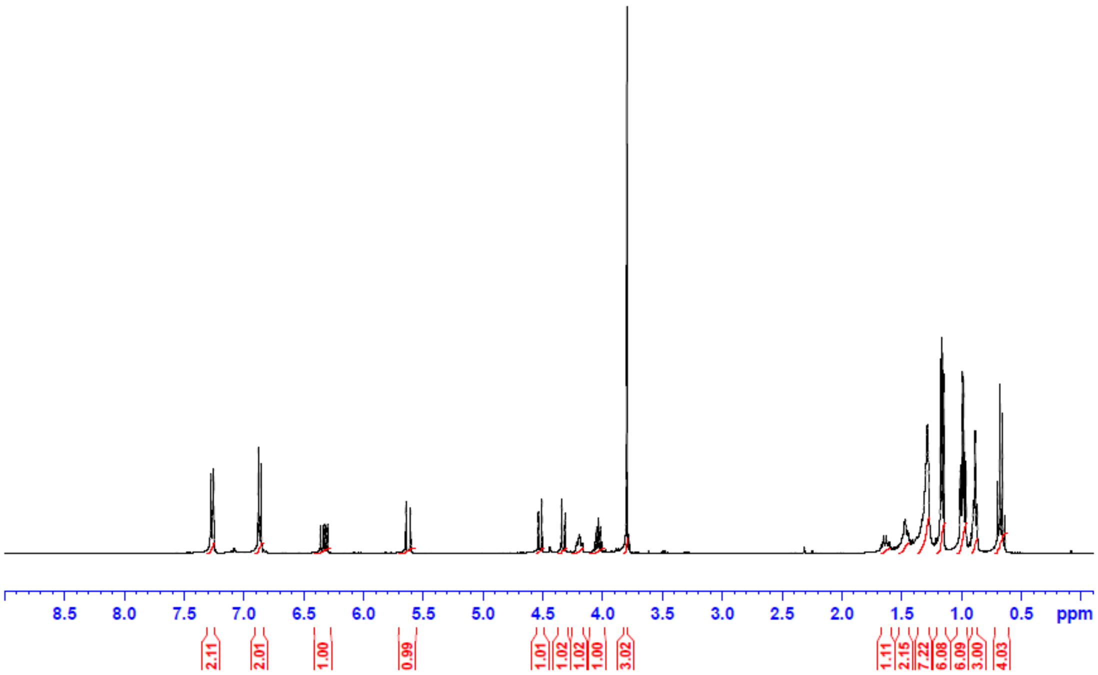

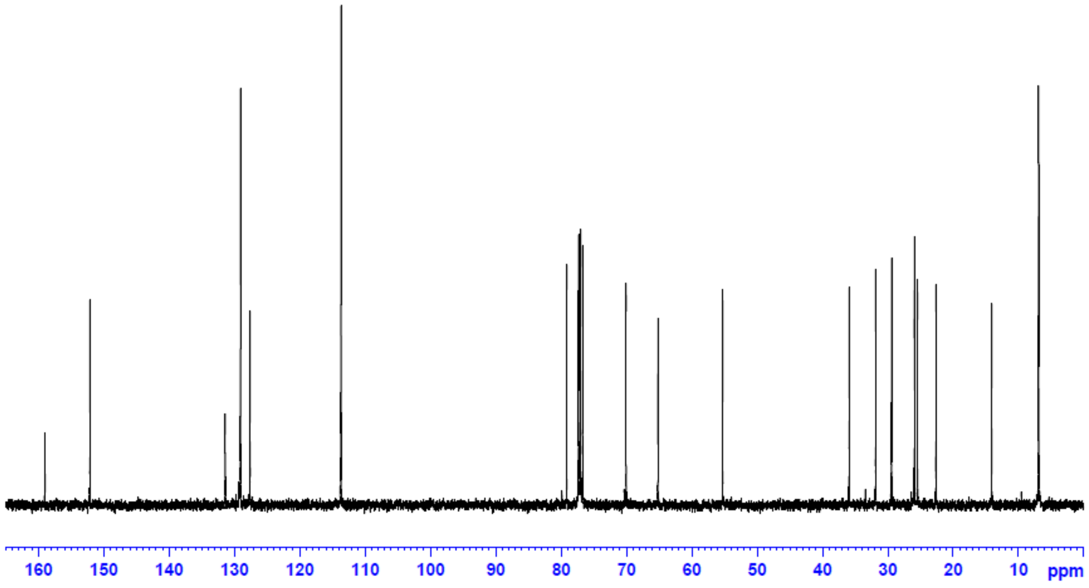

5-Cyclohexyl-2,2-diethyl-2,5-dihydro-1,2-oxasilole, 2d

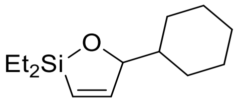

^{1}H (400 MHz, $CDCl_3$)

^{13}C (125 MHz, $CDCl_3$)

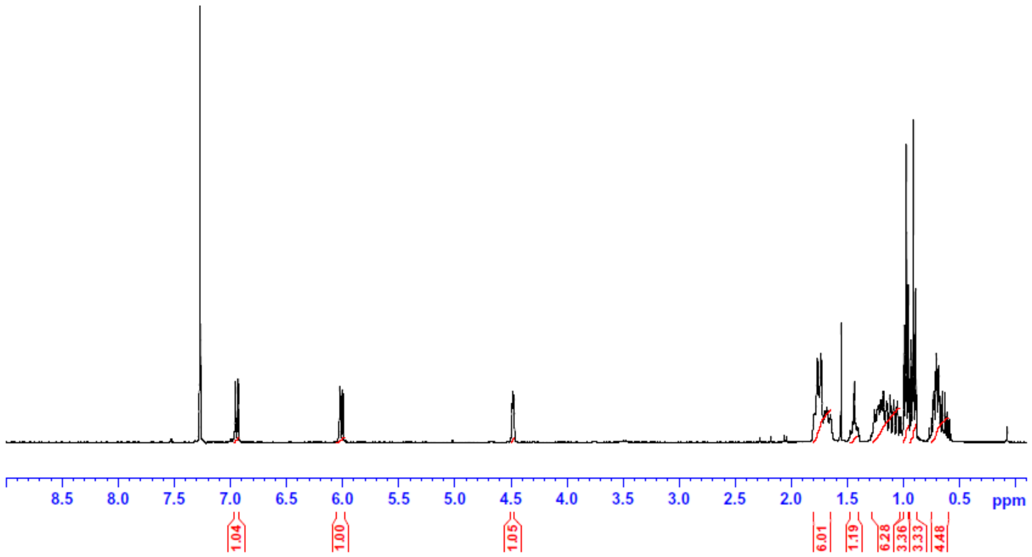

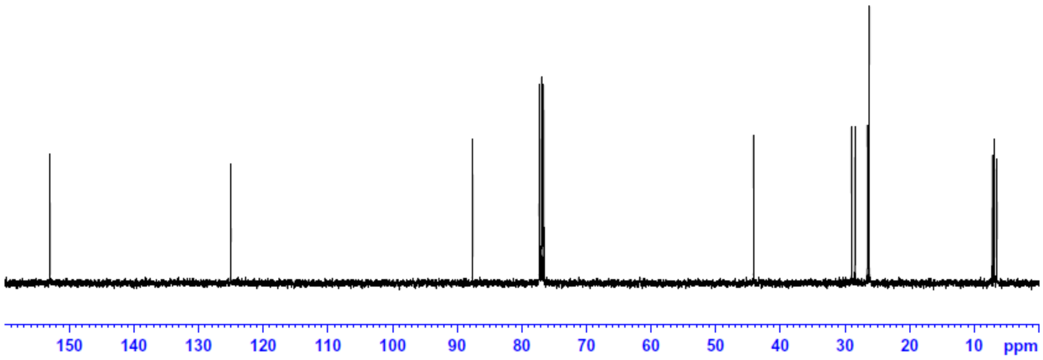

5-(2-((*Tert*-butyldimethylsilyl)oxy)ethyl)-2,2-diethyl-2,5-dihydro-1,2-oxasilole, 2e

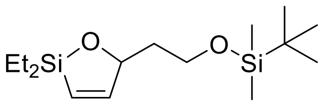

^{1}H (400 MHz, $CDCl_3$)

^{13}C (125 MHz, $CDCl_3$)

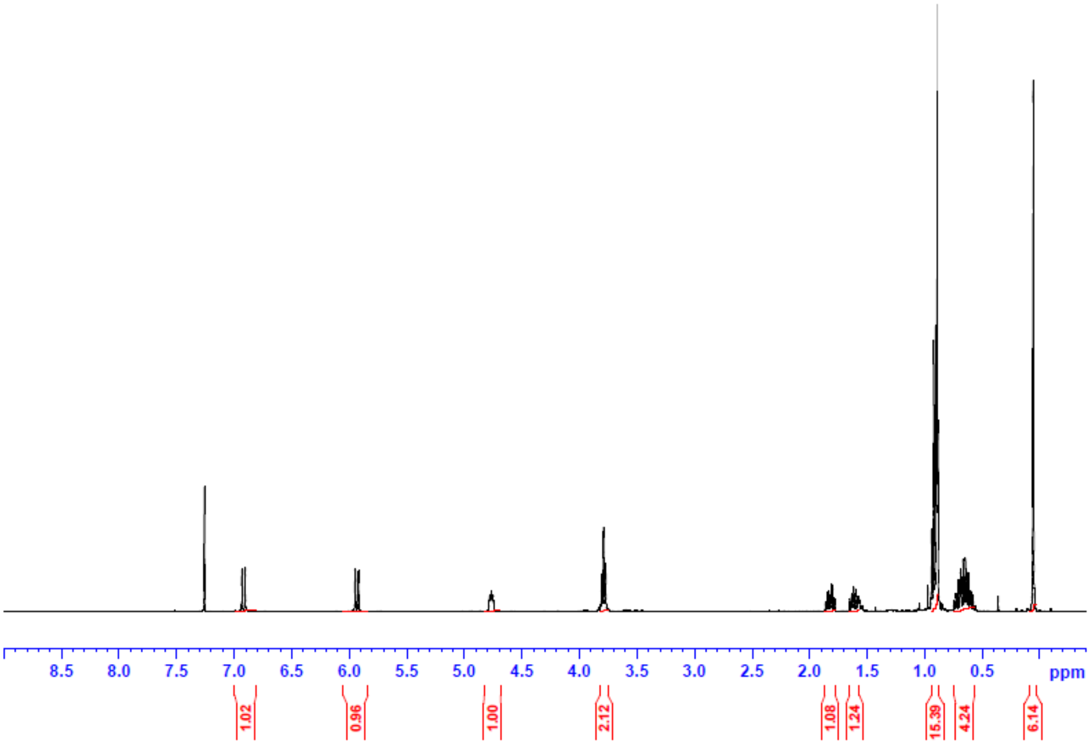

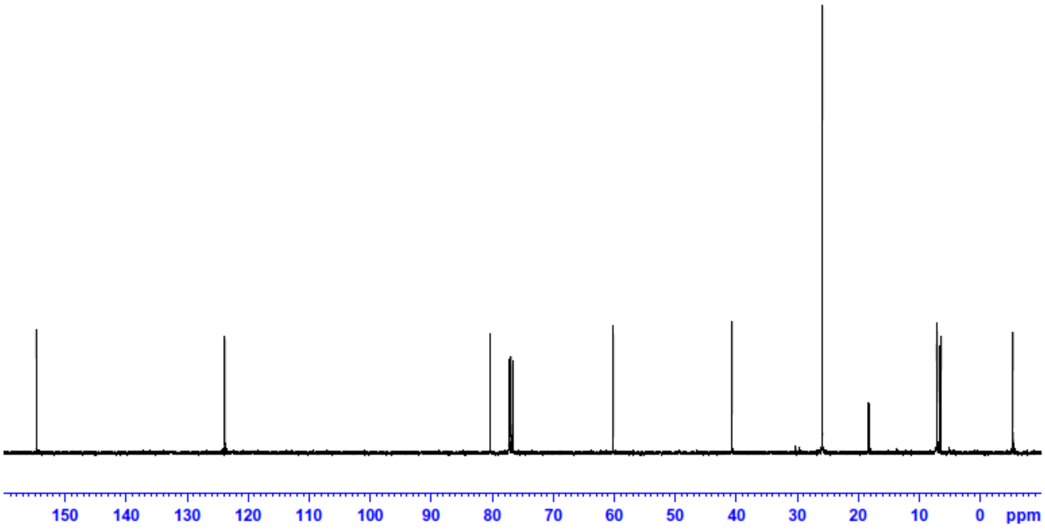

2,2-diethyl-5-(2-((4-methoxybenzyl)oxy)ethyl)-2,5-dihydro-1,2-oxasilole, 2f

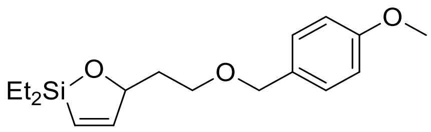

^{1}H (400 MHz, $CDCl_3$)

^{13}C (100 MHz, $CDCl_3$)

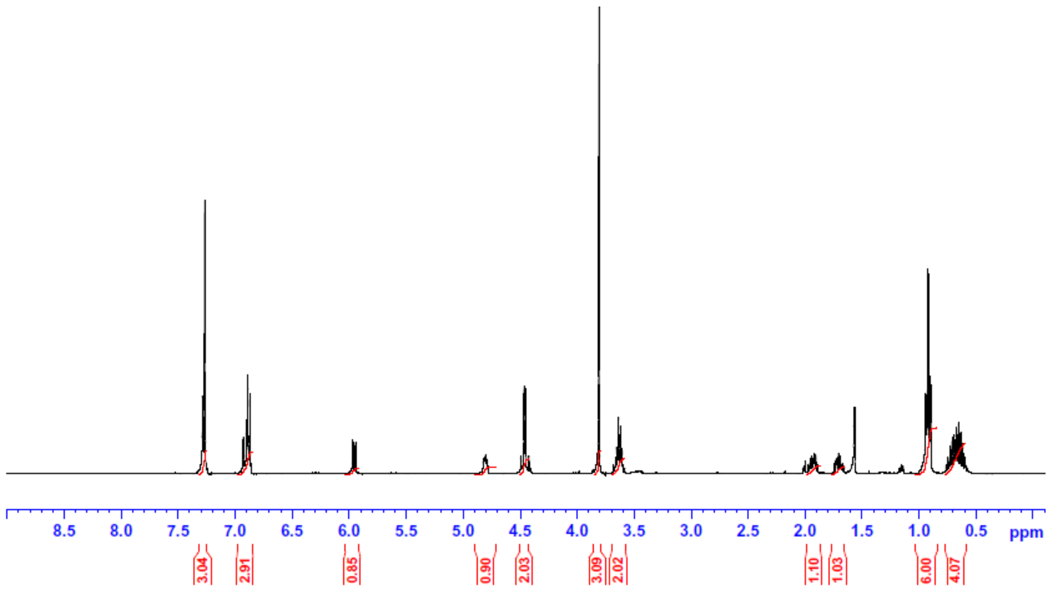

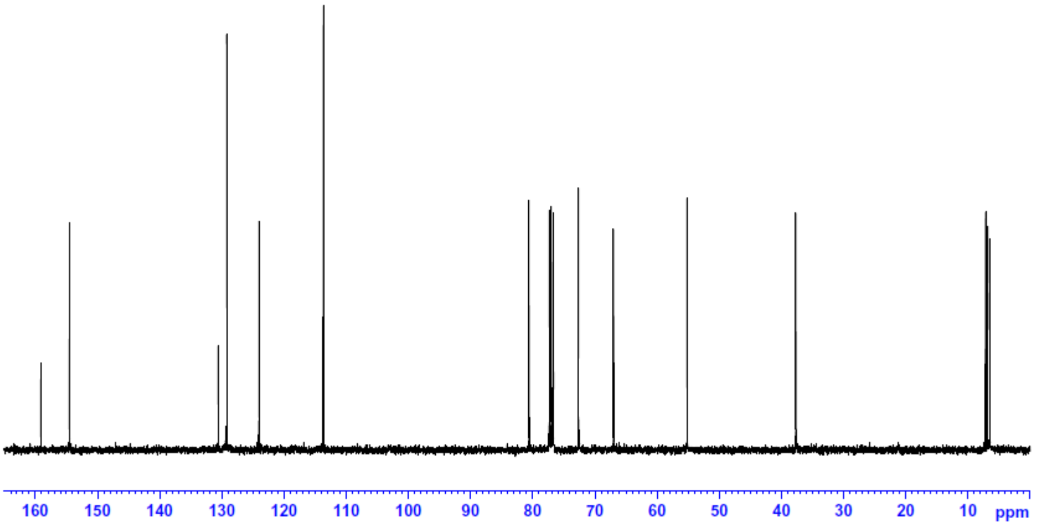

2,2,2-Diethyl-5-phenyl-2,5-dihydro-1,2-oxasilole, 2g

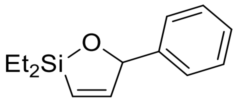

^{1}H (400 MHz, $CDCl_3$)

^{13}C (125 MHz, $CDCl_3$)

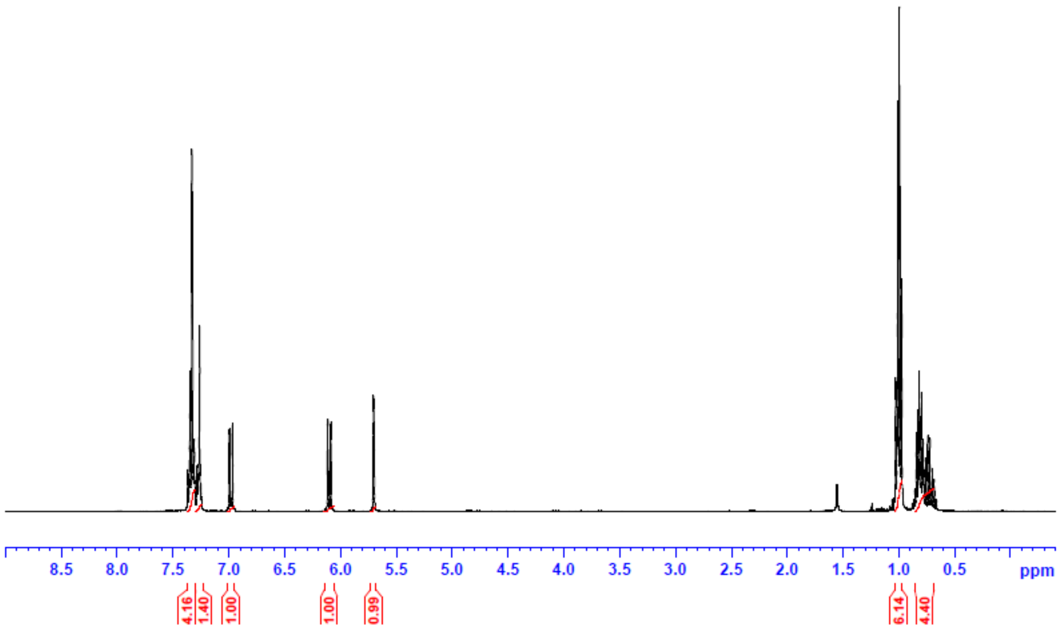

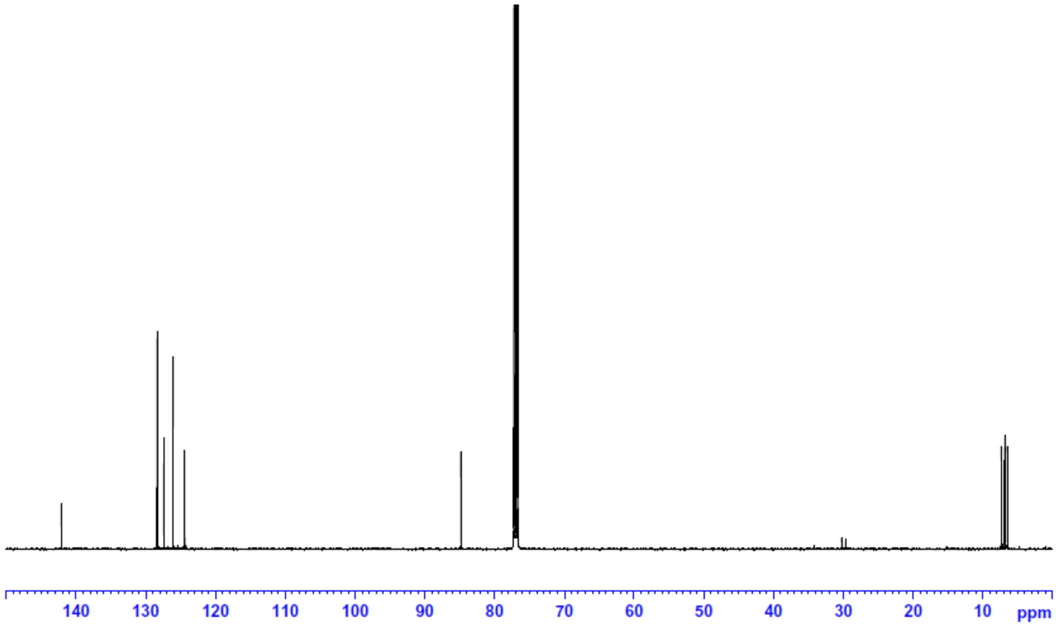

2-(2,2-Diethyl-2,5-dihydro-1,2-oxasilol-5-yl)propane-1,2-diol, 1h

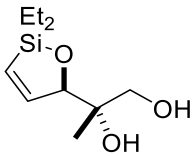

^1H (400 MHz, $CDCl_3$)

^{13}C (125 MHz, $CDCl_3$)

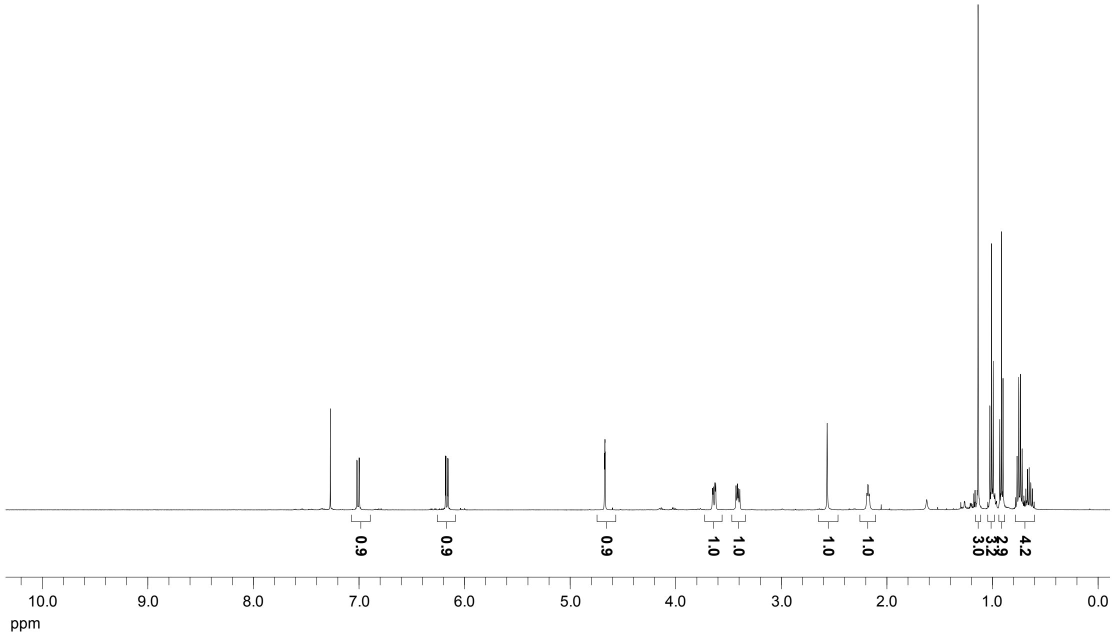

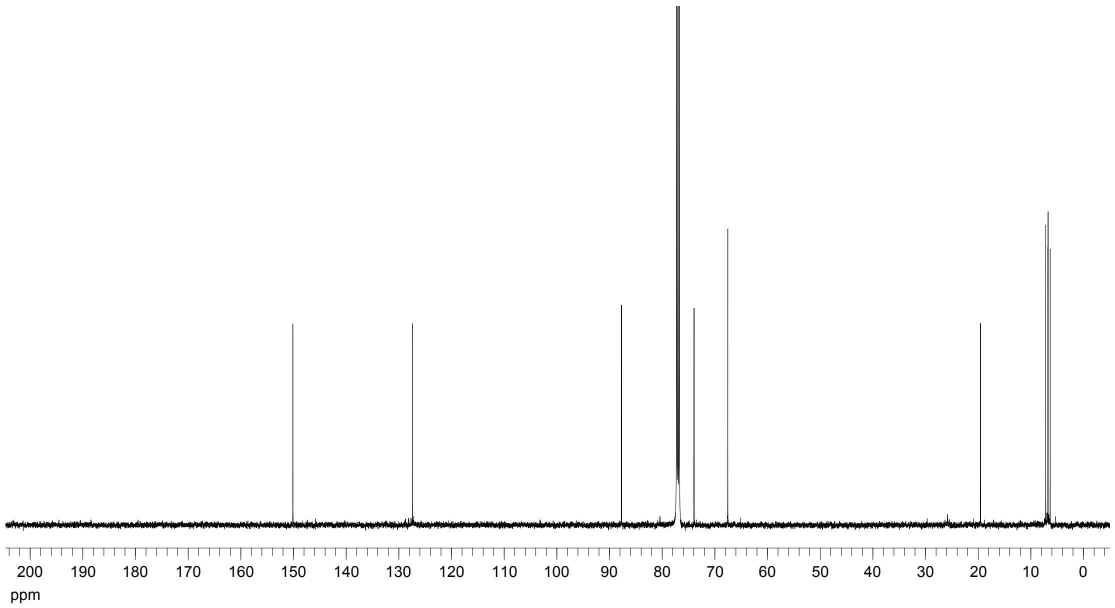

(5*S*,6*R*)-2,2-Diethyl-6-[hydroxymethyl]-6-methyl-5,6-dihydro-2*H*-1,2-oxasilin-5-yl benzoate, 1i

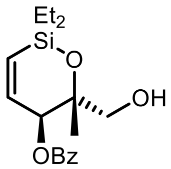

^{1}H (400 MHz, $CDCl_3$)

^{13}C (125 MHz, $CDCl_3$)

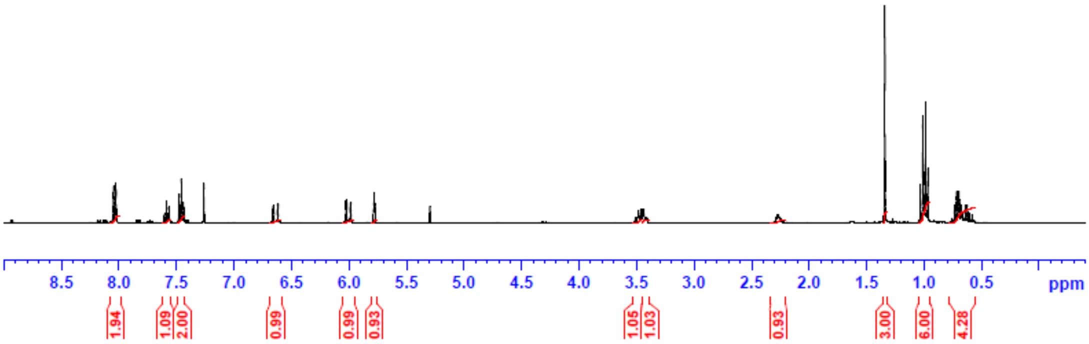

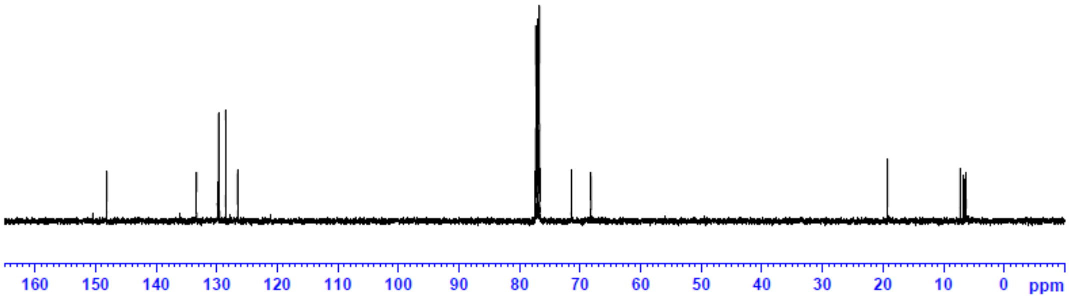

1-(2,2-Diethyl-2,5-dihydro-1,2-oxasilol-5-yl)-3-((4-methoxybenzyl)oxy)propan-1-ol, 1j

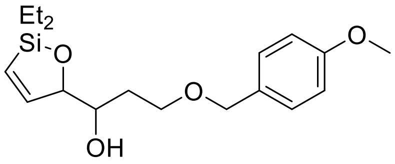

^{1}H (500 MHz, $CDCl_3$)

^{13}C (125 MHz, $CDCl_3$)

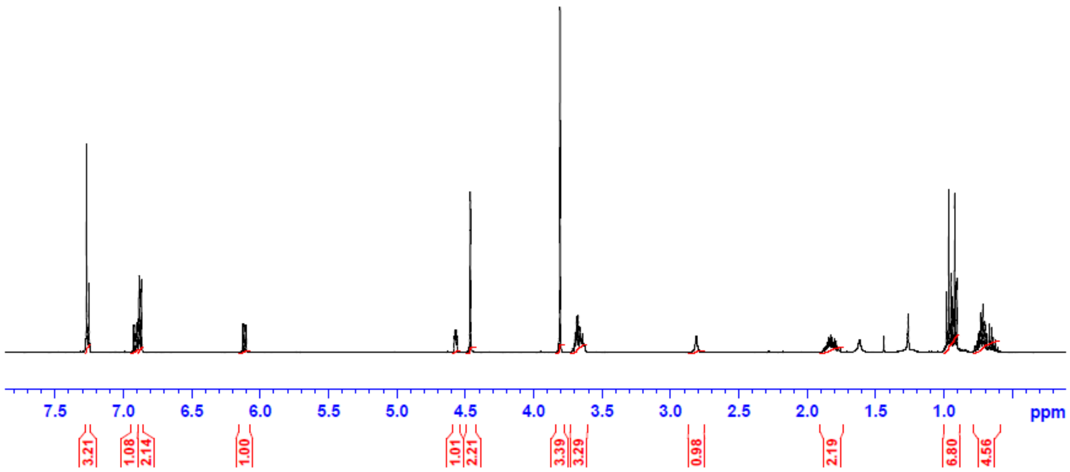

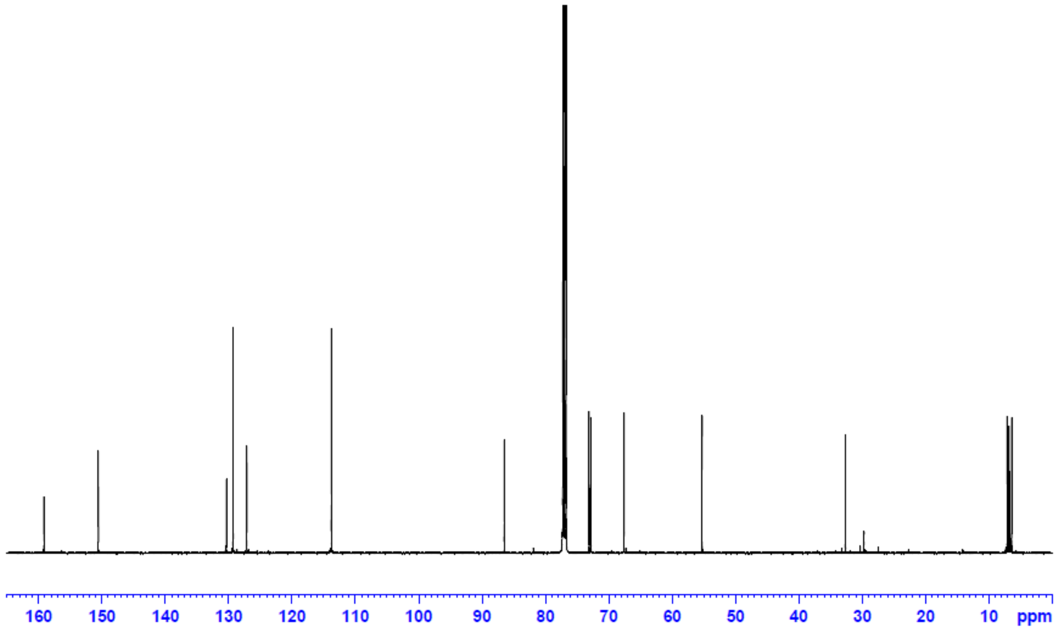

2,2-Diethyl-1-oxa-2-silaspiro[4.5]dec-3-ene, 1k

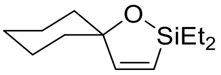

^{1}H (400 MHz, $CDCl_3$)

^{13}C (100 MHz, $CDCl_3$)

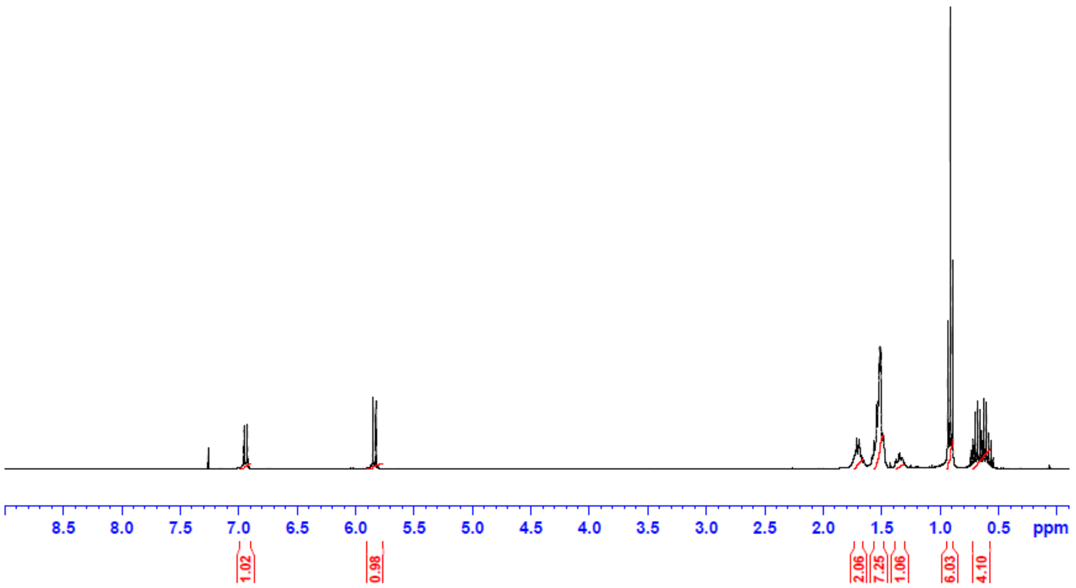

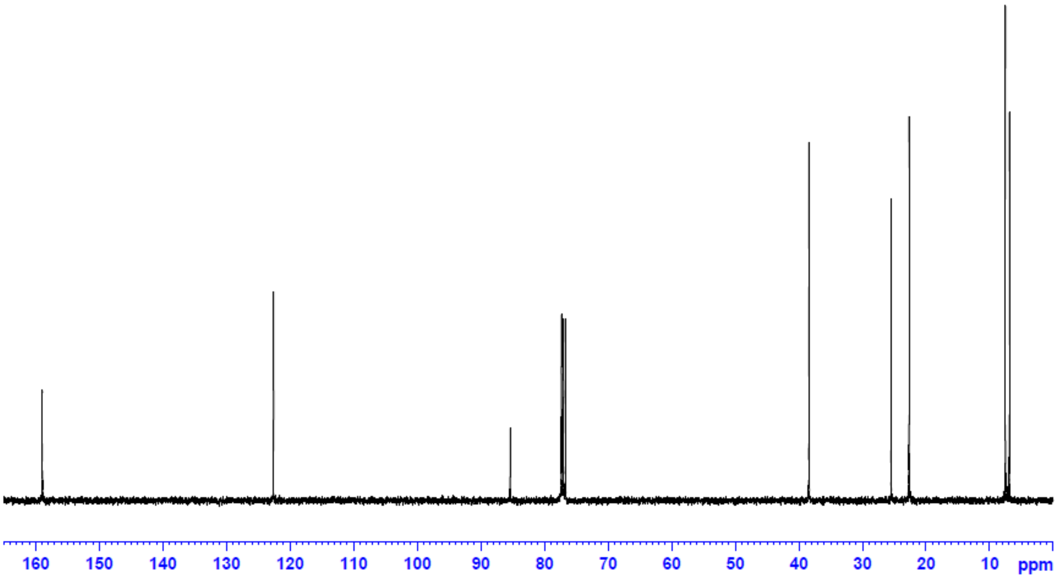

2,2-Diethyl-6-hexyl-5,6-dihydro-2H-1,2-oxasiline, 1l

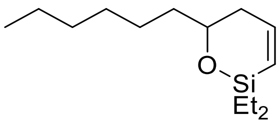

^{1}H (400 MHz, $CDCl_3$)

^{13}C (100 MHz, $CDCl_3$)

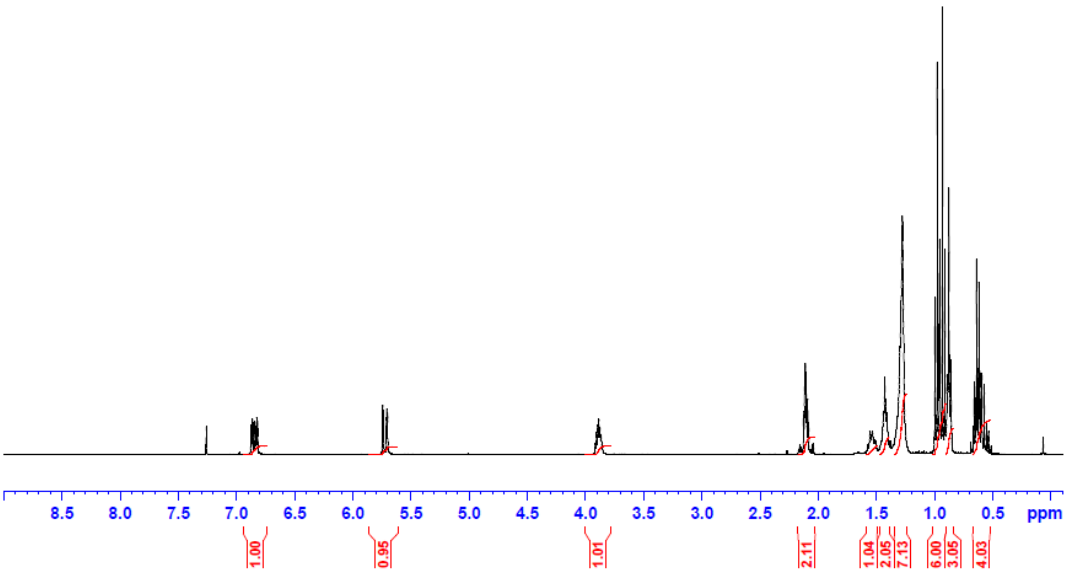

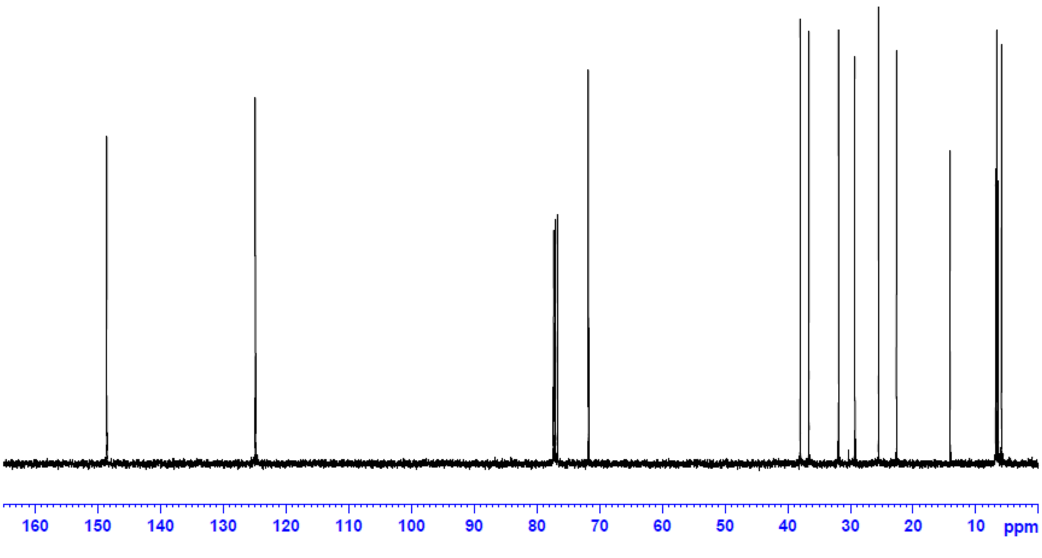

6-Cyclohexyl-2,2-diethyl-5,6-dihydro-2H-1,2-oxasiline, 1m

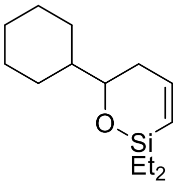

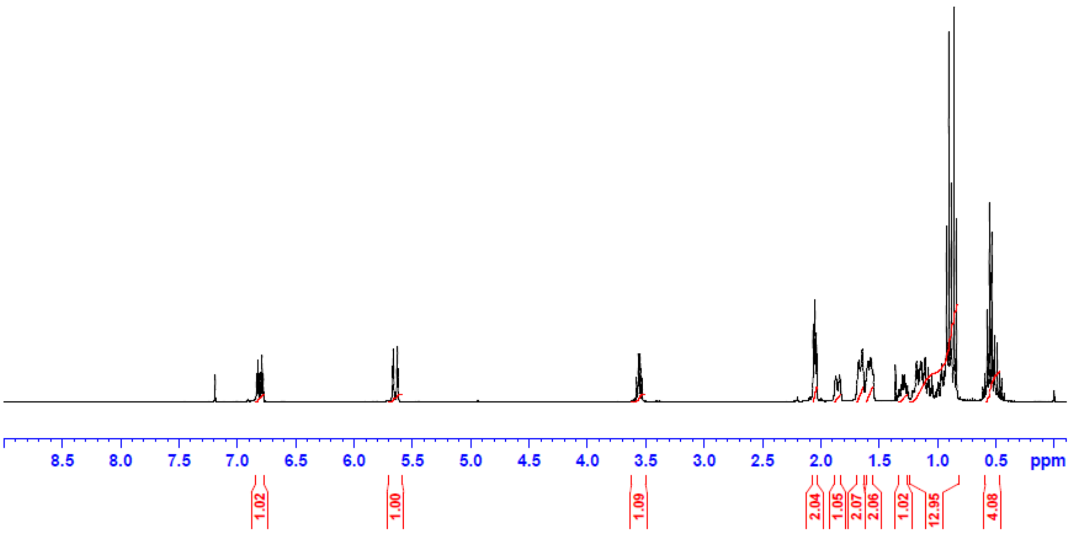

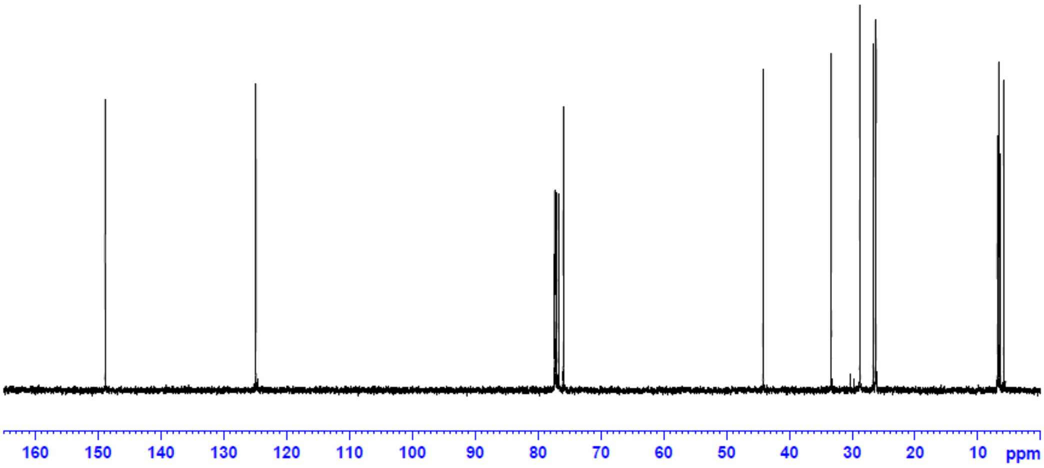

6-(2-((*Tert*-butyldimethylsilyl)oxy)ethyl)-2,2-diethyl-5,6-dihydro-2H-1,2-oxasiline, 1n

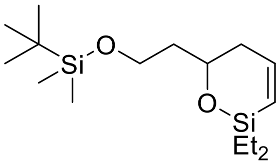

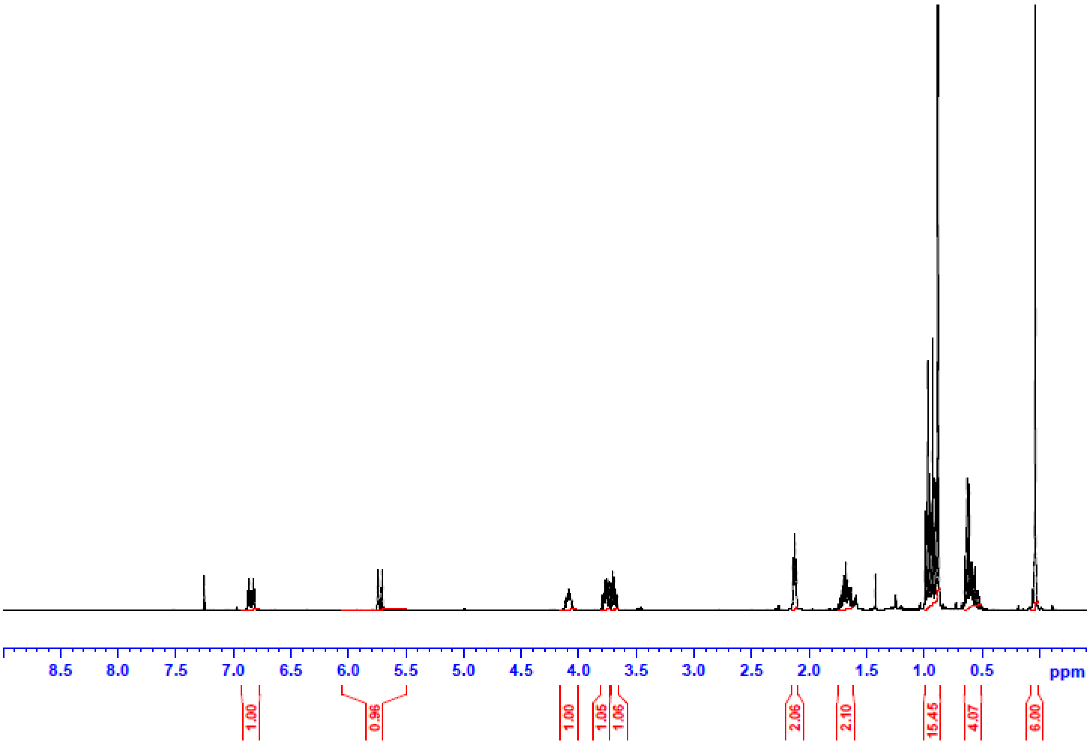

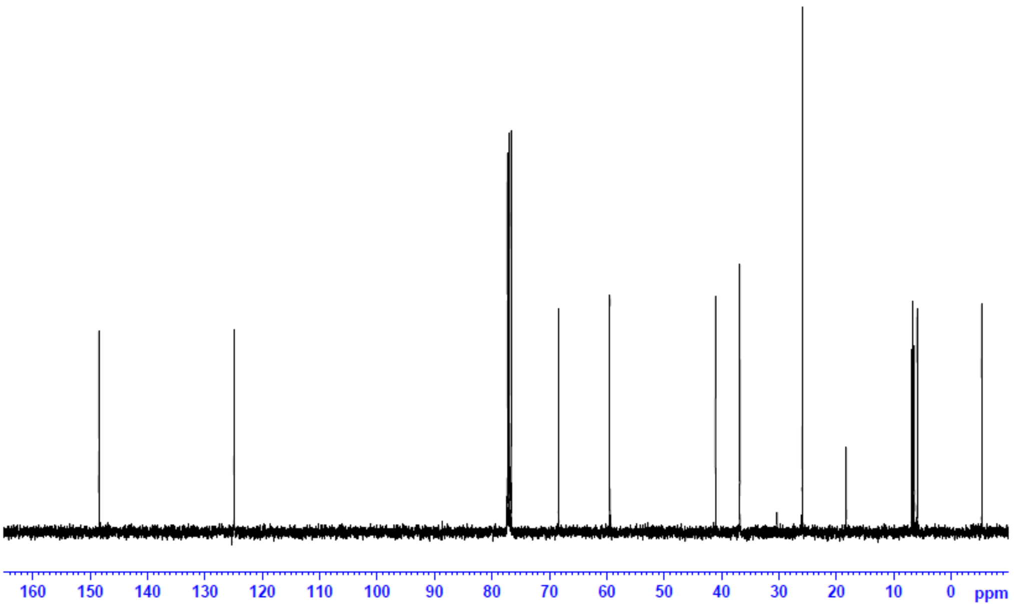

2,2-Diethyl-6-(2-((4-methoxybenzyl)oxy)ethyl)-5,6-dihydro-2H-1,2-oxasiline, 1o

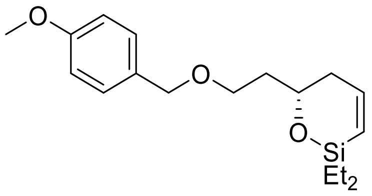

^{1}H (400 MHz, $CDCl_3$)

^{13}C (100 MHz, $CDCl_3$)

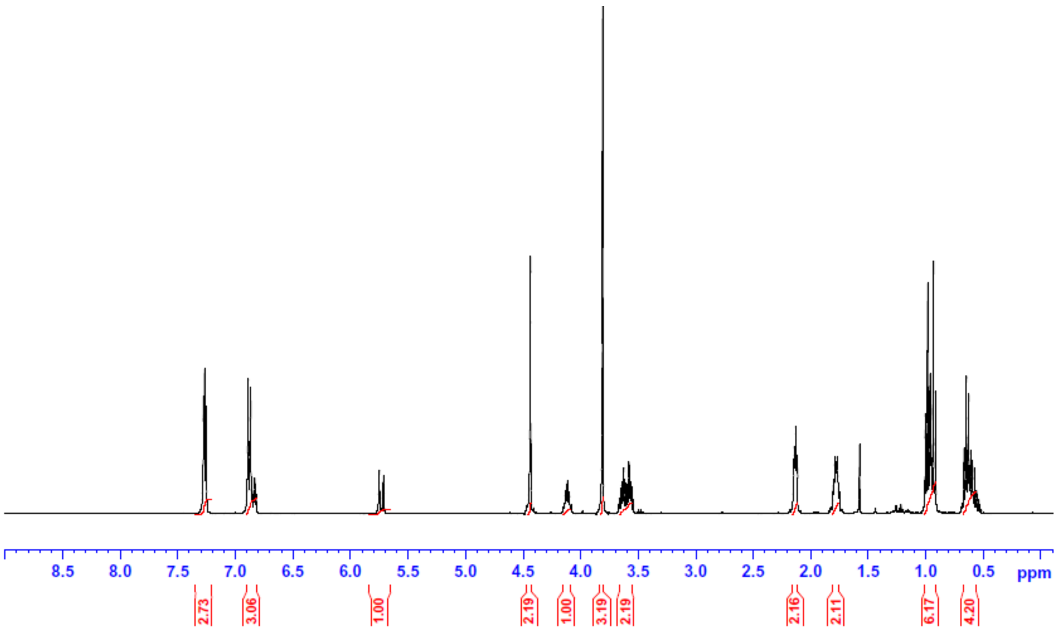

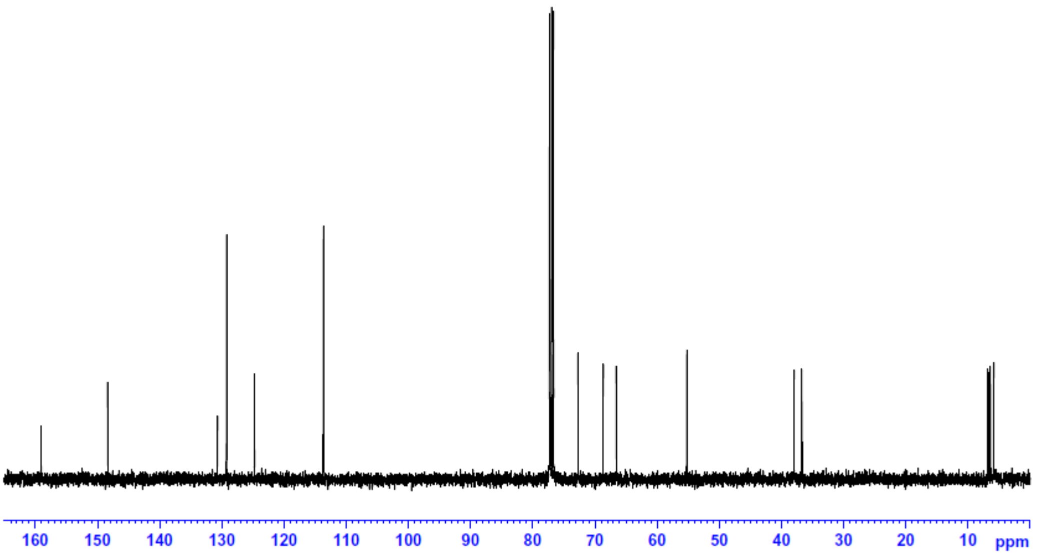

2,2-Diethyl-4a,5,6,7,8,8a-hexahydro-2H-benzo[e][1,2]oxasiline, 1p

^{1}H (500 MHz, $CDCl_3$)

^{13}C (125 MHz, $CDCl_3$)

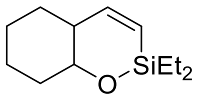

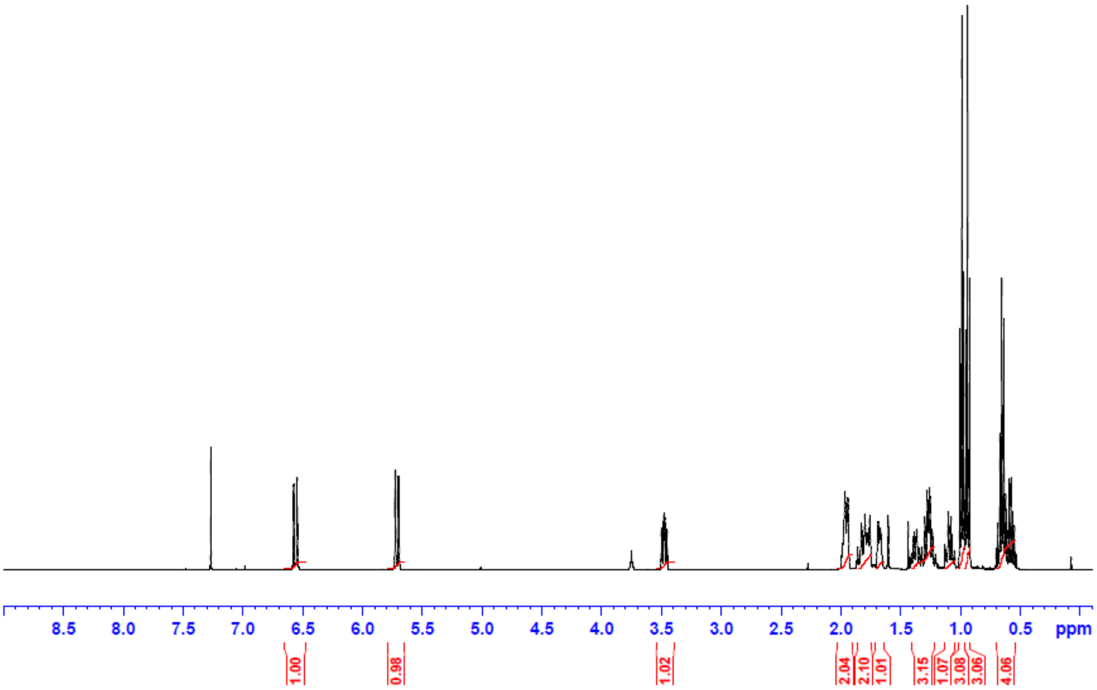

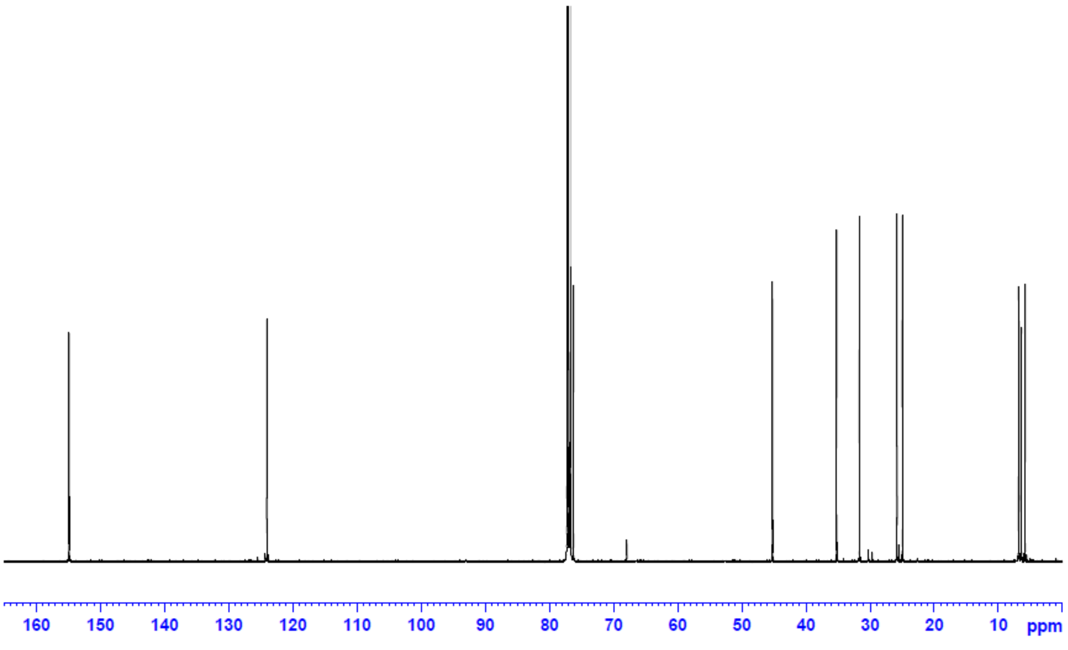

(*Z*)-2-Chloro-3-(2-iodovinyl)phenol, 4g-S2

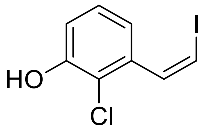

^{1}H (400 MHz, $CDCl_3$)

^{13}C (100 MHz, $CDCl_3$)

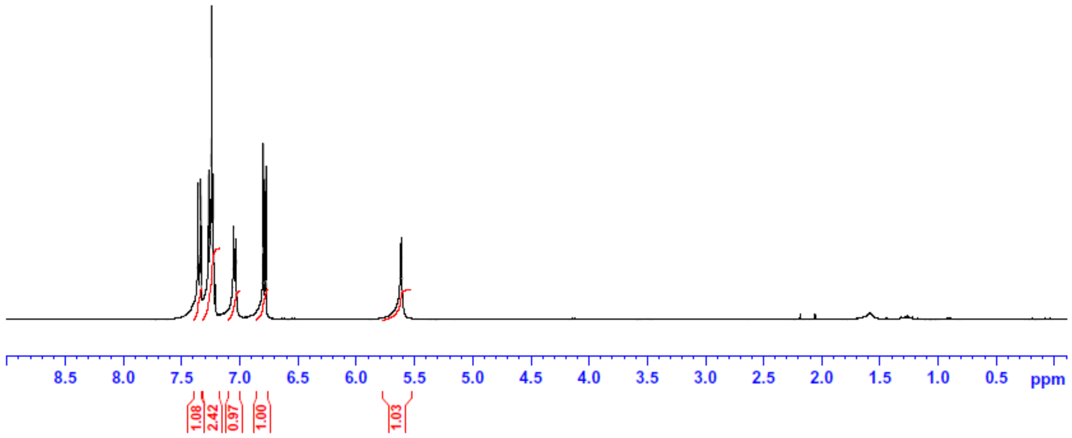

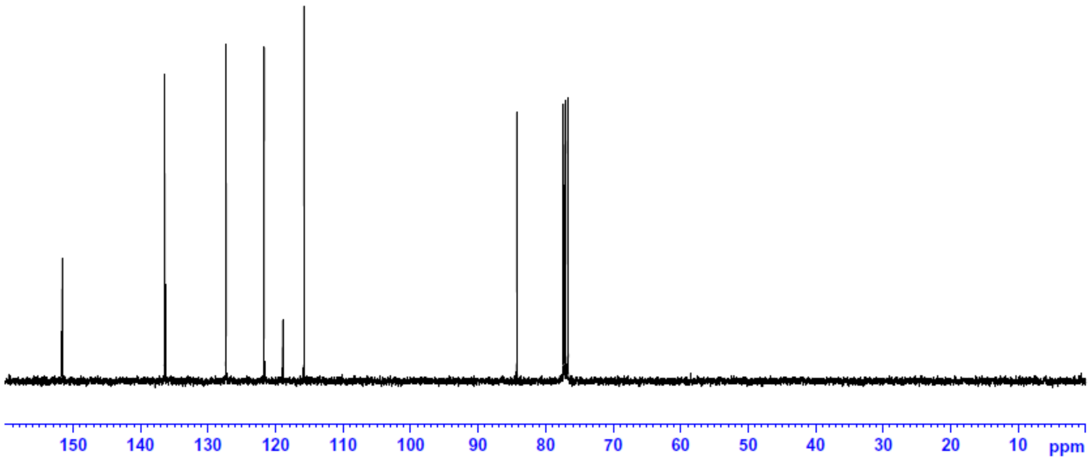

2-Chloro-3-ethynylphenol, 4g-S3

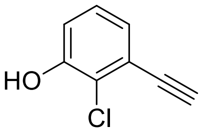

^{1}H (400 MHz, $CDCl_3$)

^{13}C (100 MHz, $CDCl_3$)

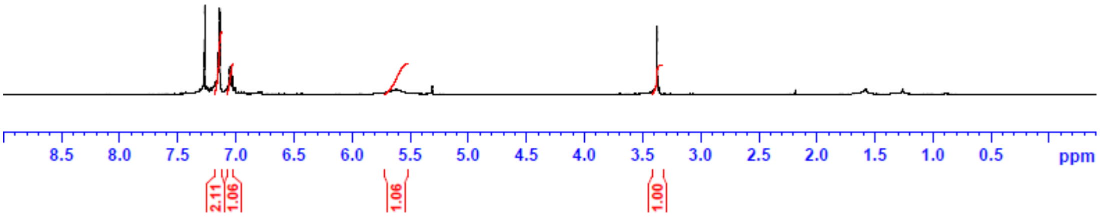

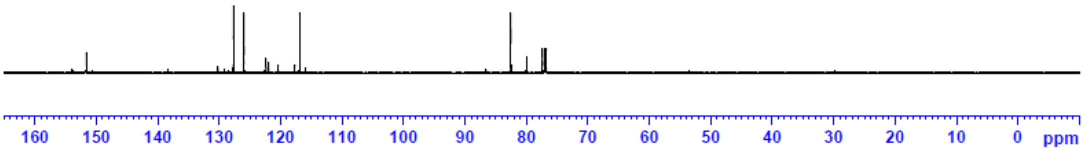

(*E*)-2-Chloro-3-(2-iodovinyl)phenol, 4g

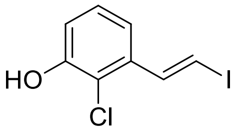

^{1}H (400 MHz, $CDCl_3$)

^{13}C (100 MHz, $CDCl_3$)

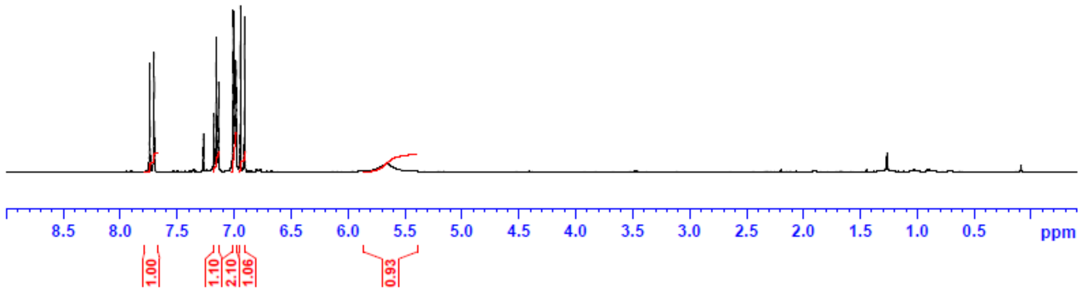

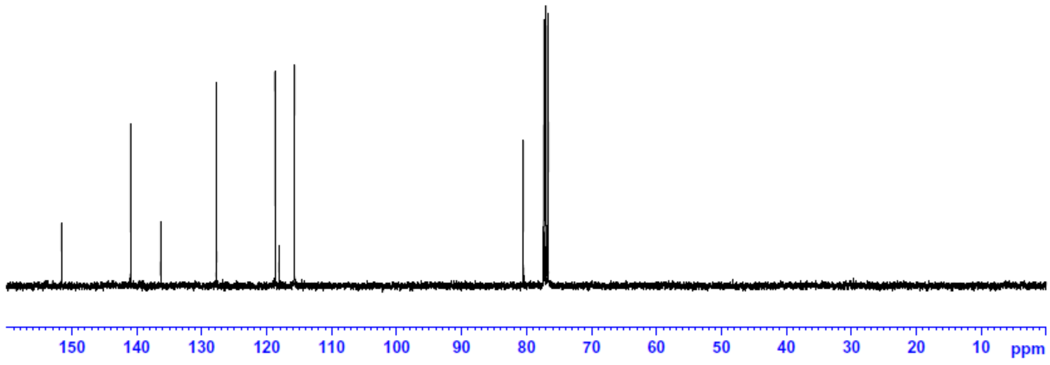

(1*E*,3*Z*)-1-Phenylundeca-1,3-dien-5-ol, 5aa

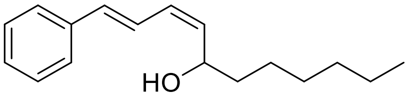

^{1}H (400 MHz, C_6D_6)

^{13}C (101 MHz, C_6D_6)

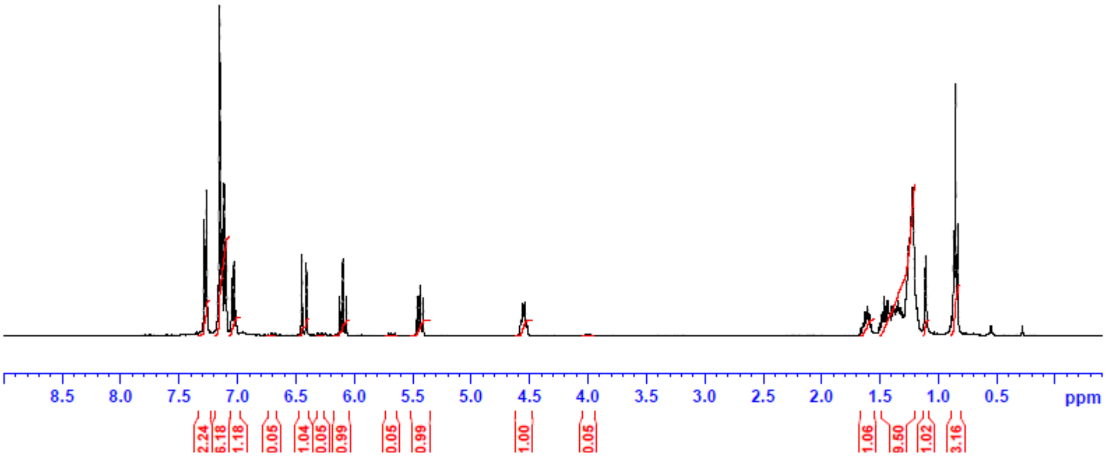

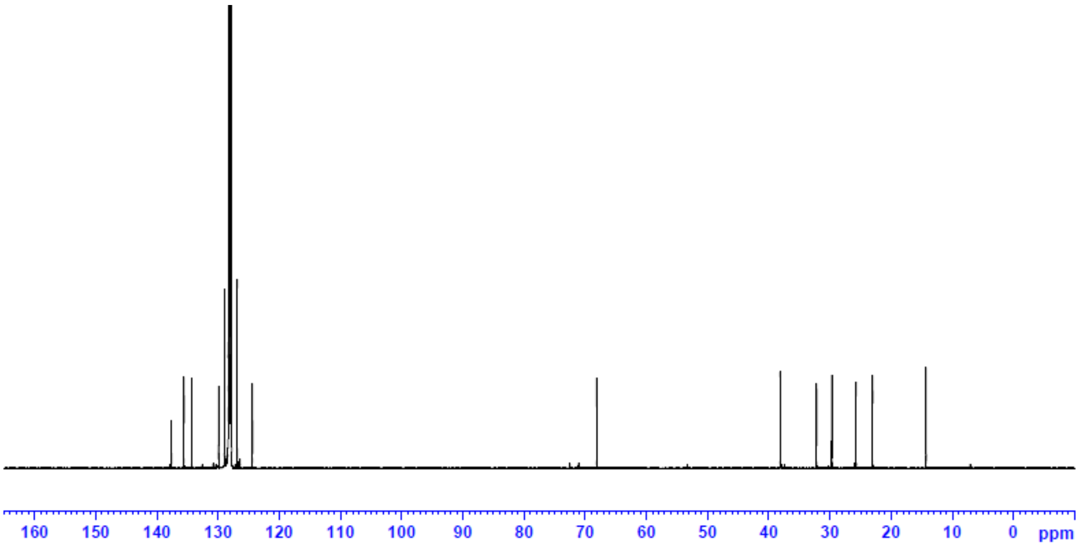

(8*Z*,10*Z*)-Octadeca-8,10-diene–7,12-diol

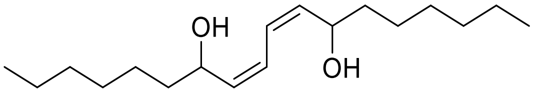

^{1}H (400 MHz, C_6D_6)

^{13}C (101 MHz, C_6D_6)

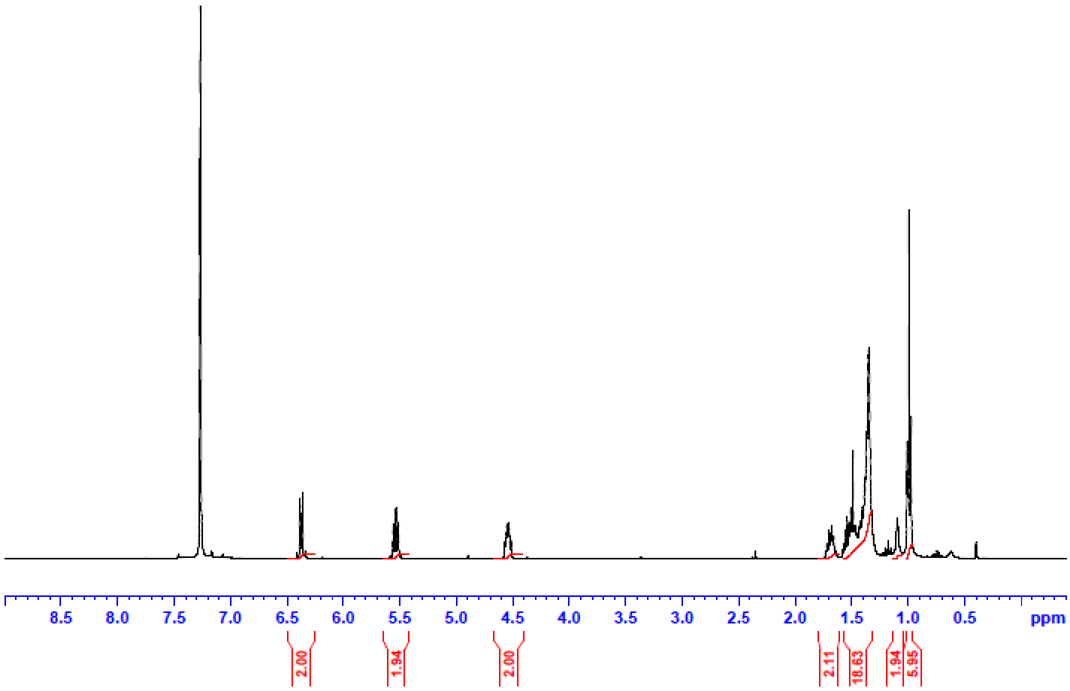

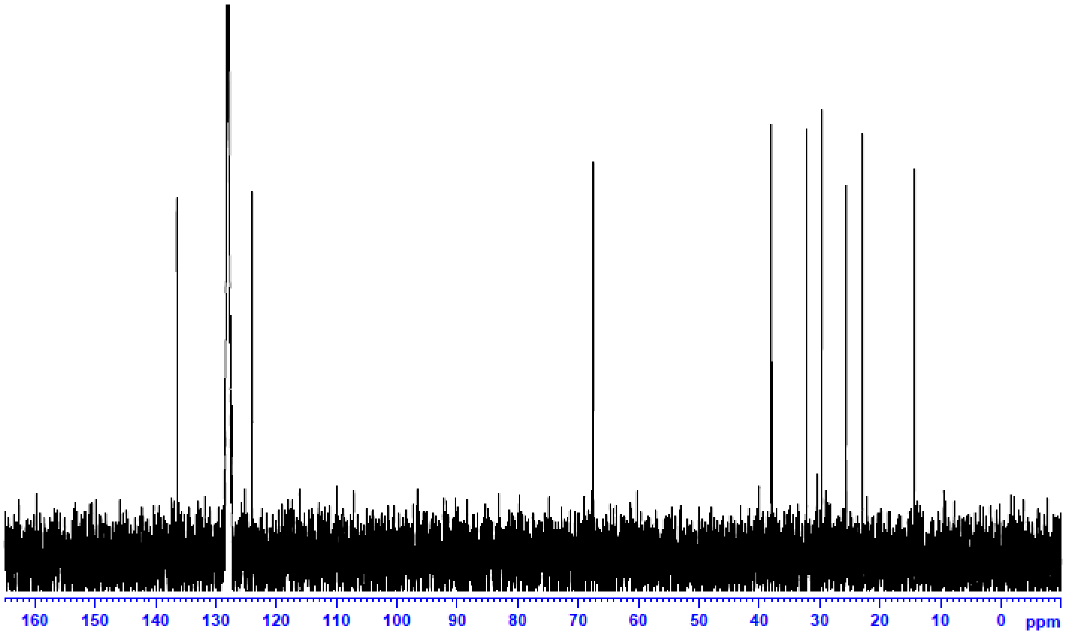

(*Z*)-1-Phenylnon-1-en-3-ol, 5ba

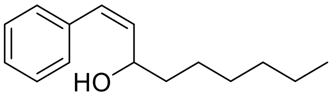

^{1}H (400 MHz, $CDCl_3$)

^{13}C (100 MHz, $CDCl_3$)

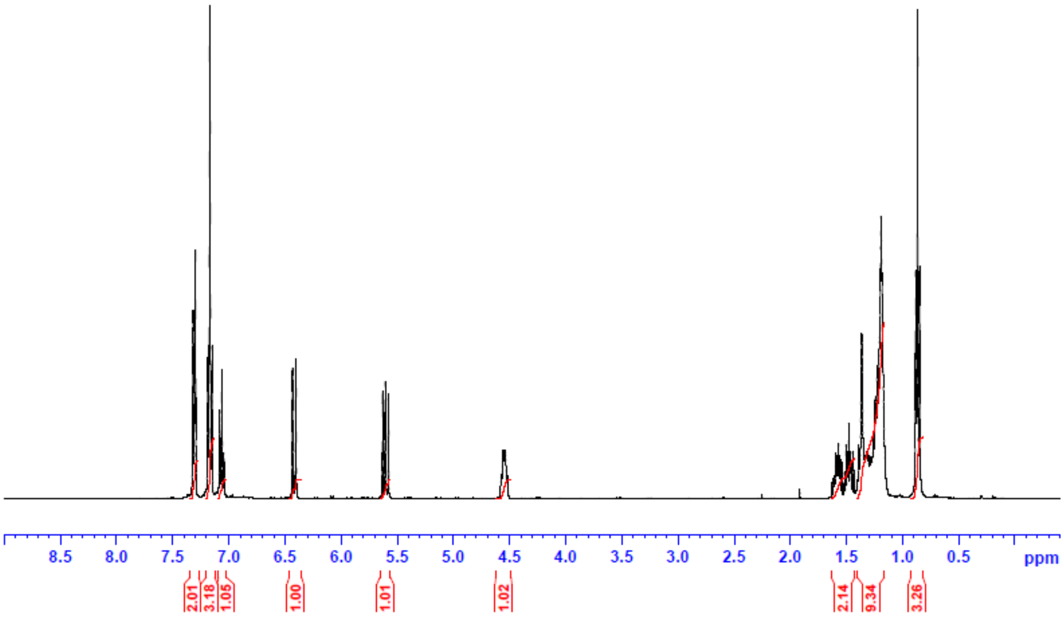

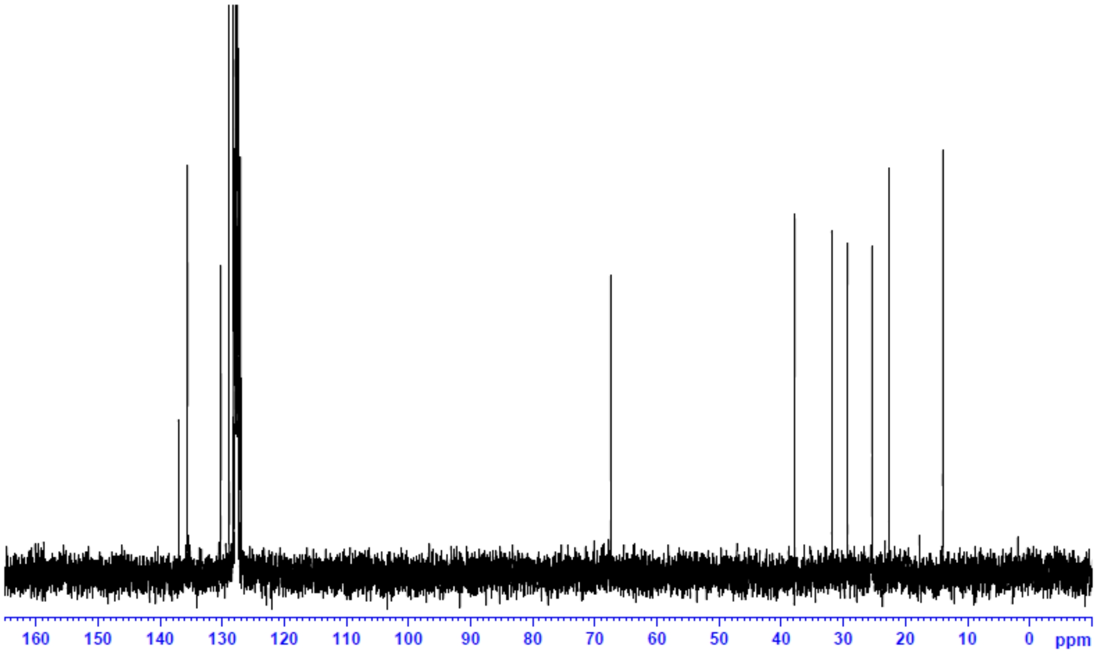

(*Z*)-1-Cyclohexyl-3-phenylprop-2-en-1-ol, 5bd

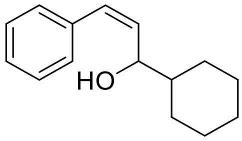

^{1}H (400 MHz, C_6D_6)

^{13}C (100 MHz, C_6D_6)

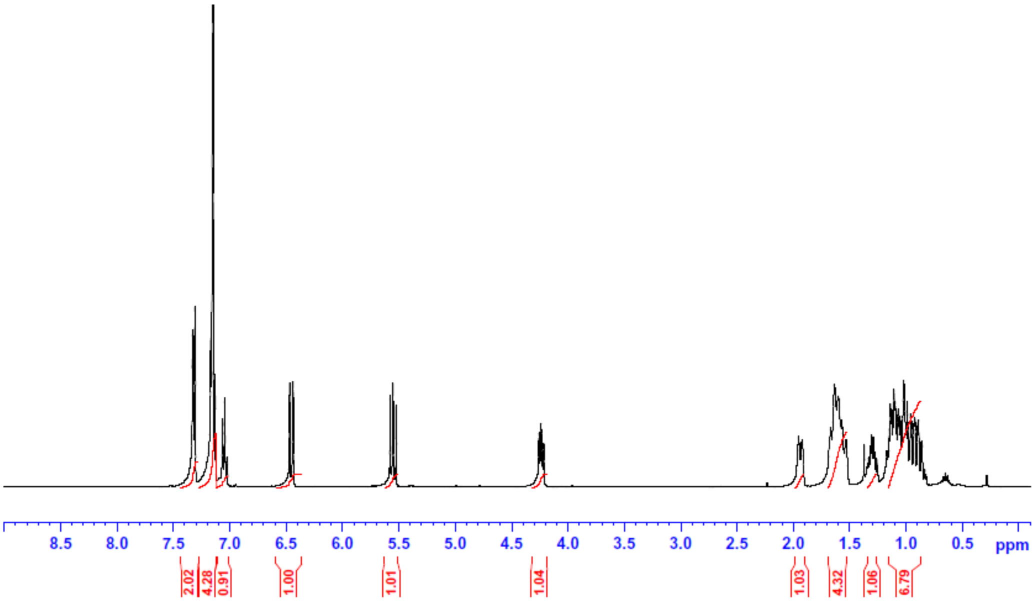

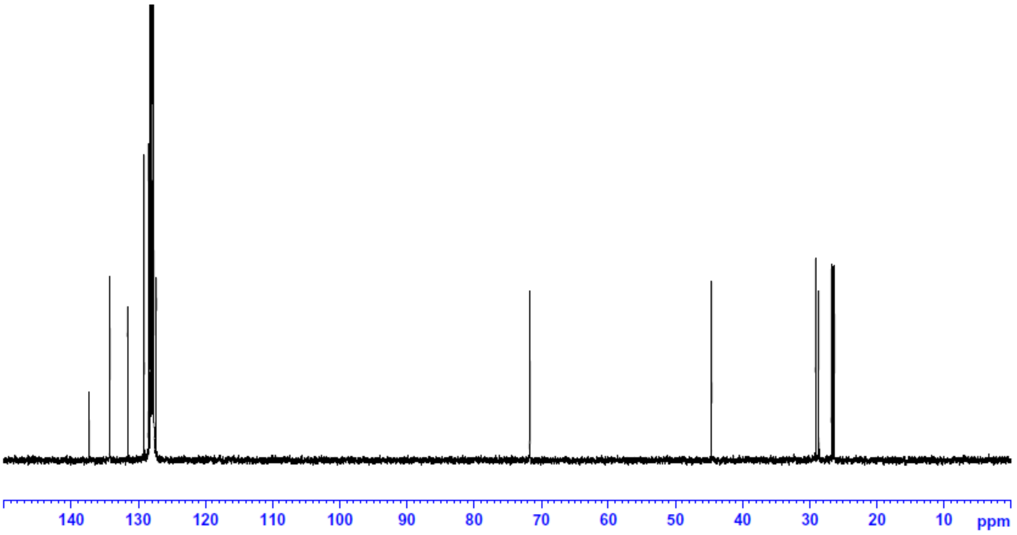

(2*E*,4*Z*)-1-(Benzyloxy)dodeca-2,4-dien-6-ol, 5ca

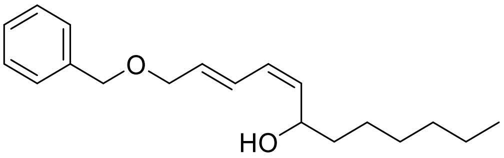

^{1}H (500 MHz, $CDCl_3$)

^{13}C (125 MHz, $CDCl_3$)

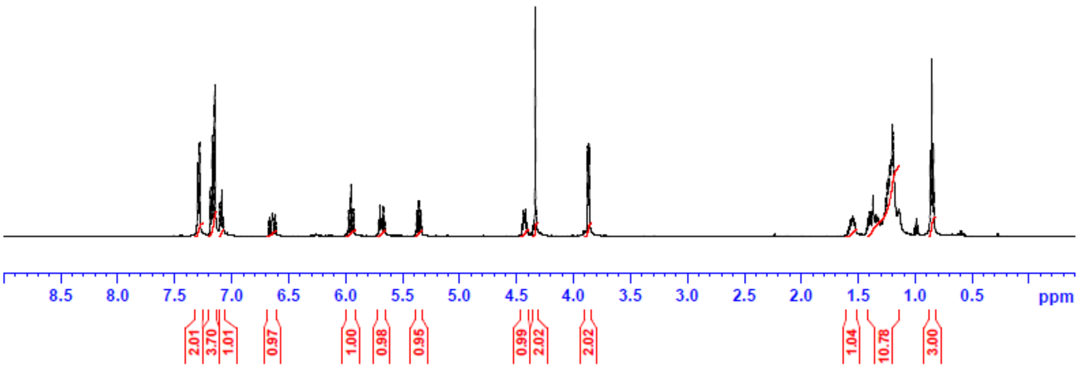

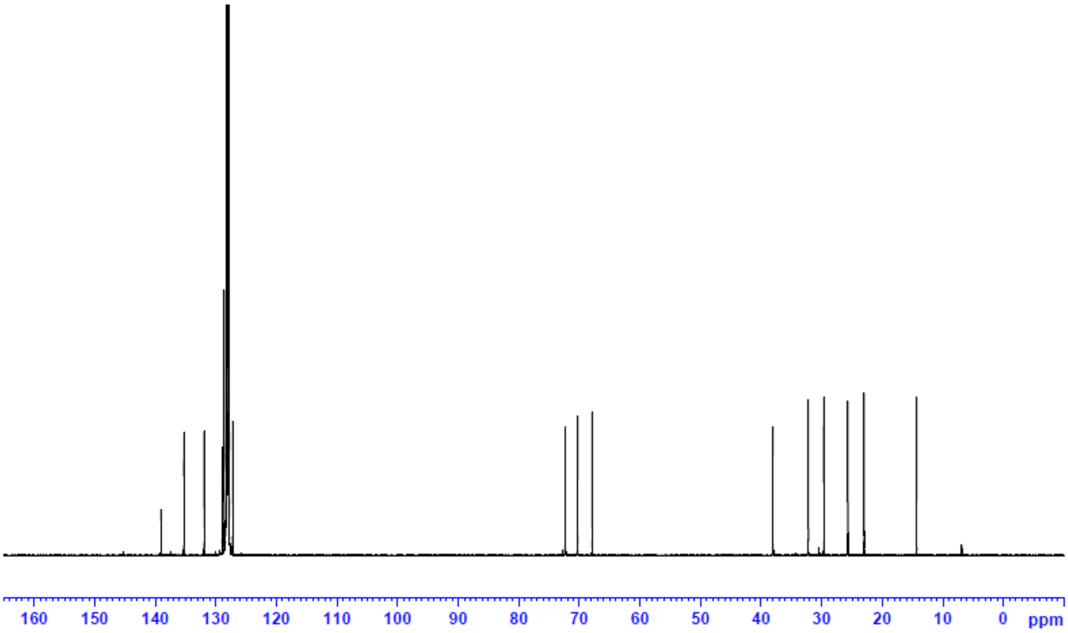

(2*Z*,4*E*)-1-Phenylnona-2,4-dien-1-ol, 5dg

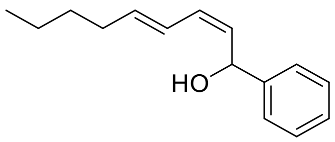

^{1}H (500 MHz, C_6D_6)

^{13}C (125 MHz, C_6D_6)

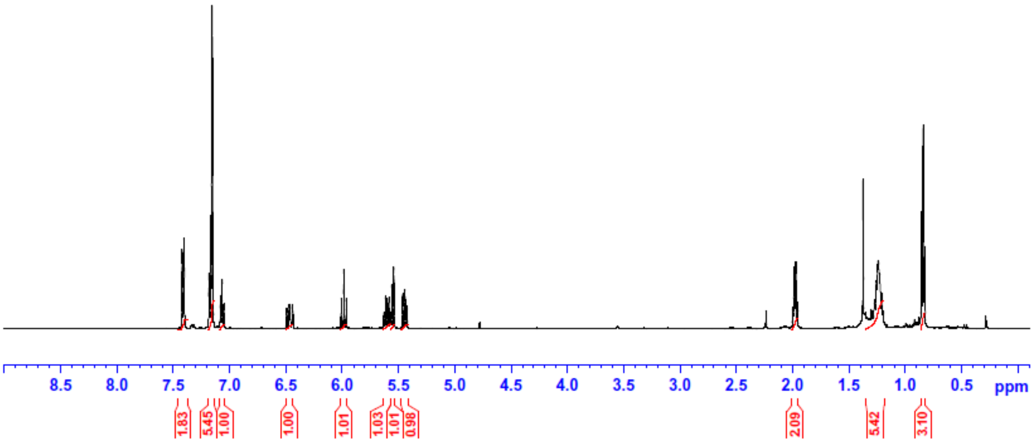

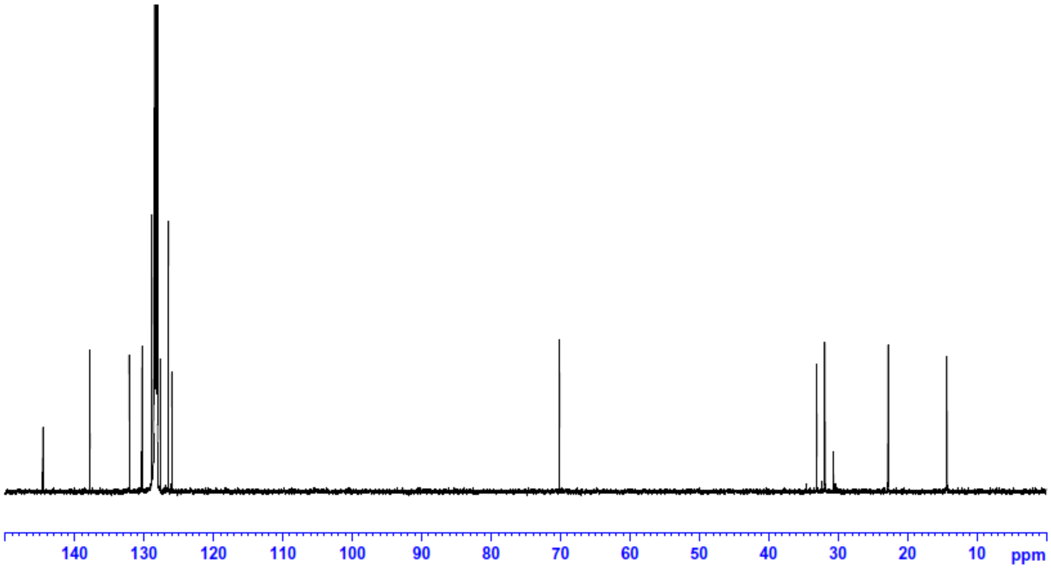

(4*Z*,6*E*)-1-((*Tert*-butyldimethylsilyl)oxy)–7-phenylhepta-4,6-dien-3-ol, 5ae

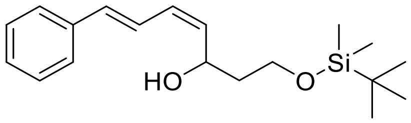

^{1}H (500 MHz, C_6D_6)

^{13}C (125 MHz, C_6D_6)

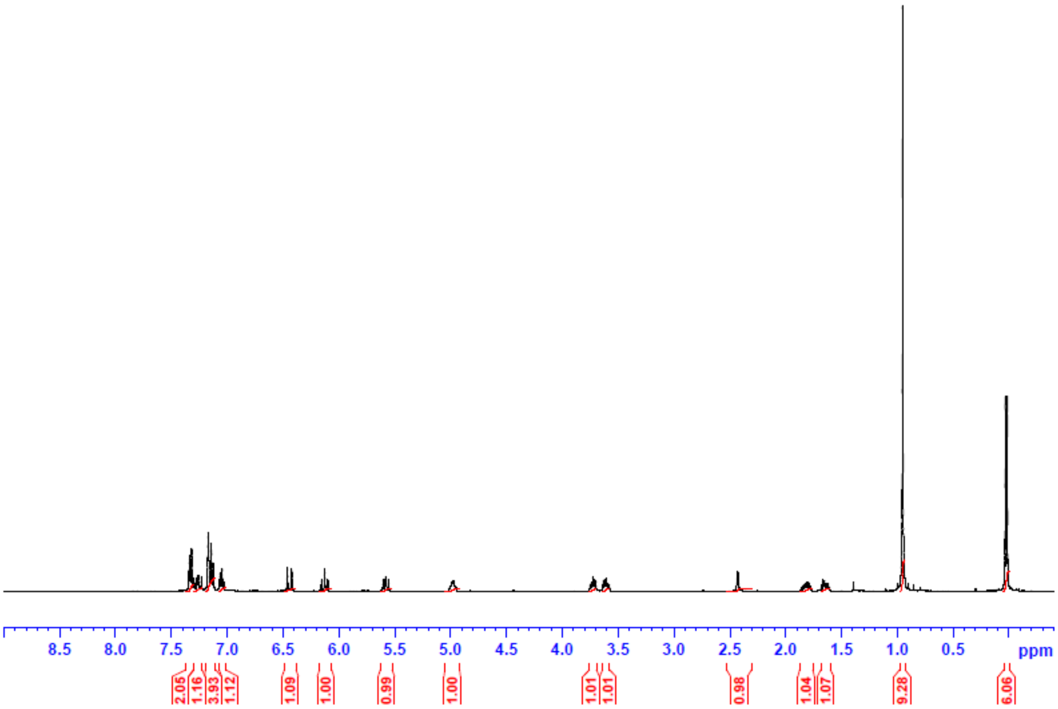

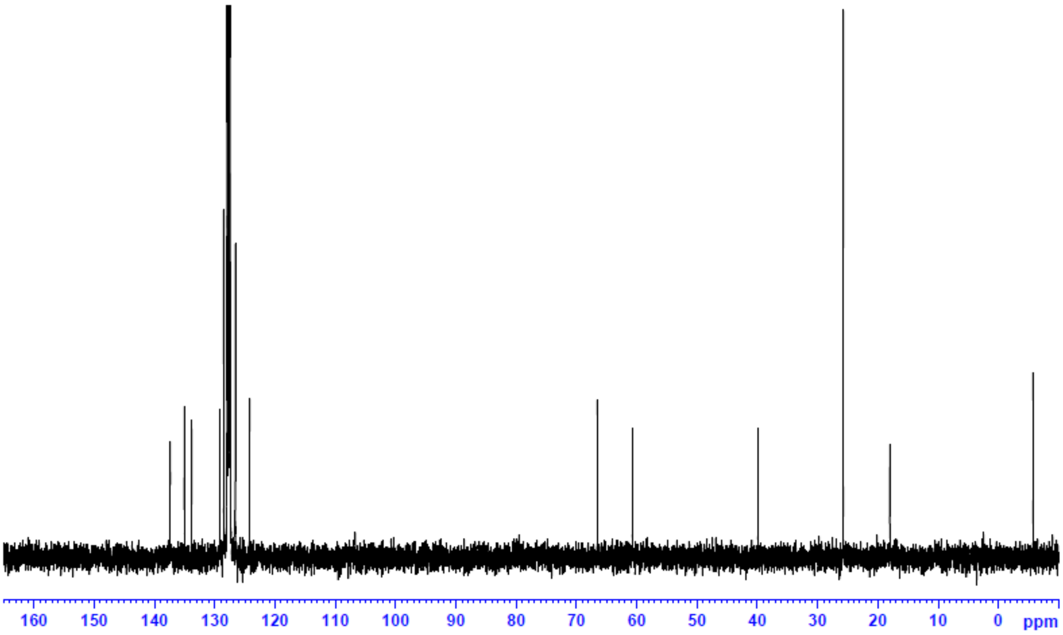

(*Z*)-5-((*Tert*-butyldimethylsilyl)oxy)-1-phenylpent-1-en-3-ol, 5be

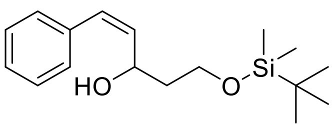

^{1}H (500 MHz, C_6D_6)

^{13}C (125 MHz, C_6D_6)

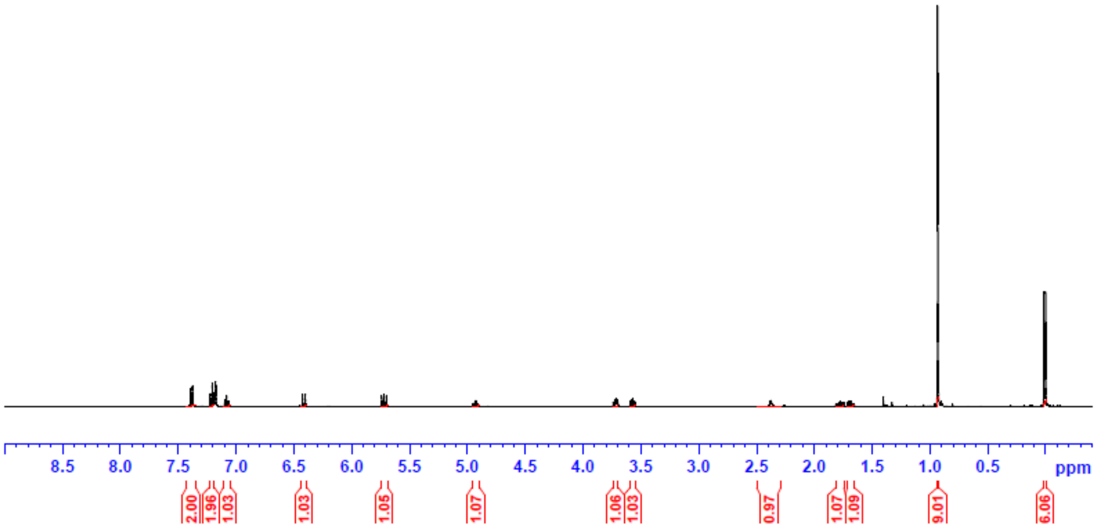

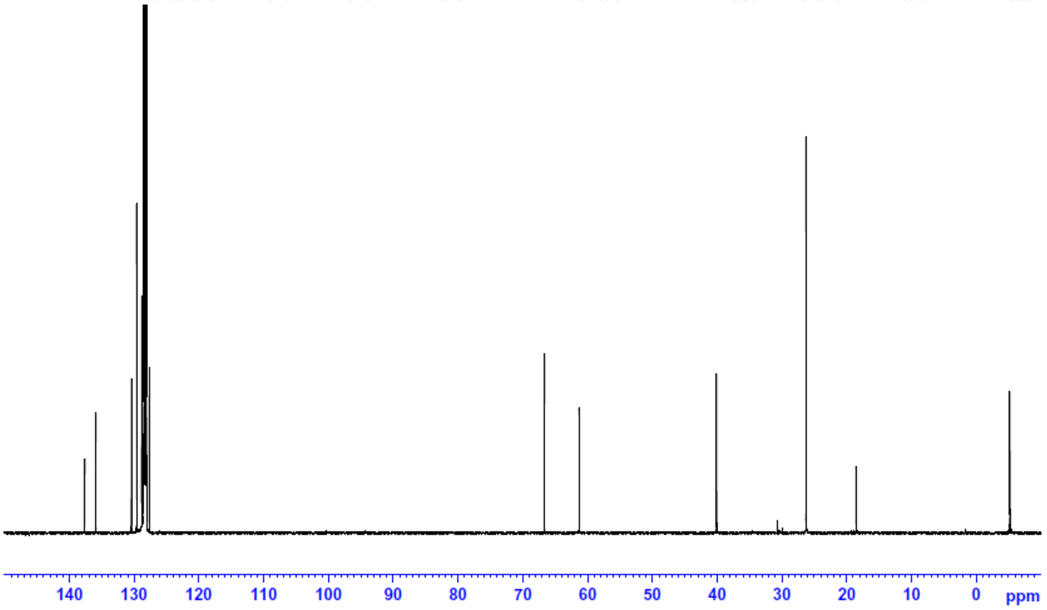

((2*R*,3*S*,4*Z*,6*E*)-2-Methyl–7-phenylhepta-4,6-diene-1,2,3-triol, 5ah

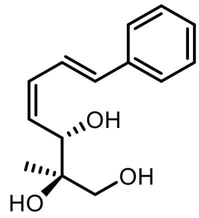

^{1}H (400 MHz, C_6D_6)

^{13}C (100 MHz, C_6D_6)

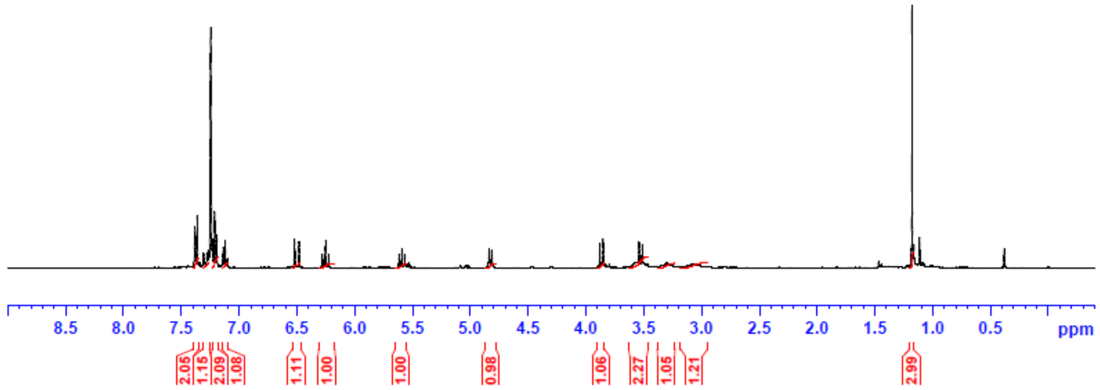

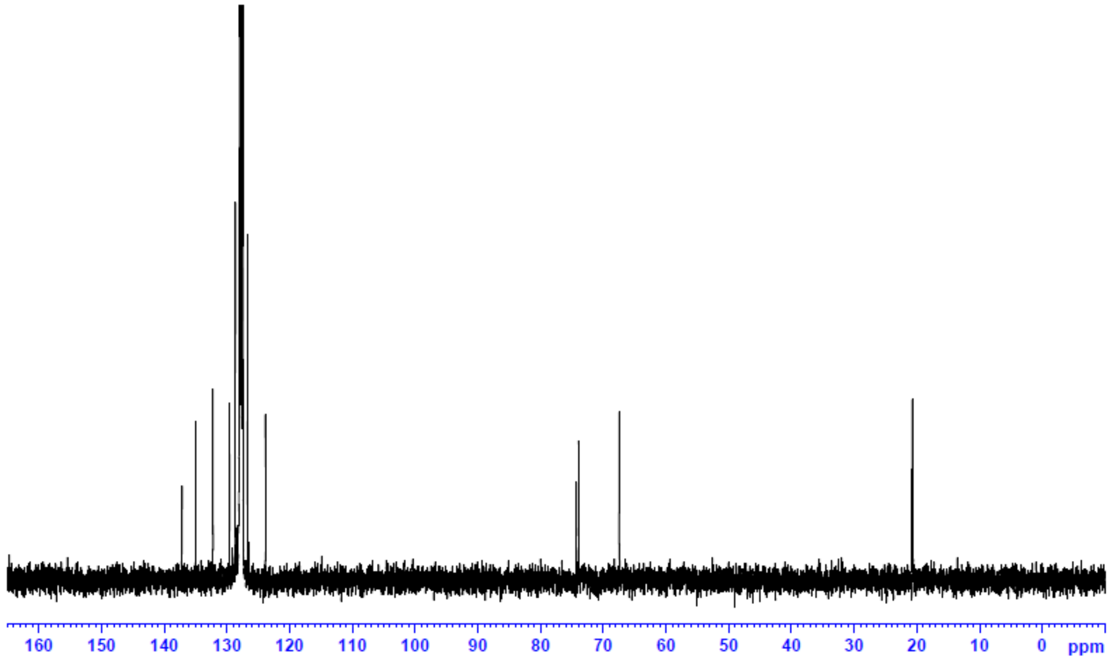

(5*Z*,7*E*)-1-((4-Methoxybenzyl)oxy)-8-phenylocta-5,7-dien-3-ol, 5ao

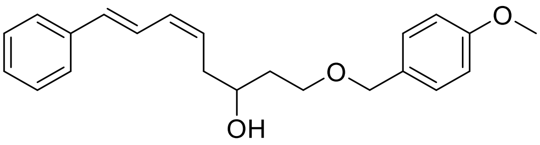

^{1}H (400 MHz, C_6D_6)

^{13}C (100 MHz, C_6D_6)

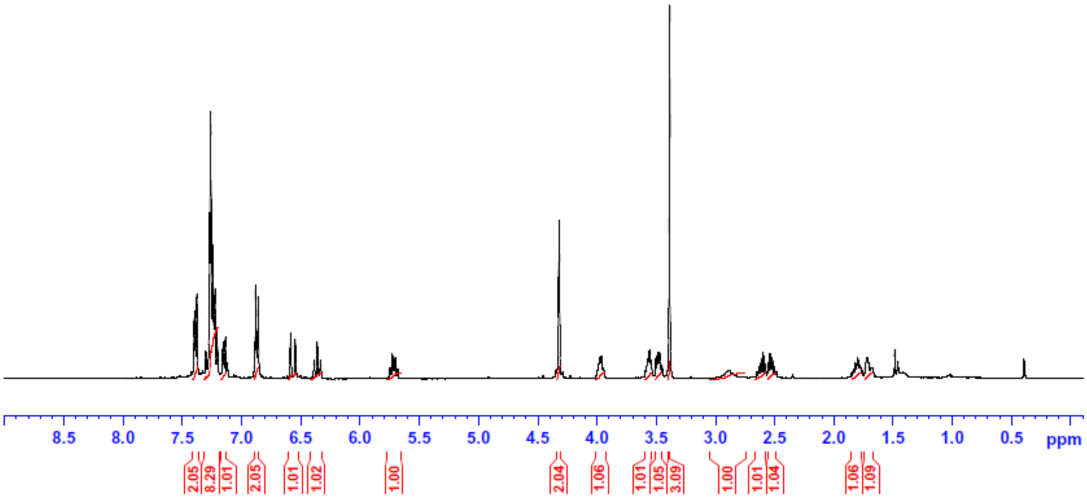

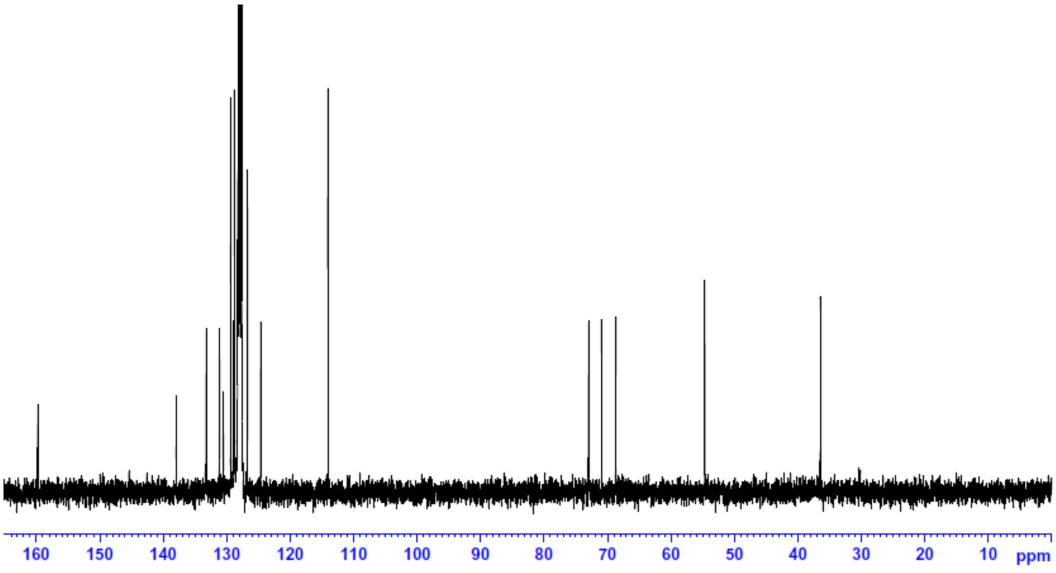

(*Z*)-1-((4-Methoxybenzyl)oxy)-6-phenylhex-5-en-3-ol, 5bo

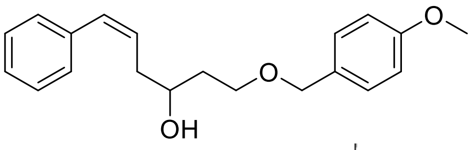

^{1}H (400 MHz, C_6D_6)

^{13}C (100 MHz, C_6D_6)

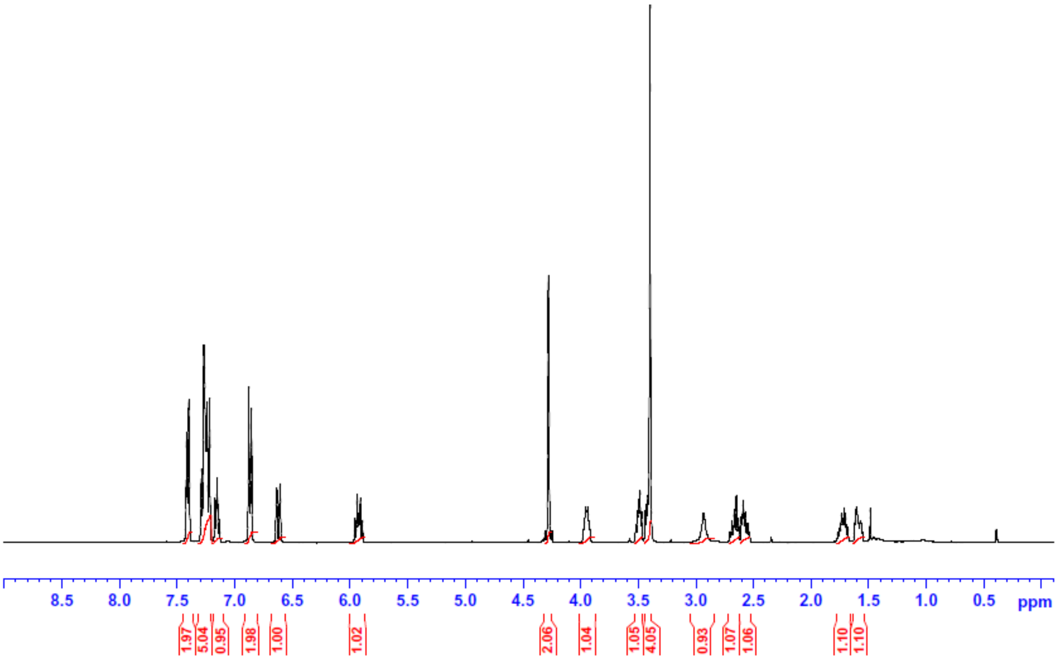

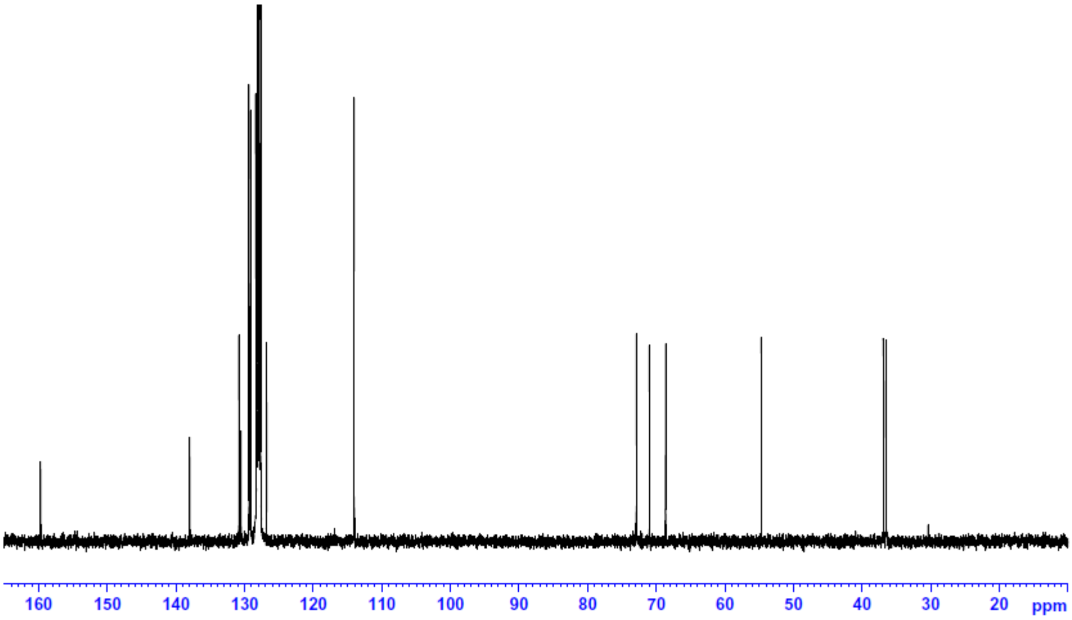

(*Z*)-5-((4-Methoxybenzyl)oxy)-1-phenylpent-1-en-3-ol, 5bf

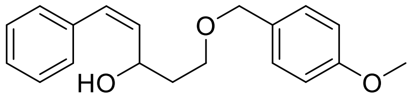

^{1}H (400 MHz, C_6D_6)

^{13}C (101 MHz, C_6D_6)

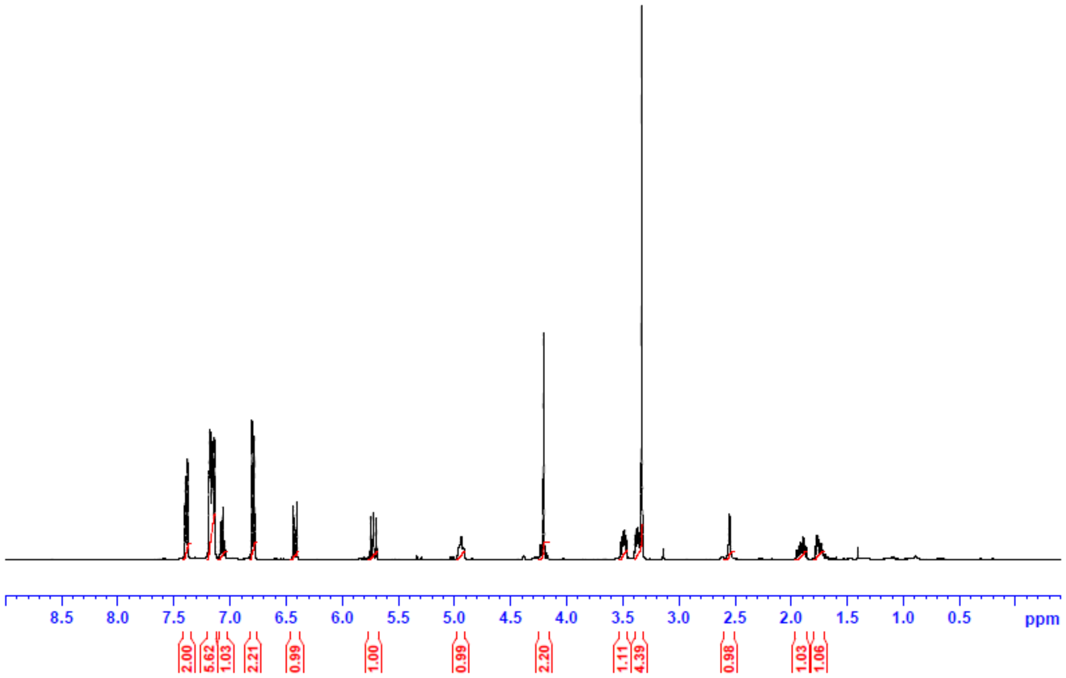

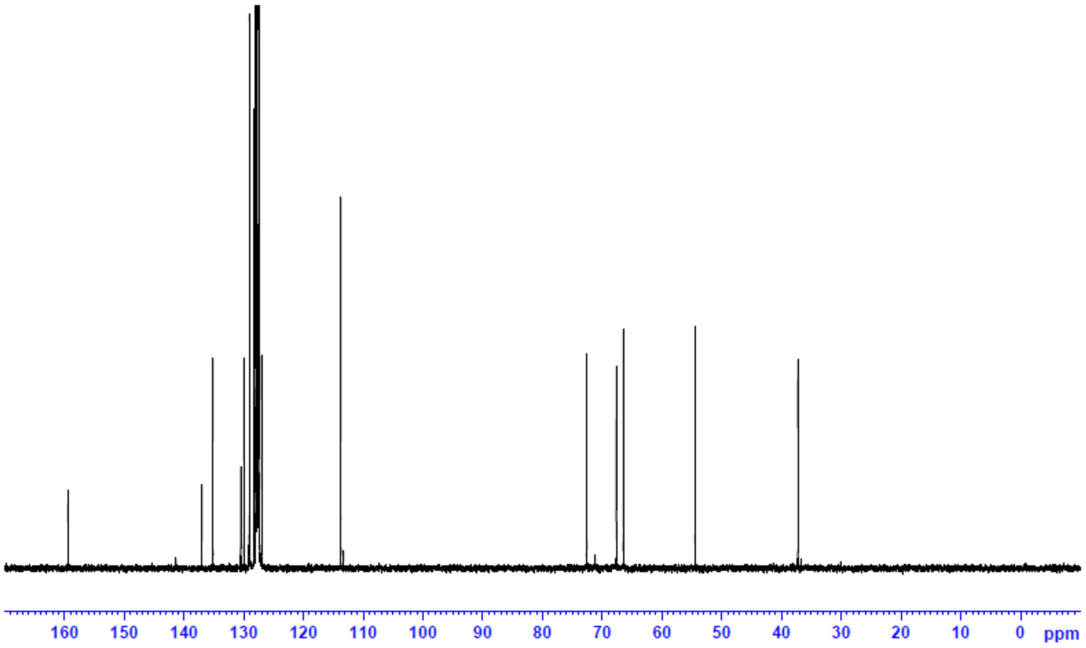

(4*Z*,6*E*)-1-((4-Methoxybenzyl)oxy)–7-phenylhepta-4,6-dien-3-ol, 5af

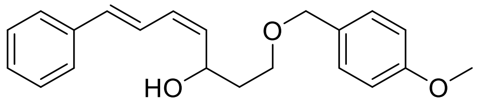

^{1}H (500 MHz, C_6D_6)

^{13}C (125 MHz, C_6D_6)

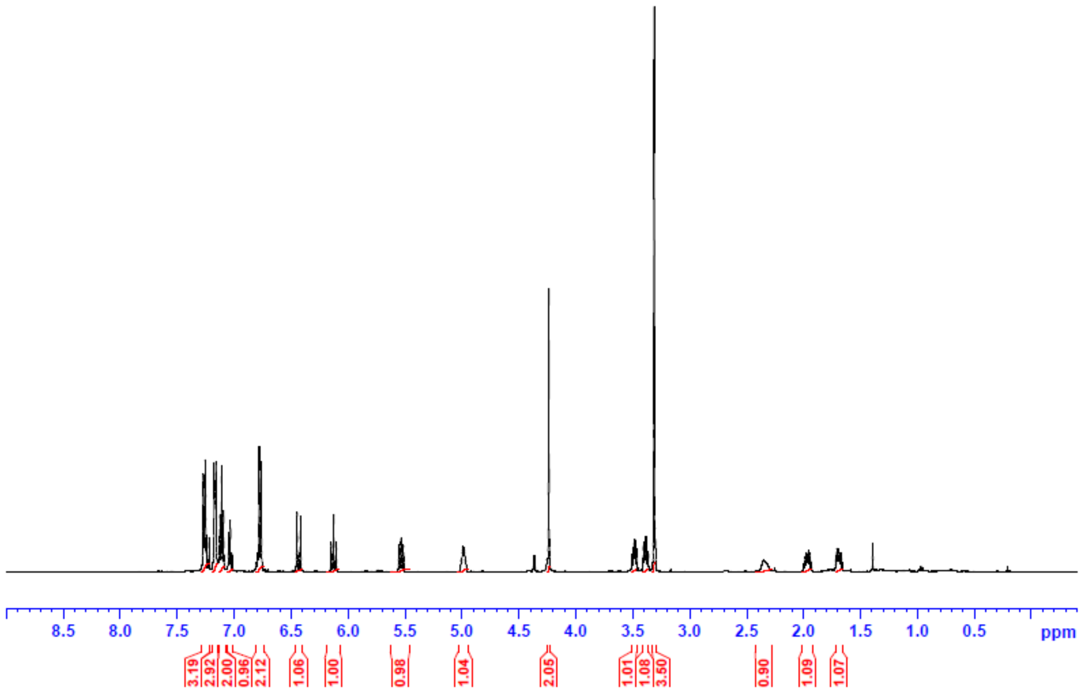

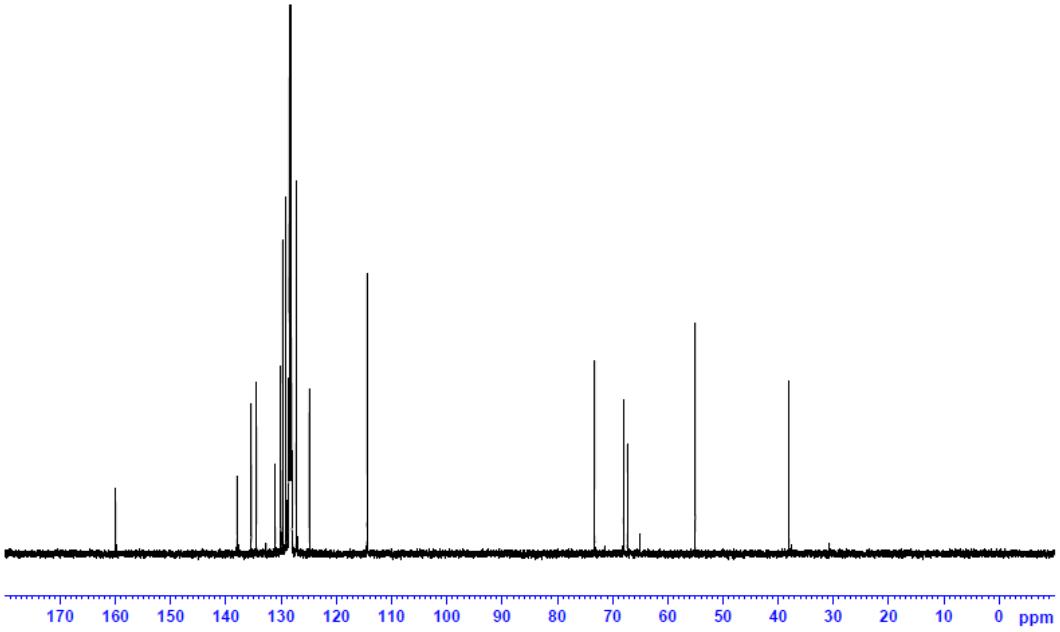

(*R*)-(2*E*,4*Z*,6*Z*)-10-((4-Methoxybenzyl)oxy)deca-2,4,6-triene-1,8-diol, 8

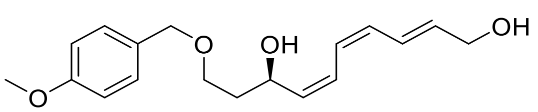

^{1}H (400 MHz, $CDCl_3$)

^{13}C (100 MHz, $CDCl_3$)

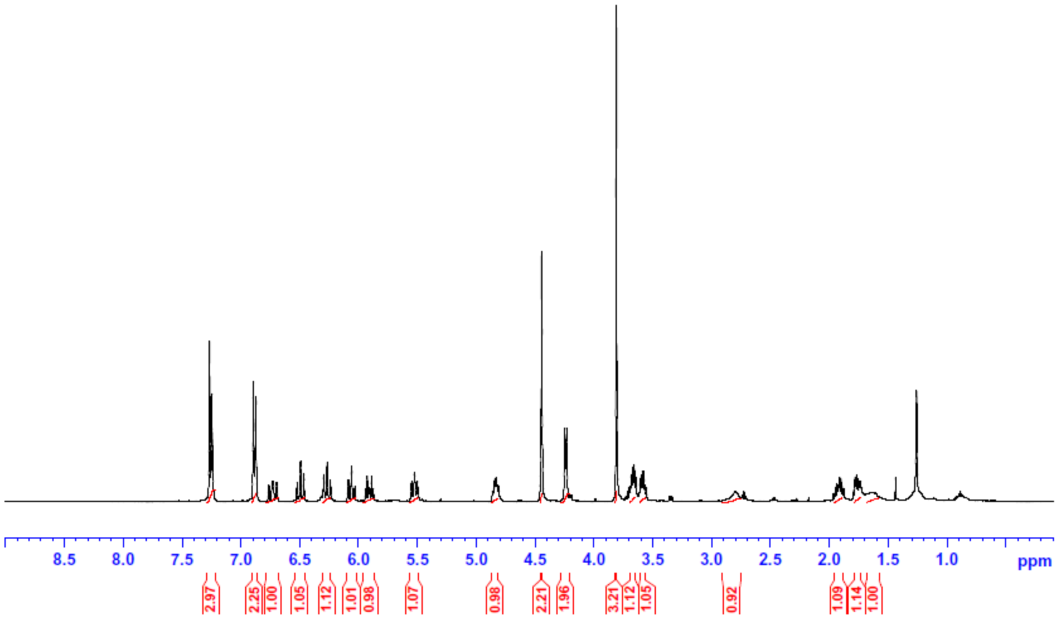

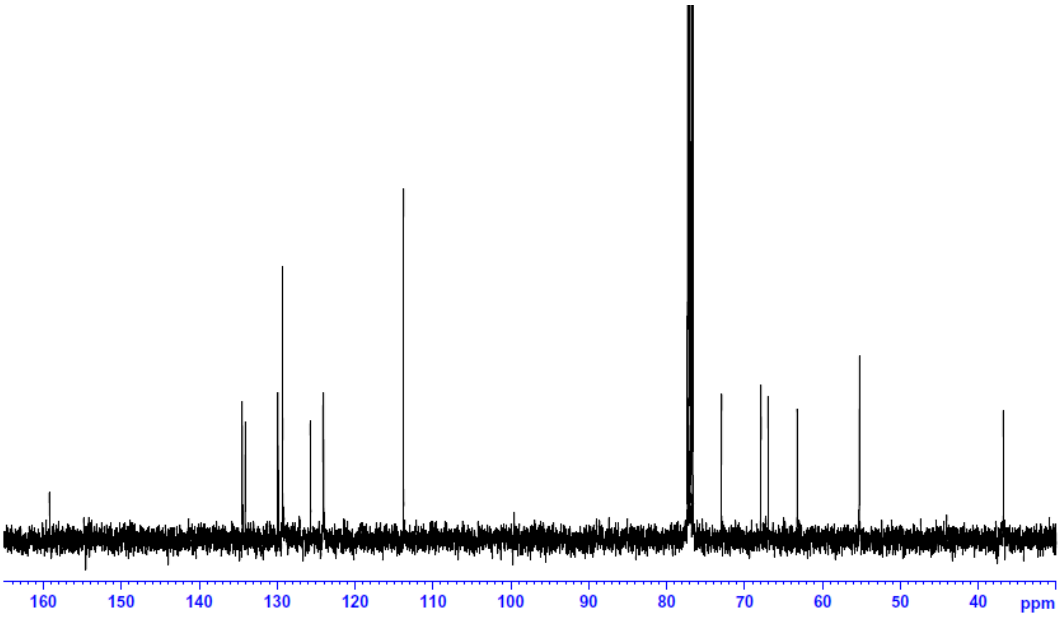

(*R*)-(4*Z*,6*Z*)–7-Cyclohexyl-1-((4-methoxybenzyl)oxy)hepta-4,6-dien-3-ol, 9

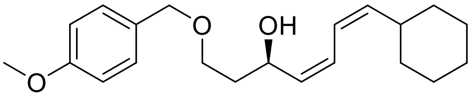

^{1}H (500 MHz, C_6D_6)

^{13}C (125 MHz, C_6D_6)

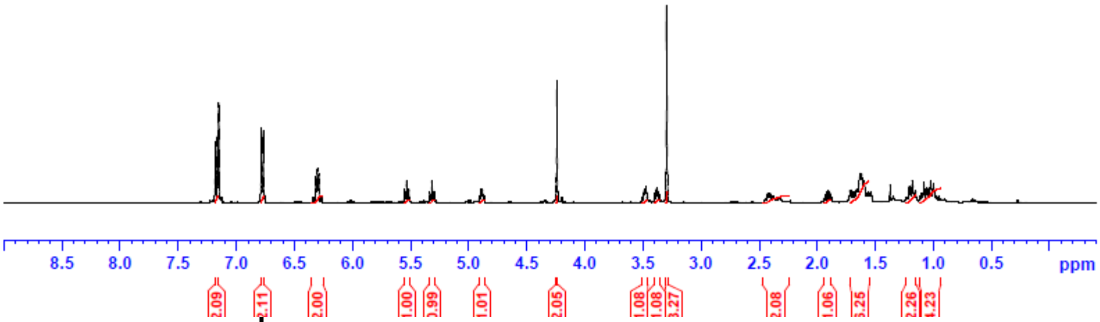

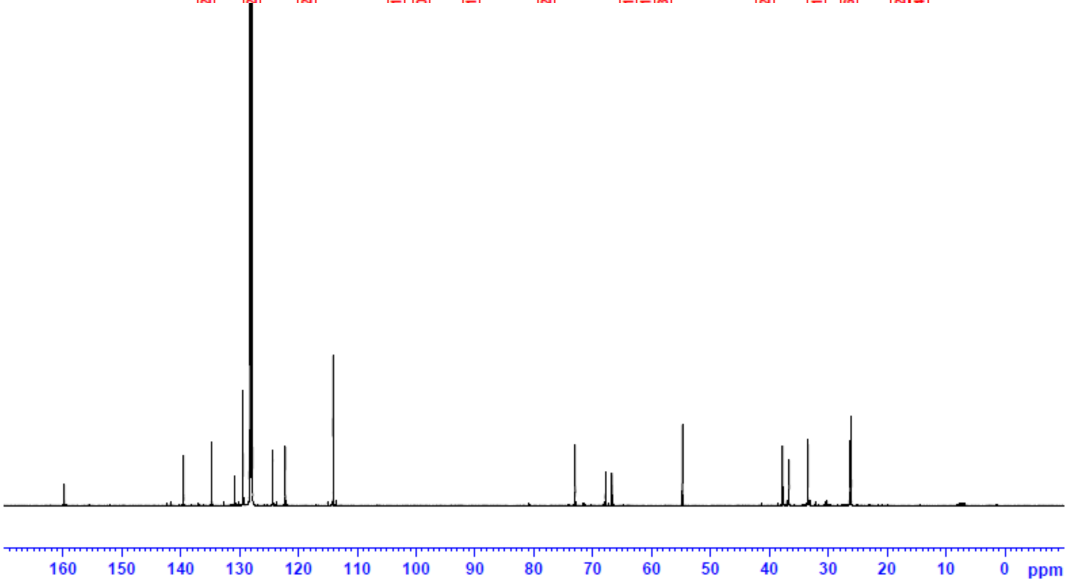

(*R*)-2-Chloro-3-((1*E*,3*Z*)-5-hydroxy–7-((4-methoxybenzyl)oxy)hepta-1,3-dien-1-yl)phenol, 10

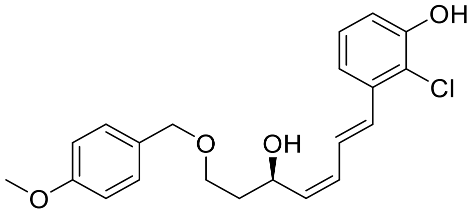

^{1}H (400 MHz, C_6D_6)

^{13}C (125 MHz, C_6D_6)

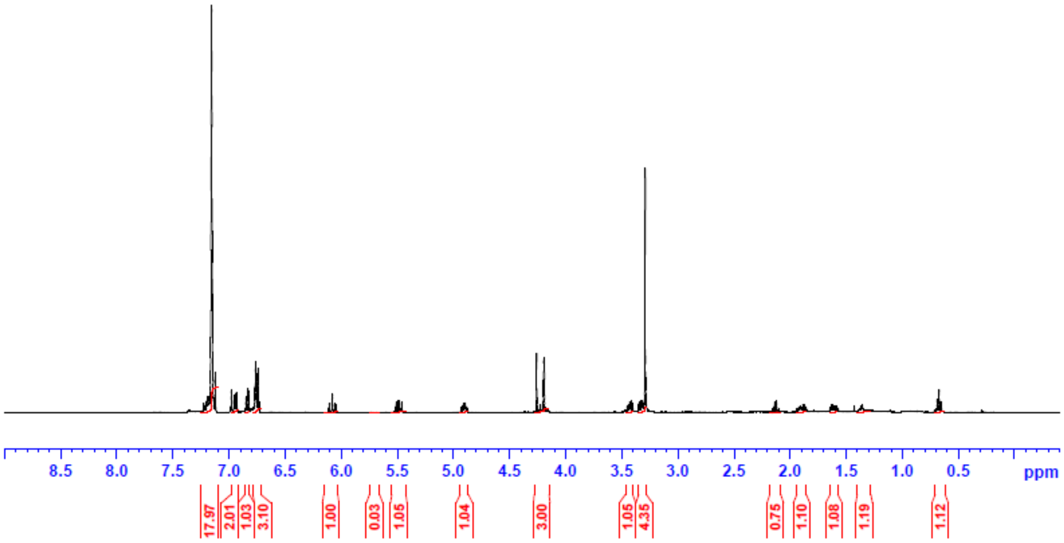

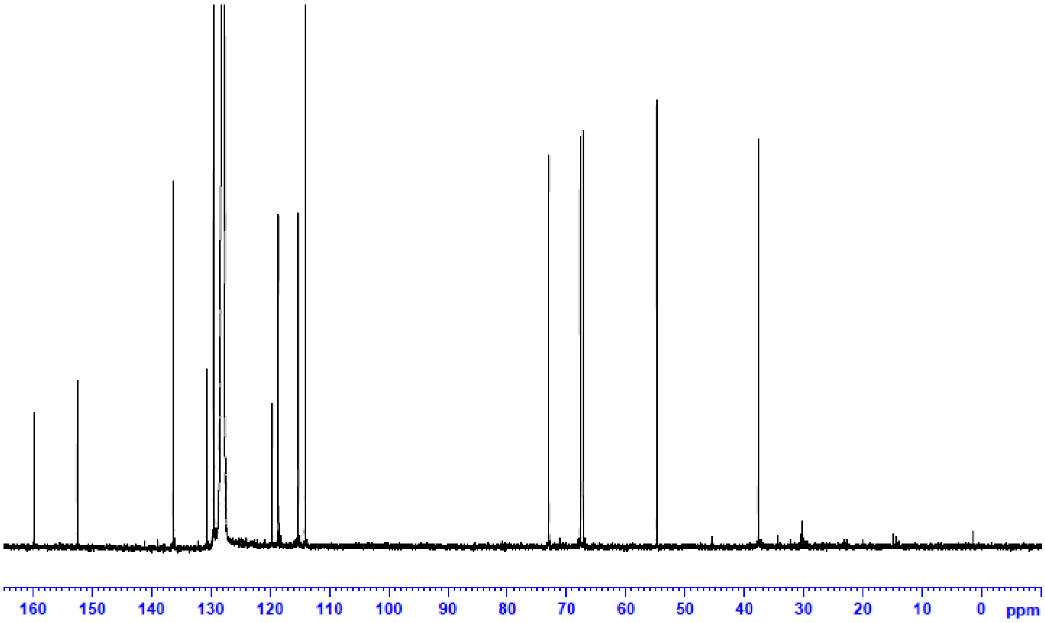

1-Methoxy-4-((((1*E*,3*Z*)-1-phenylundeca-1,3-dien-5-yl)oxy)methyl)benzene, 5az

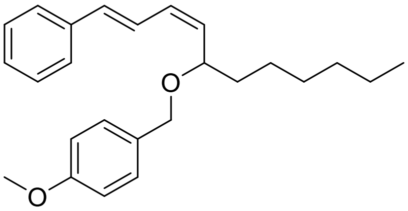

^{1}H (500 MHz, $CDCl_3$)

^{13}C (125 MHz, $CDCl_3$)

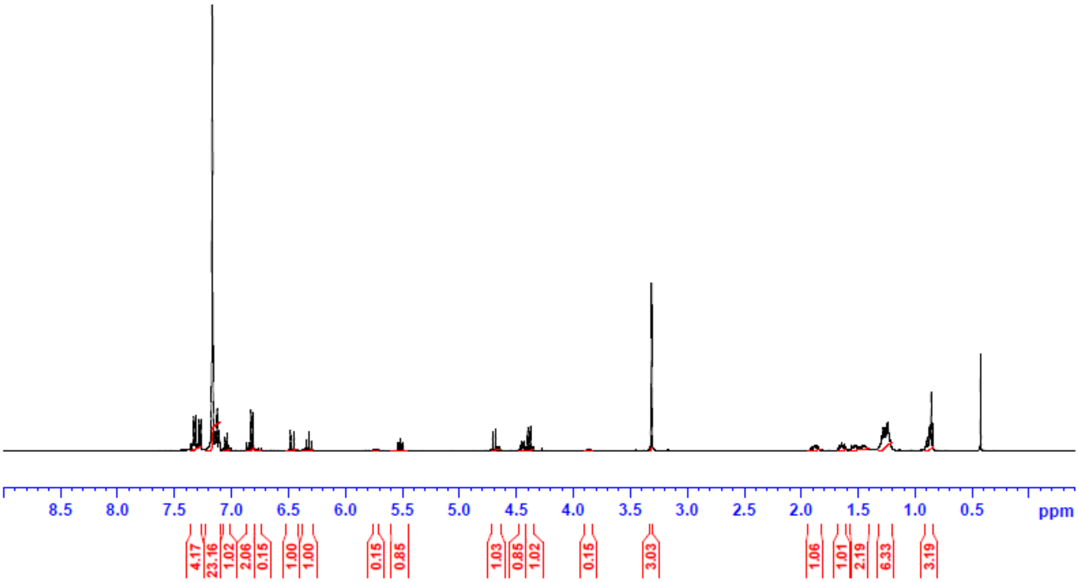

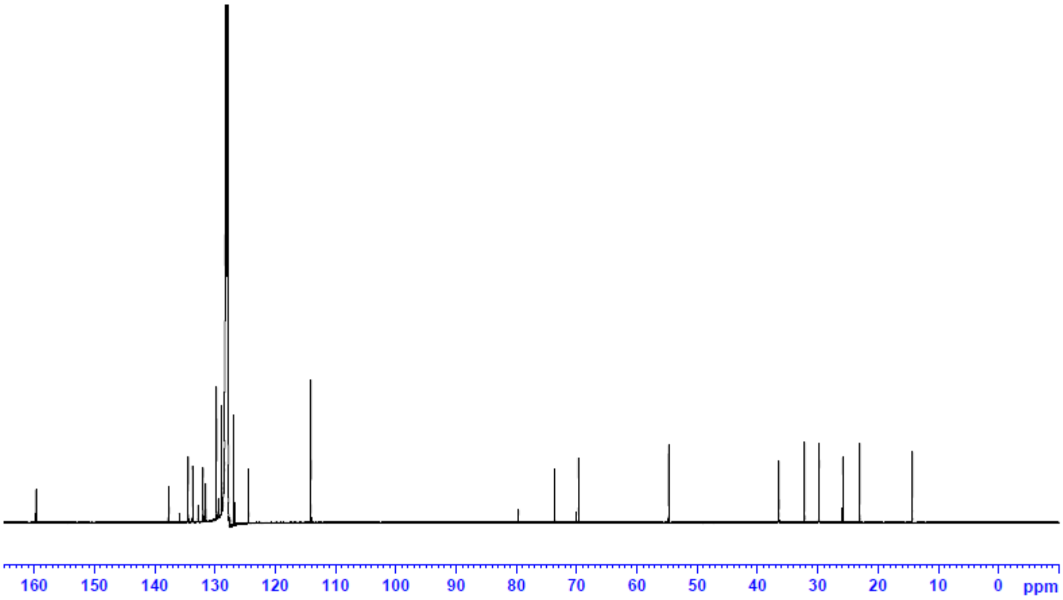

3. References

1. Yamaguchi, M.; Hirao, I., *Tetrahedron Lett.,* **1983**, *24*, 391-394.

2. Beignet, J.; Jervis, P. J.; Cox, L. R., *J. Org. Chem.* **2008**, *73*, 5462-5475.

3. Voronkov, M. G.; Kashik, T. V.; Deriglazova, E. S.; Lukevits, E. Y.; Pestunovich, A. E.; Cturkovich, R. Y., *Zh. Obschch. Khim.* **1975**, *45*, 2194-2200.

4. Lou, S.; Westbrook, J. A.; Schaus, S. E., *J. Am. Chem. Soc.* **2004**, *126*, 11440-11441.

5. Fernandes, R. A.; Chavan, V. P. *Tetrahedron: Asymmetry* **2011**, *22*, 1312-1319.

6. Gieseler, M. T.; Kalesse, M., *Org. Lett.*, **2011**, *13*, 2430–2432.

7. Kretschmer, M.; Menche, D., *Org. Lett.* **2012**, *14*, 382-385.

8. Hayashi, Y.; Yamaguchi, H.; Toyoshima, M.; Okado, K.; Toyo, T.; Shoji, M., *Chem. Eur. J.* **2010**, *16*, 10150-10159.

9. Matsumura, K.; Hashiguchi, S.; Ikariya, T.; Noyori, R., *J. Am. Chem. Soc.*, **1997**, *119*, 8738-8739.

10. Crimmins, M. T.; Jacobs, D. L., *Org. Lett.* **2009**, *11*, 2695-2698.

11. Dineen, T.A.; Roush, W.R., *Org. Lett.* **2003**, *5*, 4725-4728.

12. Nishizoro, N.; Akama, Y.; Agata, M.; Sugo, M.; Yamaguchi, Y.; Oda, K., *Tetrahedron* **2011**, *67*, 358-363.

13. Myers, A. G.; Lanman, B. A., *J. Am. Chem. Soc.* **2002**, *124*, 12969-12971.

14. Piccinini, A.; Kavanagh, S. A.; Connon, P. B.; Connon, S. J., *Org. Lett.* **2012**, *12*, 608-611.

15. Raheem, I. T.; Goodman, S. N.; Jacobsen, E. N., *J. Am. Chem. Soc.* **2004**, *126*, 706–707.

16. Tan, N. P. H.; Donner, C. D., *Tetrahedron,* **2009**, *65*, 4007-4012.

17. Barbazanges, M.; Meyer, C.; Cossy, J., *Org. Lett.* **2008**, *10*, 4489-4492.

18. Dubey, A.; Kauloorkar, S. V.; Kumar, P., *Tetrahedron* **2010**, *66*, 3159-3164.

19. Marshall, J. A.; Schaaf, G.; Nolting, A., *Org. Lett.* **2005**, *7*, 5331-5333.

20. Mori, K.; Shikichi, Y.; Shankar, S.; Yew, J. Y., *Tetrahedron*, **2010**, *66*, 7161–7168.

21. Bull, J. A.; Mousseau, J. J.; Charette, A. B., *Org. Lett.* **2008**, *10*, 5485-5488.

22. Li, H. J.; Guillot, R.; Gandon, V., *J. Org. Chem.* **2010**, *75*, 8435-8449.

23. Hu, D. X.; Clift, M. D.; Lazarski, K. E.; Thomson, R. J., *J. Am. Chem. Soc.*, **2011**, *133*, 1799-1804.

24. Spino, C.; Rezaei, H.; Dupont-Gaudet, K.; Bélanger, F., *J. Am. Chem. Soc.* **2004**, *126*, 9926-9927.

25. Trost, B. M.; Frederiksen, M. U.; Papillon, J.P.N.; Harrington, P. E.; Shin, S.; Shireman, B. T., *J. Am. Chem. Soc.* **2005**, *127*, 3666-3667.

26. Beshai, M.; Dhudshia, B.; Mills, R.; Thadani, A. N., *Tetrahedron Lett.* **2008**, *49*, 6794-6796.

27. Giles, R. G. F.; Green, I. R.; Li, S-H., *Aust. J. Chem.* **2005**, *58*, 565-571.

28. Teeter, H. M.; Bell, E. W., *Org. Synth.* **1952**, *32*, 20.

29. Trend, R. M.; Ramtohul, Y. K.; Stoltz, B. M., *Org. Lett*., **2005**, *127*, 17778-17788.

30. Huang, Z.; Negishi, E., *Org. Lett*. **2006**, *8*, 3675-3678.

31 Bourland, T. C.; Carter, R. G.; Yokochi, A. F. T. *Org. Biomol. Chem.* **2004**, *2*, 1315-1329.

32. Silva, M. S.; Comasseto, J. V.; Dos Santos, A. A., *Tetrahedron Lett.* **2010**, *51*, 5426-5429.
